# Genome-Wide Transcriptional Profile Analysis of *Prunus persica* in Response to Low Sink Demand after Fruit Removal

**DOI:** 10.3389/fpls.2016.00883

**Published:** 2016-06-22

**Authors:** Wei Duan, Hongguo Xu, Guotian Liu, Peige Fan, Zhenchang Liang, Shaohua Li

**Affiliations:** Beijing Key Laboratory of Grape Science and Enology and Key Laboratory of Plant Resources, Institute of Botany, The Chinese Academy of SciencesBeijing, China

**Keywords:** peach, low sink demand, photosynthesis, transcriptional profile, fruit removal

## Abstract

*Prunus persica* fruits were removed from 1-year-old shoots to analysis photosynthesis, chlorophyll fluorescence and genes changes in leaves to low sink demand caused by fruit removal (−fruit) during the final stage of rapid fruit growth. A decline in net photosynthesis rate was observed, accompanied with a decrease in stomatal conductance. The intercellular CO_2_ concentrations and leaf temperature increased as compared with a normal fruit load (+fruit). Moreover, low sink demand significantly inhibited the donor side and the reaction center of photosystem II. 382 genes in leaf with an absolute fold change ≥1 change in expression level, representing 116 up- and 266 down-regulated genes except for unknown transcripts. Among these, 25 genes for photosynthesis were down-regulated, 69 stress and 19 redox related genes up-regulated under the low sink demand. These studies revealed high leaf temperature may result in a decline of net photosynthesis rate through down-regulation in photosynthetic related genes and up-regulation in redox and stress related genes, especially heat shock proteins genes. The complex changes in genes at the transcriptional level under low sink demand provided useful starting points for in-depth analyses of source-sink relationship in *P. persica*.

## Introduction

Photosynthesis is the basis of plant growth and development, and it plays a decisive role in crop yield and quality. The fruit is the most important sink organ for most horticultural plants. The presence or absence of the fruits has a significant effect on source leaf photosynthesis in many plant species including peach (Duan et al., 2008). Therefore, fruit removal has often been used to change the sink–source relationship in order to study photosynthetic responses under low sink demand.

Leaf net photosynthesis rate (*P*_n_) was found to be reduced when the sink demand was lowered by removing fruits or flowers in herbaceous species such as potato (Basu et al., 1999), tomato (Walker and Ho, 1977), soybean (Setter et al., 1980), and Dahlia (Yan et al., 2011) as well as in woody plants such as grape (Downton et al., 1987), kiwifruit (Buwalda and Smith, 1990), apple (Gucci et al., 1995; Fan et al., 2010), citrus (Iglesias et al., 2002), coffee (DaMatta et al., 2008), peach (Li et al., 2005; Duan et al., 2008), and pine (López et al., 2015). A lot of studies support the hypothesis of end-product inhibition of photosynthesis to explain the response of the decline of *P*_n_ under the low sink demand (Paul and Foyer, 2001; Iglesias et al., 2002; Zhou and Quebedeaux, 2003; Wu et al., 2008). However, this conclusion is controversial (Li et al., 2007; DaMatta et al., 2008). In our previous work it was found that low sink demand increased leaf temperature (Li et al., 2001, 2005, 2007; Duan et al., 2008; Cheng et al., 2009; Fan et al., 2010). So we speculated high leaf temperature might cause irreversible damage to photosynthetic apparatus when it was above the optimum temperature of photosynthesis. To date, the specific mechanism for the effect of low sink demand on photosynthesis is unclear.

In order to understand the molecular basis of change in source-sink response, gene expression profiling using expressed sequence tags or microarray were carried out in some plant species. For example, leaf shading treatment in C_4_ plants such as sugarcane resulted in the up-regulation of several genes associated with photosynthesis, mitochondrial metabolism, and sugar transport (McCormick et al., 2008). cDNA microarray analysis in sugarcane showed that elevated CO_2_ levels modify the expression of genes related to photosynthesis and development (De Souza et al., 2008). Moreover, severely defoliated plants of perennial ryegrass showed increased abundance of photosynthesis-related gene transcripts (Lee et al., 2011). Changes in gene expression due to sink removal in soybean leaves were monitored using an oligonucleotide microarray in combination with targeted metabolite profiling (Turner et al., 2012). However, the genes related to metabolism and the selected signature genes showed diverse profiles in the above mentioned studies. Therefore, there is a lack of systematic analysis of changes in leaf gene expression under the source-sink regulation.

In this study, we studied the changes in photosynthesis and chlorophyll fluorescence parameters in *P. persica* leaves under normal sink demand and low sink demand by fruit removal. Moreover, we performed deep sequencing analysis using the Solexa digital gene expression system to compare the differentially expressed genes in response to −fruit and +fruit. These sequencing datasets allowed us to comprehensively characterize the molecular basis of the physiological processes under low sink demand and gain insight for further research.

## Materials and methods

### Plant materials

In this study, we used 4-year-old peach “Zaojiubao” (mutant of “Okubo”) [*Prunus persica* (L.) Batch] trees, which have a mid-ripening peach with fruit maturity occurring in the middle of July. The trees were planted 2 m apart within rows and 5 m apart between rows. They were trained to “Y” training systems and pruned using the long pruning method in winter (Li et al., 1994).

### Treatments

During the final stage of rapid fruit growth (on 23 July 2010, about 85 days after full blossom), 1-year-old shoots located on the southwest and southeast sides of the tree in the outer part of the crown were used as the unit of sink-source manipulation. Those 1-year-old shoots with similar light exposure were selected according to their uniformity in length (40–50 cm) and growth status (at least one new shoot per 1-year-old shoot). Each selected 1-year-old shoot, which supported one fruit and one new shoot, was considered a plot. Eight mature leaves were retained on each new shoot by topping and removing the smaller basal leaves. Half of the shoots from the previous season had fruits while the fruit were removed from the other half after sunset on 23 July 2010. Moreover, the export of assimilates from the treated and untreated parts, including the base and top parts of the 1-year-old shoots, was strictly controlled by girdling of the 1-year-old shoots. Twenty one-year-old shoots per treatment were selected for measurements of gas exchange and chlorophyll fluorescence, and leaves were sampled from 12 one-year-old shoots per treatment for the gene analyses.

### Measurement of photosynthetic gas exchange parameters

Photosynthetic gas exchange parameters including *P*_n_, stomatal conductance (*g*_s_), and intercellular CO_2_ concentration (*C*_i_) were measured using a Li-6400 portable photosynthesis system (Li-Cor Inc., Lincoln, NE, USA). The measurements were recorded between 0700 and 1800 h, on 25 July 2010, the 2nd day after initiating the source-sink manipulation on five leaves from each of five 1-year-old shoots per treatment. Photosynthetically active radiation (PAR), *g*_s_, transpiration rate (*E*), *C*_i_ and leaf temperature (*T*_leaf_) were obtained when *P*_n_ was measured.

### Chlorophyll a fluorescence kinetics transient analysis (OJIP-test)

The OJIP-test parameters were also measured on 25 July 2010, the same day as gas exchange measurement as Luo's methods (Luo et al., 2011). A Handy-Plant Efficiency Analyzer (Hansatech Instruments, King's Lynn, Norfolk, UK) was used for determine the fluorescence signals on the same leaves used for gas exchange measurements. The measurements were made after dark adaption for more than 15 min. The transients were induced by red light of about 3000 μmol m^−2^ s^−1^ provided by an array of six light emitting diodes (peak wavelength 650 nm). The fluorescence signals were recorded from 10 μs to 1 s with a data acquisition rate of 10 μs for the first 2 ms and every 1 ms thereafter. The following data from the original measurements were used: *F*_m_: maximal fluorescence intensity; *F*_k_: fluorescence intensity at 300 μs [required for calculation of the initial slope (*M*_o_) of the relative variable fluorescence (V) kinetics and W_k_]; *F*_j_: the fluorescence intensity at 2 ms (the J-step), *F*_i_: the fluorescence intensity at 30 ms (the I-step). The derived parameters were as follows: *F*_o_: fluorescence intensity at 50 μs. The parameter W_k_ on donor side of photosystem II (PSII), represents the damage to oxygen evolving complex (OEC), W_k_ = (*F*_k_ − *F*_o_)/(*F*_j_ − *F*_o_); the parameter RC_QA_ on reaction center of PSII, represents the density of Q_A_-reducing reaction centers, RC_QA_ = φ_Po_ × (*V*_j_/*M*_o_) × (ABS/CS); the parameter φ_*Po*_ on acceptor side of PSII, represents the maximum quantum yield of primary photochemistry at *t* = 0, φ_Po_ = *TR*o/ABS = 1 – *F*_o_/*F*_m_; the parameter φ_Eo_ on acceptor side of PSII, represents quantum yield for electron transport (at *t* = 0), φ_Eo_ = *ET*_o_/ABS = (*F*_m_– *F*_j_)/*F*_m_; the parameter ψ_Eo_ on acceptor side of PSII, represents the probability with which a trapped exciton moves an electron into the electron transport chain beyond ${\text{Q}}_{\text{A}}^{-}$, ψ_Eo_ = *ET*_o_/*TR*o = (*F*_m_ – *F*_j_)/(*F*_m_ – *F*_o_). The calculation and derivation of a range of new parameters from O-J-I-P transients is shown in Table S1. Five independent replicates were used in both treatments and controls respectively.

### Digital expression library construction and solexa sequencing

Leaves ware sampled at 1400 h on 25 July, the same day as gas exchange measurement. Total RNA was isolated from the pooled samples of three replicates with or without source-sink treatment, using plant total RNA isolation kit (Tiandz Inc.; Beijing, China). Gene Expression Sample Prep Kit (Illumina Inc.; San Diego, CA, USA) was used for sequence tag preparation according to the manufacturer's protocol. Six micrograms of total RNA were extracted and the mRNA was purified via Biotin-Oligo (dT) magnetic bead adsorption. First strand cDNA was synthesized with oligo (dT) on the bead. After second-strand cDNA synthesis, double strand cDNA was digested with NlaIII endonuclease producing a bead-bound cDNA fragment containing sequence from the 39-most CATG to the poly-A tail. These cDNA fragments were purified with magnetic bead precipitation and Illumina adapter 1(GEX adapter 1) was added to newly formed 5′ sticky end of cDNA fragments. The junction of GEX adapter 1 and CATG site was recognized by MmeI, which cuts 17 bp downstream of the CATG site, producing 17 bp cDNA sequence tags with GEX adapter 1. The 3′ fragments were removed using magnetic bead precipitation; and the Illumina adapter 2 (GEX adapter 2) was ligated to the new 3′ end of the cDNA fragment, which represented the tag library.

The cDNA fragments with GEX adapters 1 and 2 were subject to 15 cycles of linear PCR amplification by Phusion polymerase (Finnzymes, Espoo, Finland). The resulting 85 base fragments were purified by 6% TBE PAGE Gel electrophoresis. After double strand denaturation, the single chain molecules were fixed onto the Solexa Sequencing Chip (flow cell). Each molecule grew into a cluster sequencing template through *in situ* amplification, which represented a single tag derived from a single transcript. The sequencing was by the Beijing Genomics Institute (BGI, www.genomics.org.cn) using an Illumina HiSeq 2000 System. Four color-labeled nucleotides were added during sequencing; and the produced 49 bp sequences contained target tags and a 3′adaptor. Base-calling was performed using the Illumina Pipeline. After purity filtering and initial quality tests, the reads were sorted and counted for the following analysis. The clean reads data of −fruit and +fruit used in this manuscript have been uploaded respectively to SRA database at NCBI (accession numbers: SAMN05178616 and SAMN05178617).

### Sequence annotation

“Clean Tags” were obtained by trimming adapter sequences and filtering adaptor-only tags and low-quality tags (containing ambiguous bases) using the Fastx-toolkit (http://hannonlab.cshl.edu/fastx_toolkit). Sequence alignment was done with Bowtie 0.12.8 using the Peach Genome database (http://www.rosaceae.org/species/prunus_persica/genome_v1.0). All clean tags were annotated based on transcript sequences of peach reference genes, masked peach genome sequences (excluding the repeating sequences), and NCBI. For conservative and precise annotation, only sequences with perfect homology or one nucleotide mismatch were considered for further annotation.

### Identification of differentially expressed genes

Numbers of annotated clean tags for each gene were calculated after alignment and then normalized to TPM (tags per million clean tags) (AC't Hoen et al., 2008; Morrissy et al., 2009). The genes that had < 10 TPM in both +fruit and −fruit libraries were excluded first. The default value (tag number) of genes that not found in any of the libraries was one. Differentially expressed genes (DEGs) in −fruit as compared with +fruit were identified based on a rigorous algorithm (Audic and Claverie, 1997). *P*-value was used to test the authenticity of differential transcript accumulation (Audic and Claverie, 1997; Wu et al., 2010). In the *P*-value formula below, the total clean tag number of the +fruit library is noted as N1, and total clean tag number of −fruit library as N2; gene A holds x tags in +fruit and y tags in −fruit library. The probability of gene A expressed equally between two samples can be calculated with:

$$\begin{array}{l} \text{P}(\text{y|x})={\left(\frac{\text{N2}}{\text{N1}}\right)}^{\text{y}}\frac{(\text{x}+\text{y})!}{\text{x}!\text{y}!{\left(\text{1}+\frac{{\text{N}}_{2}}{{\text{N}}_{1}}\right)}^{\left(\text{x}+\text{y}+\text{1}\right)}} \end{array}$$

The Bonferroni corrected *P*-value was applied to control the false discovery rate (FDR) in the multiple comparison and analysis during the identification of DEGs (Benjamini et al., 2001). An :FDR < 0.001 and the absolute value of log_2_ ratio ≥1” was used as the threshold to determine the significance of gene expression differences. The differently expressed genes were categorized into functional groups and mapped using Mapman (version 3.5.1R2) according to the standard protocol (Usadel et al., 2009).

### Real-time PCR analysis

Total RNA was isolated using the same method as used for DGE analysis. Real-time PCR was carried out using three independent biological replicates each containing three technical replicates. First-strand cDNA was synthesized using Oligo (dT)_15_ (Sigma, Hamburg, Germany) and Superscript III Reverse Transcriptase (Invitrogen, Carlsbad, CA, USA). cDNAs were diluted 20 fold for use as template. Specific primer pairs of 10 transcripts were designed using Primer3 (v. 0.4.0; http://frodo.wi.mit.edu/) and shown in Table S2. Experiments were carried out using FastStart Universal SYBR Green Master (Roche Diagnostics, Mannheim, Germany) with SteopOneplus™ Real-Time PCR system (Applied Biosystems). Data were analyzed using qbase^PLUS^ software (http://www.biogazelle.com/products). Transcript levels were normalized against the peach reference glyceraldehyde-3-phosphate dehydrogenase (GAPDH) gene (ppa006087m; Forward primer: 5-GAAATTCGATTTGCATGAGC-3, Reverse primer: 5-CAATGCCATTCAAGCTAAGG-3) according to Tong et al. (2009). The fold change in mRNA expression was estimated using threshold cycles, by the △△CT method.

## Results

### Diurnal variations of photosynthetic parameters

**Figure 1:**
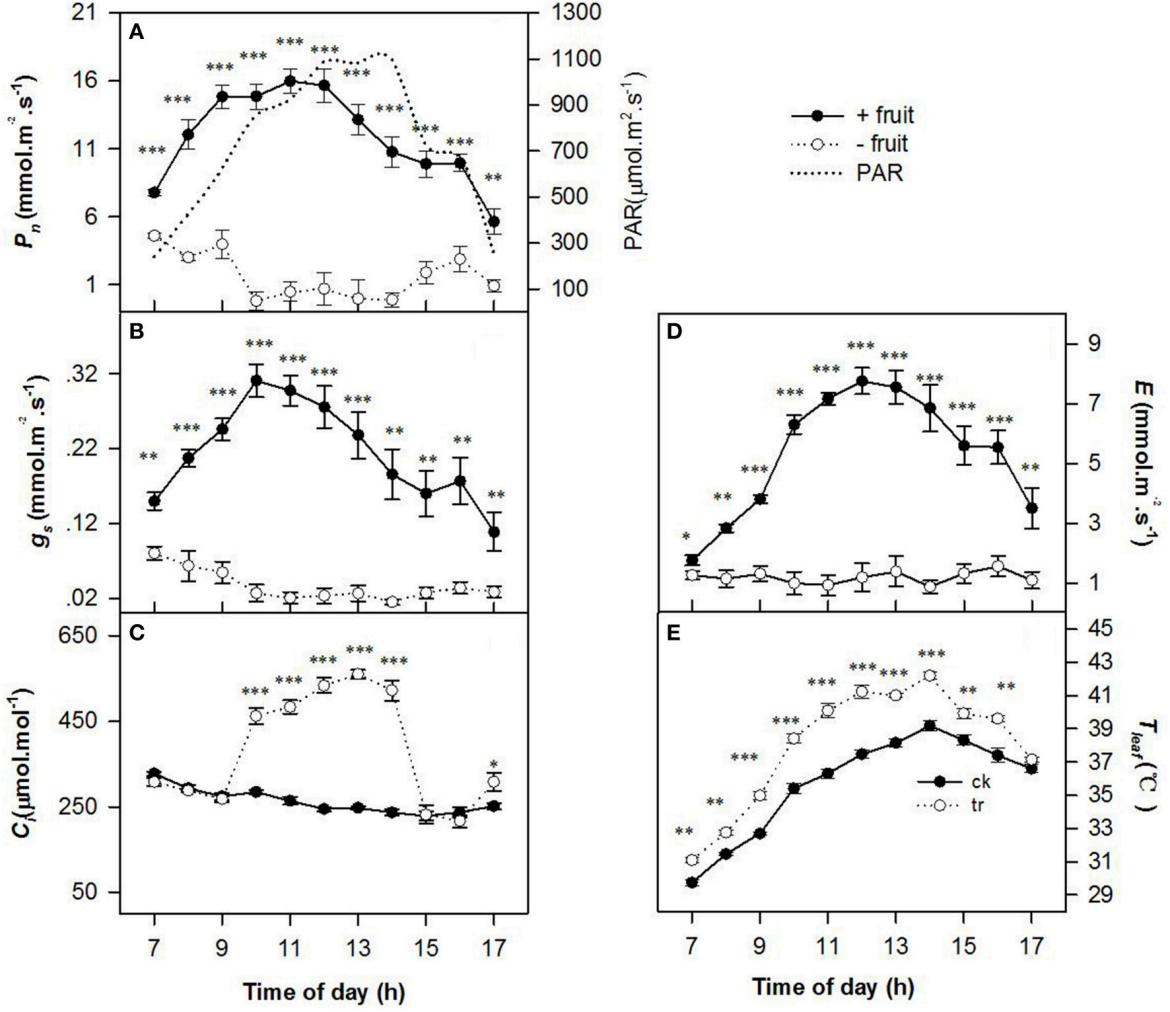
**Diurnal variation in gas exchange parameters, including net photosynthesis rate (***P***_**n**_) (A), stomatal conductance (***g***_**s**_) (B), intercellular CO_**2**_ concentration (***C***_**i**_) (C), transpiration rate (***E***) (D), and leaf temperature (***T***_**leaf**_) (E) in peach source leaves in response to low sink demand on the 2nd day after removing fruit**. The time course of PAR is given in **(A)**. Each value represents the mean ± *SE* of five replicates. The asterisks ^*^, ^**^, and ^***^ indicate significant differences between −fruit and +fruit at *P* < 0.05, 0.01, and 0.001, respectively.

Low sink demand had significant effects on the diurnal variations in *P*_n_, *g*_s_, *C*_i_, *E*, and *T*_leaf_ (Figure 1) on the 2nd day after removing fruit. Values of *P*_n_, *g*_s_, and *E* were gradually increased until 1100 h reached maximum when PAR about 1000 μ mol m^−2^ s^−1^, thereafter *P*_n_, *g*_s_, and *E* decreased slowly as PAR increased in +fruit shoots. The −fruit treatment significantly reduced *P*_n_, *g*_s_, and *E* throughout most of the day compared with the +fruit treatment (Figures 1A,B,D). At 1300 h, values of *P*_n_,*g*_s_, and *E* of −fruit were only 2.13, 11.05, and 1.38% of +fruit values respectively. *P*_n_ and *g*_s_ decreased to almost zero between 1000 h and 1400 h. Trends in *T*_leaf_ (Figure 1E) were similar to those in PAR. *T*_leaf_ reached the maximum (42.19°C) when PAR was about 1100 μ mol m^−2^ s^−1^ at 1400 h. Throughout most of the day *T*_leaf_ values in leaves of −fruit shoots were significantly higher than in leaves of +fruit shoots (Figure 1E). The pattern of diurnal change in *C*_i_, differed between the +fruit and −fruit treatments (Figure 1C). Maximal *C*_i_ occurred just after sunrise then decreased gradually in leaves in both treatments. Values of *C*_i_ decreased until the lowest value at 1100 h, and began to recover at 1600 h in the afternoon in +fruit, whereas it increased sharply at 0900 h, and high *C*_i_ was maintained between 1000 h to 1400 h in −fruit. Moreover, significantly higher *C*_i_ values were obtained in −fruit than in +fruit from 1000 until 1400 h.

### Diurnal variations of chl fluorescence parameters

**Figure 2:**
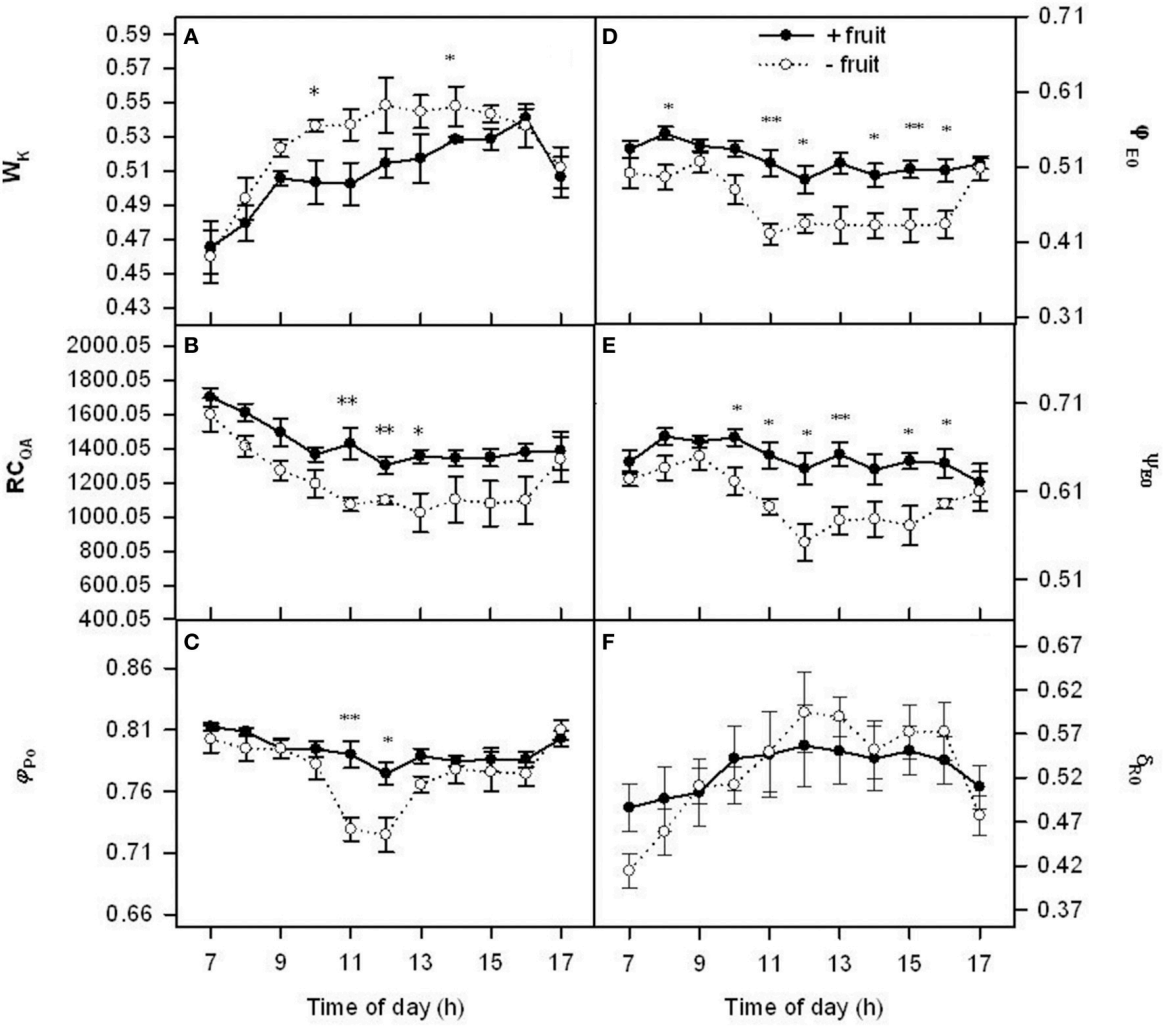
**Diurnal variations in donor side (Wk), reaction center (RC_**QA**_), acceptor side (φ_**Po**_, φ_**Eo**_, ψ_**Eo**_) (A–E) parameters of PSII and δ_**Ro**_ (the efficiency with an electron can move from plastoquinone (PQ) through PSII to the PSI end electron acceptor) (F) in peach source leaves in response to low sink demand on the 2nd day after removing fruit**. Each value represents the mean ± S.E. of five replicates. The asterisks ^*^, ^**^, and ^***^ indicate significant differences between −fruit and +fruit at *P* < 0.05, 0.01, and 0.001, respectively. The detailed meanings of W_k_, RC_QA_, φ_Po_, ψ_Eo_, φ_Eo_, and δ_Ro_ were shown in Table S1.

We further investigated the relationship between *P*_n_ decline and electron transport chain of PSII under the low demand by chlorophyll *a* fluorescence kinetics transient (OJIP-test). Wk had similar diurnal variation patterns in both −fruit and +fruit (Figure 2A). In the morning Wk increased progressively up to about 1300 h, and then they decreased. Parameters RC_QA_, φ_Po_, φ_Eo_, ψ_Eo_, and δ_Ro_ remained relatively stable throughout the day in +fruit plants, however they were at a maximum at 0700 h, then decreased progressively up to about mid-day, and remained at a low level in the afternoon (Figures 2B–E) except δ_Ro_ in −fruit plants. Low sink demand resulted in RC_QA_, φ_Po_, φ_Eo_, ψ_Eo_ about 24, 13, 16, 11% lower values, and W_k_ about 8% higher than +fruit at 1300 h respectively. Parameter δ_Ro_ signifies the redox state of photosystem I (PSI). However there was not significant differences in δ_Ro_ between −fruit and +fruit although lower in the beginning and the end of day and higher values around noon were observed in −fruit than in +fruit (Figure 2F).

### Digital expression libraries construction and tag sequencing

**Figure 3:**
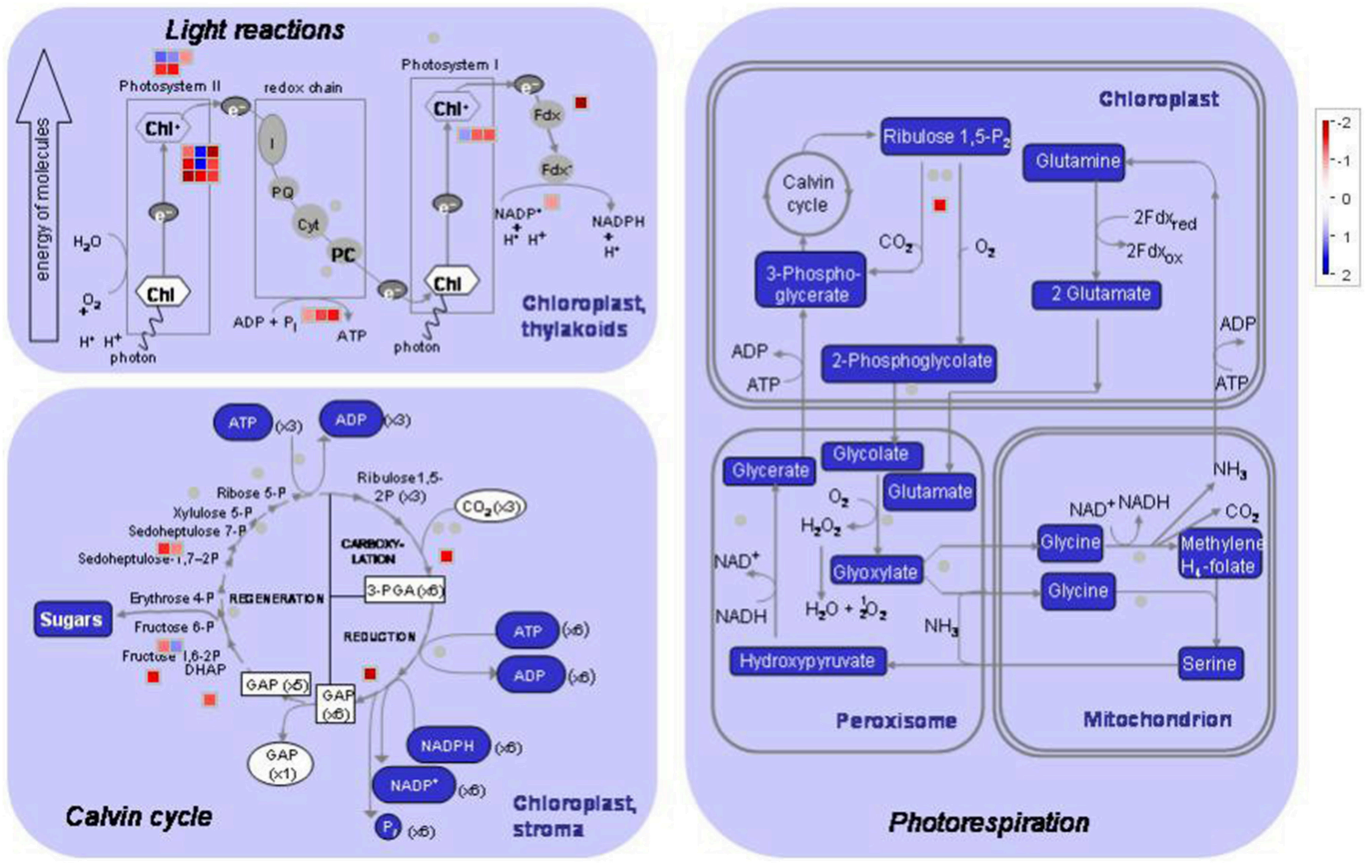
**MapMan visualization of photosynthesis in peach leaves under low sink demand**. Each square corresponds to a gene that is differentially regulated. Red indicates significant up-regulation while blue indicates down-regulation under low sink treatment. Only the genes that were significantly differentially expressed are represented in the MAPMAN figure.

Unique tags that perfectly matched reference genes in each library were normalized to tags per million clean tags (TPM) and used to evaluate the expression level of transcripts. The transcripts detected with at least two-fold differences in the two libraries are shown in Figure 3 (FDR < 0.001). The details of DEGs, including original TPM, fold-change, annotation, *P* value and FDR in both materials are shown in Table S3.

The distribution of unique tags with different copy numbers (clean tags) in +fruit and −fruit libraries were counted (Table 1). A total of 6,039,500 and 5,857,099 raw tags were sequenced in +fruit and −fruit libraries, including 247,102 and 243,331 distinct tags, respectively. Low quality tags and virus contaminations were filtered, and single-copy tags were excluded after which 118,192 and 104,826 distinct tags were obtained in each library. The majority of clean tags (about 82% from each library) were present in low copy numbers (< 10 copies), and ~10% tags from each library were counted between 11 and 100 times. Approximately, 3.3% tags were detected more than a 100 times.

**Table 1:** **Distribution of the sequenced tags from libraries of peach leaves under normal sink demand (+fruit) or low sink demand (−fruit)**.

	**+fruit**	**−fruit**
Total tags	6,039,500	5,857,099
Clean tags	5,902,114	5,709,974
Total number of distinct tags	247,102	243,331
Unique tag	118,192	104,826
Tag copy muber < 2 (clean tag)	121,198(49.05%)	130,727(53.72%)
2–5	67,343 (27.25%)	57,105 (23.47%)
6–10	15,889 (6.43%)	14,452 (5.93%)
11–20	11,000 (4.45%)	10,247 (4.21%)
21–50	10,093 (4.08%)	9697 (3.99%)
51–100	5514 (2.23%)	5315 (2.18%)
>100	8353 (3.38%)	8010 (3.29%)

### Analysis of tag mapping

The sequencing saturation was analyzed in the two libraries based on the number of identified genes to estimate whether the sequencing depth was sufficient for the transcriptome coverage. The number of tags reached saturation when no new genes were detected (Figure S1). All samples reached a plateau shortly after four million tags or higher were sequenced in both +fruit and −fruit libraries. No new genes were identified as the tag number approached six million in both libraries indicating that the capacity of the two libraries had approached saturation.

To identify the genes corresponding to 118,192 and 104,826 meaningful tags in each library, an essential dataset containing 286,689 reference genes expressed in the peach genome from http://www.rosaceae.org/node/355 was used. Altogether, 270,059 genes (94.32%) have the CATG sites, resulting in a total number of 147,813 unambiguous reference tags. By assigning the experimental Solexa tags to the virtual reference ones (Table S4), we observed that 44,173(37.4%) and 37, 007 (35.3%) tags were perfectly matched to +fruit and −fruit libraries respectively for the reference genes. Moreover, ~18% tags in the two libraries were mapped to the antisense strands suggesting that those regions might be directionally transcribed.

Altogether, there were 52,347 (44.3%) tags in the +fruit library and 43,863 (41.8%) tags in the −fruit library were found to match the annotated reference genes. The unmatched tags were then blasted against the peach genome, and ~40% tags were matched to the genomic sequences in the two libraries. As a result of the significant sequencing depth of Solexa technology and incomplete annotation of the peach genome. However, there were 19.3 and 17.8% unmatched tags in each library as result of the significant sequencing depth of Solexa technology and incomplete annotation of the peach genome.

### Function categories of differentially expressed genes

**Figure 4:**
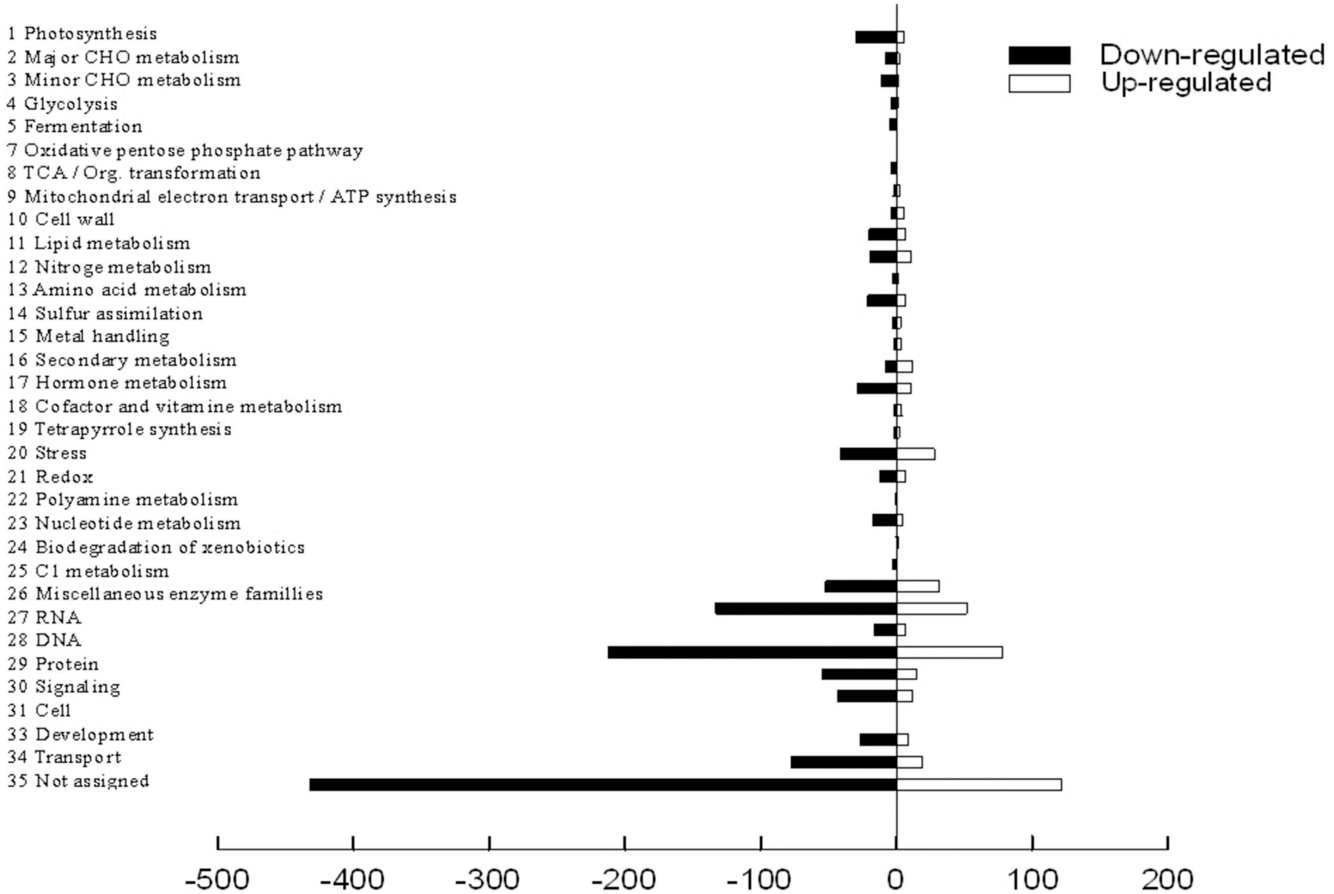
**Mapping and visualization of the differentially expressed genes in the leaves of ***P.persica*** under low sink demand using MAPMAN**. Black bars indicate down-regulated genes while red bars indicate up-regulated genes under low sink demand.

The functional classification of DEGs was further examined in peach to investigate the pattern of transcriptome regulation that occurred under the low sink demand. These genes were found to cover a lot of functions by using MapMan functional categories. Thereafter, the 1765 differently expressed proteins were classified into functional categories with the exception of 554 genes that were not assigned to any groups (Figure 4). The main categories included protein (16.4%), RNA (10.5%), and transport (5.4%). Miscellaneous enzyme families, signaling, stress, cell, hormone metabolism, development, and photosynthesis categories each accounted for 2.0–5.0% of the DEGs. Each of the other categories accounted for < 2% of DEGs. Full datasets are available online in Table S3. The photosynthesis related genes were regulated by source-sink relationship treatment.

As regards genes related to photosynthesis, a total of 25 genes were down-regulated under low sink demand (Table 2, Figure 3). 17 genes were involved in the light reaction. Among these groups, one oxygen-evolving complex-related gene was severely inhibited. The expression levels of LHCB3 and LHCA2 were inhibited under low sink demand. However, the expression of LHCB6 and LHC2.1 increased in the expression of genes. The PsaO subunit of PSI is declined under low sink demand. PPL2 (PsbP-like protein 2), PsbP, PsbY, and thylakoid lumenal 19 kDa protein were repressed while PsbR increased under low sink demand. In the Calvin cycle, seven genes included seduheptulose bisphosphatase (SBPase), fructose-1,6-bisphosphatase (FBPase), aldolase, triose-phosphate isomerase (TPI), glyceraldehyde-3-phosphate dehydrogenase B subunit (GAPB), and Rubisco activase (RCA) were severely repressed. Only RCA was down-regulated in photorespiration.

**Table 2:** **The list of genes photosynthesis regulated under low sink demand, based on MapMan functional categories**.

**Biological process**		**Accession number**	**Fold change**	**Bin**	**Species**	**Annotation**
Light reaction	PSII	XP_002298178.1	1.27	1.1.1.1	*Populus trichocarpa*	Light-harvesting complex II protein Lhcb6
		AAC34983.1	1.09	1.1.1.1	*Prunus persica*	Light harvesting chlorophyll a/b binding protein
		XP_002510744.1	–1.05	1.1.1.1	*Ricinus communis*	Chlorophyll a/b binding protein, Lhcb7
		XP_002525758.1	–1.48	1.1.1.1	*Ricinus communis*	Chlorophyll a/b binding protein, Lhcb3
		XP_002299309.1	–1.56	1.1.1.1	*Populus trichocarpa*	Light-harvesting complex I protein Lhca2
		NP_565906.1	–1.23	1.1.1.2	*Arabidopsis thaliana*	PPL2 (psbp-like protein 2); calcium ion binding
		ADB93062.1	1.57	1.1.1.2	*Jatropha curcas*	Chloroplast photosystem II 10 kDa polypeptide
		XP_002526766.1	–1.97	1.1.1.2	*Ricinus communis*	Thylakoid lumenal 19 kDa protein, chloroplast precursor, putative
		XP_002521576.1	–1.71	1.1.1.2	*Ricinus communis*	Oxygen-evolving enhancer protein 2, chloroplast precursor, putative
		XP_002512708.1	1.66	1.1.1.2	*Ricinus communis*	Photosystem II 11 kDa protein precursor, putative
		XP_002515034.1	–1.39	1.1.1.2	*Ricinus communis*	Photosystem II core complex proteins psbY, chloroplast precursor
		NP_196706.2	–2.49	1.1.1.2	*Arabidopsis thaliana*	PsbP domain-containing protein 5
		AAM61552.1	–1.69	1.1.1.2	*Arabidopsis thaliana*	Thylakoid lumen protein, chloroplast precursor
		NP_563737.1	–1.42	1.1.1.2	*Arabidopsis thaliana*	Photosystem II D1 precursor processing protein PSB27-H2
	PSI	AAO85557.1	1.00	1.1.2.2	*Nicotiana attenuata*	Photosystem I subunit XI
		BAA07667.1	–1.27	1.1.2.2	*Nicotiana sylvestris*	PSI-E subunit of photosystem I
		CAB75430.1	–1.32	1.1.2.2	*Nicotiana tabacum*	Putative 16kDa membraneprotein
	Redox chain	NP_565711.1	–1.01	1.1.4	*Arabidopsis thaliana*	ATP synthase protein I -related
		XP_002518477.1	–1.39	1.1.4.4	*Ricinus communis*	ATP synthase gamma chain 2, chloroplast, putative
		NP_194953.1	–1.56	1.1.4.9	*Arabidopsis thaliana*	ATP synthase family
		XP_002516617.1	–2.63	1.1.5.2	*Ricinus communis*	Electron carrier, putative
		XP_002533800.1	–1.03	1.1.5.3	*Ricinus communis*	Ferredoxin–NADP reductase, putative
Calvin cycle		ABK76304.1	–1.47	1.3.9	*Morus alba var. multicaulis*	Chloroplast sedoheptulose-1,7-bisphosphatase
		XP_002530415.1	–1.09	1.3.9	*Ricinus communis*	Sedoheptulose-1,7-bisphosphatase,chloroplast, putative
		ABW38330.1	–1.18	1.3.7	*Fragaria X ananassa*	Chloroplast fructose-1,6-bisphosphatase I
		ABW38331.1	1.08	1.3.7	*Fragaria X ananassa*	Chloroplast fructose-1,6-bisphosphatase II
		AAR86689.1	–1.66	1.3.6	*Glycine max*	Fructose-bisphosphate aldolase
		XP_002529248.1	–1.34	1.3.5	*Ricinus communis*	Triosephosphate isomerase, putative
		ABA86964.1	–1.89	1.3.4	*Glycine max*	Glyceraldehyde-3-phosphate dehydrogenase B subunit
		ADD60242.1	–1.67	1.3.13	*Glycine max*	Alpha-form rubisco activase
Photorespiration		ADD60242.1	–1.67	1.3.13	*Glycine max*	Alpha-form rubisco activase

### Confirmation of DEGs by real-time PCR analysis

Ten candidate genes that showed change in the pattern of expression in response to low sink demand were randomly selected from the peach DEGs for Real-time PCR analysis. Among them, six genes were up-regulated and four genes were down-regulated. The list of the genes and the comparison of fold changes between deep sequencing and Real-time PCR in +fruit and –fruit were shown in Table S5. The primers used for Real-time PCR of the selected genes are listed in Table S2. The Real-time PCR based expression patterns of all 10 selected genes showed a trend similar to that detected by the Solexa-sequencing method, which confirmed the reliability of our transcriptome analysis (Table S5).

## Discussion

Leaf transpiration and temperature play an important role on the source-sink relationship. Low sink demand by removing fruit or tuberous root sink resulted in significantly decreased *g*_s_ but increased *T*_leaf_ in higher plants (DaMatta et al., 2008; Duan et al., 2008; Wu et al., 2008; Fan et al., 2010; Yan et al., 2011). So Li et al. (2001) suggested that the decreased *g*_s_ may be considered as the trigger or promoter and increased *T*_leaf_ as the actor for regulating photosynthesis under a lower sink-source ratio. Low sink by fruit removal resulted in a decreased *P*_n_ with lower *g*_s_ and higher *T*_leaf_ in this study (Figure 1), which corroborates the results of previous studies in peaches or in other higher plants (Li et al., 2005; Fan et al., 2010; Yan et al., 2013). Moreover, significantly higher *C*_i_ was observed in –fruit than in +fruit (Figure 1C). In general, leaf *C*_i_ increases with a decrease in *g*_s_ and *P*_n_ when there is non-stomatal limitation in higher plant (Farquhar and Sharkey, 1982). Thus, the lower *P*_n_ under low sink demand in –fruit in this study was primarily due to non-stomatal limitation.

In OJIP-test, RC_QA_ shows the density of the of Q_A_-reducing PSII reaction centers. W_k_ is used as a specific indicator of damage to PSII donor side (Strasser, 1997), while φPo, φEo, and ψEo represent the acceptor side parameters of PSII. Low sink demand mainly resulted in a decrease in the acceptor side parameters φPo and ψEo of PSII and PSII reaction centers parameters RC_QA_ (Figure 2). These results in the present study were similar to the results obtained on bean at late stages after the removal of the sink of roots and pods plants (Yan et al., 2013). The *P*_n_ reduction could be attributed to essentially the probability that a trapped exciton moves an electron into the electron transport chain beyond ${\text{Q}}_{\text{A}}^{-}$ and φPo (Xiang et al., 2013).

Photosynthesis is one of the most heat sensitive processes and it can be completely inhibited by high temperature before other symptoms of the stress are detected (Berry and Bjôrkman, 1980). In this study 31 genes involved in the light reaction, Calvin cycle and photorespiration were down-regulated under low sink demand (Table 2, Figure 3). These changes in photosynthesis-related genes were similar to those observed in the application of a cold-girdle to C_4_ sugarcane (McCormick et al., 2008). Linear electron flow involves light-stimulated electron transfer between PSII and PSI, which stores the majority of photosynthetic energy. A total of 17 genes related to electron transfer were significantly down-regulated (Table 2), suggesting that the light reaction might be repressed by low sink demand. The repression electron transport causes the production of significant reactive oxygen species (ROS) early in the low sink response resulting in the inhibition of plant photosynthesis (Duan et al., 2008).

The light-harvesting complex (LHC) functions as a light receptor, and captures and delivers excitation energy to photosystems. LHCB3 serves as an intermediary in light energy transfer from the main LHCB1/LHCB2 antenna to the core of PSII (Standfuss and Kühlbrandt, 2004). In this study, the expression levels of LHCB7, LHCB3, and LHCA2 were inhibited under low sink demand. However, the expression of LHCB6 and LHC2.1 increased, indicating that they may be stable under low sink demand. The PsaO subunit of PSI is involved in balancing the excitation pressure between the two photosystems. Consistent with this observation, the levels of PsaE-2 and PsaO declined under low sink demand (Table 2).

PsbP (23 kD) is one of three extrinsic nuclear-encoded subunits of eukaryotic PSII oxygen-evolving complex (OEC). PsbR (10 kD) protein found in plant PSII plays a role in water oxidation (Roose et al., 2007). PsbY is one of the low molecular mass subunits of oxygen-evolving PSII (Kawakami et al., 2007). A PsbP-like protein 2 was previously shown to be essential for the accumulation of the chloroplast NAD(P)H dehydrogenase (NDH) complex (Ishihara et al., 2007). In the present study, PPL2 (PsbP-like protein 2), PsbP, PsbY, and thylakoid lumenal 19 kDa protein were repressed while PsbR increased under low sink demand. Moreover, the chlorophyll fluorescence parameter W_*k*_ also showed that the OEC of PSII was damaged under low sink demand.

In the Calvin cycle, seven genes (SBPase, FBPase, TPI, GAPB, RCA) were severely repressed involved in the reduction, regeneration, and carboxylation (Table 2). The repression of these genes suggested that these processes were negatively regulated by low sink demand. Only one gene was down-regulated in photorespiration indicating that most genes involved in photorespiration are not responsive to low sink demand. Arabidopsis plants growing for long periods under high CO_2_ resulted in a significant decrease in rbcL and rbcS transcripts, which encode the large and small subunits of Rubisco, respectively (Cheng et al., 1998).

In this study, nine Hsps were up-regulated under the low sink demand (Table 3). Most of them were belonged to one of the three major classes of molecular chaperones, HSP90, HSP70, and sHSPs. Four sHsps were up-regulated in low sink demand compared to control. In plants, sHsps have been reported to be involved in protecting macromolecules like enzymes, lipids, nucleic acid, and mRNAs from dehydration (Yamaguchi-Shinozaki et al., 2002). sHSPs are the most abundant and diverse HSPs produced at high temperatures (Palmblad et al., 2008). Furthermore, some sHSPs are also known to be induced by various abiotic stresses such as cold, salinity, drought, and chemical pollution (Palmblad et al., 2008). Proteins from the HSP70 family are essential for preventing aggregation and assisting re-folding of non-native proteins under stressing environmental conditions (Boston et al., 1996). HSP70 were accumulated under heat stress (Kosova et al., 2011; Liu et al., 2014). In this study, two members of the HSP70 family were up-regulated (Table 3). Hsp90 is one of the most common of the heat-related proteins. The majority of HSP90 known substrates are signal transduction proteins (Richter and Buchner, 2001), and it also uses a novel protein-folding strategy (Young et al., 2001). A putative HSP90 was up-regulated in *P. euphratica* at the early stage of heat stress. The –fruit treatment resulted in up-regulating two members of the HSP90 family (Table 3), which should play a role for preventing aggregation and assisting re-folding of non-native proteins. Therefore, we should say the Hsps may have important functions when the sink demand is low in *P. persica*.

**Table 3:** **The list of genes up-regulated of stress and redox under low sink demand, based on MapMan functional categories**.

**Accession number**	**Fold change**	**BIN**	**Species**	**Annotation**
**BIOTIC STRESS**				
ABA26457.1	3.70	20.10	*Citrullus lanatus*	Acidic class III chitinase
ACE80957.1	3.03	20.10	*Prunus dulcis X Prunus persica*	Allergen prup 2.01a, putative
ADM22305.1	2.93	20.10	*Prunus domestica*	Pathogenesis related protein 5
ACZ52964.1	2.21	20.10	*Dimocarpus longan*	Chitinase
ACM45716.1	1.31	20.10	*Pyrus pyrifolia*	Class IV chitinase
AAK82460.1	1.30	20.10	*Cinnamomum camphora*	Type 2 ribosome-inactivating protein cinnamomin III precursor
AAR28754.1	1.25	20.10	*Solanum lycopersicum*	Bax inhibitor
ACM45716.1	1.12	20.10	*Pyrus pyrifolia*	Class IV chitinase
ABC47922.1	1.07	20.10	*Malus X Domestica*	Pathogenesis-related protein 1a
XP_002519358.1	1.56	20.1.7	*Ricinus communis*	Leucine-rich repeat-containing protein, putative
**ABIOTIC STRESS**				
XP_002285199.1	2.10	20.20	*Vitis vinifera*	Spx domain-containing protein 2 isoform 1
XP_002318460.1	3.23	20.2.1	*Populus trichocarpa*	Heat shock 22k family protein
XP_006486450.1	3.18	20.2.1	*Citrus sinensis*	18.2 kDa class I heat shock protein-like
P30236.1	2.49	20.2.1	*Glycine max*	22.0 kDa class iv heat shock protein
NP_200076.1	1.94	20.2.1	*Arabidopsis thaliana*	Heat shock protein 90.1
EOX91407.1	1.82	20.2.1	*Theobroma cacao*	Heat shock factor 4
XP_002332067.1	1.72	20.2.1	*Populus trichocarpa*	Heat shock protein 70 cognate
CAA52149.1	1.28	20.2.1	*Cucumis sativus*	Heat shock protein 70
XP_004306709.1	1.26	20.2.1	*Fragaria vesca subsp. vesca*	Bag family molecular chaperone regulator 6-like
XP_002515568.1	1.19	20.2.1	*Ricinus communis*	Heat shock protein binding protein, putative
XP_002879575.1	1.18	20.2.1	*Arabidopsis lyrata subsp. lyrata*	DNAJ/Hsp40 heat shock N-terminal domain-containing protein
NP_178487.1	1.01	20.2.1	*Arabidopsis thaliana*	Heat shock protein 90
ADN33944.1	1.15	20.2.2	*Cucumis melo subsp. melo*	Cold-shock DNA-binding family protein
ADP30960.1	1.60	20.2.3	*Gossypium hirsutum*	Dehydration-induced 19-like protein
XP_002535200.1	1.93	20.2.99	*Ricinus communis*	Major latex protein, putative
XP_002864359.1	1.26	20.2.99	*Arabidopsis lyrata subsp. lyrata*	Pollen ole e 1 allergen and extensin family protein
ABD33344.1	1.18	20.2.99	*Medicago truncatula*	Pollen ole e 1 allergen and extensin
NP_850016.1	1.03	20.2.99	*Arabidopsis thaliana*	Rd2
**REDOX**				
AAD33596.1	1.77	21.10	*Hevea brasiliensis*	Thioredoxin H
XP_003517423.1	1.45	21.10	*Glycine max*	Thioredoxin-like 2, Chloroplastic-Like
CAH59452.1	1.44	21.10	*Plantago major*	Thioredoxin 3
NP_196046.2	1.06	21.10	*Arabidopsis thaliana*	WCRKC2 (WCRKC thioredoxin 2)
XP_002878810.1	1.57	21.20	*Arabidopsis lyrata subsp. lyrata*	Membrane-associated progesterone binding protein 2
XP_002869447.1	1.05	21.2.2	*Arabidopsis lyrata subsp. lyrata*	Gamma-glutamyl transpeptidase 3
CAD42908.1	2.41	21.60	*Prunus persica*	Catalase

Antioxidant enzymes play important roles in scavenging or reducing excessive ROS produced under stress conditions (Lee et al., 2007). Fruit removal remarkably increased the activities of antioxidant enzymes (Duan et al., 2008). However, only the antioxidant enzyme catalase (CAT) was up-regulated in our study. Thioredoxins are proteins that act as antioxidants by catalyzing thiol-disulfide interchange involved in the regulation of the redox environment in cells (Serrato et al., 2002; Gelhaye et al., 2005). Four thioredoxins (thioredoxin h, thioredoxin 3, thioredoxin 2, and thioredoxin 3-2) were up-regulated in our study (Table 3) suggesting that CAT and thioredoxin play an important role in maintaining redox homeostasis in *P. persica* cells under low sink demand.

## Conclusion

This study provided a global picture of gene changes in peach leaves under low sink demand using the Solexa digital gene expression system. Under low sink demand condition, net photosynthesis rate may be reduced due to increased leaf temperature, during which some genes related to the electron transport chain of photosynthesis and HSPs were differentially regulated. It helped to gain insight into how peach leave photosynthesis adapted to low demand.

## Author contributions

WD performed the experiments and wrote the manuscript. HX and GL helped perform the experiments and data analysis. ZL and PF helped design the experiment. SL designed the experiment and reviewed the manuscript. All authors have read and approved the final manuscript.

## Funding

This work is supported by the National Natural Science Foundation of China (No. 30800743 and 31071758).

### Conflict of interest statement

The authors declare that the research was conducted in the absence of any commercial or financial relationships that could be construed as a potential conflict of interest.

## Supplementary material

Figure S1 **Sequencing saturation analysis of peach leaf libraries under normal sink demand (+fruit) or low sink demand (−fruit)**.

Table S1 **Summary of parameters, formulae and their description using data extracted from chlorophyll a fluorescence transient (O-J-I-P-test)**.

Table S2 **List of primers used for the Real-time PCR**.

Table S3 **Detail information of peach DEGs under low sink demand**.

Table S4 **Alignment of the unique tags to the reference genome and annotated genes from peach leaves under normal sink demand (+fruit) or low sink demand (–fruit) peach trees**.

Table S5 **List of genes selected for Real-time PCR**.

## Abbreviations

CAT: catalase
*C*_i_: Intercellular CO_2_ concentrations
DEGs: Differentially expressed genes
*E*: Transpiration rate
ESTs: Expressed sequence tags
FBPase: Fructose-1,6-bisphosphatase
GAPB: Glyceraldehyde-3-phosphate dehydrogenase B
GAPDH: Glyceraldehyde-3-phosphate dehydrogenase
*g*_s_: Stomatal conductance
Hsp: Heat shock proteins
LHC: Light-harvesting complex
NDH: NAD(P)H dehydrogenase
OEC: oxygen-evolving complex
PAR: Photosynthetically active radiation
*P*_n_: Net photosynthesis rate
PQ: Plastoquinone
PSI: photosystem I
PSII: Photosystem II
RCA: Rubisco activase
ROS: reactive oxygen species
SBPase: Seduheptulose bisphosphatase
sHsps: small Hsps
*T*_leaf_: Leaf temperature
TPI: triose-phosphate isomerase
TPM: Tags per million clean tags.

## References

[B1] Audic S. Claverie J. M. (1997). The significance of digital gene expression profiles. Genome Res. 7, 986–995. 9331369 10.1101/gr.7.10.986

[B2] Basu P. S. Sharma A. Garg I. D. Sukumaran N. P. (1999). Tuber sink modifies photosynthetic response in potato under water stress. Environ. Exp. Bot. 42, 25–39. 10.1016/S0098-8472(99)00017-9

[B3] Benjamini Y. Drai D. Elmer G. Kafkafi N. Golani I. (2001). Controlling the false discovery rate in behavior genetics research. Behav. Brain Res. 125, 279–284. 10.1016/S0166-4328(01)00297-2 11682119

[B4] Berry J. Bjôrkman O. (1980). Photosynthetic response and adaptation to temperature in higher plants. Annu. Rev. Plant Physiol. 31, 491–543. 10.1146/annurev.pp.31.060180.002423

[B5] Boston R. S. Viitanen P. V. Vierling E. (1996). Molecular chaperones and protein folding in plants. Plant Mol. Biol. 32, 191–222. 10.1007/BF00039383 8980480

[B6] Buwalda J. G. Smith G. S. (1990). Effects of partial defoliation at various stages of the growing season on fruit yields, root growth and return bloom of kiwifruit vines. Sci. Hortic. 42, 29–44. 10.1016/0304-4238(90)90145-5

[B7] Cheng J. S. Fan P. G. Liang Z. C. Wang Y. Q. Niu N. Li W. D. (2009). Accumulation of end products in source leaves affects photosynthetic rate in peach via alteration of stomatal conductance and photosynthetic efficiency. J. Am. Soc. Hortic. Sci. 134, 667–676.

[B8] Cheng S. H. Moore B. Seemann J. R. (1998). Effects of short- and long-term elevated CO_2_ on the expression of Ribulose-1,5-bisphosphate carboxylase/oxygenase genes and carbohydrate accumulation in leaves of *Arabidopsis thaliana* (L.) Heynh. Plant Physiol. 116, 715–723. 10.1104/pp.116.2.715 9489018 PMC35131

[B9] DaMatta F. M. Cunha R. L. Antunes W. C. Martins S. C. Araujo W. L. Fernie A. R.. (2008). In field-grown coffee trees source–sink manipulation alters photosynthetic rates, independently of carbon metabolism, via alterations in stomatal function. New Phytol. 178, 348–357. 10.1111/j.1469-8137.2008.02367.x 18266616

[B10] De Souza A. P. Gaspar M. Da Silva E. A. Ulian E. C. Waclawovsky A. J. Nishiyama-Jr M. Y.. (2008). Elevated CO_2_ increases photosynthesis, biomass and productivity, and modifies gene expression in sugarcane. Plant Cell Environ. 31, 1116–1127. 10.1111/j.1365-3040.2008.01822.x 18433443

[B11] Downton W. J. S. Grant W. J. R. Loveys B. R. (1987). Diurnal changes in the photosynthesis of field-grown grape vines. New Phytol. 105, 71–80. 10.1111/j.1469-8137.1987.tb00111.x 33874032

[B12] Duan W. Fan P. G. Wang L. J. Li W. D. Yan S. T. Li S. H. (2008). Photosynthetic response to low sink demand after fruit removal in relation to photoinhibition and photoprotection in peach trees. Tree Physiol. 28, 123–132. 10.1093/treephys/28.1.123 17938121

[B13] Fan P. G. Li L. S. Duan W. Li W. D. Li S. H. (2010). Photosynthesis of young apple trees in response to low sink demand under different air temperatures. Tree Physiol. 30, 313–325. 10.1093/treephys/tpp114 20071359

[B14] Farquhar G. D. Sharkey T. D. (1982). Stomatal conductance and photosynthesis. Annu. Rev. Plant Physiol. 33, 317–345. 10.1146/annurev.pp.33.060182.001533

[B15] Gelhaye E. Rouhier N. Navrot N. Jacquot J. P. (2005). The plant thioredoxin system. Cell Mol. Life Sci. 62, 24–35. 10.1007/s00018-004-4296-4 15619004 PMC11924577

[B16] Gucci R. Corelli G. Tustin S. Ravaglia G. (1995). The effect of defruiting at different stages of fruit development on leave photosynthsis of Golden Delicious apple. Tree Physiol. 15, 35–40. 14966009 10.1093/treephys/15.1.35

[B17] AC't Hoen P. Ariyurek Y. Thygesen H. H. Vreugdenhil E. Vossen R. H. de Menezes R. X.. (2008). Deep sequencing-based expression analysis shows major advances in robustness, resolution and inter-lab portability over five microarray platforms. Nucleic. Acids Res. 36, e141. 10.1093/nar/gkn705 18927111 PMC2588528

[B18] Iglesias D. J. Lliso I. Tadeo F. R. Talon M. (2002). Regulation of photosynthesis through source: sink imbalance in citrus is mediated by carbohydrate content in leaves. Physiol. Plant 116, 563–572. 10.1034/j.1399-3054.2002.1160416.x

[B19] Ishihara S. Takabayashi A. Ido K. Endo T. Ifuku K. Sato F. (2007). Distinct functions for the two PsbP-like proteins PPL1 and PPL2 in the chloroplast thylakoid lumen of Arabidopsis. Plant Physiol. 145, 668–679. 10.1104/pp.107.105866 17827269 PMC2048798

[B20] Kawakami K. Iwai M. Ikeuchi M. Kamiya N. Shen J. R. (2007). Location of PsbY in oxygen-evolving photosystem II revealed by mutagenesis and X-ray crystallography. FEBS Lett. 581, 4983–4987. 10.1016/j.febslet.2007.09.036 17910960

[B21] Kosoví K. Vítámvás P. Prášil I. T. Renaut J. (2011). Plant proteome changes under abiotic stress - Contribution of proteomics studies to understanding plant stress response. J. Proteomics. 74, 1301–1322. 10.1016/j.jprot.2011.02.006 21329772

[B22] Lee D. G. Ahsan N. Lee S. H. Kang K. Y. Bahk J. D. Lee I. J.. (2007). A proteomic approach in analyzing heat-responsive proteins in rice leaves. Proteomics 7, 3369–3383. 10.1002/pmic.200700266 17722143

[B23] Lee J. M. Sathish P. Donaghy D. J. Roche J. R. (2011). Impact of defoliation severity on photosynthesis, carbon metabolism and transport gene expression in perennial ryegrass. Funct. Plant Biol. 38, 808–817. 10.1071/FP11048 32480938

[B24] Li S. H. M. Génard C. Bussi J. G. Huguet R. Habib J. (2001). Fruit quality and leaf photosynthesis in response to microenvironment modification around individual fruit by covering the fruit with plastic in nectarine and peach trees. J. Hortic. Sci. Biotech. 76, 61–69. 10.1080/14620316.2001.11511328

[B25] Li S. H. Zhang X. P. Meng Z. Q. Wang X. (1994). Responses of peach trees to modified pruning. I. vegetative growth. N. Zeal. J. Crop Hortic. Sci. 22, 401–409. 10.1080/01140671.1994.9513852

[B26] Li W. D. Duan W. Fan F. G. Yan S. T. Li S. H. (2007). Photosynthesis in response to sink-source activity in relation to the end products and metabolic enzymes in peach trees. Tree Physiol. 27, 1307–1318. 10.1093/treephys/27.9.1307 17545130

[B27] Li W. D. Li S. H. Yang S. H. Yang J. M. Zheng X. B. Li X. D. (2005). Photosynthesis in response to sink–source manipulation during different phenological stages of fruit development in peach trees: regulation by stomatal aperture and leaf temperature. J. Hortic. Sci. Biotechnol. 80, 481–487. 10.1080/14620316.2005.11511964

[B28] Liu G. T. Ma L. Duan W. Wang B. C. Li J. H. Xu H. G.. (2014). Differential proteomic analysis of grapevine leaves by iTRAQ reveals responses to heat stress and subsequent recovery. BMC Plant Biol. 14:110. 10.1186/1471-2229-14-110 24774513 PMC4108046

[B29] López R. Brossa R. Gil L. Pita P. (2015). Stem girdling evidences a trade-off between cambial activity and sprouting and dramatically reduces plant transpiration due to feedback inhibition of photosynthesis and hormone signaling. Front. Plant Sci. 6:285. 10.3389/fpls.2015.00285 25972884 PMC4413673

[B30] Luo H. B. Ma L. Xi H. F. Duan W. Li S. H. Loescher W.. (2011). Photosynthetic responses to heat treatments at different temperatures and following recovery in grapevine (*Vitis amurensis* L.) leaves. PLoS ONE 6:e23033. 10.1371/journal.pone.0023033 21887227 PMC3162573

[B31] McCormick A. J. Cramer M. D. Watt D. A. (2008). Changes in photosynthetic rates and gene expression of leaves during a source-sink perturbation in sugarcane. Ann. Bot. 101, 89–102. 10.1093/aob/mcm258 17942591 PMC2701831

[B32] Morrissy A. S. Morin R. D. Delaney A. Zeng T. McDonald H. Jones S.. (2009). Next-generation tag sequencing for cancer gene expression profiling. Genome Res. 19, 1825–1835. 10.1101/gr.094482.109 19541910 PMC2765282

[B33] Palmblad M. Mills D. J. Bindschedler L. V. (2008). Heat-shock response in *Arabidopsis thaliana* explored by multiplexed quantitative proteomics using differential metabolic labeling. J. Proteome Res. 7, 780–785. 10.1021/pr0705340 18189342

[B34] Paul M. J. Foyer C. (2001). Sink regulation of photosynthesis. J. Exp. Bot. 52, 1383–1400. 10.1093/jexbot/52.360.1383 11457898

[B35] Richter K. Buchner J. (2001). Hsp90: chaperoning signal transduction. J. Cell Physiol. 188, 281–290. 10.1002/jcp.1131 11473354

[B36] Roose J. L. Wegener K. M. Pakrasi H. B. (2007). The extrinsic proteins of Photosystem II. Photosynth. Res. 92, 369–387. 10.1007/s11120-006-9117-1 17200881

[B37] Serrato A. J. Pérez-Ruiz J. M. Cejudo F. J. (2002). Cloning of thioredoxin h reductase and characterization of the thioredoxin reductase-thioredoxin h system from wheat. Biochem. J. 367, 491–497. 10.1042/bj20020103 12106017 PMC1222897

[B38] Setter T. L. Btun W. A. Brenner M. L. (1980). Stomatal closure and photosynthetic inhibition in soybean leaves induced by petiole girdling and pod removal. Plant Physiol. 6, 884–887. 10.1104/pp.65.5.884 16661301 PMC440443

[B39] Standfuss J. Kühlbrandt W. J. (2004). The three isoforms of the light-harvesting complex II: spectroscopic feature, trimer formation, and functional roles. J. Biol. Chem. 279, 36884–36891. 10.1074/jbc.M402348200 15208324

[B40] Strasser B. J. (1997). Donor side capacity of Photosystem II probed by chlorophyll a fluorescence transients. Photosynth. Res. 52, 147–155. 10.1023/A:1005896029778

[B41] Tong Z. Gao Z. Wang F. Zhou J. Zhang Z. (2009). Selection of reliable reference genes for gene expression studies in peach using real-time PCR. BMC Mol. Biol. 10:71. 10.1186/1471-2199-10-71 19619301 PMC3224724

[B42] Turner G. Yu O. Subramanian S. (2012). Genome organization and characteristics of soybean microRNAs. BMC Genomics 13:169. 10.1186/1471-2164-13-169 22559273 PMC3481472

[B43] Usadel B. Poree F. Nagel A. Lohse M. Czedik-Eysenberg A. Stitt M. (2009). A guide to using MapMan to visualize and compare Omics data in plants: a case study in the crop species, Maize. Plant Cell Environ. 32, 1211–1229. 10.1111/j.1365-3040.2009.01978.x 19389052

[B44] Walker A. J. Ho L. C. (1977). Carbon translocation in the tomato: carbon import and fruit growth. Ann. Bot. 41, 813–823.

[B45] Wu B. H. Huang H. Q. Fan P. G. Liu G. J. Li S. H. (2008). Photosynthetic responses to sink–source manipulation in five peach cultivars varying in maturity date. J. Am. Soc. Hortic. Sci. 133, 278–283.

[B46] Wu J. Zhang Y. Zhang H. Huang H. Folta K. M. Lu J. (2010). Whole genome wide expression profiles of *Vitis amurensis* grape responding to downy mildew by using Solexa sequencing technology. BMC Plant Biol. 10:234. 10.1186/1471-2229-10-234 21029438 PMC3017854

[B47] Xiang M. Chen S. Wang L. Dong Z. Huang J. Zhang Y.. (2013). Effect of vulculic acid produced by *Nimbya alternantherae* on the photosynthetic apparatus of *Alternanthera philoxeroides*. Plant Physiol. Biochem. 65, 81–88. 10.1016/j.plaphy.2013.01.013 23434925

[B48] Yamaguchi-Shinozaki K. Kasuga M. Liu Q. Nakashima K. Sakuma Y. Abe H. (2002). Biological mechanisms of drought stress response. JIRCAS Work Rep. 23, 1–8.

[B49] Yan B. F. Duan W. Liu G. T. Xu H. G. Wang L. J. Li S. H. (2013). Response of bean (*Vicia faba L*.) plants to low sink demand by measuring the gas exchange rates and chlorophyll a fluorescence kinetics. PLoS ONE 8:e80770. 10.1371/journal.pone.0080770 24324626 PMC3851463

[B50] Yan S. T. Li X. D. Li W. D. Fan P. G. Duan W. Li S. H. (2011). Photosynthesis and chlorophyll fluorescence response to low sink demand of tubers and roots in *Dahlia pinnata* source leaves. Biol. Plantarum 55, 83–89. 10.1007/s10535-011-0011-0

[B51] Young J. C. Moarefi I. Hartl F. U. (2001). Hsp90: a specialized but essential proteinfolding tool. J. Cell Biol. 154, 267–274. 10.1007/s10535-011-0011-0 11470816 PMC2150759

[B52] Zhou R. Quebedeaux B. (2003). Changes in photosynthesis and carbohydrate metabolism in mature apple leaves in response to whole plant source–sink manipulation. J. Am. Soc. Hortic. Sci. 128, 113–119.

