# Supplementary figures and images for: Genome-Wide Transcriptional Profile Analysis of Prunus persica in Response to Low Sink Demand after Fruit Removal
Source: Front Plant Sci. 2016 Jun 22;7:883. doi: 10.3389/fpls.2016.00883 (PMC4916340; doi:10.3389/fpls.2016.00883)

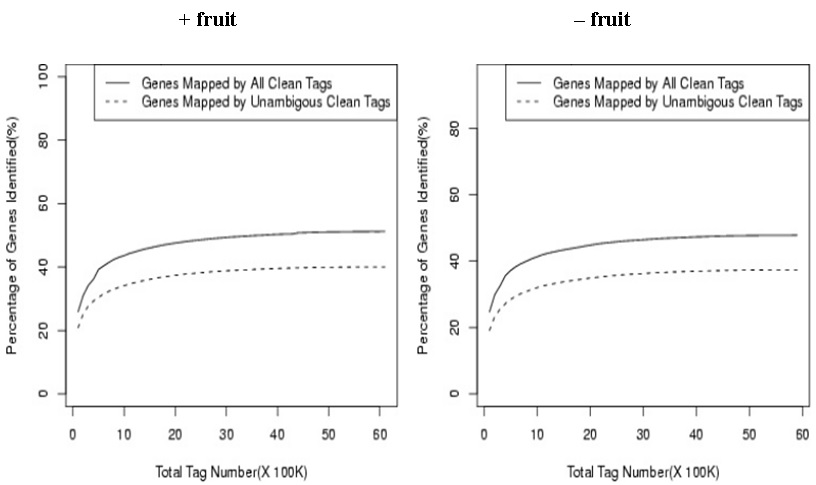

Supplement: Figure S1 — Sequencing saturation analysis of peach leaf libraries under normal sink demand (+fruit) or low sink demand (−fruit). [file Image1.JPEG]
