# Supplementary material for: Genome-Wide Transcriptional Profile Analysis of Prunus persica in Response to Low Sink Demand after Fruit Removal
Source: Front Plant Sci. 2016 Jun 22;7:883. doi: 10.3389/fpls.2016.00883 (PMC4916340; doi:10.3389/fpls.2016.00883)
Supplement: Table S1 — Summary of parameters, formulae and their description using data extracted from chlorophyll a fluorescence transient (O-J-I-P-test). [file Table1.DOCX]

**Table S1** Summary of parameters, formulae and their description using data extracted from chlorophyll a fluorescence transient (O-J-I-P-test)

| **Fluorescence parameters** | **Fluorescence parameters Description** |
| --- | --- |
| *Extracted parameters* |  |
| *F*_t_ | Fluorescence intensity at time t after onset of actinic illumination |
| *F*_50 µs_ | Minimum reliable recorded fluorescence at 50 µs with the Handy PEA |
| *F*_k_ (F_300 µs_) | Fluorescence intensity at 300 µs |
| *F*_P_ | Maximum recorded (= maximum possible) fluorescence at P-step |
| Area | Total complementary area between fluorescence induction curve and *F* = *F*_m_ |
| *Derived parameters* |  |
| *F*_o_ ≌ *F*_50 µs_ | Minimum fluorescence, when all PSII RCs are open |
| *F*_m_ = *F*_P_ | Maximum fluorescence, when all PSII RCs are closed |
| *V*_j_ = (*F*_2 ms_ - *F*_o_)/(*F*_m_ - *F*_o_) | Relative variable fluorescence at the J-step (2 ms) |
| *V*_i_ = (*F*_30 ms_ - *F*_o_)/(*F*_m_ - *F*_o_)  W_K_ = (F_k_-F_o_/(F_j_-F_o_)  Mo=4 (F_300 ms_-F_o_)/(F_m_-F_o_) | Relative variable fluorescence at the I-step (30 ms)  Represent the damage to oxygen evolving complex(OEC)  Approximated initial slope (in ms^-1^) of the fluorescence transient V = f(t) |
| *φ*_Po_ = TR_o_/ABS = 1 - (*F*_o_/*F*_m_) = *F*_v_/*F*_m_ | Maximum quantum yield of primary photochemistry at t = 0 |
| *φ*_Eo_ = ET_o_/ABS = (*F*_v_/*F*_m_) × (1 – *V*_j_) | Quantum yield for electron transport at t = 0 |
| *ψ*_Eo_ = ET_o_/TR_o_ = 1 - *V*_j_ | Probability (at time 0) that a trapped exciton moves an electron into the electron transport chain beyond Q_A_^-^ |
| *δ*_Ro_ = (1-V_i_)/(1-V_j_)  RC_QA_ =*φ*_Po_ × (ABS/CS_m_) × (*V*_j_/*M*_o_) | Efficiency with which an electron can move from the PQ through PSI to the PSI end electron acceptors  Amount of active PSII RCs (Q_A_-reducing PSII reaction centers) per CS at t = m |
