# Supplementary material for: Genome-Wide Transcriptional Profile Analysis of Prunus persica in Response to Low Sink Demand after Fruit Removal
Source: Front Plant Sci. 2016 Jun 22;7:883. doi: 10.3389/fpls.2016.00883 (PMC4916340; doi:10.3389/fpls.2016.00883)
Supplement: Table S2 — List of primers used for the Real-time PCR. [file Table2.DOCX]

**Table S2.** List of primers used for the Real-time PCR.

| **Gene ID** | **Forward primer** | **Reverse primer** | **Target size** |
| --- | --- | --- | --- |
| ppa003959m | AGACAAGTCCAAGGCAGATG | TTGATTCGGGCACTAAGAAG | 119 |
| ppa011130m | GGATGTCAAGGTGGCTGTAG | AAGCTGAAGGCGGGTATTAT | 134 |
| ppa001029m | TCGTAAAGGGGATGTGGTAA | CTGGAAACTCCATGTTGCTC | 120 |
| ppa001820m | CAGGTGTCCAGGAGAAAAGA | TGTTAACCAATGGGCAATTT | 126 |
| ppa010952m | AAACAATGTTCGTCCGGTTA | ACGCTAAGTTGGGTGCAATA | 144 |
| ppa015151m | AGAAGAAAGGGGATCATTGG | GAGAACCCCATTCTCCAACT | 122 |
| ppa007999m | AAAGAGGCCTGGAAAAGCTA | GGGAGAAAAGACCGAAGTGT | 101 |
| ppa010479m | TACTGTTGCACTCCCCCTAA | AAGGTGCTGCTCTTGTCATC | 122 |
| ppa011103m | GACCAAGATCAGGAAGCTCA | GAATGTTCTTGCCCACAATC | 124 |
| ppa009328m | GGAGTTATGCTTTGGAGCAA | TGGGGAAAACCTACTGATGA | 136 |
