# Supplementary material for: Genome-Wide Transcriptional Profile Analysis of Prunus persica in Response to Low Sink Demand after Fruit Removal
Source: Front Plant Sci. 2016 Jun 22;7:883. doi: 10.3389/fpls.2016.00883 (PMC4916340; doi:10.3389/fpls.2016.00883)
Supplement: Table S3 — Detail information of peach DEGs under low sink demand. [file Table3.DOC]

| **Table S3.** Detail information of peach DEGs under low sink demand | | | | | |  |  |
| --- | --- | --- | --- | --- | --- | --- | --- |
| **Gene ID** | **gi** | **Accession Number** | **Fold Change** | **BIN** | **NAME** | **Annotation** | **E value** |
| ppa003423m | gi|50086713| | ACX55864.1 | 9.08 | 16.1.5 | secondary metabolism.isoprenoids.terpenoids | GCN5-related N-acetyltransferase (GNAT) family protein [ *Pyrus communis* ] | 0 |
| ppa007707m | gi|118485828| | AAQ57202.1 | 8.65 | 14.2 | S-assimilation.APR | Adenosine 5' phosphosulfate reductase [ *Populus tremula x Populus alba* ] | 0 |
| ppa005494m | gi|297739417| | XP_002533520.1 | 8.65 | 29.4.1.57 | protein.postranslational modification.kinase.receptor like cytoplasmatic kinase VII | BRASSINOSTEROID INSENSITIVE 1-associated receptor kinase 1 precursor, putative [ *Ricinus communis* ] | 2E-171 |
| ppa016710m | gi|224140975| | XP_002323852.1 | 8.65 | 33.99 | development.unspecified | NAC domain protein, IPR003441 [ *Populus trichocarpa* ] | 0 |
| ppa024165m | gi|225465204| | XP_002526429.1 | 8.45 | 29.1.4 | protein.aa activation.leucine-tRNA ligase | Leucyl-trna synthetase, putative [ *Ricinus communis* ] | 0 |
| ppa016831m | gi|224069525| | XP_002868815.1 | 8.13 | 17.8.1 | hormone metabolism.salicylic acid.synthesis-degradation | S-adenosyl-L-methionine:carboxyl methyltransferase family protein [ *Arabidopsis lyrata subsp. Lyrata* ] | 2E-89 |
| ppa003107m | gi|225444009| | ADP88925.1 | 7.94 | 34.3 | transport.amino acids | Cationic amino acid transporter [ *Populus trichocarpa* ] | 0 |
| ppa010837m | gi|296085576| | XP_003631369.1 | 7.94 | 35.2 | not assigned.unknown | Unnamed protein product [ *Vitis vinifera* ] | 2.00E-146 |
| ppa018063m | gi|255584168| | XP_002532823.1 | 7.83 | 26.9 | misc.glutathione S transferases | Glutathione s-transferase, putative [ *Ricinus communis* ] | 0 |
| ppa004913m | gi|225434355| | ABY52426.1 | 7.83 | 29.4 | protein.postranslational modification | NN mitogen-activated protein kinase [ *Nicotiana tabacum* ] | 4E-124 |
| ppa001710m | gi|57281695| | BAD86587.1 | 7.71 | 11.8.1 | lipid metabolism.'exotics' (steroids, squalene etc).sphingolipids | Sphingosine kinase [ *Lotus japonicus* ] | 0 |
| ppa023262m | gi|147778583| | XP_002524941.1 | 4.50 | 26.1 | misc.cytochrome P450 | Cytochrome P450, putative [ *Ricinus communis* ] | 0 |
| ppa014796m | gi|225463687| | XP_002273824.2 | 4.00 | 30.2.11 | signalling.receptor kinases.leucine rich repeat XI | LRR receptor-like serine/threonine-protein kinase FLS2-like [ *Vitis vinifera* ] | 0 |
| ppa003639m | gi|255564230| | XP_002523112.1 | 3.90 | 10.8.1 | cell wall.pectin*esterases.PME | Pectinesterase-2 precursor, putative [ *Ricinus communis* ] | 0 |
| ppa013754m | - | unkown | 3.77 | 35.2 | not assigned.unknown | Unkown | 0 |
| ppa009328m | gi|224116850| | ABA26457.1 | 3.70 | 20.1 | stress.biotic | Acidic class III chitinase [ *Citrullus lanatus* ] | 4E-118 |
| ppa008039m | gi|224137370| | NP_180221.1 | 3.69 | 30.3 | signalling.calcium | AR781 [ *Arabidopsis thaliana* ] | 3E-51 |
| ppa010796m | gi|224072727| | ACV92007.1 | 3.52 | 27.3.32 | RNA.regulation of transcription.WRKY domain transcription factor family | WRKY transcription factor 5 [ *(Populus tomentosa x P. Bolleana) x P. Tomentosa* ] | 9E-152 |
| ppb017079m | gi|225456081| | ABW38009.1 | 3.44 | 17.8.1 | hormone metabolism.salicylic acid.synthesis-degradation | Loganic acid methyltransferase [ *Catharanthus roseus* ] | 2E-93 |
| ppa008877m | gi|82604209| | ABB84756.1 | 3.41 | 27.3.25 | RNA.regulation of transcription.MYB domain transcription factor family | MYB8 [ *Malus x domestica* ] | 5E-174 |
| ppa017577m | gi|225450954| | XP_002513811.1 | 3.36 | 28.99 | DNA.unspecified | Esterase precursor, putative [ *Ricinus communis* ] | 3E-153 |
| ppa011103m | gi|172050751| | XP_002318460.1 | 3.23 | 20.2.1 | stress.abiotic.heat | heat shock 22K family protein [ *Populus trichocarpa* ] | 5E-98 |
| ppa023839m | gi|224082796| | ADJ67439.1 | 3.21 | 17.5.2 | hormone metabolism.ethylene.signal transduction | Ethylene response factor 10 [ *Actinidia deliciosa* ] | 2.00E-98 |
| ppa012795m | gi|41059801| | XP_006486450.1 | 3.18 | 20.2.1 | stress.abiotic.heat | 18.2 kDa class I heat shock protein-like [ *Citrus sinensis* ] | 2.00E-78 |
| ppa006006m | gi|255574623| | XP_002528222.1 | 3.07 | 10.1.6 | cell wall.precursor synthesis.GAE | UDP-glucuronate 5-epimerase, putative [ *Ricinus communis* ] | 0 |
| ppa010479m | gi|190613907| | ACE80957.1 | 3.03 | 20.1 | stress.biotic | Putative allergen Pru p 2.01A [ *Prunus dulcis x Prunus persica* ] | 5E-140 |
| ppa004664m | gi|224093824| | XP_002310007.1 | 2.98 | 26.1 | misc.cytochrome P450 | Cytochrome P450 [ *Populus trichocarpa* ] | 0 |
| ppa010568m | gi|1729981| | ADM22305.1 | 2.93 | 20.1 | stress.biotic | Pathogenesis related protein 5 [ *Prunus domestica* ] | 8.00E-150 |
| ppa005715m | gi|55375996| | AAV50009.1 | 2.89 | 16.2 | secondary metabolism.phenylpropanoids | Anthranilate N-hydroxycinnamoyl/benzoyltransferase [ *Malus x domestica* ] | 0 |
| ppa010573m | gi|255567494| | XP_002524726.1 | 2.85 | 35.1 | not assigned.no ontology | Transmembrane BAX inhibitor motif-containing protein, putative [ *Ricinus communis* ] | 4E-135 |
| ppa009238m | gi|255554410| | XP_002518244.1 | 2.85 | 26.13 | misc.acid and other phosphatases | Phosphatase DCR2, putative [ *Ricinus communis* ] | 0 |
| ppa016778m | gi|224065427| | NP_189165.1 | 2.80 | 34.13 | transport.peptides and oligopeptides | Proton-dependent oligopeptide transport (POT) family protein [ *Arabidopsis thaliana* ] | 2E-173 |
| ppa013711m | gi|224105067| | ABN47005.1 | 2.74 | 33.99 | development.unspecified | Early nodulin 93 protein [ *Populus alba x Populus tremula var. Glandulosa* ] | 3E-48 |
| ppa021697m | gi|225466956| | NP_001045085.1 | 2.71 | 35.2 | not assigned.unknown | Os01g0897100 [ *Oryza sativa Japonica Group* ] | 1E-129 |
| ppa010086m | gi|89276793| | ABD66595.1 | 2.70 | 15.2 | metal handling.binding, chelation and storage | Iron-binding protein [ *Pyrus pyrifolia* ] | 3E-152 |
| ppa007758m | gi|170676254| | ACB30366.1 | 2.63 | 29.4.1.57 | protein.postranslational modification.kinase.receptor like cytoplasmatic kinase VII | Pto kinase interactor 1 [ *Capsicum annuum* ] | 0 |
| ppa007999m | gi|29134843| | BAC66141.1 | 2.59 | 26.4.1 | misc.beta 1,3 glucan hydrolases.glucan endo-1,3-beta-glucosidase | Beta-1,3-glucanase [ *Fragaria x ananassa* ] | 0 |
| ppa005427m | gi|224080189| | XP_002532899.1 | 2.56 | 26.2 | misc.UDP glucosyl and glucoronyl transferases | UDP-glucosyltransferase, putative [ *Ricinus communis* ] | 1E-151 |
| ppa001246m | gi|225447492| | XP_002884662.1 | 2.50 | 35.2 | not assigned.unknown | Hypothetical protein ARALYDRAFT_896937 [ *Arabidopsis lyrata subsp. Lyrata* ] | 0 |
| ppa015151m | gi|255630012| | P30236.1 | 2.49 | 20.2.1 | stress.abiotic.heat | 22.0 kda class IV heat shock protein [ *Glycine max* ] | 1E-84 |
| ppa009438m | gi|255640977| | BAG50066.1 | 2.46 | 27.3.26 | RNA.regulation of transcription.MYB-related transcription factor family | Transcription factor NAC [ *Lotus japonicus* ] | 5E-121 |
| ppb010767m | gi|255570463| | XP_002526190.1 | 2.43 | 35.2 | not assigned.unknown | ATP binding protein, putative [ *Ricinus communis* ] | 0 |
| ppa014063m | gi|4105810| | AAD02561.1 | 2.42 | 35.2 | not assigned.unknown | PGPS/NH21 [ *Petunia x hybrida* ] | 1E-38 |
| ppa004776m | gi|32526566| | CAD42908.1 | 2.41 | 21.6 | redox.dismutases and catalases | Catalase [ *Prunus persica* ] | 0 |
| ppa000865m | gi|224121262| | BAB86283.1 | 2.41 | 31.1 | cell.organisation | Kinesin-like protein NACK1 [ *Nicotiana tabacum* ] | 0 |
| ppa010816m | gi|255568460| | XP_002525204.1 | 2.40 | 26.9 | misc.glutathione S transferases | Glutathione-s-transferase omega, putative [ *Ricinus communis* ] | 2E-137 |
| ppa010830m | gi|225462531| | NP_001045860.1 | 2.39 | 35.2 | not assigned.unknown | Os02g0141500 [ *Oryza sativa Japonica Group* ] | 3E-20 |
| ppa001885m | gi|225426710| | XP_002275471.1 | 2.37 | 29.5.1 | protein.degradation.subtilases | Similar to subtilase [ *Vitis vinifera* ] | 0 |
| ppa003313m | gi|255565988| | ABA95619.1 | 2.35 | 33.99 | development.unspecified | Nodulin-like family protein, expressed [ *Oryza sativa Japonica Group* ] | 0 |
| ppa012715m | gi|255559739| | XP_002465312.1 | 2.30 | 35.2 | not assigned.unknown | Hypothetical protein SORBIDRAFT_01g036205 [ *Sorghum bicolor* ] | 0.00002 |
| ppa016413m | gi|224082250| | XP_002306617.1 | 2.26 | 35.2 | not assigned.unknown | Predicted protein [ *Populus trichocarpa* ] | 2E-99 |
| ppa010582m | gi|255583578| | NP_001144265.1 | 2.24 | 35.2 | not assigned.unknown | Hypothetical protein LOC100277139 [ *Zea mays* ] | 3E-08 |
| ppa007645m | gi|225448679| | XP_002520865.1 | 2.22 | 27.3.35 | RNA.regulation of transcription.bZIP transcription factor family | Transcription factor RF2b, putative [ *Ricinus communis* ] | 7E-128 |
| ppa011493m | gi|255624305| | XP_002877267.1 | 2.21 | 35.2 | not assigned.unknown | Hypothetical protein ARALYDRAFT_905411 [ *Arabidopsis lyrata subsp. Lyrata* ] | 3E-25 |
| ppa010952m | gi|269927197| | ACZ52964.1 | 2.21 | 20.1 | stress.biotic | Chitinase [ *Dimocarpus longan* ] | 2E-131 |
| ppa010445m | gi|255539370| | NP_001117322.1 | 2.19 | 27.3.99 | RNA.regulation of transcription.unclassified | Zinc-binding family protein [ *Arabidopsis thaliana* ] | 1E-99 |
| ppa010437m | gi|224108313| | NP_001061872.1 | 2.15 | 35.2 | not assigned.unknown | Os08g0433200 [ *Oryza sativa Japonica Group* ] | 3E-81 |
| ppa014165m | - | unkown | 2.14 | 35.2 | not assigned.unknown | Unkown | 0 |
| ppa009473m | gi|255567031| | XP_002285199.1 | 2.10 | 20.2 | stress.abiotic | SPX domain-containing protein 2 isoform 1 [ *Vitis vinifera* ] | 1.00E-157 |
| ppa013145m | gi|225451247| | XP_002518257.1 | 2.08 | 29.2.1.2.1.24 | protein.synthesis.ribosomal protein.eukaryotic.40S subunit.S24 | 40S ribosomal protein S24, putative [ *Ricinus communis* ] | 3E-69 |
| ppa008301m | gi|206584345| | ACI15345.1 | 2.07 | 33.99 | development.unspecified | NAC domain protein NAC4 [ *Gossypium hirsutum* ] | 0 |
| ppa020514m | gi|225442009| | XP_002533245.1 | 2.05 | 27.3.99 | RNA.regulation of transcription.unclassified | Zinc finger protein, putative [ *Ricinus communis* ] | 2E-129 |
| ppa016992m | gi|224056272| | XP_002522135.1 | 2.05 | 29.5.5 | protein.degradation.serine protease | Serine carboxypeptidase, putative [ *Ricinus communis* ] | 3E-179 |
| ppa015265m | gi|224075499| | XP_002871553.1 | 2.04 | 35.2 | not assigned.unknown | Hypothetical protein ARALYDRAFT_488135 [ *Arabidopsis lyrata subsp. Lyrata* ] | 0 |
| ppa025459m | gi|255578319| | XP_002530026.1 | 2.02 | 35.1 | not assigned.no ontology | Leucine-rich repeat-containing protein, putative [ *Ricinus communis* ] | 0 |
| ppa005689m | gi|255578375| | XP_002530054.1 | 2.01 | 29.4 | protein.postranslational modification | AFC, putative [ *Ricinus communis* ] | 0 |
| ppa011839m | gi|224116054| | XP_002317195.1 | 2.01 | 35.2 | not assigned.unknown | Predicted protein [ *Populus trichocarpa* ] | 5.00E-149 |
| ppa011885m | - | unkown | 2.01 | 35.2 | not assigned.unknown | Unkown | 0 |
| ppa010880m | gi|255538222| | ABD32636.1 | 1.96 | 35.2 | not assigned.unknown | Hypothetical protein mtrdraft_AC150207g26v2 [ *Medicago truncatula* ] | 2E-64 |
| ppa002833m | gi|255571162| | XP_002269414.2 | 1.96 | 33.99 | development.unspecified | WD repeat-containing protein 70-like [ *Vitis vinifera* ] | 0 |
| ppa002187m | gi|255545176| | NP_200076.1 | 1.94 | 20.2.1 | stress.abiotic.heat | heat shock protein 90.1 [ *Arabidopsis thaliana* ] | 0 |
| ppa008972m | gi|255588003| | XP_002534470.1 | 1.94 | 27.2 | RNA.transcription | Transcription initiation factor iib, putative [ *Ricinus communis* ] | 0 |
| ppa008293m | gi|300153385| | XP_002884208.1 | 1.93 | 9.1.1 | mitochondrial electron transport / ATP synthesis.NADH-DH.complex I | ATPHB6 [ *Arabidopsis lyrata subsp. Lyrata* ] | 2.00E-147 |
| ppa012615m | gi|224101857| | XP_002535200.1 | 1.93 | 20.2.99 | stress.abiotic.unspecified | Major latex protein, putative [ *Ricinus communis* ] | 1E-50 |
| ppa012766m | gi|148807152| | ABR13286.1 | 1.92 | 35.2 | not assigned.unknown | Putative glycine-rich protein [ *Prunus dulcis* ] | 1E-52 |
| ppa005810m | gi|255558712| | XP_002520380.1 | 1.90 | 26.2 | misc.UDP glucosyl and glucoronyl transferases | Glycosyltransferase QUASIMODO1, putative [ *Ricinus communis* ] | 0 |
| ppa000037m | gi|255544986| | XP_002513554.1 | 1.89 | 12.2.1 | N-metabolism.ammonia metabolism.glutamate synthase | Glutamate synthase, putative [ *Ricinus communis* ] | 0 |
| ppa002140m | gi|224108351| | XP_002526597.1 | 1.89 | 34.99 | transport.misc | Auxin:hydrogen symporter, putative [ *Ricinus communis* ] | 0 |
| ppa013205m | gi|225460067| | XP_002274416.1 | 1.89 | 35.2 | not assigned.unknown | Hypothetical protein [ *Vitis vinifera* ] | 2E-79 |
| ppa003391m | gi|225433375| | XP_002285608.1 | 1.87 | 29.6 | protein.folding | Similar to groel-like chaperone, atpase [ *Vitis vinifera* ] | 0 |
| ppa008267m | gi|255564908| | XP_002523447.1 | 1.87 | 29.5.3 | protein.degradation.cysteine protease | Cysteine protease, putative [ *Ricinus communis* ] | 0 |
| ppa004332m | gi|147767047| | XP_002511297.1 | 1.87 | 26.1 | misc.cytochrome P450 | Cytochrome P450, putative [ *Ricinus communis* ] | 0 |
| ppa011685m | gi|255640396| | CAA58731.1 | 1.86 | 30.1 | signalling.in sugar and nutrient physiology | PAR-1b [ *Nicotiana tabacum* ] | 3E-65 |
| ppa013218m | gi|255538684| | ACF23034.1 | 1.86 | 35.2 | not assigned.unknown | ST225 [ *Thellungiella halophila* ] | 1E-13 |
| ppa001725m | gi|224100647| | XP_002311961.1 | 1.83 | 35.2 | not assigned.unknown | Predicted protein [ *Populus trichocarpa* ] | 0 |
| ppa006609m | gi|225440538| | XP_002275792.1 | 1.82 | 35.2 | not assigned.unknown | Hypothetical protein [ *Vitis vinifera* ] | 0 |
| ppa009274m | gi|296082424| | XP_002277765.2 | 1.82 | 20.2.1 | stress.abiotic.heat | Heat shock factor protein HSF24-like [ *Vitis vinifera* ] | 0 |
| ppa002894m | gi|225439287| | XP_002525724.1 | 1.82 | 10.6.1 | cell wall.degradation.cellulases and beta -1,4-glucanases | Hydrolase, hydrolyzing O-glycosyl compounds, putative [ *Ricinus communis* ] | 0 |
| ppa007443m | gi|225443377| | XP_002513312.1 | 1.82 | 34.9 | transport.metabolite transporters at the mitochondrial membrane | Mitochondrial deoxynucleotide carrier, putative [ *Ricinus communis* ] | 4E-161 |
| ppa004000m | gi|255575031| | XP_002528421.1 | 1.81 | 26.7 | misc.oxidases - copper, flavone etc | Multicopper oxidase, putative [ *Ricinus communis* ] | 0 |
| ppa005851m | gi|224062651| | XP_002520369.1 | 1.80 | 16.1.3.1 | secondary metabolism.isoprenoids.tocopherol biosynthesis.hydroxyphenylpyruvate dioxygenase | 4-hydroxyphenylpyruvate dioxygenase, putative [ *Ricinus communis* ] | 0 |
| ppa002111m | gi|164605000| | BAF98296.1 | 1.79 | 16.1.1.6 | secondary metabolism.isoprenoids.non-mevalonate pathway.HDS | 4-hydroxy-3-methylbut-2-en-1-yl diphosphate synthase [ *Hevea brasiliensis* ] | 0 |
| ppa000422m | gi|255548928| | XP_002515520.1 | 1.79 | 27.2 | RNA.transcription | RNA-dependent RNA polymerase, putative [ *Ricinus communis* ] | 0 |
| ppa014506m | gi|3334263| | AAF78526.1 | 1.78 | 35.2 | not assigned.unknown | Metallothionein-like protein [ *Pyrus pyrifolia* ] | 6E-19 |
| ppa002540m | gi|224075096| | ABC02880.1 | 1.78 | 11.1.9 | lipid metabolism.FA synthesis and FA elongation.long chain fatty acid CoA ligase | ACS1 [ *Ricinus communis* ] | 0 |
| ppa005616m | gi|224142001| | XP_002515387.1 | 1.77 | 27.3.99 | RNA.regulation of transcription.unclassified | Aspartic proteinase nepenthesin-2 precursor, putative [ *Ricinus communis* ] | 8E-169 |
| ppa004369m | gi|224086589| | XP_002521054.1 | 1.77 | 19.3 | tetrapyrrole synthesis.GSA | Aminobutyrate aminotransferase, putative [ *Ricinus communis* ] | 0 |
| ppa013342m | gi|4928460| | AAD33596.1 | 1.77 | 21.1 | redox.thioredoxin | Thioredoxin h [ *Hevea brasiliensis* ] | 3E-67 |
| ppa003449m | gi|224115418| | XP_002864311.1 | 1.77 | 27.3.99 | RNA.regulation of transcription.unclassified | Mitochondrial transcription termination factor-related / mterf-related [ *Arabidopsis thaliana* ] | 8E-109 |
| ppa004067m | gi|296085209| | XP_002517369.1 | 1.76 | 35.2 | not assigned.unknown | ATP binding protein, putative [ *Ricinus communis* ] | 2E-95 |
| ppa008801m | gi|15148912| | AAK84883.1 | 1.76 | 33.99 | development.unspecified | NAC domain protein NAC1 [ *Phaseolus vulgaris* ] | 6E-180 |
| ppa006477m | gi|225448765| | XP_002525069.1 | 1.76 | 35.2 | not assigned.unknown | Erythroblast macrophage protein emp, putative [ *Ricinus communis* ] | 0 |
| ppa026506m | gi|224066971| | XP_002513938.1 | 1.75 | 26.2 | misc.UDP glucosyl and glucoronyl transferases | UDP-glucuronosyltransferase, putative [ *Ricinus communis* ] | 0 |
| ppa002673m | gi|224089259| | XP_002308665.1 | 1.74 | 29.5.11.4.3.2 | protein.degradation.ubiquitin.E3.SCF.FBOX | Ein3-binding f-box protein 3 [ *Populus trichocarpa* ] | 0 |
| ppa004845m | gi|224091028| | XP_002309151.1 | 1.74 | 34.5 | transport.ammonium | Ammonium transporter [ *Populus trichocarpa* ] | 0 |
| ppa009612m | gi|255566888| | XP_002524427.1 | 1.74 | 29.2.1.1.1.1.530 | protein.synthesis.ribosomal protein.prokaryotic.chloroplast.30S subunit.S30A | Plastid-specific 30S ribosomal protein 1, chloroplast precursor, putative [ *Ricinus communis* ] | 3E-180 |
| ppa024953m | gi|297743775| | XP_002533443.1 | 1.73 | 19.40 | tetrapyrrole synthesis.regulation | Tetrapyrrole-binding protein, chloroplast precursor, putative [ *Ricinus communis* ] | 4E-77 |
| ppa014023m | gi|255578827| | AAF06043.1 | 1.73 | 35.2 | not assigned.unknown | EST gb|T44882 comes from this gene [ *Arabidopsis thaliana* ] | 8E-25 |
| ppa000503m | gi|297830294| | NP_188306.2 | 1.73 | 33.99 | development.unspecified | TPR2 (TOPLESS-RELATED 2) [ *Arabidopsis thaliana* ] | 0 |
| ppa001412m | gi|157313306| | ABV32546.1 | 1.72 | 26.3.2 | misc.gluco-, galacto- and mannosidases.beta-galactosidase | Beta-galactosidase protein 1 [ *Prunus persica* ] | 0 |
| ppa002591m | gi|224115828| | XP_002332067.1 | 1.72 | 20.2.1 | stress.abiotic.heat | heat shock protein 70 cognate [ *Populus trichocarpa* ] | 0 |
| ppa010125m | gi|224136566| | XP_002510349.1 | 1.71 | 35.1 | not assigned.no ontology | Peroxisomal biogenesis factor, putative [ *Ricinus communis* ] | 3E-109 |
| ppa003730m | gi|225459633| | NP_192096.1 | 1.70 | 35.1 | not assigned.no ontology | Tolb protein-related [ *Arabidopsis thaliana* ] | 1E-129 |
| ppa008004m | gi|255559983| | XP_002521010.1 | 1.70 | 27.3.6 | RNA.regulation of transcription.bHLH,Basic Helix-Loop-Helix family | DNA binding protein, putative [ *Ricinus communis* ] | 0 |
| ppa005390m | gi|296089698| | EOY11503.1 | 1.70 | 11.3 | lipid metabolism.Phospholipid synthesis | Phospholipid/glycerol acyltransferase family protein [ *Theobroma cacao* ] | 0 |
| ppa011645m | gi|255579787| | XP_004146166.1 | 1.70 | 30.5 | signalling.G-proteins | Rho gtpase-activating protein 8-like [ *Cucumis sativus* ] | 8.00E-100 |
| ppa008418m | gi|255642900| | CAE85073.1 | 1.70 | 26.13 | misc.acid and other phosphatases | Putative acid phosphatase [ *Lupinus luteus* ] | 3E-159 |
| ppa004016m | gi|225434610| | XP_002525632.1 | 1.69 | 35.1 | not assigned.no ontology | Hydrolase, acting on ester bonds, putative [ *Ricinus communis* ] | 0 |
| ppa000543m | gi|224117970| | XP_002533849.1 | 1.68 | 27.3.67 | RNA.regulation of transcription.putative transcription regulator | Nuclear transcription factor, X-box binding, putative [*Ricinus communis* ] | 0 |
| ppa005997m | gi|302142458| | NP_565102.1 | 1.67 | 35.2 | not assigned.unknown | RRA2 (REDUCED RESIDUAL ARABINOSE 2) [*Arabidopsis thaliana* ] | 0 |
| ppa012598m | gi|217074082| | XP_002532879.1 | 1.67 | 16.4.3.1 | secondary metabolism.N misc.cyanogenic glycosides.cyanase | Cyanate hydratase, putative [ *Ricinus communis* ] | 4E-71 |
| ppa012425m | gi|225432660| | XP_002512708.1 | 1.66 | 1.1.1.2 | PS.lightreaction.photosystem II.PSII polypeptide subunits | Photosystem II 11 kda protein precursor, putative [ *Ricinus communis* ] | 6E-94 |
| ppa014332m | - | unkown | 1.66 | 35.2 | not assigned.unknown | Unkown | 0 |
| ppa001001m | gi|296086606| | XP_002520662.1 | 1.66 | 29.5.9 | protein.degradation.AAA type | Peroxisome assembly factor-2, putative [ *Ricinus communis* ] | 0 |
| ppa013228m | gi|255561174| | ABA54810.1 | 1.66 | 35.2 | not assigned.unknown | Late embryogenesis abundant protein [ *Picea abies* ] | 0.000007 |
| ppa003143m | gi|255555301| | XP_002518687.1 | 1.65 | 13.1.5.1.1 | amino acid metabolism.synthesis.serine-glycine-cysteine group.serine.phosphoglycerate dehydrogenase | D-3-phosphoglycerate dehydrogenase, putative [ *Ricinus communis* ] | 0 |
| ppa012014m | gi|289466349| | ADC94860.1 | 1.65 | 17.5.2 | hormone metabolism.ethylene.signal transduction | ERF transcription factor 4 [ *Vitis pseudoreticulata* ] | 2E-101 |
| ppa007586m | gi|255561403| | ACG35019.1 | 1.65 | 35.1 | not assigned.no ontology | Dehydrodolichyl diphosphate synthase 6 [ *Zea mays* ] | 2E-55 |
| ppa017359m | gi|297742152| | CBI33939.3 | 1.65 | 35.2 | not assigned.unknown | Unnamed protein product [ *Vitis vinifera* ] | 8.00E-65 |
| ppa008687m | gi|225438613| | XP_002878588.1 | 1.65 | 29.5.11.4.2 | protein.degradation.ubiquitin.E3.RING | Protein binding protein [ *Arabidopsis lyrata subsp. Lyrata* ] | 2E-154 |
| ppa012310m | gi|225437024| | NP_563677.1 | 1.64 | 27.3.70 | RNA.regulation of transcription.Silencing Group | GCN5-related N-acetyltransferase (GNAT) family protein [ *Arabidopsis thaliana* ] | 3E-95 |
| ppa009904m | gi|255641141| | XP_002527425.1 | 1.64 | 26.13 | misc.acid and other phosphatases | Phosphoethanolamine/phosphocholine phosphatase, putative [ *Ricinus communis* ] | 3E-118 |
| ppa008776m | gi|225459431| | NP_564475.1 | 1.63 | 29.6 | protein.folding | Co-chaperone grpe family protein [ *Arabidopsis thaliana* ] | 1E-64 |
| ppa004872m | gi|224134557| | ACN57982.1 | 1.63 | 35.2 | not assigned.unknown | At5g51670-like protein [ *Capsella grandiflora* ] | 9E-47 |
| ppa013361m | gi|255550964| | EOY29794.1 | 1.63 | 27.3.40 | RNA.regulation of transcription.Aux/IAA family | Phytochrome-associated protein 1, putative isoform 1 [ *Theobroma cacao* ] | 2.00E-89 |
| ppa009505m | gi|296089030| | XP_002517841.1 | 1.62 | 9.1.1 | mitochondrial electron transport / ATP synthesis.NADH-DH.complex I | Prohibitin, putative [ *Ricinus communis* ] | 7E-149 |
| ppa000901m | gi|225470737| | XP_002892935.1 | 1.61 | 29.5.11.5 | protein.degradation.ubiquitin.ubiquitin protease | Ubiquitin-specific protease 15 [ *Arabidopsis lyrata subsp. Lyrata* ] | 0 |
| ppa004531m | gi|225444434| | XP_002519047.1 | 1.61 | 23.2 | nucleotide metabolism.degradation | Allantoinase, putative [ *Ricinus communis* ] | 0 |
| ppa009429m | gi|302143319| | NP_199241.2 | 1.61 | 29.5.11.4.2 | protein.degradation.ubiquitin.E3.RING | RING1A (RING 1A); protein binding / zinc ion binding [ *Arabidopsis thaliana* ] | 9E-113 |
| ppa006860m | gi|255546939| | XP_002514527.1 | 1.61 | 35.2 | not assigned.unknown | Conserved hypothetical protein [ *Ricinus communis* ] | 0 |
| ppa002208m | gi|225458499| | EOY14589.1 | 1.60 | 29.4 | protein.postranslational modification | Mitogen-activated protein kinase kinase kinase 5, putative isoform 3 [ *Theobroma cacao* ] | 0 |
| ppa009369m | gi|224110250| | ADP30960.1 | 1.60 | 20.2.3 | stress.abiotic.drought/salt | Dehydration-induced 19-like protein [ *Gossypium hirsutum* ] | 5E-63 |
| ppa003580m | gi|209420826| | AAB09228.1 | 1.59 | 16.1 | secondary metabolism.simple phenols | Laccase [ *Acer pseudoplatanus* ] | 0 |
| ppa004453m | gi|225458659| | XP_002510192.1 | 1.59 | 29.4.1.57 | protein.postranslational modification.kinase.receptor like cytoplasmatic kinase VII | Serine/threonine-protein kinase PBS1, putative [ *Ricinus communis* ] | 0 |
| ppa011364m | gi|255560187| | XP_002521111.1 | 1.59 | 35.2 | not assigned.unknown | Conserved hypothetical protein [ *Ricinus communis* ] | 2E-152 |
| ppa009687m | gi|87240747| | ABD32605.1 | 1.58 | 35.2 | not assigned.unknown | Conserved hypothetical protein [ *Medicago truncatula* ] | 1E-161 |
| ppa006444m | gi|255551481| | XP_002516786.1 | 1.58 | 35.1 | not assigned.no ontology | Membrane protein PB1A10.07c, putative [ *Ricinus communis* ] | 0 |
| ppa012356m | gi|224121706| | ADM67612.1 | 1.58 | 18.7 | Co-factor and vitamine metabolism.iron-sulphur clusters | Iron-sulfur cluster scaffold protein [ *Hevea brasiliensis* ] | 5.00E-96 |
| ppa013107m | gi|225430081| | ADB93062.1 | 1.57 | 1.1.1.2 | PS.lightreaction.photosystem II.PSII polypeptide subunits | Chloroplast photosystem II 10 kda polypeptide [ *Jatropha curcas* ] | 8E-61 |
| ppa014090m | gi|257219554| | ACV50430.1 | 1.57 | 35.2 | not assigned.unknown | Copper chaperone [ *Jatropha curcas* ] | 1E-44 |
| ppa013839m | gi|224143346| | XP_002878810.1 | 1.57 | 21.2 | redox.ascorbate and glutathione | Membrane-associated progesterone binding protein 2 [ *Arabidopsis lyrata subsp. Lyrata* ] | 3.00E-53 |
| ppa013639m | gi|296082348| | ACG34249.1 | 1.57 | 35.2 | not assigned.unknown | E2 protein isoform 5 [ *Zea mays* ] | 1E-43 |
| ppa007848m | gi|225452099| | NP_001151953.1 | 1.57 | 35.2 | not assigned.unknown | Esterase/lipase/thioesterase [ *Zea mays* ] | 6E-130 |
| ppa000525m | gi|255556649| | XP_002519358.1 | 1.56 | 20.1.7 | stress.biotic.PR-proteins | Leucine-rich repeat-containing protein, putative [ *Ricinus communis* ] | 0 |
| ppa003240m | gi|255564699| | NP_566076.1 | 1.56 | 29.5.11 | protein.degradation.ubiquitin | Phosphatidylinositol 3- and 4-kinase family protein / ubiquitin family protein [ *Arabidopsis thaliana* ] | 0 |
| ppa008401m | gi|224060149| | NP_193037.1 | 1.56 | 26.7 | misc.oxidases - copper, flavone etc | Oxidoreductase, zinc-binding dehydrogenase family protein [ *Arabidopsis thaliana* ] | 4E-147 |
| ppa006081m | gi|297738784| | NP_194617.2 | 1.55 | 29.4 | protein.postranslational modification | Ckl3 (Casein Kinase I-like 3); ATP binding / kinase/ protein kinase/ protein serine/threonine kinase [ *Arabidopsis thaliana* ] | 0 |
| ppa002102m | gi|225442114| | AAQ23899.1 | 1.55 | 30.3 | signalling.calcium | RSH2 [ *Nicotiana tabacum* ] | 0 |
| ppa010298m | gi|224118004| | NP_567660.1 | 1.55 | 11.8.1 | lipid metabolism.'exotics' (steroids, squalene etc).sphingolipids | ATCES1; catalytic/ hydrolase, acting on carbon-nitrogen (but not peptide) bonds, in linear amides [ *Arabidopsis thaliana* ] | 1E-128 |
| ppa013898m | gi|224099491| | ABN46980.1 | 1.55 | 29.6 | protein.folding | Small molecular heat shock protein 10 [ *Nelumbo nucifera* ] | 1E-35 |
| ppa013270m | gi|82568700|d | BAE48663.1 | 1.55 | 35.2 | not assigned.unknown | Pm52 [ *Prunus mume* ] | 1E-68 |
| ppa012768m | gi|255627393| | XP_002524079.1 | 1.54 | 35.1 | not assigned.no ontology | Phosphatidylglycerol/phosphatidylinositol transfer protein precursor, putative [ *Ricinus communis* ] | 2E-61 |
| ppa006361m | gi|225442675| | NP_564606.1 | 1.54 | 30.5 | signalling.G-proteins | Ran-binding protein 1 domain-containing protein / ranbp1 domain-containing protein [ *Arabidopsis thaliana* ] | 1E-107 |
| ppa001397m | gi|255540727| | XP_002511428.1 | 1.54 | 3.2.3 | minor CHO metabolism.trehalose.potential TPS/TPP | Trehalose-6-phosphate synthase, putative [ *Ricinus communis* ] | 0 |
| ppa002715m | gi|14572556| | AAK64657.1 | 1.54 | 16.1.2.3 | secondary metabolism.isoprenoids.mevalonate pathway.HMG-CoA reductase | 3-hydroxy-3-methylglutaryl coenzyme A reductase [ *Malus x domestica* ] | 0 |
| ppa004544m | gi|68164961|g | AAY87450.1 | 1.53 | 26.1 | misc.cytochrome P450 | Cinnamic acid hydroxylase [ *Malus x domestica* ] | 0 |
| ppa010400m | gi|296086977| | NP_193961.2 | 1.53 | 11.9.3.2 | lipid metabolism.lipid degradation.lysophospholipases.carboxylesterase | SOBER1 (suppressor of avrbst-elicited resistance 1); carboxylesterase [ *Arabidopsis thaliana* ] | 0 |
| ppa009165m | gi|224129602| | ACN87274.1 | 1.52 | 26.22 | misc.short chain dehydrogenase/reductase (SDR) | Short chain dehydrogenase/reductase [ *Chelidonium majus* ] | 7E-91 |
| ppa007287m | gi|118489341| | NP_849323.1 | 1.52 | 27.3.99 | RNA.regulation of transcription.unclassified | Zinc finger (C2H2 type) family protein [ *Arabidopsis thaliana* ] | 7E-128 |
| ppa004802m | gi|15485720| | CAC67501.1 | 1.51 | 15 | metal handling | Selenium binding protein [ *Medicago sativa* ] | 0 |
| ppa008747m | gi|255550754| | XP_002866150.1 | 1.51 | 34.9 | transport.metabolite transporters at the mitochondrial membrane | Mitochondrial substrate carrier family protein [ *Arabidopsis lyrata subsp. Lyrata* ] | 1E-145 |
| ppa013416m | gi|224077114| | ACH87172.1 | 1.51 | 27.3.99 | RNA.regulation of transcription.unclassified | Zinc finger protein LSD2-like protein [ *Camellia sinensis* ] | 1E-28 |
| ppa008873m | gi|255578282| | ABN08265.1 | 1.50 | 10.8.1 | cell wall.pectin*esterases.PME | Pectinesterase [ *Medicago truncatula* ] | 2E-160 |
| ppa008153m | gi|225446056| | NP_001077915.1 | 1.50 | 31.4 | cell.vesicle transport | Syntaxin family protein [ *Arabidopsis thaliana* ] | 3E-50 |
| ppa023947m | - | unkown | 1.50 | 35.2 | not assigned.unknown | Unkown | 0 |
| ppa013127m | gi|242050704| | ACG40694.1 | 1.50 | 9.1.2 | mitochondrial electron transport / ATP synthesis.NADH-DH.localisation not clear | NADH-ubiquinone oxidoreductase 18 kda subunit [ *Zea mays* ] | 3E-83 |
| ppa022767m | gi|224095017| | XP_002525303.1 | 1.49 | 35.2 | not assigned.unknown | Phytosulfokines precursor, putative [ *Ricinus communis* ] | 7E-29 |
| ppa002270m | gi|225464023| | XP_002525541.1 | 1.49 | 27.2 | RNA.transcription | DNA-directed RNA polymerase III subunit, putative [ *Ricinus communis* ] | 0 |
| ppa016063m | gi|225458637| | XP_002510203.1 | 1.49 | 11.9.2.1 | lipid metabolism.lipid degradation.lipases.triacylglycerol lipase | Triacylglycerol lipase, putative [ *Ricinus communis* ] | 2E-172 |
| ppa006772m | gi|255539106| | XP_002510618.1 | 1.48 | 29.5.11.4.3.2 | protein.degradation.ubiquitin.E3.SCF.FBOX | Ubiquitin-protein ligase, putative [ *Ricinus communis* ] | 0 |
| ppa002155m | gi|225448930| | XP_002271948.2 | 1.48 | 35.1 | not assigned.no ontology | Hypothetical protein [ *Vitis vinifera* ] | 0 |
| ppa005695m | gi|225459860| | XP_003078220.1 | 1.47 | 35.2 | not assigned.unknown | RNA polymerase II transcription elongation factor DSIF/SUPT5H/SPT5 (ISS) [ *Ostreococcus tauri* ] | 7E-39 |
| ppa004075m | gi|297742567| | XP_002509715.1 | 1.47 | 29.5 | protein.degradation | Ara4-interacting protein, putative [ *Ricinus communis* ] | 0 |
| ppa011870m | gi|224101647| | XP_002514546.1 | 1.47 | 26.24 | misc.GCN5-related N-acetyltransferase | N-acetyltransferase, putative [ *Ricinus communis* ] | 5E-72 |
| ppa014417m | - | unkown | 1.46 | 35.2 | not assigned.unknown | Unkown | 0 |
| ppa011072m | gi|255637772| | XP_003517423.1 | 1.45 | 21.1 | redox.thioredoxin | Thioredoxin-like 2, chloroplastic-like [ *Glycine max* ] | 5.00E-154 |
| ppa008652m | gi|225468218| | XP_002331442.1 | 1.44 | 27.3.59 | RNA.regulation of transcription.Methyl binding domain proteins | Methyl binding domain protein [ *Populus trichocarpa* ] | 4E-113 |
| ppa006034m | gi|225452805| | XP_002323861.1 | 1.44 | 34.3 | transport.amino acids | Amino acid transporter [ *Populus trichocarpa* ] | 8E-168 |
| ppa010210m | gi|296084042| | NP_001150080.1 | 1.44 | 35.2 | not assigned.unknown | Growth inhibition and differentiation-related protein 88 [ *Zea mays* ] | 1E-47 |
| ppa008006m | gi|255548948| | XP_002515530.1 | 1.44 | 34.8 | transport.metabolite transporters at the envelope membrane | Triose phosphate/phosphate translocator, non-green plastid, chloroplast precursor, putative [ *Ricinus communis* ] | 0 |
| ppa002956m | gi|255558332| | XP_002520193.1 | 1.44 | 29.5.11.3 | protein.degradation.ubiquitin.E2 | Ubiquitin-protein ligase, putative [ *Ricinus communis* ] | 0 |
| ppa013299m | gi|53748519| | CAH59452.1 | 1.44 | 21.1 | redox.thioredoxin | Thioredoxin 3 [ *Plantago major* ] | 4E-78 |
| ppa008879m | gi|255545406| | AGG38119.1 | 1.43 | 31.1 | cell.organisation | Maternal effect embryo arrest 66 protein [ *Dimocarpus longan* ] | 1.00E-82 |
| ppb022557m | gi|225430977| | XP_002514852.1 | 1.43 | 27.3.32 | RNA.regulation of transcription.WRKY domain transcription factor family | WRKY transcription factor, putative [ *Ricinus communis* ] | 3E-168 |
| ppa011866m | gi|224093164| | AAX92905.1 | 1.43 | 35.2 | not assigned.unknown | Expressed protein [ *Oryza sativa Japonica Group* ] | 7E-44 |
| ppa026365m | gi|225426334| | XP_002265840.1 | 1.42 | 35.2 | not assigned.unknown | Hypothetical protein [ *Vitis vinifera* ] | 0 |
| ppa008791m | gi|7108577| | AAF36483.1 | 1.42 | 17.5.1 | hormone metabolism.ethylene.synthesis-degradation | 1-aminocyclopropane-1-carboxylate oxidase [ *Prunus persica* ] | 0 |
| ppa009629m | gi|38679321| | EOY06105.1 | 1.42 | 31.1 | cell.organisation | Plastid-lipid associated protein PAP / fibrillin family protein [ *Theobroma cacao* ] | 2.00E-126 |
| ppa002288m | gi|225424719| | XP_002521030.1 | 1.42 | 29.1.3 | protein.aa activation.threonine-tRNA ligase | Threonyl-trna synthetase, putative [ *Ricinus communis* ] | 0 |
| ppa009406m | gi|296085199| | NP_567140.1 | 1.42 | 35.2 | not assigned.unknown | Unnamed protein product [ *Vitis vinifera* ] | 0 |
| ppa007450m | gi|224111448| | EOY01084.1 | 1.41 | 17.5.1 | hormone metabolism.ethylene.synthesis-degradation | 2-oxoglutarate and Fe(II)-dependent oxygenase superfamily protein, putative [ *Theobroma cacao* ] | 0 |
| ppa005982m | gi|296088312| | XP_002319550.1 | 1.41 | 29.3.3 | protein.targeting.chloroplast | Amidase family protein [ *Populus trichocarpa* ] | 0 |
| ppa004554m | gi|225442426| | XP_002515947.1 | 1.41 | 29.3.2 | protein.targeting.mitochondria | Mitochondrial processing peptidase alpha subunit, putative [ *Ricinus communis* ] | 0 |
| ppa001893m | gi|224083077| | XP_002510554.1 | 1.40 | 27.1.2 | RNA.processing.RNA helicase | Dead box ATP-dependent RNA helicase, putative [ *Ricinus communis* ] | 0 |
| ppa000009m | gi|225459044| | XP_002527362.1 | 1.40 | 29.5.11.4.1 | protein.degradation.ubiquitin.E3.HECT | E3 ubiquitin protein ligase upl2, putative [ *Ricinus communis* ] | 0 |
| ppa002396m | gi|157313302| | ABV32544.1 | 1.40 | 35.1 | not assigned.no ontology | Alpha-L-arabinofuranosidase protein [ *Prunus persica* ] | 0 |
| ppa002575m | gi|255543639| | XP_002870652.1 | 1.40 | 27.3.67 | RNA.regulation of transcription.putative transcription regulator | DNA binding protein [ *Arabidopsis lyrata subsp. Lyrata* ] | 2E-132 |
| ppa002189m | gi|255571544| | XP_002526718.1 | 1.40 | 27.3.12 | RNA.regulation of transcription.C3H zinc finger family | Nucleic acid binding protein, putative [ *Ricinus communis* ] | 0 |
| ppa004349m | gi|255578112| | XP_002529926.1 | 1.39 | 29.5.4 | protein.degradation.aspartate protease | Aspartic proteinase precursor, putative [ *Ricinus communis* ] | 0 |
| ppa009562m | gi|224085938| | AAF79893.1 | 1.39 | 35.2 | not assigned.unknown | Contains similarity to pigpen protein from Mus musculus gb|AF224264 and contains protein of unknown function DUF78 PF|01918 domain. Ests gb|N38077, gb|BE037702, gb|AV442191, gb|AV441368, gb|Z17998, gb|AV527266, gb|AV520794, gb|AI997847, gb|AV543000 come from this gene [ *Arabidopsis thaliana* ] | 4E-68 |
| ppa008474m | gi|126656814| | ABO26221.1 | 1.39 | 30.2.11 | signalling.receptor kinases.leucine rich repeat XI | Polygalacturonase inhibiting protein [ *Prunus persica* ] | 0 |
| ppa009784m | gi|225442256| | CAN71449.1 | 1.39 | 35.2 | not assigned.unknown | Hypothetical protein [ *Vitis vinifera* ] | 0 |
| ppa002282m | gi|302144247| | AAF14635.1 | 1.39 | 11.9.4.2 | lipid metabolism.lipid degradation.beta-oxidation.acyl CoA DH | Acyl-coa oxidase [ *Petroselinum crispum* ] | 0 |
| ppa004048m | gi|297746327| | XP_002513510.1 | 1.38 | 27.1 | RNA.processing | Trna (5-methylaminomethyl-2-thiouridylate)-methyltransferase, putative [ *Ricinus communis* ] | 0 |
| ppa010832m | gi|255566360| | XP_002524166.1 | 1.38 | 11.1.5 | lipid metabolism.FA synthesis and FA elongation.beta hydroxyacyl ACP dehydratase | Hydroxyacyl-ACP Dehydrase [ *Ricinus communis* ] | 1E-135 |
| ppa009651m | gi|224091915| | XP_002530162.1 | 1.38 | 27.1.19 | RNA.processing.ribonucleases | Ccr4-associated factor, putative [ *Ricinus communis* ] | 8E-107 |
| ppa009212m | gi|255642074| | XP_636793.1 | 1.38 | 10.6.1 | cell wall.degradation.cellulases and beta -1,4-glucanases | DUF1649 family protein [ *Dictyostelium discoideum AX4* ] | 4E-20 |
| ppa012813m | gi|297839753| | XP_002887758.1 | 1.38 | 29.5.11.3 | protein.degradation.ubiquitin.E2 | Ubiquitin-conjugating enzyme 1 [ *Arabidopsis lyrata subsp. Lyrata* ] | 5E-84 |
| ppa005219m | gi|8131905|gb | AAF73132.1 | 1.38 | 13.2.6.2 | amino acid metabolism.degradation.aromatic aa.tyrosine | Homogentisate 1,2-dioxygenase [ *Solanum lycopersicum* ] | 0 |
| ppa013206m | gi|255629416| | XP_002324843.1 | 1.38 | 28.1.3 | DNA.synthesis/chromatin structure.histone | Histone 2 [ *Populus trichocarpa* ] | 2E-63 |
| ppb004684m | gi|148807183| | ABR13301.1 | 1.38 | 35.2 | not assigned.unknown | Putative proline-rich cell wall protein [ *Prunus dulcis* ] | 5E-61 |
| ppa005222m | gi|255557699| | XP_002519879.1 | 1.37 | 34.3 | transport.amino acids | Amino acid transporter, putative [ *Ricinus communis* ] | 0 |
| ppa014141m | gi|224076777| | AAO12870.1 | 1.36 | 35.2 | not assigned.unknown | Wound induced protein-like [ *Vitis vinifera* ] | 6E-18 |
| ppa008494m | gi|225434692| | ABB29956.1 | 1.36 | 2.2.1.1 | major CHO metabolism.degradation.sucrose.fructokinase | Fructokinase-like [ *Solanum tuberosum* ] | 9E-161 |
| ppa000076m | gi|108711620| | NP_193111.2 | 1.36 | 35.2 | not assigned.unknown | ATP binding [ *Arabidopsis thaliana* ] | 0 |
| ppa003290m | gi|185179560| | ACC77697.1 | 1.36 | 31.1 | cell.organisation | NPR1-like protein [ *Malus x domestica* ] | 0 |
| ppa010917m | gi|255558526| | NP_001168337.1 | 1.35 | 35.2 | not assigned.unknown | Hypothetical protein LOC100382105 [ *Zea mays* ] | 9E-53 |
| ppa004148m | gi|225424793| | AAB61100.1 | 1.35 | 35.2 | not assigned.unknown | Ests gb|R30459,gb|N38441 come from this gene [ *Arabidopsis thaliana* ] | 0 |
| ppa007047m | gi|255644546| | XP_002516727.1 | 1.35 | 28.2 | DNA.repair | Uv excision repair protein rad23, putative [ *Ricinus communis* ] | 1E-158 |
| ppa013346m | gi|297738940| | EFN55876.1 | 1.35 | 29.2.1.2.2.57 | protein.synthesis.ribosomal protein.eukaryotic.60S subunit.L7A | Ribosomal protein l7ae, putative [ *Ricinus communis* ] | 5.00E-75 |
| ppa000452m | gi|225447927| | NP_173706.1 | 1.34 | 35.1 | not assigned.no ontology | T-complex protein 11 [ *Arabidopsis thaliana* ] | 0 |
| ppa004425m | gi|224118264| | XP_002513552.1 | 1.34 | 27.1.1 | RNA.processing.splicing | Splicing factor 3a, putative [ *Ricinus communis* ] | 0 |
| ppa000897m | gi|255544824| | XP_002513473.1 | 1.34 | 34.12 | transport.metal | Copper-transporting atpase p-type, putative [ *Ricinus communis* ] | 0 |
| ppa025549m | gi|255559320| | NP_594278.1 | 1.34 | 35.2 | not assigned.unknown | Rrna processing protein Fyv7 (predicted) [ *Schizosaccharomyces pombe 972h-* ] | 0.18 |
| ppa004150m | gi|225441959| | XP_002264087.1 | 1.34 | 35.2 | not assigned.unknown | Uncharacterized protein LOC100254979 isoform 1 [ *Vitis vinifera* ] | 0 |
| ppa013706m | gi|225456262| | NP_001105682.1 | 1.34 | 35.2 | not assigned.unknown | Brain protein 44-like [ *Zea mays* ] | 2E-48 |
| ppa010235m | gi|296088813| | XP_002263023.2 | 1.34 | 35.2 | not assigned.unknown | Unnamed protein product [ *Vitis vinifera* ] | 0 |
| ppa002696m | gi|224082628| | XP_002284757.1 | 1.34 | 30.5 | signalling.G-proteins | Mitochondrial Rho gtpase 1 [ *Vitis vinifera* ] | 0 |
| ppa013233m | gi|224066847| | NP_001151051.1 | 1.33 | 35.2 | not assigned.unknown | Glutaredoxin 2 [ *Zea mays* ] | 2E-33 |
| ppa007865m | gi|224123248| | XP_002330269.1 | 1.33 | 17.5.1 | hormone metabolism.ethylene.synthesis-degradation | 2-oxoglutarate-dependent dioxygenase [ *Populus trichocarpa* ] | 0 |
| ppa002098m | gi|224109458| | ABP63534.1 | 1.33 | 13.1.2.2 | amino acid metabolism.synthesis.glutamate family.proline | Pyrroline-5-carboxylate synthetase [ *Populus euphratica* ] | 0 |
| ppa003779m | gi|255540633| | XP_002511381.1 | 1.33 | 29.2.2.3.1 | protein.synthesis.ribosome biogenesis.Pre-rRNA processing and modifications.snoRNPs | U3 small nucleolar ribonucleoprotein protein mpp10, putative [ *Ricinus communis* ] | 0 |
| ppa012496m | gi|242199344| | ACS87993.1 | 1.33 | 26.2 | misc.UDP glucosyl and glucoronyl transferases | UDP-glucosyltransferase family 1 protein [ *Citrus sinensis* ] | 0 |
| ppa012957m | gi|224066545| | XP_002521598.1 | 1.33 | 29.2.1.2.1.12 | protein.synthesis.ribosomal protein.eukaryotic.40S subunit.S12 | 40S ribosomal protein S12, putative [ *Ricinus communis* ] | 2E-59 |
| ppa002485m | gi|225442468| | NP_178091.2 | 1.32 | 27.3 | RNA.regulation of transcription | Protein early flowering 7 [ *Arabidopsis thaliana* ] | 0 |
| ppa002526m | gi|224125542| | EOY28187.1 | 1.32 | 27.3.99 | RNA.regulation of transcription.unclassified | Zinc finger C-x8-C-x5-C-x3-H type family protein, putative isoform 1 [ *Theobroma cacao* ] | 5.00E-150 |
| ppa002024m | gi|224060139| | NP_974810.1 | 1.32 | 29.4 | protein.postranslational modification | GPI transamidase component family protein / Gaa1-like family protein [ *Arabidopsis thaliana* ] | 0 |
| ppa002475m | gi|255549238| | XP_002515673.1 | 1.32 | 34.13 | transport.peptides and oligopeptides | Oligopeptide transporter, putative [ *Ricinus communis* ] | 0 |
| ppa011387m | gi|225462930| | XP_002522481.1 | 1.32 | 35.1 | not assigned.no ontology | Transmembrane emp24 domain-containing protein 10 precursor, putative [ *Ricinus communis* ] | 2E-88 |
| ppa007472m | gi|255538698| | XP_002510414.1 | 1.31 | 29.5.11.20 | protein.degradation.ubiquitin.proteasom | Proteasome subunit alpha type, putative [ *Ricinus communis* ] | 9E-166 |
| ppa004221m | gi|225449963| | NP_196884.1 | 1.31 | 35.1 | not assigned.no ontology | Hydrolase, alpha/beta fold family protein [ *Arabidopsis thaliana* ] | 2E-172 |
| ppa007454m | gi|225427714| | ABX79341.1 | 1.31 | 29.5.3 | protein.degradation.cysteine protease | Cysteine protease [ *Vitis vinifera* ] | 0 |
| ppa003662m | gi|224115922| | AAC67586.1 | 1.31 | 4.1.4 | glycolysis.cytosolic branch.phosphofructokinase (PFK) | Pyrophosphate-dependent phosphofructokinase beta subunit [ *Citrus x paradisi* ] | 0 |
| ppa009950m | gi|22331369| | BAA95721.1 | 1.31 | 35.2 | not assigned.unknown | Unnamed protein product [ *Arabidopsis thaliana* ] | 0 |
| ppa009661m | gi|222139394| | ACM45716.1 | 1.31 | 20.1 | stress.biotic | Class IV chitinase [ *Pyrus pyrifolia* ] | 5E-159 |
| ppa007957m | gi|255585327| | XP_002533361.1 | 1.30 | 29.5 | protein.degradation | O-sialoglycoprotein endopeptidase, putative [ *Ricinus communis* ] | 0 |
| ppa003083m | gi|225433795| | XP_002880034.1 | 1.30 | 35.2 | not assigned.unknown | Hypothetical protein ARALYDRAFT_483433 [ *Arabidopsis lyrata subsp. Lyrata* ] | 0 |
| ppa004682m | gi|255556860| | XP_002519463.1 | 1.30 | 29.4 | protein.postranslational modification | Protein phosphatase 2a, regulatory subunit, putative [ *Ricinus communis* ] | 0 |
| ppa009637m | gi|15081588| | AAK82460.1 | 1.30 | 20.1 | stress.biotic | Type 2 ribosome-inactivating protein cinnamomin III precursor [ *Cinnamomum camphora* ] | 0 |
| ppa005102m | gi|255571968| | ABB89022.1 | 1.30 | 35.2 | not assigned.unknown | CXE carboxylesterase [ *Actinidia deliciosa* ] | 0 |
| ppa011013m | gi|255563594| | XP_002522799.1 | 1.30 | 35.2 | not assigned.unknown | Pre-mrna-splicing factor cwc15, putative [ *Ricinus communis* ] | 4E-128 |
| ppa006127m | gi|225456323| | XP_002511184.1 | 1.30 | 35.1 | not assigned.no ontology | Ubiquitin-activating enzyme E1 domain-containing protein, putative [ *Ricinus communis* ] | 0 |
| ppa011028m | gi|225439410| | ADN33788.1 | 1.29 | 15 | metal handling | Selenoprotein t precursor [ *Cucumis melo subsp. Melo* ] | 6E-90 |
| ppa001840m | gi|225442611| | ADL36724.1 | 1.29 | 27.3.22 | RNA.regulation of transcription.HB,Homeobox transcription factor family | HD domain class transcription factor [ *Malus x domestica* ] | 0 |
| ppa004312m | gi|225469228| | AAT46067.1 | 1.29 | 27.3.32 | RNA.regulation of transcription.WRKY domain transcription factor family | DNA binding protein WRKY2 [ *Vitis vinifera* ] | 0 |
| ppa021622m | gi|82697943| | ABB89006.1 | 1.29 | 35.1 | not assigned.no ontology | CXE carboxylesterase [ *Malus pumila* ] | 0 |
| ppa011185m | gi|296087850| | XP_002513027.1 | 1.28 | 27.3.35 | RNA.regulation of transcription.bZIP transcription factor family | Transcription factor HBP-1b(c1), putative [ *Ricinus communis* ] | 0 |
| ppa003217m | gi|224100903| | XP_002526791.1 | 1.28 | 31.1 | cell.organisation | Ankyrin repeat-containing protein, putative [ *Ricinus communis* ] | 0 |
| ppa002252m | gi|297735064| | XP_002273723.2 | 1.28 | 35.2 | not assigned.unknown | Unnamed protein product [ *Vitis vinifera* ] | 0 |
| ppa002499m | gi|147773525| | XP_002512955.1 | 1.28 | 27.3.22 | RNA.regulation of transcription.HB,Homeobox transcription factor family | Bel1 homeotic protein, putative [ *Ricinus communis* ] | 0 |
| ppa002222m | gi|225456004| | CAA52149.1 | 1.28 | 20.2.1 | stress.abiotic.heat | Heat shock protein 70 [ *Cucumis sativus* ] | 0 |
| ppa008559m | gi|224055529| | AAC49785.1 | 1.28 | 8.3 | TCA / org transformation.carbonic anhydrases | Carbonic anhydrase [ *Populus tremula x Populus tremuloides* ] | 0 |
| ppa010195m | gi|224095992| | XP_002298178.1 | 1.27 | 1.1.1.1 | PS.lightreaction.photosystem II.LHC-II | Light-harvesting complex II protein Lhcb6 [ *Populus trichocarpa* ] | 2E-142 |
| ppa020356m | gi|160690596| | ABX46128.1 | 1.27 | 10.7 | cell wall.modification | Blight-associated protein P12 [ *Citrus webberi* ] | 6E-60 |
| ppa007140m | gi|225453720| | XP_002509740.1 | 1.27 | 29.5.11.4.2 | protein.degradation.ubiquitin.E3.RING | Cleavage and polyadenylation specificity factor, putative [ *Ricinus communis* ] | 1E-127 |
| ppa010335m | gi|224065411| | XP_002865655.1 | 1.27 | 31.3 | cell.cycle | Cyclin family protein [ *Arabidopsis lyrata subsp. Lyrata* ] | 6E-112 |
| ppa010365m | gi|38678108|d | BAD04010.1 | 1.27 | 34.19.2 | transport.Major Intrinsic Proteins.TIP | Tonoplast intrinsic protein [ *Prunus persica* ] | 2E-137 |
| ppa000840m | gi|255573730| | XP_002527786.1 | 1.26 | 35.2 | not assigned.unknown | Hypothetical protein RCOM_0629030 [ *Ricinus communis* ] | 0 |
| ppa007437m | gi|224088832| | XP_002880742.1 | 1.26 | 29.7 | protein.glycosylation | Galactosyltransferase family protein [ *Arabidopsis lyrata subsp. Lyrata* ] | 3E-155 |
| ppa010685m | gi|255629542| | XP_003635162.1 | 1.26 | 27.4 | RNA.RNA binding | Uncharacterized RNA-binding protein C25G10.01-like [ *Vitis vinifera* ] | 1.00E-65 |
| ppa002787m | gi|255579273| | XP_002530482.1 | 1.26 | 8.1.7 | TCA / org transformation.TCA.succinate dehydrogenase | Succinate dehydrogenase, putative [ *Ricinus communis* ] | 0 |
| ppa012958m | gi|255563951| | XP_002864359.1 | 1.26 | 20.2.99 | stress.abiotic.unspecified | Pollen ole e 1 allergen and extensin family protein [ *Arabidopsis lyrata subsp. Lyrata* ] | 4E-58 |
| ppa016854m | gi|224058657| | CAJ00007.1 | 1.26 | 35.2 | not assigned.unknown | Serine rich protein [ *Medicago truncatula* ] | 2E-29 |
| ppa002811m | gi|262070776| | ACY08857.1 | 1.26 | 26.2 | misc.UDP glucosyl and glucoronyl transferases | Xyloglucan galactosyltransferase [ *Eucalyptus grandis* ] | 0 |
| ppa006784m | gi|255563442| | XP_002522723.1 | 1.26 | 13.1.5.3.2 | amino acid metabolism.synthesis.serine-glycine-cysteine group.cysteine.SAT | Serine acetyltransferase 3, mitochondrial precursor, putative [ *Ricinus communis* ] | 0 |
| ppa011322m | gi|296086667| | XP_002529743.1 | 1.26 | 30.1 | signalling.phosphorelay | Histidine phosphotransfer protein [ *Populus trichocarpa* ] | 4E-177 |
| ppa009977m | gi|255577735| | XP_002319035.1 | 1.26 | 31.4 | cell.vesicle transport | Syntaxin, putative [ *Ricinus communis* ] | 3E-60 |
| ppa007738m | gi|162424441| | ABX89941.1 | 1.26 | 17.5.1 | hormone metabolism.ethylene.synthesis-degradation | Leucoanthocyanidin dioxygenase [ *Prunus persica* ] | 0 |
| ppa000511m | gi|255541756| | XP_004306709.1 | 1.26 | 20.2.1 | stress.abiotic.heat | BAG family molecular chaperone regulator 6-like [ *Fragaria vesca subsp. Vesca* ] | 7.00E-116 |
| ppa006281m | gi|225451613| | XP_002275732.1 | 1.25 | 35.2 | not assigned.unknown | Hypothetical protein [ *Vitis vinifera* ] | 0 |
| ppa010468m | gi|224137940| | AAR28754.1 | 1.25 | 20.1 | stress.biotic | Bax inhibitor [ *Solanum lycopersicum* ] | 1E-113 |
| ppa012549m | gi|225425310| | XP_002273292.1 | 1.25 | 35.2 | not assigned.unknown | Hypothetical protein [ *Vitis vinifera* ] | 2E-90 |
| ppa006014m | gi|225459507| | XP_002513782.1 | 1.25 | 30.4.1 | signalling.phosphinositides.phosphatidylinositol-4-phosphate 5-kinase | Aminotransferase ybdl, putative [ *Ricinus communis* ] | 0 |
| ppa008754m | gi|255545734| | XP_002301056.1 | 1.25 | 35.1 | not assigned.no ontology | Predicted protein [ *Populus trichocarpa* ] | 2.00E-176 |
| ppa012597m | gi|224089118| | XP_002514360.1 | 1.25 | 26.24 | misc.GCN5-related N-acetyltransferase | Pre-mrna-splicing factor cwc24, putative [ *Ricinus communis* ] | 7E-81 |
| ppa010620m | gi|224091361| | NP_194662.2 | 1.25 | 9.1.2 | mitochondrial electron transport / ATP synthesis.NADH-DH.localisation not clear | DER1 (derlin-1) [ *Arabidopsis thaliana* ] | 1E-98 |
| ppa009625m | gi|151347473| | ABS01349.1 | 1.24 | 35.1 | not assigned.no ontology | Hypersensitive-induced response protein [ *Carica papaya* ] | 2E-166 |
| ppa006045m | gi|297738784| | XP_002514257.1 | 1.24 | 29.4 | protein.postranslational modification | Casein kinase, putative [ *Ricinus communis* ] | 0 |
| ppa006945m | gi|31414896| | AAP46143.1 | 1.24 | 16.8.3.1 | secondary metabolism.flavonoids.dihydroflavonols.dihydroflavonol 4-reductase | Cinnamoyl coa reductase [ *Fragaria x ananassa* ] | 0 |
| ppa012265m | gi|225451301| | XP_002278320.1 | 1.24 | 35.2 | not assigned.unknown | PITH domain-containing protein At3g04780 [ *Vitis vinifera* ] | 4E-126 |
| ppa007568m | gi|255565200| | XP_002523592.1 | 1.23 | 27.4 | RNA.RNA binding | RNA-binding protein, putative [ *Ricinus communis* ] | 0 |
| ppa002248m | gi|5902708| | ADP69105.1 | 1.23 | 17.1.1.1.1 | hormone metabolism.abscisic acid.synthesis-degradation.synthesis.zeaxanthin epoxidase | Zeaxanthin epoxidase [ *Cucumis sativus* ] | 0 |
| ppa006030m | gi|225449446| | ADN34070.1 | 1.23 | 27.3.99 | RNA.regulation of transcription.unclassified | Aspartic proteinase nepenthesin-1 precursor [ *Cucumis melo subsp. Melo* ] | 0 |
| ppa000169m | gi|225439753| | XP_002525185.1 | 1.23 | 29.5.11.4.1 | protein.degradation.ubiquitin.E3.HECT | Ubiquitin protein ligase e3a, putative [ *Ricinus communis* ] | 0 |
| ppa012887m | gi|255629617| | XP_002514169.1 | 1.23 | 29.2.1.2.2.28 | protein.synthesis.ribosomal protein.eukaryotic.60S subunit.L28 | 60S ribosomal protein L28, putative [ *Ricinus communis* ] | 8E-66 |
| ppa001381m | gi|255543715| | XP_002512920.1 | 1.22 | 29.2.2.3.3 | protein.synthesis.ribosome biogenesis.Pre-rRNA processing and modifications.methylotransferases | Ribosomal RNA methyltransferase, putative [ *Ricinus communis* ] | 0 |
| ppa010921m | gi|225452132| | CAN62725.1 | 1.21 | 35.2 | not assigned.unknown | Hypothetical protein VITISV_031003 [ *Vitis vinifera* ] | 1E-108 |
| ppa005366m | gi|224057042| | XP_002299116.1 | 1.21 | 34.3 | transport.amino acids | Neutral amino acid transport protein [ *Populus trichocarpa* ] | 0 |
| ppa002929m | gi|225443339| | NP_175569.1 | 1.21 | 27.4 | RNA.RNA binding | KH domain-containing protein [ *Arabidopsis thaliana* ] | 1E-144 |
| ppa012213m | gi|225448647| | XP_002279538.1 | 1.21 | 35.2 | not assigned.unknown | Uncharacterized protein LOC100241651 [ *Vitis vinifera* ] | 2.00E-126 |
| ppa000384m | gi|225465273| | XP_002527826.1 | 1.21 | 35.1 | not assigned.no ontology | Tip120, putative [ *Ricinus communis* ] | 0 |
| ppa009107m | gi|224138952| | XP_002520066.1 | 1.21 | 16.8.3.1 | secondary metabolism.flavonoids.dihydroflavonols.dihydroflavonol 4-reductase | Cinnamoyl-coa reductase, putative [ *Ricinus communis* ] | 2E-123 |
| ppa004919m | gi|225465405| | NP_851036.1 | 1.21 | 34.16 | transport.ABC transporters and multidrug resistance systems | ZIFL1 (zinc induced facilitator-like 1); tetracycline:hydrogen antiporter [ *Arabidopsis thaliana* ] | 0 |
| ppa011992m | gi|297743870| | ACG34656.1 | 1.21 | 29.6 | protein.folding | Co-chaperone grpe family protein [ *Arabidopsis thaliana* ] | 5E-49 |
| ppa010237m | gi|225435731| | NP_187966.1 | 1.21 | 27.1.1 | RNA.processing.splicing | SCL30a; RNA binding / nucleic acid binding / nucleotide binding [ *Arabidopsis thaliana* ] | 4E-67 |
| ppa006288m | gi|225462533| | ABX10445.1 | 1.20 | 11.3.6 | lipid metabolism.Phospholipid synthesis.choline-phosphate cytidylyltransferase | Ethanolamine-phosphate cytidylyltransferase 1 [ *Gossypium hirsutum* ] | 0 |
| ppa004902m | gi|224096247| | AAD48837.1 | 1.20 | 13.1.1.3.11 | amino acid metabolism.synthesis.central amino acid metabolism.alanine.alanine-glyoxylate aminotransferase | Alanine:glyoxylate aminotransferase 2 homolog [ *Arabidopsis thaliana* ] | 2E-173 |
| ppa002411m | gi|255563230| | NP_568989.1 | 1.20 | 30.11 | signalling.light | NPH3 (non-phototropic hypocotyl 3); protein binding / signal transducer [ *Arabidopsis thaliana* ] | 0 |
| ppa008114m | gi|297741046| | NP_173694.1 | 1.20 | 18.6 | Co-factor and vitamine metabolism.biotin | Methyltransferase [ *Arabidopsis thaliana* ] | 5E-40 |
| ppa021267m | gi|225454077| | XP_002512585.1 | 1.20 | 29.5.11.4.2 | protein.degradation.ubiquitin.E3.RING | Ring finger, putative [ *Ricinus communis* ] | 7E-146 |
| ppa000466m | gi|225428063| | XP_002886086.1 | 1.20 | 29.5.11 | protein.degradation.ubiquitin | Ubiquitin-conjugating enzyme 22 [ *Arabidopsis lyrata subsp. Lyrata* ] | 0 |
| ppa007884m | gi|224117588| | XP_002884932.1 | 1.20 | 29.5.11.4.2 | protein.degradation.ubiquitin.E3.RING | Protein binding protein [ *Arabidopsis lyrata subsp. Lyrata* ] | 3E-85 |
| ppa005900m | gi|224116398| | NP_001033134.2 | 1.20 | 35.1.41 | not assigned.no ontology.hydroxyproline rich proteins | Vacuolar protein sorting-associated protein VTA1 homolog [ *Bos taurus* ] | 2E-24 |
| ppa003789m | gi|296083879| | NP_568258.2 | 1.20 | 23.2.1.4 | nucleotide metabolism.degradation.pyrimidine.dihydropyrimidinase | Dihydropyrimidinase / dhpase / dihydropyrimidine amidohydrolase / hydantoinase (PYD2) [ *Arabidopsis thaliana* ] | 0 |
| ppa009233m | gi|255559496| | XP_002520768.1 | 1.19 | 35.1 | not assigned.no ontology | Phenazine biosynthesis protein, putative [ *Ricinus communis* ] | 0 |
| ppa009609m | gi|255540995| | XP_002511562.1 | 1.19 | 35.2 | not assigned.unknown | Conserved hypothetical protein [ *Ricinus communis* ] | 0 |
| ppa023795m | gi|224065707| | XP_002510634.1 | 1.19 | 26.11 | misc.alcohol dehydrogenases | Alcohol dehydrogenase, putative [ *Ricinus communis* ] | 0 |
| ppa000017m | gi|225445342| | XP_002281542.1 | 1.19 | 20.2.1 | stress.abiotic.heat | dnaJ homolog subfamily C member 13-like [ *Vitis vinifera* ] | 0 |
| ppa008854m | gi|255560992| | XP_002521508.1 | 1.19 | 26.1 | misc.misc2 | Epoxide hydrolase, putative [ *Ricinus communis* ] | 0 |
| ppa005058m | gi|224062848| | XP_002524352.1 | 1.19 | 16.5.99.1 | secondary metabolism.sulfur-containing.misc.alliinase | Nucleotide binding protein, putative [ *Ricinus communis* ] | 0 |
| ppa002947m | gi|225440606| | NP_974263.1 | 1.18 | 28.1 | DNA.synthesis/chromatin structure | Mrna capping enzyme family protein [ *Arabidopsis thaliana* ] | 0 |
| ppa023813m | gi|255538116| | ADG56545.1 | 1.18 | 33.99 | development.unspecified | Progesterone 5-beta-reductase [ *Erysimum rhaeticum* ] | 0 |
| ppa005741m | gi|255567051| | XP_002524508.1 | 1.18 | 29.4 | protein.postranslational modification | CBL-interacting serine/threonine-protein kinase, putative [ *Ricinus communis* ] | 0 |
| ppa013636m | gi|224069344| | XP_002879575.1 | 1.18 | 20.2.1 | stress.abiotic.heat | DNAJ heat shock N-terminal domain-containing protein [ *Arabidopsis lyrata subsp. Lyrata* ] | 8.00E-61 |
| ppa006144m | gi|6177796| | XP_004299510.1 | 1.18 | 23.2.1.3 | nucleotide metabolism.degradation.pyrimidine.dihydrouracil dehydrogenase | NAD-dependent dihydropyrimidine dehydrogenase subunit prea-like [ *Fragaria vesca subsp. Vesca* ] | 0 |
| ppa006928m | gi|296086430| | XP_002276462.1 | 1.18 | 35.2 | not assigned.unknown | Hypothetical protein [ *Vitis vinifera* ] | 0 |
| ppa005351m | gi|224119206| | ABD33344.1 | 1.18 | 20.2.99 | stress.abiotic.unspecified | Pollen ole e 1 allergen and extensin [ *Medicago truncatula* ] | 8E-50 |
| ppa000963m | gi|297734947| | ZP_01461415.1 | 1.17 | 35.2 | not assigned.unknown | BNR repeat domain protein [ *Stigmatella aurantiaca DW4/3-1* ] | 0.093 |
| ppa006865m | gi|224093972| | NP_568192.2 | 1.17 | 18.3 | Co-factor and vitamine metabolism.riboflavin | Riboflavin biosynthesis protein-related [ *Arabidopsis thaliana* ] | 6E-118 |
| ppa014129m | gi|255633602| | ACU17160.1 | 1.17 | 35.2 | not assigned.unknown | Unknown [ *Glycine max* ] | 5E-39 |
| ppa009621m | gi|255568599| | XP_002525273.1 | 1.17 | 14.15 | S-assimilation.AKN | Adenyl sulfate kinase, putative [ *Ricinus communis* ] | 1E-163 |
| ppa010402m | gi|225433053| | XP_002529294.1 | 1.17 | 9.3 | mitochondrial electron transport / ATP synthesis.electron transfer flavoprotein | Electron transfer flavoprotein beta-subunit, putative [ *Ricinus communis* ] | 3E-126 |
| ppa008299m | gi|225426220| | NP_194136.1 | 1.16 | 26.22 | misc.short chain dehydrogenase/reductase (SDR) | Short-chain dehydrogenase/reductase (SDR) family protein [ *Arabidopsis thaliana* ] | 5E-150 |
| ppa004110m | gi|24637539|g | AAN63805.1 | 1.16 | 29.6 | protein.folding | Heat shock protein 60 [ *Prunus dulcis* ] | 0 |
| ppa005275m | gi|255543799| | XP_002512962.1 | 1.16 | 26.13 | misc.acid and other phosphatases | Purple acid phosphatase precursor, putative [ *Ricinus communis* ] | 0 |
| ppa008889m | gi|255645841| | XP_002509816.1 | 1.16 | 26.22 | misc.short chain dehydrogenase/reductase (SDR) | Short-chain dehydrogenase, putative [ *Ricinus communis* ] | 8E-143 |
| ppa005578m | gi|224143576| | AAF34804.1 | 1.16 | 29.4 | protein.postranslational modification | CDK-activating kinase [ *Euphorbia esula* ] | 0 |
| ppa006453m | gi|225446219| | XP_002530758.1 | 1.15 | 11.10.1 | lipid metabolism.glycolipid synthesis.MGDG synthase | 1,2-diacylglycerol 3-beta-galactosyltransferase, putative [ *Ricinus communis* ] | 0 |
| ppa006060m | gi|225448996| | XP_002530935.1 | 1.15 | 29.4.1.57 | protein.postranslational modification.kinase.receptor like cytoplasmatic kinase VII | Protein kinase APK1B, chloroplast precursor, putative [ *Ricinus communis* ] | 0 |
| ppa003520m | gi|225425063| | AAK15493.1 | 1.15 | 17.3.1.2.8 | hormone metabolism.brassinosteroid.synthesis-degradation.sterols.DWF1 | Brassinosteroid biosynthetic protein LKB [ *Pisum sativum* ] | 0 |
| ppa011293m | gi|255632436| | NP_198080.1 | 1.15 | 27.3.99 | RNA.regulation of transcription.unclassified | Zinc finger (DNL type) family protein [ *Arabidopsis thaliana* ] | 8E-52 |
| ppa009374m | gi|225451038| | XP_002891911.1 | 1.15 | 35.1 | not assigned.no ontology | Thylakoid lumen 18.3 kda protein [ *Arabidopsis lyrata subsp. Lyrata* ] | 1E-108 |
| ppa008876m | gi|225465643| | XP_002521533.1 | 1.15 | 29.5.11.4.2 | protein.degradation.ubiquitin.E3.RING | Ring finger protein, putative [ *Ricinus communis* ] | 5E-96 |
| ppa005353m | gi|225432812| | ABF47291.2 | 1.15 | 14.1 | S-assimilation.ATPS | ATP sulfurylase [ *Camellia sinensis* ] | 0 |
| ppa010531m | gi|225431120| | ADN33944.1 | 1.15 | 20.2.2 | stress.abiotic.cold | Cold-shock DNA-binding family protein [ *Cucumis melo subsp. Melo* ] | 2E-42 |
| ppa002106m | gi|225438549| | XP_002310031.1 | 1.15 | 34.16 | transport.ABC transporters and multidrug resistance systems | ABC transporter family protein [ *Populus trichocarpa* ] | 0 |
| ppa004848m | gi|225457648| | XP_002300622.1 | 1.15 | 27.3.44 | RNA.regulation of transcription.Chromatin Remodeling Factors | Chromatin remodeling complex subunit [ *Populus trichocarpa* ] | 1E-152 |
| ppa009503m | gi|255555295| | NP_565372.1 | 1.14 | 29.3.3 | protein.targeting.chloroplast | TIC21 (TRANSLOCON AT INNER MEMBRANE OF CHLOROPLASTS 21); copper uptake transmembrane transporter/ iron ion transmembrane transporter/ protein homodimerization [ *Arabidopsis thaliana* ] | 4E-96 |
| ppa012614m | gi|148841111| | ABR14728.1 | 1.14 | 29.5.11.3 | protein.degradation.ubiquitin.E2 | Ubiquitin conjugating enzyme [ *Gossypium hirsutum* ] | 6E-92 |
| ppa005431m | gi|225436309| | AAY89374.1 | 1.14 | 2.2.2.1.2 | major CHO metabolism.degradation.starch.starch cleavage.beta amylase | Beta-amylase 1 [ *Nicotiana langsdorffii x Nicotiana sanderae* ] | 0 |
| ppa005083m | gi|147817015| | NP_001236066.1 | 1.14 | 27.3.26 | RNA.regulation of transcription.MYB-related transcription factor family | MYB transcription factor MYB177   [ *Glycine max* ] | 2.00E-113 |
| ppa013390m | gi|8843745| | NP_568996.1 | 1.14 | 35.2 | not assigned.unknown | LIP1 (Light Insensitive Period1); gtpase [ *Arabidopsis thaliana* ] | 1E-108 |
| ppa013338m | gi|225440831| | NP_565836.1 | 1.14 | 29.5.11.1 | protein.degradation.ubiquitin.ubiquitin | Ubiquitin extension protein 2 (UBQ2) / 60S ribosomal protein L40 (RPL40A) [ *Arabidopsis thaliana* ] | 2E-68 |
| ppa013005m | gi|255557182| | XP_002519622.1 | 1.14 | 35.2 | not assigned.unknown | Conserved hypothetical protein [ *Ricinus communis* ] | 4.00E-104 |
| ppa013904m | gi|125620176| | ABN46980.1 | 1.14 | 29.6 | protein.folding | Small molecular heat shock protein 10 [ *Nelumbo nucifera* ] | 9E-48 |
| ppa002911m | gi|119507457| | BAF42036.1 | 1.14 | 10.6.1 | cell wall.degradation.cellulases and beta -1,4-glucanases | Cellulase1 [ *Pyrus communis* ] | 0 |
| ppa001592m | gi|225433588| | AAC99620.1 | 1.13 | 29.1.10 | protein.aa activation.methionine-tRNA ligase | Methionyl-trna synthetase [ *Oryza sativa* ] | 0 |
| ppa002669m | gi|227344531| | ACP27856.1 | 1.13 | 30.11 | signalling.light | Blue light photoreceptor [ *Fragaria vesca* ] | 0 |
| ppa009342m | gi|225444181| | XP_002533767.1 | 1.13 | 26.22 | misc.short chain dehydrogenase/reductase (SDR) | Short-chain dehydrogenase, putative [ *Ricinus communis* ] | 2E-137 |
| ppa013336m | gi|255566991| | XP_002524478.1 | 1.13 | 29.2.1.2.2.57 | protein.synthesis.ribosomal protein.eukaryotic.60S subunit.L7A | Ribosomal protein l7ae, putative [ *Ricinus communis* ] | 8E-68 |
| ppa012665m | gi|225462336| | XP_002519896.1 | 1.13 | 27.3.99 | RNA.regulation of transcription.unclassified | Surfeit locus protein, putative [ *Ricinus communis* ] | 6E-62 |
| ppa000479m | gi|225445672| | AAZ81522.1 | 1.13 | 27.3.4 | RNA.regulation of transcription.ARF, Auxin Response Factor family | Auxin response factor 2 [ *Gossypium arboreum* ] | 0 |
| ppa010896m | gi|225452847| | XP_002531853.1 | 1.13 | 29.2.1.2.2.34 | protein.synthesis.ribosomal protein.eukaryotic.60S subunit.L34 | 60S ribosomal protein L34, putative [ *Ricinus communis* ] | 4E-85 |
| ppa011652m | gi|255582607| | ZP_05789556.1 | 1.13 | 35.2 | not assigned.unknown | 4-amino-4-deoxy-L-arabinose transferase [ *Synechococcus sp. WH 8109* ] | 9.7 |
| ppa000877m | gi|225457929| | NP_190389.1 | 1.13 | 35.2 | not assigned.unknown | Bromo-adjacent homology (BAH) domain-containing protein [ *Arabidopsis thaliana* ] | 9E-15 |
| ppa002104m | gi|255548335| | XP_002320956.1 | 1.13 | 34.18 | transport.unspecified anions | Anion exchanger family protein [ *Populus trichocarpa* ] | 0 |
| ppa009160m | gi|82697941| | ABB89005.1 | 1.13 | 35.1 | not assigned.no ontology | CXE carboxylesterase [ *Malus pumila* ] | 3E-177 |
| ppa003807m | gi|255547996| | XP_002515055.1 | 1.13 | 26.3.5 | misc.gluco-, galacto- and mannosidases.glycosyl hydrolase family 5 | Hydrolase, hydrolyzing O-glycosyl compounds, putative [ *Ricinus communis* ] | 0 |
| ppa013814m | gi|255631404| | XP_004165917.1 | 1.12 | 35.2 | not assigned.unknown | Late embryogenesis abundant protein Lea5-A-like isoform 3 [ *Cucumis sativus* ] | 5.00E-29 |
| ppa009653m | gi|222139394| | ACM45716.1 | 1.12 | 20.1 | stress.biotic | Class IV chitinase [ *Pyrus pyrifolia* ] | 5E-159 |
| ppa023964m | gi|224144330| | ADL36716.1 | 1.11 | 27.3.22 | RNA.regulation of transcription.HB,Homeobox transcription factor family | HD domain class transcription factor [ *Malus x domestica* ] | 5E-140 |
| ppa004869m | gi|224133898| | NP_200824.1 | 1.11 | 29.5.4 | protein.degradation.aspartate protease | Aspartyl aminopeptidase, putative [ *Arabidopsis thaliana* ] | 0 |
| ppa007468m | gi|218184262| | ABF95025.1 | 1.11 | 27.3.99 | RNA.regulation of transcription.unclassified | Zinc finger family protein, putative, expressed [ *Oryza sativa Japonica Group* ] | 6E-155 |
| ppa022127m | gi|296085068| | XP_002524591.1 | 1.11 | 35.2 | not assigned.unknown | Catalytic, putative [ *Ricinus communis* ] | 0 |
| ppa002726m | gi|147858172| | CAN83928.1 | 1.10 | 35.2 | not assigned.unknown | Hypothetical protein VITISV_036910 [ *Vitis vinifera* ] | 0 |
| ppa012896m | gi|297740141| | ACF06620.1 | 1.10 | 29.2.1.2.1.14 | protein.synthesis.ribosomal protein.eukaryotic.40S subunit.S14 | Ribosomal protein S14 [ *Elaeis guineensis* ] | 3E-73 |
| ppa005919m | gi|255558958| | NP_851219.1 | 1.10 | 31.1 | cell.organisation | Myosin heavy chain-related [ *Arabidopsis thaliana* ] | 8E-167 |
| ppa013294m | gi|225463325| | ABS72193.1 | 1.10 | 34.1.1 | transport.p- and v-ATPases.H+-transporting two-sector ATPase | Vacuolar proton pump subunit F [ *Corchorus olitorius* ] | 2E-64 |
| ppa011679m | gi|255539475| | XP_002510802.1 | 1.10 | 35.2 | not assigned.unknown | Pre-mrna cleavage factor im, 25kd subunit, putative [ *Ricinus communis* ] | 2E-115 |
| ppa011295m | gi|225447109| | XP_002321145.1 | 1.10 | 27.3.44 | RNA.regulation of transcription.Chromatin Remodeling Factors | Ebs-bah-phd domain-containing protein [ *Populus trichocarpa* ] | 2E-118 |
| ppa003858m | gi|224071926| | NP_564815.1 | 1.10 | 29.5 | protein.degradation | Protease-associated (PA) domain-containing protein [ *Arabidopsis thaliana* ] | 0 |
| ppa013160m | gi|224057529| | CAN83221.1 | 1.09 | 35.2 | not assigned.unknown | Hypothetical protein VITISV_031365 [ *Vitis vinifera* ] | 5E-31 |
| ppa004837m | gi|297735345| | XP_002532228.1 | 1.09 | 34.1.1.1 | transport.p- and v-ATPases.H+-transporting two-sector ATPase.subunit B | TRANSPARENT TESTA 12 protein, putative [ *Ricinus communis* ] | 7E-167 |
| ppa002958m | gi|225428796| | NP_565377.1 | 1.09 | 35.1.5 | not assigned.no ontology.pentatricopeptide (PPR) repeat-containing protein | Pentatricopeptide (PPR) repeat-containing protein [ *Arabidopsis thaliana* ] | 5E-155 |
| ppa010078m | gi|255645717| | AAC34983.1 | 1.09 | 1.1.1.1 | PS.lightreaction.photosystem II.LHC-II | Light harvesting chlorophyll A/B binding protein [ *Prunus persica* ] | 3E-152 |
| ppa000795m | gi|225456581| | NP_189619.2 | 1.09 | 35.1 | not assigned.no ontology | NLI interacting factor (NIF) family protein [ *Arabidopsis thaliana* ] | 1E-42 |
| ppa009077m | gi|255563653| | ADL36593.1 | 1.09 | 27.3.6 | RNA.regulation of transcription.bHLH,Basic Helix-Loop-Helix family | BHLH domain class transcription factor [ *Malus x domestica* ] | 9E-97 |
| ppa017898m | gi|302143268| | NP_192259.1 | 1.09 | 31.1 | cell.organisation | Ankyrin repeat family protein [ *Arabidopsis thaliana* ] | 3E-107 |
| ppa003823m | gi|225425320| | NP_200987.1 | 1.09 | 30.3 | signalling.calcium | Calnexin 1 (CNX1) [ *Arabidopsis thaliana* ] | 0 |
| ppa013792m | gi|297788304| | ACG30677.1 | 1.09 | 28.1.3 | DNA.synthesis/chromatin structure.histone | Histone H4 [ *Zea mays* ] | 6.00E-63 |
| ppa006537m | gi|118175929| | ABW38331.1 | 1.08 | 1.3.7 | PS.calvin cycle.FBPase | Chloroplast fructose-1,6-bisphosphatase II [ *Fragaria x ananassa* ] | 0 |
| ppa007210m | gi|225426552| | XP_002518926.1 | 1.08 | 27.4 | RNA.RNA binding | RNA-binding protein, putative [ *Ricinus communis* ] | 5E-138 |
| ppa001500m | gi|255539268| | XP_002510699.1 | 1.08 | 29.5.11.4.2 | protein.degradation.ubiquitin.E3.RING | Ubiquitin-protein ligase, putative [ *Ricinus communis* ] | 0 |
| ppa002753m | gi|225450263| | XP_002531707.1 | 1.08 | 29.7.5 | protein.glycosylation.alpha-1,3-mannosyl-glycoprotein-beta-1,2-N-acetylglucosaminyltransferase(GnTI) | Beta-hexosaminidase, putative [ *Ricinus communis* ] | 0 |
| ppa005961m | gi|118486628| | XP_002273988.1 | 1.08 | 29.5.4 | protein.degradation.aspartate protease | Aspartic proteinase Asp1 [ *Vitis vinifera* ] | 0 |
| ppa009703m | gi|72256932| | AAZ67353.1 | 1.07 | 15.2 | metal handling.binding, chelation and storage | Chloroplast ferritin [ *Malus x domestica* ] | 9E-159 |
| ppa006733m | gi|255637121| | AAC36698.1 | 1.07 | 29.4 | protein.postranslational modification | Protein phosphatase-2C [ *Mesembryanthemum crystallinum* ] | 7E-148 |
| ppa001477m | gi|296082731| | XP_002513397.1 | 1.07 | 17.1.1 | hormone metabolism.abscisic acid.synthesis-degradation | Molybdopterin cofactor sulfurase, putative [ *Ricinus communis* ] | 0 |
| ppa004292m | gi|225455316| | NP_197822.1 | 1.07 | 28.99 | DNA.unspecified | 3'-5' exonuclease domain-containing protein [ *Arabidopsis thaliana* ] | 0 |
| ppa005662m | gi|225452181| | NP_851109.1 | 1.07 | 34.3 | transport.amino acids | LHT1; amino acid transmembrane transporter [ *Arabidopsis thaliana* ] | 0 |
| ppa012946m | gi|225429410| | XP_002516491.1 | 1.07 | 35.2 | not assigned.unknown | Glutamyl-trna(Gln) amidotransferase subunit C, putative [ *Ricinus communis* ] | 2E-39 |
| ppa001467m | gi|224109624| | ACM89533.1 | 1.07 | 29.4.1.57 | protein.postranslational modification.kinase.receptor like cytoplasmatic kinase VII | Protein kinase [ *Glycine max* ] | 0 |
| ppa009581m | gi|66271031| | AAY43793.1 | 1.07 | 35.2 | not assigned.unknown | E6-4 [ *Gossypium hirsutum* ] | 6E-137 |
| ppa004767m | gi|225435336| | XP_002285216.1 | 1.07 | 35.2 | not assigned.unknown | Protein-tyrosine sulfotransferase [ *Vitis vinifera* ] | 0 |
| ppa013370m | gi|83853951|g | ABC47922.1 | 1.07 | 20.1 | stress.biotic | Pathogenesis-related protein 1a [ *Malus x domestica* ] | 2E-89 |
| ppa011631m | gi|255564659| | NP_192004.1 | 1.07 | 35.2 | not assigned.unknown | MEE47 (maternal effect embryo arrest 47); transcription factor [ *Arabidopsis thaliana* ] | 4E-25 |
| ppa009449m | gi|225449218| | NP_565918.1 | 1.07 | 26.13 | misc.acid and other phosphatases | Acid phosphatase class B family protein [ *Arabidopsis thaliana* ] | 2E-61 |
| ppa003410m | gi|224066587| | XP_002302150.1 | 1.07 | 33.99 | development.unspecified | NAC domain protein, IPR003441 [ *Populus trichocarpa* ] | 0 |
| ppa008069m | gi|60280213| | AAX16491.1 | 1.06 | 16.8.3.1 | secondary metabolism.flavonoids.dihydroflavonols.dihydroflavonol 4-reductase | Dihydroflavonol 4-reductase [ *Crataegus monogyna* ] | 0 |
| ppa002778m | gi|94421690| | BAF98289.1 | 1.06 | 16.1.1.1 | secondary metabolism.isoprenoids.non-mevalonate pathway.DXS | 1-deoxy-D-xylulose 5-phosphate synthase [ *Hevea brasiliensis* ] | 0 |
| ppa003813m | gi|255547277| | NP_200933.2 | 1.06 | 35.1 | not assigned.no ontology | Atgus1 (Arabidopsis thaliana glucuronidase 1); beta-glucuronidase [ ] | 0 |
| ppa012752m | gi|182407844| | ACB87913.1 | 1.06 | 29.5.11.4.3.1 | protein.degradation.ubiquitin.E3.SCF.SKP | SKP1-like protein 1 [ *Malus x domestica* ] | 7E-85 |
| ppa005215m | gi|297736559| | XP_002520778.1 | 1.06 | 26.13 | misc.acid and other phosphatases | Multiple inositol polyphosphate phosphatase 1 precursor, putative [ *Ricinus communis* ] | 0 |
| ppa005757m | gi|255543657| | XP_002877006.1 | 1.06 | 27.3.99 | RNA.regulation of transcription.unclassified | Zinc finger family protein [ *Arabidopsis lyrata subsp. Lyrata* ] | 0 |
| ppa009790m | gi|225448990| | XP_002273467.2 | 1.06 | 21.1 | redox.thioredoxin | Thioredoxin-like 3-2, chloroplastic-like [ *Vitis vinifera* ] | 3E-141 |
| ppa000568m | gi|225465288| | XP_002270977.1 | 1.06 | 13.1.3.4.13 | amino acid metabolism.synthesis.aspartate family.methionine.methionine S-methyltransferase | Methionine S-methyltransferase-like [ *Vitis vinifera* ] | 0 |
| ppa006284m | gi|224073138| | XP_002303990.1 | 1.06 | 35.2 | not assigned.unknown | Predicted protein [ *Populus trichocarpa* ] | 0 |
| ppa013381m | gi|53749476| | AAU90329.1 | 1.06 | 35.2 | not assigned.unknown | Hypothetical protein SDM1_56t00007 [ *Solanum demissum* ] | 3E-58 |
| ppa007281m | gi|297738062| | AAF98566.1 | 1.06 | 31.1 | cell.organisation | Contains similarity to cobw protein from Rhodobacter capsulatus gi|7448322 [ *Arabidopsis thaliana* ] | 2E-141 |
| ppa008628m | gi|224054408| | NP_188773.1 | 1.06 | 35.2 | not assigned.unknown | Electron carrier/ oxidoreductase [ *Arabidopsis thaliana* ] | 2E-145 |
| ppa008987m | gi|224075359| | NP_174376.1 | 1.06 | 35.1 | not assigned.no ontology | Molybdenum cofactor sulfurase family protein [ *Arabidopsis thaliana* ] | 2E-128 |
| ppa007665m | gi|225469850| | XP_002531778.1 | 1.06 | 27.4 | RNA.RNA binding | Plasminogen activator inhibitor 1 RNA-binding protein, putative [ *Ricinus communis* ] | 1E-94 |
| ppa009172m | gi|224132840| | ACB59355.1 | 1.05 | 29.3.5 | protein.targeting.peroxisomes | Peroxisome biogenesis factor 13 [ *Nicotiana tabacum* ] | 2E-67 |
| ppa007016m | gi|224139042| | ACG34021.1 | 1.05 | 29.5.11.20 | protein.degradation.ubiquitin.proteasom | 26S proteasome non-atpase regulatory subunit 13 [ *Zea mays* ] | 0 |
| ppa006217m | gi|224133234| | NP_563816.1 | 1.05 | 30.3 | signalling.calcium | CRT3 (CALRETICULIN 3); calcium ion binding / unfolded protein binding [ *Arabidopsis thaliana* ] | 0 |
| ppa013092m | gi|224108031| | NP_565737.1 | 1.05 | 24.2 | Biodegradation of Xenobiotics.lactoylglutathione lyase | Lactoylglutathione lyase family protein / glyoxalase I family protein [ *Arabidopsis thaliana* ] | 7E-46 |
| ppa002352m | gi|84468448| | AAU04772.1 | 1.05 | 29.2.2.3.4 | protein.synthesis.ribosome biogenesis.Pre-rRNA processing and modifications.WD-repeat proteins | WD40 [ *Cucumis melo* ] | 0 |
| ppa006136m | gi|224087031| | ABN08209.1 | 1.05 | 23.4.2 | nucleotide metabolism.phosphotransfer and pyrophosphatases.guanylate kinase | Guanylate kinase/L-type calcium channel region; Galactose oxidase, central [ *Medicago truncatula* ] | 7E-162 |
| ppa013103m | gi|225427328| | NP_190511.1 | 1.05 | 31.4 | cell.vesicle transport | Got1-like family protein [ *Arabidopsis thaliana* ] | 4E-64 |
| ppa002703m | gi|255539242| | XP_002510686.1 | 1.05 | 27.3.42 | RNA.regulation of transcription.Bromodomain proteins | Bromodomain-containing protein [ *Ricinus communis* ] | 0 |
| ppa002923m | gi|225445031| | XP_002280190.1 | 1.05 | 21.2.2 | redox.ascorbate and glutathione.glutathione | gamma-glutamyltranspeptidase 1 [ *Vitis vinifera* ] | 0 |
| ppa026720m | gi|255576987| | NP_200605.1 | 1.05 | 28.2 | DNA.repair | Methyladenine glycosylase family protein [ *Arabidopsis thaliana* ] | 1E-132 |
| ppa009034m | gi|224109896| | ACP43458.1 | 1.04 | 11.3.3 | lipid metabolism.Phospholipid synthesis.phosphatidate cytidylyltransferase | Phytol kinase [ *Lactuca sativa* ] | 1E-97 |
| ppa013539m | gi|225463799| | XP_002871347.1 | 1.04 | 27.2 | RNA.transcription | Positive transcription elongation factor/ zinc ion binding protein [ *Arabidopsis lyrata subsp. Lyrata* ] | 5E-52 |
| ppa006429m | gi|225427324| | NP_850244.1 | 1.03 | 23.1.3 | nucleotide metabolism.synthesis.PRS-PP | Ribose-phosphate pyrophosphokinase 1 / phosphoribosyl diphosphate synthetase 1 (PRSI) [ *Arabidopsis thaliana* ] | 0 |
| ppa003686m | gi|225441397| | ABF57287.1 | 1.03 | 35.2 | not assigned.unknown | At5g06830 [ *Arabidopsis thaliana* ] | 0 |
| ppa007330m | gi|224122328| | XP_002867562.1 | 1.03 | 18.7 | Co-factor and vitamine metabolism.iron-sulphur clusters | ATSUFE/CPSUFE/EMB1374 [ *Arabidopsis lyrata subsp. Lyrata* ] | 4E-112 |
| ppa012787m | gi|225448325| | ACA64832.1 | 1.03 | 35.2 | not assigned.unknown | SKIP interacting protein 15 [ *Oryza sativa* ] | 7E-38 |
| ppa006916m | gi|255543014| | XP_002512570.1 | 1.03 | 13.1.3.4 | amino acid metabolism.synthesis.aspartate family.methionine | S-adenosylmethionine synthetase, putative [ *Ricinus communis* ] | 0 |
| ppa004469m | gi|255539108| | ADN34131.1 | 1.03 | 29.1.30 | protein.aa activation.pseudouridylate synthase | Trna pseudouridine synthase family protein [ *Cucumis melo subsp. Melo* ] | 0 |
| ppa007139m | gi|255548808| | CAN73956.1 | 1.03 | 35.2 | not assigned.unknown | Hypothetical protein VITISV_026134 [ *Vitis vinifera* ] | 3E-108 |
| ppa012319m | gi|30681471|r | NP_850016.1 | 1.03 | 20.2.99 | stress.abiotic.unspecified | RD2 [ *Arabidopsis thaliana* ] | 2E-91 |
| ppa011912m | gi|255563310| | XP_002522658.1 | 1.03 | 35.2 | not assigned.unknown | Magnesium-dependent phosphatase, putative [ *Ricinus communis* ] | 5E-108 |
| ppa012422m | gi|118487739| | XP_002889865.1 | 1.02 | 35.2 | not assigned.unknown | Partner of Y14-mago [ *Arabidopsis lyrata subsp. Lyrata* ] | 5E-61 |
| ppa004338m | gi|11094371| | AAG29596.1 | 1.02 | 29.4 | protein.postranslational modification | Ser/Thr specific protein phosphatase 2A B regulatory subunit beta isoform [ *Medicago sativa subsp. X varia* ] | 0 |
| ppa001688m | gi|255540511| | NP_194200.1 | 1.02 | 29.5.11 | protein.degradation.ubiquitin | Ubiquitin-associated (UBA)/TS-N domain-containing protein / octicosapeptide/Phox/Bemp1 (PB1) domain-containing protein [ *Arabidopsis thaliana* ] | 2E-153 |
| ppa008152m | gi|224145116| | NP_192728.2 | 1.01 | 26.8 | misc.nitrilases, *nitrile lyases, berberine bridge enzymes, reticuline oxidases, troponine reductases | MES12 (methyl esterase 12); hydrolase [ *Arabidopsis thaliana* ] | 7E-162 |
| ppa001590m | gi|224099261| | XP_006484274.1 | 1.01 | 20.2.1 | stress.abiotic.heat | heat shock protein 83-like [ *Citrus sinensis* ] | 0 |
| ppa002887m | gi|225443912| | XP_002863419.1 | 1.01 | 27.4 | RNA.RNA binding | KH domain-containing protein [ *Arabidopsis lyrata subsp. Lyrata* ] | 0 |
| ppa011809m | gi|44889030| | AAS46231.1 | 1.01 | 29.4 | protein.postranslational modification | Methionine sulfoxide reductase A [ *Populus trichocarpa x Populus deltoides* ] | 3E-82 |
| ppa000308m | gi|224119992| | XP_002513693.1 | 1.01 | 29.5.5 | protein.degradation.serine protease | Tripeptidyl peptidase II, putative [ *Ricinus communis* ] | 0 |
| ppa000102m | gi|225441991| | EOY27383.1 | 1.00 | 30.4.1 | signalling.phosphinositides.phosphatidylinositol-4-phosphate 5-kinase | Phosphatidylinositol-4-phosphate 5-kinase family protein, putative isoform 1 [ *Theobroma cacao* ] | 0 |
| ppa011229m | gi|255543290| | AAO85557.1 | 1.00 | 1.1.2.2 | PS.lightreaction.photosystem I.PSI polypeptide subunits | Photosystem I subunit XI [ *Nicotiana attenuata* ] | 2E-121 |
| ppa001358m | gi|225425480| | XP_002514933.1 | 1.00 | 26.3 | misc.gluco-, galacto- and mannosidases | Mannosyl-oligosaccharide glucosidase, putative [ *Ricinus communis* ] | 0 |
| ppa011418m | gi|224072753| | XP_002889267.1 | 1.00 | 35.2 | not assigned.unknown | Hypothetical protein ARALYDRAFT_477150 [ *Arabidopsis lyrata subsp. Lyrata* ] | 4E-61 |
| ppa003286m | gi|87240748| | XP_004146581.1 | 1.00 | 9.2.1.2 | mitochondrial electron transport / ATP synthesis.NADH-DH.type II.external | NAD(P)H dehydrogenase B3, mitochondrial-like [ *Cucumis sativus* ] | 0 |
| ppa009133m | gi|224082053| | XP_002513981.1 | 1.00 | 27.4 | RNA.RNA binding | Ribonucleoprotein, chloroplast, putative [ *Ricinus communis* ] | 1E-85 |
| ppa011151m | gi|255634739| | XP_002779971.1 | -1.00 | 35.2 | not assigned.unknown | High mobility group protein B1, putative [ *Perkinsus marinus ATCC 50983* ] | 0 |
| ppa003189m | gi|255545299| | NP_201260.1 | -1.00 | 35.1 | not assigned.no ontology | Loricrin-related [ *Arabidopsis thaliana* ] | 6E-131 |
| ppa006276m | gi|296082781| | XP_002513417.1 | -1.00 | 30.5 | signalling.G-proteins | GTP-binding protein, putative [ *Ricinus communis* ] | 3E-144 |
| ppa012293m | gi|297742118| | CBI33905.3 | -1.00 | 35.2 | not assigned.unknown | Unnamed protein product [ *Vitis vinifera* ] | 0 |
| ppa007920m | gi|224145057| | XP_002525534.1 | -1.00 | 35.1 | not assigned.no ontology | Protein phosphatase methylesterase, putative [ *Ricinus communis* ] | 0 |
| ppa003086m | gi|167374781| | ABZ79223.1 | -1.00 | 5.2 | fermentation.PDC | Pyruvate decarboxylase [ *Prunus armeniaca* ] | 0 |
| ppa014283m | - | unkown | -1.00 | 35.2 | not assigned.unknown | Unkown | 0 |
| ppa008005m | gi|224093434| | NP_201203.2 | -1.00 | 23.2 | nucleotide metabolism.degradation | SAL1; 3'(2'),5'-bisphosphate nucleotidase/ inositol or phosphatidylinositol phosphatase [ *Arabidopsis thaliana* ] | 0 |
| ppa001969m | gi|255542364| | XP_002512245.1 | -1.01 | 29.4 | protein.postranslational modification | Kinase, putative [ *Ricinus communis* ] | 0 |
| ppa003899m | gi|255540003| | XP_002511066.1 | -1.01 | 30.3 | signalling.calcium | Calnexin, putative [ *Ricinus communis* ] | 0 |
| ppa003043m | gi|225461963| | XP_002530422.1 | -1.01 | 29.4 | protein.postranslational modification | Serine/threonine protein kinase, putative [ *Ricinus communis* ] | 0 |
| ppa009192m | gi|225451281| | NP_850995.1 | -1.01 | 35.2 | not assigned.unknown | Binding [ *Arabidopsis thaliana* ] | 0 |
| ppa007879m | gi|255544039| | XP_002513082.1 | -1.01 | 31.1 | cell.organisation | Annexin, putative [ *Ricinus communis* ] | 0 |
| ppa002599m | gi|225431818| | XP_002516980.1 | -1.01 | 35.1.12 | not assigned.no ontology.pumilio/Puf RNA-binding domain-containing protein | Protein penguin, putative [ *Ricinus communis* ] | 0 |
| ppa009615m | gi|255578055| | XP_002529898.1 | -1.01 | 27.1 | RNA.processing | Small nuclear ribonucleoprotein-associated protein, putative [ *Ricinus communis* ] | 3E-45 |
| ppa005100m | gi|224067066| | NP_194186.1 | -1.01 | 29.3.4.99 | protein.targeting.secretory pathway.unspecified | Clathrin adaptor complexes medium subunit family protein [ *Arabidopsis thaliana* ] | 0 |
| ppa011217m | gi|255576818| | XP_002529295.1 | -1.01 | 27.3.6 | RNA.regulation of transcription.bHLH,Basic Helix-Loop-Helix family | DNA binding protein, putative [ *Ricinus communis* ] | 0 |
| ppa013572m | gi|224128924| | XP_002514854.1 | -1.01 | 27.3.11 | RNA.regulation of transcription.C2H2 zinc finger family | Transcription factor, putative [ *Ricinus communis* ] | 6E-153 |
| ppa010791m | gi|255574159| | XP_002527995.1 | -1.01 | 29.5.11.20 | protein.degradation.ubiquitin.proteasom | Proteasome subunit beta type 6,9, putative [ *Ricinus communis* ] | 8E-133 |
| ppa008256m | gi|225461315| | NP_565711.1 | -1.01 | 1.1.4 | PS.lightreaction.ATP synthase | ATP synthase protein I -related [ *Arabidopsis thaliana* ] | 0 |
| ppa005881m | gi|255583653| | XP_002532581.1 | -1.02 | 29.4 | protein.postranslational modification | CBL-interacting serine/threonine-protein kinase, putative [ *Ricinus communis* ] | 1E-125 |
| ppa000869m | gi|224115854| | XP_002518434.1 | -1.02 | 29.1.4 | protein.aa activation.leucine-tRNA ligase | Leucyl-trna synthetase, putative [ *Ricinus communis* ] | 3E-100 |
| ppa024137m | gi|255552299| | XP_002517194.1 | -1.02 | 29.5.11.4.2 | protein.degradation.ubiquitin.E3.RING | Protein binding protein, putative [ *Ricinus communis* ] | 2E-83 |
| ppa007786m | gi|225463960| | XP_002521956.1 | -1.02 | 27.3.67 | RNA.regulation of transcription.putative transcription regulator | DNA binding protein, putative [ *Ricinus communis* ] | 0 |
| ppa012560m | gi|255627933| | NP_566991.2 | -1.02 | 20.2.2 | stress.abiotic.cold | Universal stress protein (USP) family protein [ *Arabidopsis thaliana* ] | 0 |
| ppa008977m | gi|225451665| | AAP45159.1 | -1.02 | 35.2 | not assigned.unknown | Plant viral-response family protein [ *Solanum bulbocastanum* ] | 0 |
| ppa008744m | gi|29468504| | NP_567672.1 | -1.02 | 1.1.40 | PS.lightreaction.cyclic electron flow-chlororespiration | PGR5-LIKE A [ *Arabidopsis thaliana* ] | 6E-68 |
| ppa011863m | gi|225460590| | XP_003634526.1 | -1.02 | 35.2 | not assigned.unknown | Hypothetical protein [ *Vitis vinifera* ] | 7.00E-160 |
| ppa003013m | gi|255564966| | EOY17161.1 | -1.03 | 29.2.3 | protein.synthesis.initiation | Eukaryotic translation initiation factor SUI1 family protein isoform 1 [ *Theobroma cacao* ] | 0 |
| ppa015440m | gi|224140401| | NP_566341.1 | -1.03 | 29.4.1.57 | protein.postranslational modification.kinase.receptor like cytoplasmatic kinase VII | Protein kinase family protein [ *Arabidopsis thaliana* ] | 8E-143 |
| ppa007547m | gi|3560529| | XP_002533800.1 | -1.03 | 1.1.5.3 | PS.lightreaction.other electron carrier (ox/red).ferredoxin reductase | Ferredoxin--NADP reductase, putative [ *Ricinus communis* ] | 0 |
| ppa001208m | gi|297746431| | XP_002530869.1 | -1.03 | 29.5.11.4.99 | protein.degradation.ubiquitin.E3.unspecified | Ubiquitin-protein ligase bre-1, putative [ *Ricinus communis* ] | 0 |
| ppa005536m | gi|255538012| | XP_002510071.1 | -1.03 | 29.5.11.4.2 | protein.degradation.ubiquitin.E3.RING | E3 ubiquitin ligase PUB14, putative [ *Ricinus communis* ] | 6E-75 |
| ppa000078m | gi|296089008| | NP_567189.5 | -1.03 | 33.99 | development.unspecified | Binding / protein binding / zinc ion binding [ *Arabidopsis thaliana* ] | 0 |
| ppa003388m | gi|297745402| | ABC87761.1 | -1.03 | 17.2.3 | hormone metabolism.auxin.induced-regulated-responsive-activated | JAR1-like protein [ *Nicotiana attenuata* ] | 0 |
| ppa002596m | gi|225447229| | XP_002532772.1 | -1.03 | 35.1.5 | not assigned.no ontology.pentatricopeptide (PPR) repeat-containing protein | Pentatricopeptide repeat-containing protein, putative [ *Ricinus communis* ] | 3E-16 |
| ppa003261m | gi|302143904| | XP_002516670.1 | -1.03 | 29.5.5 | protein.degradation.serine protease | ATP-dependent clp protease ATP-binding subunit clpx, putative [ *Ricinus communis* ] | 3E-137 |
| ppa006364m | gi|224284516| | BAA35121.1 | -1.03 | 20.2.1 | stress.abiotic.heat | Dnaj homolog [ *Salix gilgiana* ] | 3E-137 |
| ppa010098m | gi|224110350| | XP_002516969.1 | -1.03 | 33.99 | development.unspecified | DAG protein, chloroplast precursor, putative [ *Ricinus communis* ] | 0 |
| ppa005819m | gi|225444926| | NP_566005.1 | -1.03 | 27.3.99 | RNA.regulation of transcription.unclassified | Mitochondrial transcription termination factor-related / mterf-related [ *Arabidopsis thaliana* ] | 0 |
| ppa004700m | gi|297744366| | NP_189128.2 | -1.03 | 29.3.4.99 | protein.targeting.secretory pathway.unspecified | SEC14 cytosolic factor, putative / phosphoglyceride transfer protein, putative [ *Arabidopsis thaliana* ] | 0 |
| ppa007790m | gi|224130494| | XP_002532663.1 | -1.04 | 34.9 | transport.metabolite transporters at the mitochondrial membrane | Grave disease carrier protein, putative [ *Ricinus communis* ] | 0 |
| ppa014368m | gi|255557133| | XP_002519598.1 | -1.04 | 35.2 | not assigned.unknown | Conserved hypothetical protein [ *Ricinus communis* ] | 4.00E-44 |
| ppa012651m | gi|82492265| | ABB78006.1 | -1.04 | 27.1.19 | RNA.processing.ribonucleases | Major allergen Pru p 1 [ *Prunus persica* ] | 6E-56 |
| ppa013202m | gi|225461156| | XP_002316965.1 | -1.04 | 28.1.3 | DNA.synthesis/chromatin structure.histone | Histone H2 [ *Populus trichocarpa* ] | 4E-53 |
| ppa003954m | gi|224134268| | XP_002521570.1 | -1.04 | 29.5.11.4.2 | protein.degradation.ubiquitin.E3.RING | Ubiquitin-protein ligase, putative [ *Ricinus communis* ] | 2E-66 |
| ppa007574m | gi|224122914| | XP_002528712.1 | -1.04 | 26.28 | misc.GDSL-motif lipase | Carboxylic ester hydrolase, putative [ *Ricinus communis* ] | 7E-46 |
| ppa009245m | gi|187372986| | ACD03227.1 | -1.04 | 10.7 | cell wall.modification | Xyloglucan endotransglucosylase/hydrolase 3 [ *Malus x domestica* ] | 3E-154 |
| ppa000480m | gi|225432963| | ABA99200.2 | -1.04 | 35.1 | not assigned.no ontology | MATH domain containing protein, expressed [ *Oryza sativa Japonica Group* ] | 0 |
| ppa009431m | gi|255574251| | EOY17070.1 | -1.04 | 26.3 | misc. other Ferredoxins and Rieske domain | Rieske domain-containing protein isoform 1 [ *Theobroma cacao* ] | 4.00E-123 |
| ppa003464m | gi|225449991| | XP_002517755.1 | -1.04 | 35.2 | not assigned.unknown | Gd2b, putative [ *Ricinus communis* ] | 3E-108 |
| ppa013158m | gi|225451247| | XP_002518257.1 | -1.04 | 29.2.1.2.1.24 | protein.synthesis.ribosomal protein.eukaryotic.40S subunit.S24 | 40S ribosomal protein S24, putative [ *Ricinus communis* ] | 8.2 |
| ppa012019m | gi|255564158| | XP_002523076.1 | -1.04 | 35.2 | not assigned.unknown | Conserved hypothetical protein [ *Ricinus communis* ] | 9.00E-135 |
| ppa009719m | gi|224132810| | NP_001151864.1 | -1.04 | 35.2 | not assigned.unknown | CCT motif family protein [ *Zea mays* ] | 0 |
| ppa011692m | gi|224090631| | CBH13171.1 | -1.05 | 29.5.11.3 | protein.degradation.ubiquitin.E2 | Ubiquitin-protein ligase, putative [ *Trypanosoma brucei gambiense DAL972* ] | 3E-86 |
| ppa008546m | gi|118488826| | XP_002510744.1 | -1.05 | 1.1.1.1 | PS.lightreaction.photosystem II.LHC-II | Chlorophyll A/B binding protein, Lhcb7 [ *Ricinus communis* ] | 0 |
| ppa021889m | gi|224142461| | XP_002532502.1 | -1.05 | 34.98 | transport.membrane system unknown | Regulatory protein uhpc, putative [ *Ricinus communis* ] | 4E-163 |
| ppa008840m | gi|255638977| | XP_002523640.1 | -1.05 | 35.1 | not assigned.no ontology | WD-repeat protein, putative [ *Ricinus communis* ] | 2E-134 |
| ppa1027217m | gi|296082991| | XP_002526088.1 | -1.05 | 27.3.99 | RNA.regulation of transcription.unclassified | Zinc finger protein, putative [ *Ricinus communis* ] | 0 |
| ppa003268m | gi|224078844| | XP_002524688.1 | -1.05 | 29.5.11.3 | protein.degradation.ubiquitin.E2 | Ubiquitin-protein ligase, putative [ *Ricinus communis* ] | 0 |
| ppa012494m | gi|296081545| | XP_002530278.1 | -1.05 | 26.21 | misc.protease inhibitor/seed storage/lipid transfer protein (LTP) family protein | Lipid binding protein, putative [ *Ricinus communis* ] | 8E-164 |
| ppa005813m | gi|255543128| | XP_002512627.1 | -1.05 | 27.3.11 | RNA.regulation of transcription.C2H2 zinc finger family | Nucleic acid binding protein, putative [ *Ricinus communis* ] | 0 |
| ppa009988m | gi|225449370| | XP_002524232.1 | -1.05 | 35.1 | not assigned.no ontology | Protein YIPF1, putative [ *Ricinus communis* ] | 7E-43 |
| ppa009571m | gi|255542702| | ACJ39219.1 | -1.05 | 35.1.19 | not assigned.no ontology.C2 domain-containing protein | Cold-regulated protein [ *Glycine max* ] | 0 |
| ppa007732m | gi|27597158| | BAC55113.1 | -1.05 | 22.1.2 | polyamine metabolism.synthesis.SAM decarboxylase | S-adenosylmethionine decarboxylase [ *Malus x domestica* ] | 1E-60 |
| ppa005374m | gi|224069026| | XP_002302882.1 | -1.05 | 35.2 | not assigned.unknown | Predicted protein [ *Populus trichocarpa* ] | 0 |
| ppa021815m | gi|255626741| | NP_001237429.1 | -1.05 | 35.2 | not assigned.unknown | Uncharacterized protein LOC100305838 [ *Glycine max* ] | 1.00E-108 |
| ppa008164m | gi|5531418|em | CAB50915.1 | -1.05 | 35.2 | not assigned.unknown | Outer envelope protein [ *Pisum sativum* ] | 0 |
| ppa008881m | gi|225469097| | AAD24540.1 | -1.05 | 31.1 | cell.organisation | Vacuole-associated annexin vcab42 [ *Nicotiana tabacum* ] | 9E-180 |
| ppa002256m | gi|225432604| | XP_002517212.1 | -1.05 | 11.1.9 | lipid metabolism.FA synthesis and FA elongation.long chain fatty acid CoA ligase | Acyl-coa synthetase [ *Ricinus communis* ] | 0 |
| ppa003005m | gi|225445541| | XP_002515505.1 | -1.05 | 35.1.21 | not assigned.no ontology.epsin N-terminal homology (ENTH) domain-containing protein | Clathrin assembly protein, putative [ *Ricinus communis* ] | 0 |
| ppa000534m | gi|297742578| | XP_002969734.1 | -1.05 | 35.2 | not assigned.unknown | Hypothetical protein SELMODRAFT_92955 [ *Selaginella moellendorffii* ] | 0 |
| ppa007308m | gi|225449309| | CAN66597.1 | -1.05 | 35.2 | not assigned.unknown | Hypothetical protein VITISV_030013 [ *Vitis vinifera* ] | 1E-74 |
| ppa011247m | gi|225455663| | XP_002263260.1 | -1.05 | 35.2 | not assigned.unknown | Uncharacterized protein LOC100245789 [ *Vitis vinifera* ] | 0 |
| ppb006523m | gi|217073944| | XP_002533669.1 | -1.05 | 17.5.1 | hormone metabolism.ethylene.synthesis-degradation | Flavonol synthase/flavanone 3-hydroxylase, putative [ *Ricinus communis* ] | 0 |
| ppa005426m | gi|118483001| | XP_002522052.1 | -1.06 | 13.1.3.5.3 | amino acid metabolism.synthesis.aspartate family.lysine.LL-diaminopimelic acid aminotransferase | Transaminase mtne, putative [ *Ricinus communis* ] | 2E-136 |
| ppa012755m | gi|225457881| | ABS17589.1 | -1.06 | 29.5.11.4.3.1 | protein.degradation.ubiquitin.E3.SCF.SKP | SKP1 component-like 1 [ *Humulus lupulus* ] | 3E-175 |
| ppa007019m | gi|225457064| | XP_002511419.1 | -1.06 | 29.4 | protein.postranslational modification | Protein kinase atmrk1, putative [ *Ricinus communis* ] | 0.062 |
| ppa009511m | gi|255636760| | ACF06527.1 | -1.06 | 34.99 | transport.misc | Secretory carrier membrane protein [ *Elaeis guineensis* ] | 0 |
| ppa008908m | gi|255586792| | ABN05735.1 | -1.06 | 27.3.80 | RNA.regulation of transcription.zf-HD | Homeobox domain, ZF-HD class; ZF-HD homeobox protein Cys/His-rich dimerisation region; Homeodomain-like [ *Medicago truncatula* ] | 5E-42 |
| ppa000988m | gi|255555995| | XP_002519032.1 | -1.06 | 27.4 | RNA.RNA binding | Double-stranded RNA binding protein, putative [ *Ricinus communis* ] | 0.33 |
| ppa008962m | gi|225429234| | XP_002513130.1 | -1.06 | 34.2 | transport.porins | Mitochondrial import receptor subunit tom40, putative [ *Ricinus communis* ] | 0 |
| ppa004917m | gi|255550093| | ACG50004.1 | -1.06 | 35.2 | not assigned.unknown | Salt responsive protein 2 [ *Solanum lycopersicum* ] | 2E-104 |
| ppa010301m | gi|147789492| | XP_002876384.1 | -1.06 | 35.1 | not assigned.no ontology | VQ motif-containing protein [ *Arabidopsis lyrata subsp. Lyrata* ] | 0 |
| ppa010379m | gi|29466641| | BAC66786.1 | -1.06 | 10.7 | cell wall.modification | Expansin [ *Prunus persica* ] | 4E-74 |
| ppa006706m | gi|225461726| | NP_188104.2 | -1.07 | 35.1 | not assigned.no ontology | NHL repeat-containing protein [ *Arabidopsis thaliana* ] | 3E-149 |
| ppa001709m | gi|225444025| | NP_174335.4 | -1.07 | 15.2 | metal handling.binding, chelation and storage | SIT4 phosphatase-associated family protein [ *Arabidopsis thaliana* ] | 9E-97 |
| ppa010500m | gi|118487925| | XP_002532881.1 | -1.07 | 33.99 | development.unspecified | DAG protein, chloroplast precursor, putative [ *Ricinus communis* ] | 4E-167 |
| ppa005865m | gi|296089619| | XP_002525241.1 | -1.07 | 35.1 | not assigned.no ontology | WD-repeat protein, putative [ *Ricinus communis* ] | 0 |
| ppa000274m | gi|284026888| | ADB66335.1 | -1.07 | 20.1 | stress.biotic | CC-NBS-LRR protein [ *Quercus suber* ] | 8E-178 |
| ppa009326m | gi|225427213| | XP_002280380.1 | -1.07 | 34.9 | transport.metabolite transporters at the mitochondrial membrane | Mitochondrial carnitine/acylcarnitine carrier-like protein-like isoform 1 [ *Vitis vinifera* ] | 0 |
| ppa010044m | gi|225464442| | XP_002526392.1 | -1.07 | 35.2 | not assigned.unknown | Conserved hypothetical protein [ *Ricinus communis* ] | 8.00E-131 |
| ppa020036m | gi|15242612| | NP_198839.1 | -1.07 | 33.99 | development.unspecified | Nodulin-related [ *Arabidopsis thaliana* ] | 0 |
| ppa011426m | gi|224054420| | XP_002517637.1 | -1.07 | 35.2 | not assigned.unknown | GTP binding protein, putative [ *Ricinus communis* ] | 0.24 |
| ppa006659m | gi|255638871| | BAA01091.1 | -1.07 | 29.4 | protein.postranslational modification | Casein kinase II catalytic subunit [ *Arabidopsis thaliana* ] | 0 |
| ppa000820m | gi|225429846| | XP_002527993.1 | -1.07 | 10.1.30.2 | cell wall.precursor synthesis.sugar kinases.arabinose-1-kinase | Galactokinase, putative [ *Ricinus communis* ] | 2.2 |
| ppa010759m | gi|147837826| | XP_002510906.1 | -1.07 | 29.3.99 | protein.targeting.unknown | ATP-dependent clp protease, putative [ *Ricinus communis* ] | 0 |
| ppa013060m | gi|255553532| | XP_002517807.1 | -1.07 | 35.2 | not assigned.unknown | Conserved hypothetical protein [ *Ricinus communis* ] | 2.00E-82 |
| ppa011825m | gi|255555849| | XP_002518960.1 | -1.07 | 29.2.1.2.2.9 | protein.synthesis.ribosomal protein.eukaryotic.60S subunit.L9 | 60S ribosomal protein L9, putative [ *Ricinus communis* ] | 2E-70 |
| ppa012591m | gi|255572094| | ADB54810.1 | -1.08 | 20.2.99 | stress.abiotic.unspecified | Universal stress protein 23267 [ *Hordeum vulgare subsp. Vulgare* ] | 0 |
| ppa019726m | gi|296088515| | XP_002524432.1 | -1.08 | 27.1 | RNA.processing | Transcription initiation factor ia, putative [ *Ricinus communis* ] | 0 |
| ppa006692m | gi|225459239| | NP_567342.1 | -1.08 | 29.4 | protein.postranslational modification | Phosphotyrosyl phosphatase activator (PTPA) family protein [ *Arabidopsis thaliana* ] | 0 |
| ppa003635m | gi|255549790| | XP_002515946.1 | -1.08 | 4.2.4 | glycolysis.plastid branch.phosphofructokinase (PFK) | Phosphofructokinase, putative [ *Ricinus communis* ] | 0 |
| ppa006231m | gi|225430163| | XP_002514356.1 | -1.08 | 26.7 | misc.oxidases - copper, flavone etc | Monoxygenase, putative [ *Ricinus communis* ] | 0.25 |
| ppa010171m | gi|224105087| | XP_002534094.1 | -1.08 | 10.7 | cell wall.modification | Beta-expansin 1a precursor, putative [ *Ricinus communis* ] | 0 |
| ppa000507m | gi|225425300| | NP_196843.1 | -1.08 | 35.2 | not assigned.unknown | NEF1 (NO EXINE FORMATION 1) [ *Arabidopsis thaliana* ] | 1E-36 |
| ppa006317m | gi|255585914| | XP_002533630.1 | -1.08 | 26.11 | misc.alcohol dehydrogenases | Alcohol dehydrogenase, putative [ *Ricinus communis* ] | 0 |
| ppa010930m | gi|147804852| | XP_002524079.1 | -1.09 | 35.1 | not assigned.no ontology | Phosphatidylglycerol/phosphatidylinositol transfer protein precursor, putative [ *Ricinus communis* ] | 1E-69 |
| ppa006131m | gi|225432416| | XP_002516851.1 | -1.09 | 29.5.11.4.2 | protein.degradation.ubiquitin.E3.RING | Protein binding protein, putative [ *Ricinus communis* ] | 0 |
| ppa012625m | gi|255549960| | EOY26804.1 | -1.09 | 31.1 | cell.organisation | RHO guanyl-nucleotide exchange factor 11 [ *Theobroma cacao* ] | 4.00E-54 |
| ppa006923m | gi|255585128| | XP_002533269.1 | -1.09 | 35.2 | not assigned.unknown | Conserved hypothetical protein [ *Ricinus communis* ] | 0 |
| ppa016483m | gi|225431667| | NP_851259.1 | -1.09 | 29.4 | protein.postranslational modification | PP7 (SERINE/THREONINE PHOSPHATASE 7); protein serine/threonine phosphatase [ *Arabidopsis thaliana* ] | 3E-27 |
| ppa013319m | gi|224105963| | XP_002514168.1 | -1.09 | 29.2.1.2.1.515 | protein.synthesis.ribosomal protein.eukaryotic.40S subunit.S15A | 30S ribosomal protein S8, putative [ *Ricinus communis* ] | 0 |
| ppa001206m | gi|296081721| | XP_002516143.1 | -1.09 | 34.12 | transport.metal | Copper-transporting atpase paa1, putative [ *Ricinus communis* ] | 0 |
| ppa012298m | gi|297741266| | XP_002313121.1 | -1.09 | 21.1 | redox.thioredoxin | Thioredoxin m [ *Populus trichocarpa* ] | 2E-71 |
| ppa003820m | gi|225424643| | XP_002530045.1 | -1.09 | 29.2.3 | protein.synthesis.initiation | Translation initiation factor, putative [ *Ricinus communis* ] | 2E-21 |
| ppa000570m | gi|224092524| | XP_002515477.1 | -1.09 | 35.1 | not assigned.no ontology | WD-repeat protein, putative [ *Ricinus communis* ] | 4E-73 |
| ppa004982m | gi|225427242| | XP_002278544.1 | -1.09 | 34.16 | transport.ABC transporters and multidrug resistance systems | Protein ABCI7, chloroplastic-like [ *Vitis vinifera* ] | 0 |
| ppa006859m | gi|13431949| | XP_002530415.1 | -1.09 | 1.3.9 | PS.calvin cycle.seduheptulose bisphosphatase | Sedoheptulose-1,7-bisphosphatase, chloroplast, putative [ *Ricinus communis* ] | 0 |
| ppb007460m | gi|225445865| | EGG38044.1 | -1.09 | 35.2 | not assigned.unknown | Endonuclease/exonuclease/phosphatase family protein [ *Paenibacillus sp. HGF5* ] | 0 |
| ppa013332m | gi|224105963| | XP_002514168.1 | -1.09 | 29.2.1.2.1.515 | protein.synthesis.ribosomal protein.eukaryotic.40S subunit.S15A | 30S ribosomal protein S8, putative [ *Ricinus communis* ] | 0 |
| ppa012618m | gi|118489097| | XP_001400318.2 | -1.09 | 35.2 | not assigned.unknown | Subunit of the multiprotein cohesin complex [ *Aspergillus niger CBS 513.88* ] | 0 |
| ppa011410m | gi|296086563| | XP_002515762.1 | -1.09 | 35.1 | not assigned.no ontology | AMME syndrome candidateprotein 1 protein, putative [ *Ricinus communis* ] | 4E-50 |
| ppa007896m | gi|255541682| | ABX26124.1 | -1.09 | 35.2 | not assigned.unknown | Bypass1 [ *Nicotiana benthamiana* ] | 9E-124 |
| ppa008050m | gi|255559749| | CBI80766.1 | -1.10 | 35.2 | not assigned.unknown | Bartonella effector protein (Bep); substrate of virb T4SS [ *Bartonella sp. 1-1C* ] | 0 |
| ppa002190m | gi|225460813| | XP_002515869.1 | -1.10 | 31.1 | cell.organisation | Fimbrin, putative [ *Ricinus communis* ] | 6E-42 |
| ppa025640m | gi|2688828| | AAB88878.1 | -1.10 | 17.5.1 | hormone metabolism.ethylene.synthesis-degradation | Ethylene-forming-enzyme-like dioxygenase [ *Prunus armeniaca* ] | 5E-84 |
| ppa003934m | gi|225438345| | XP_002273522.2 | -1.10 | 9.2.1.3 | mitochondrial electron transport / ATP synthesis.NADH-DH.type II.mitochondrial | NADH dehydrogenase C1, chloroplastic/mitochondrial-like [ *Vitis vinifera* ] | 0 |
| ppa001064m | gi|255547812| | XP_002514963.1 | -1.10 | 17.7.1.2 | hormone metabolism.jasmonate.synthesis-degradation.lipoxygenase | Lipoxygenase, putative [ *Ricinus communis* ] | 1E-97 |
| ppa012640m | gi|225430097| | NP_565093.1 | -1.10 | 1.1.6 | PS.lightreaction.NADH DH | NDH-O (NAD(P)H:plastoquinone dehydrogenase complex subunit O) [ *Arabidopsis thaliana* ] | 3E-35 |
| ppa004265m | gi|255586856| | XP_002534038.1 | -1.10 | 29.5.4 | protein.degradation.aspartate protease | Aspartic proteinase nepenthesin-1 precursor, putative [ *Ricinus communis* ] | 0 |
| ppa002633m | gi|225464400| | XP_002516636.1 | -1.10 | 31.4 | cell.vesicle transport | Protein binding protein, putative [ *Ricinus communis* ] | 6E-110 |
| ppa000002m | gi|297832860| | NP_186875.2 | -1.10 | 17.2.2 | hormone metabolism.auxin.signal transduction | BIG (BIG); binding / ubiquitin-protein ligase/ zinc ion binding [ *Arabidopsis thaliana* ] | 9E-137 |
| ppa012294m | gi|224141283| | CAJ13711.1 | -1.10 | 20.2.99 | stress.abiotic.unspecified | Putative ethylene response protein [ *Capsicum chinense* ] | 0 |
| ppa004628m | gi|11133818|s | BAD24712.1 | -1.10 | 21.1 | redox.thioredoxin | Protein disulfide isomerase-like protein [ *Glycine max* ] | 5E-135 |
| ppa010700m | gi|147854692| | XP_002528449.1 | -1.10 | 11.9.4.3 | lipid metabolism.lipid degradation.beta-oxidation.enoyl CoA hydratase | Carnitine racemase, putative [ *Ricinus communis* ] | 0 |
| ppa002239m | gi|301110777| | XP_002904468.1 | -1.10 | 35.2 | not assigned.unknown | Mucin-like protein [ *Phytophthora infestans T30-4* ] | 1E-90 |
| ppa010769m | gi|297739351| | CBI29341.3 | -1.10 | 35.1.40 | not assigned.no ontology.glycine rich proteins | Unnamed protein product [ *Vitis vinifera* ] | 1.00E-155 |
| ppa009744m | gi|224067266| | XP_002510224.1 | -1.10 | 29.3.3 | protein.targeting.chloroplast | Protein translocase, putative [ *Ricinus communis* ] | 0 |
| ppa012456m | gi|224085479| | XP_002307589.1 | -1.11 | 35.2 | not assigned.unknown | Predicted protein [ *Populus trichocarpa* ] | 1E-124 |
| ppa007451m | gi|224103333| | XP_002512983.1 | -1.11 | 35.1 | not assigned.no ontology | Clathrin binding protein, putative [ *Ricinus communis* ] | 6E-152 |
| ppa008503m | gi|224076226| | ABN46984.1 | -1.11 | 26.12 | misc.peroxidases | Cationic peroxidase [ *Nelumbo nucifera* ] | 0 |
| ppa009153m | gi|255581482| | XP_004136641.1 | -1.11 | 35.1 | not assigned.no ontology | Haloacid dehalogenase-like hydrolase domain-containing protein 3-like [ *Cucumis sativus* ] | 2.00E-164 |
| ppa007590m | gi|255573416| | NP_189619.2 | -1.11 | 35.1 | not assigned.no ontology | NLI interacting factor (NIF) family protein [ *Arabidopsis thaliana* ] | 8E-124 |
| ppa012886m | gi|2465015| | CAA04770.1 | -1.11 | 20.2.99 | stress.abiotic.unspecified | Ripening-induced protein [ *Fragaria vesca* ] | 3.9 |
| ppa002997m | gi|255547157| | XP_002514636.1 | -1.11 | 29.5.11.4.3.2 | protein.degradation.ubiquitin.E3.SCF.FBOX | Kelch repeat protein, putative [ *Ricinus communis* ] | 2E-139 |
| ppa019228m | gi|225464325| | XP_002324940.1 | -1.11 | 17.4.2 | hormone metabolism.cytokinin.signal transduction | Histidine kinase cytokinin receptor [ *Populus trichocarpa* ] | 0 |
| ppa005656m | gi|225458003| | NP_566054.1 | -1.11 | 29.2.3 | protein.synthesis.initiation | Eukaryotic initiation factor 3 gamma subunit family protein [ *Arabidopsis thaliana* ] | 0 |
| ppa012044m | gi|255581672| | NP_181032.1 | -1.11 | 29.6 | protein.folding | Embryo sac development arrest 3; heat shock protein binding / unfolded protein binding [ *Arabidopsis thaliana* ] | 5E-85 |
| ppa012822m | - | unkown | -1.11 | 35.2 | not assigned.unknown | Unkown | 0 |
| ppa010625m | gi|224119366| | XP_002511388.1 | -1.11 | 26.28 | misc.GDSL-motif lipase | Isoamyl acetate-hydrolyzing esterase, putative [ *Ricinus communis* ] | 0 |
| ppa009062m | gi|225449084| | XP_002533249.1 | -1.11 | 35.1 | not assigned.no ontology | Transmembrane protein, putative [ *Ricinus communis* ] | 2E-93 |
| ppa024251m | - | unkown | -1.11 | 35.2 | not assigned.unknown | Unkown | 0 |
| ppa002052m | gi|224142159| | EOY30747.1 | -1.11 | 11.9.4.9 | lipid metabolism.lipid degradation.beta-oxidation.multifunctional | Enoyl-coa hydratase/isomerase family [ *Theobroma cacao* ] | 0 |
| ppa009086m | gi|224116066| | ACG47470.1 | -1.11 | 35.2 | not assigned.unknown | Plant-specific domain TIGR01615 family protein [ *Zea mays* ] | 2E-166 |
| ppa008669m | gi|255553837| | XP_002874864.1 | -1.11 | 35.2 | not assigned.unknown | Hypothetical protein ARALYDRAFT_911855 [ *Arabidopsis lyrata subsp. Lyrata* ] | 5E-18 |
| ppa009040m | gi|225453454| | XP_002866439.1 | -1.11 | 29.5.2 | protein.degradation.autophagy | Autophagy 3 [ *Arabidopsis lyrata subsp. Lyrata* ] | 1E-34 |
| ppa008635m | gi|255582121| | NP_189539.1 | -1.11 | 17.2.2 | hormone metabolism.auxin.signal transduction | AAR3 (antiauxin-resistant 3) [ *Arabidopsis thaliana* ] | 1E-97 |
| ppa012854m | gi|296086416| | XP_002319717.1 | -1.11 | 29.5.11.3 | protein.degradation.ubiquitin.E2 | Histone ubiquitination proteins group [ *Populus trichocarpa* ] | 0 |
| ppa006950m | gi|224146457| | ABS32230.1 | -1.11 | 33.99 | development.unspecified | Transducin family protein [ *Carica papaya* ] | 0 |
| ppa001113m | gi|14041825| | BAB55002.1 | -1.11 | 12.1.1 | N-metabolism.nitrate metabolism.NR | Nitrate reductase [ *Prunus persica* ] | 0 |
| ppa008391m | gi|225449056| | XP_002311094.1 | -1.11 | 35.1 | not assigned.no ontology | Mtn21-like protein [ *Populus trichocarpa* ] | 0 |
| ppa001643m | gi|225453076| | XP_002531931.1 | -1.11 | 9.1.2 | mitochondrial electron transport / ATP synthesis.NADH-DH.localisation not clear | NADH-ubiquinone oxidoreductase, putative [ *Ricinus communis* ] | 2E-09 |
| ppa012895m | gi|297740141| | ACF06620.1 | -1.12 | 29.2.1.2.1.14 | protein.synthesis.ribosomal protein.eukaryotic.40S subunit.S14 | Ribosomal protein S14 [ *Elaeis guineensis* ] | 3E-53 |
| ppa002769m | gi|225443272| | XP_002525010.1 | -1.12 | 13.1.5.1.1 | amino acid metabolism.synthesis.serine-glycine-cysteine group.serine.phosphoglycerate dehydrogenase | D-3-phosphoglycerate dehydrogenase, putative [ *Ricinus communis* ] | 0 |
| ppa010374m | gi|255637493| | CAD48198.1 | -1.12 | 27.4 | RNA.RNA binding | RNA-binding protein [ *Medicago truncatula* ] | 0 |
| ppa001494m | gi|225432256| | XP_002516928.1 | -1.12 | 31.1 | cell.organisation | Kif4, putative [ *Ricinus communis* ] | 1E-70 |
| ppa008762m | gi|225426619| | YP_721257.1 | -1.12 | 35.2 | not assigned.unknown | Chromosome segregation atpase-like protein [ *Trichodesmium erythraeum IMS101* ] | 0 |
| ppa010223m | gi|297734458| | NP_001147139.1 | -1.12 | 29.2.1.2.2.57 | protein.synthesis.ribosomal protein.eukaryotic.60S subunit.L7A | 60S ribosomal protein L7a [ *Zea mays* ] | 1E-111 |
| ppa002609m | gi|147857536| | XP_002518131.1 | -1.12 | 31.1 | cell.organisation | Protein binding protein, putative [ *Ricinus communis* ] | 2E-61 |
| ppa009427m | gi|225448385| | NP_565921.1 | -1.12 | 35.2 | not assigned.unknown | Protein binding / structural molecule [ *Arabidopsis thaliana* ] | 0 |
| ppa009351m | gi|225434666| | XP_002525600.1 | -1.12 | 29.2.1.1.1.2.4 | protein.synthesis.ribosomal protein.prokaryotic.chloroplast.50S subunit.L4 | 50S ribosomal protein L4, putative [ *Ricinus communis* ] | 0 |
| ppa012831m | gi|225434875| | XP_002283174.1 | -1.12 | 35.2 | not assigned.unknown | Uncharacterized protein LOC100248460 [ *Vitis vinifera* ] | 7.00E-123 |
| ppa007452m | gi|225430200| | XP_002871473.1 | -1.13 | 35.2 | not assigned.unknown | Hypothetical protein ARALYDRAFT_487977 [ *Arabidopsis lyrata subsp. Lyrata* ] | 0 |
| ppa006340m | gi|147817790| | XP_002530130.1 | -1.13 | 29.5.11.3 | protein.degradation.ubiquitin.E2 | Ubiquitin-protein ligase, putative [ *Ricinus communis* ] | 5E-83 |
| ppb011759m | - | unkown | -1.13 | 35.2 | not assigned.unknown | Unkown | 0 |
| ppa011368m | gi|302142732| | NP_001149268.1 | -1.13 | 30.5 | signalling.G-proteins | Ras-related protein Rab-18 [ *Zea mays* ] | 0 |
| ppa012909m | gi|297737057| | XP_002517132.1 | -1.13 | 35.2 | not assigned.unknown | Prefoldin subunit, putative [ *Ricinus communis* ] | 7E-134 |
| ppa007364m | - | unkown | -1.13 | 35.2 | not assigned.unknown | Unkown | 0 |
| ppa003140m | gi|225433251| | XP_001393144.2 | -1.13 | 35.2 | not assigned.unknown | UDP-galactose transporter [ *Aspergillus niger CBS 513.88* ] | 0 |
| ppa010817m | gi|255574145| | XP_002527988.1 | -1.13 | 35.2 | not assigned.unknown | Conserved hypothetical protein [ *Ricinus communis* ] | 5E-143 |
| ppa002262m | gi|225438333| | ABF99782.1 | -1.13 | 35.2 | not assigned.unknown | Streptomyces cyclase/dehydrase family protein, expressed [ *Oryza sativa Japonica Group* ] | 0 |
| ppa001446m | gi|297737338| | BAD07877 | -1.14 | 35.1 | not assigned.no ontology | Putative rela/spot homologous protein RSH2 [ *Oryza sativa Japonica Group* ] | 0 |
| ppa004900m | gi|224111752| | YP_003138087.1 | -1.14 | 35.2 | not assigned.unknown | S-layer domain protein [ *Cyanothece sp. PCC 8802* ] | 2E-132 |
| ppa009477m | gi|225424246| | XP_002526031.1 | -1.14 | 35.2 | not assigned.unknown | Phytanoyl-coa dioxygenase domain containing, putative [ *Ricinus communis* ] | 0 |
| ppa005032m | gi|296082448| | ADM67557.1 | -1.14 | 13.2.6.2 | amino acid metabolism.degradation.aromatic aa.tyrosine | Prephenate aminotransferase [ *Petunia x hybrida* ] | 1E-78 |
| ppa010820m | gi|255631256| | ABA98345.2 | -1.14 | 26.19 | misc.plastocyanin-like | Plastocyanin-like domain containing protein, expressed [ *Oryza sativa Japonica Group* ] | 3.00E-24 |
| ppa008760m | gi|224131524| | XP_002512372.1 | -1.14 | 34.9 | transport.metabolite transporters at the mitochondrial membrane | Peroxisomal membrane protein pmp34, putative [ *Ricinus communis* ] | 0 |
| ppa001728m | gi|255554789| | NP_193943.2 | -1.14 | 20.2.3 | stress.abiotic.drought/salt | Early-responsive to dehydration protein-related / ERD protein-related [ *Arabidopsis thaliana* ] | 9E-162 |
| ppa013397m | gi|224082037| | XP_001766739.1 | -1.14 | 35.2 | not assigned.unknown | Qc-SNARE, plant SFT1-family [ *Physcomitrella patens subsp. Patens* ] | 6E-106 |
| ppa003561m | gi|225451597| | XP_002512848.1 | -1.14 | 13.1.3.6.1.1 | amino acid metabolism.synthesis.aspartate family.misc.homoserine.aspartate kinase | Aspartate kinase, putative [ *Ricinus communis* ] | 6E-158 |
| ppa006688m | gi|219842178| | BAH10646.1 | -1.14 | 13.1.6.3.1 | amino acid metabolism.synthesis.aromatic aa.phenylalanine.arogenate dehydratase / prephenate dehydratase | Prephenate dehydratase [ *Hevea brasiliensis* ] | 3E-122 |
| ppa010844m | gi|255544260| | XP_002513192.1 | -1.14 | 29.2.1.2.2.6 | protein.synthesis.ribosomal protein.eukaryotic.60S subunit.L6 | 60S ribosomal protein L6, putative [ *Ricinus communis* ] | 0 |
| ppa000399m | gi|255570013| | NP_176892.1 | -1.14 | 35.1 | not assigned.no ontology | Protein little nuclei1 [ *Arabidopsis thaliana* ] | 0 |
| ppa004031m | gi|225456536| | XP_002514619.1 | -1.14 | 26.2 | misc.UDP glucosyl and glucoronyl transferases | Glycosyltransferase QUASIMODO1, putative [ *Ricinus communis* ] | 2E-09 |
| ppa006410m | gi|297746445| | CBI16501.3 | -1.14 | 35.2 | not assigned.unknown | Unnamed protein product [ *Vitis vinifera* ] | 7E-45 |
| ppa001866m | gi|224090268| | EOY09319.1 | -1.14 | 30.2.17 | signalling.receptor kinases.DUF 26 | Curculin-like lectin family protein / PAN domain-containing protein, putative [ *Theobroma cacao* ] | 0 |
| ppa003438m | gi|296081121| | XP_002530501.1 | -1.14 | 20.2.1 | stress.abiotic.heat | Heat shock protein binding protein, putative [ *Ricinus communis* ] | 4E-84 |
| ppa003970m | gi|224089605| | XP_002519387.1 | -1.15 | 27.2 | RNA.transcription | Tfiif-alpha, putative [ *Ricinus communis* ] | 0.036 |
| ppa001240m | gi|225468318| | XP_002525278.1 | -1.15 | 35.1.5 | not assigned.no ontology.pentatricopeptide (PPR) repeat-containing protein | GTP binding protein, putative [ *Ricinus communis* ] | 0 |
| ppa000036m | gi|297745300| | ABD32834.1 | -1.15 | 35.2 | not assigned.unknown | 2-oxoacid dehydrogenase, lipoyl-binding site [ *Medicago truncatula* ] | 0 |
| ppa013473m | gi|224116004| | XP_002521955.1 | -1.15 | 35.2 | not assigned.unknown | Associate of C-myc, putative [ *Ricinus communis* ] | 2E-77 |
| ppa004795m | gi|1351410|sp | NP_001236678.1 | -1.15 | 29.3.4.3 | protein.targeting.secretory pathway.vacuole | Vacuolar-processing enzyme precursor [ *Glycine max* ] | 0 |
| ppa012797m | gi|224140311| | XP_002892228.1 | -1.15 | 35.1.5 | not assigned.no ontology.pentatricopeptide (PPR) repeat-containing protein | EMB2748 [ *Arabidopsis lyrata subsp. Lyrata* ] | 0 |
| ppa009193m | gi|255538544| | XP_002510337.1 | -1.15 | 29.5.11.4.2 | protein.degradation.ubiquitin.E3.RING | Rnf5, putative [ *Ricinus communis* ] | 2E-96 |
| ppa011367m | gi|255539463| | XP_002510796.1 | -1.15 | 27.3.67 | RNA.regulation of transcription.putative transcription regulator | Remorin, putative [ *Ricinus communis* ] | 9E-150 |
| ppa005325m | gi|224121128| | XP_001276514.1 | -1.15 | 35.2 | not assigned.unknown | DUF895 domain protein [ *Aspergillus clavatus NRRL 1* ] | 0 |
| ppa001512m | gi|255554176| | XP_002518128.1 | -1.15 | 35.1.1 | not assigned.no ontology.ABC1 family protein | Protein ABC1, mitochondrial precursor, putative [ *Ricinus communis* ] | 1.1 |
| ppa003739m | gi|225449659| | NP_566976.1 | -1.16 | 4.1.14 | glycolysis.cytosolic branch.pyruvate kinase (PK) | Pyruvate kinase, putative [ *Arabidopsis thaliana* ] | 4E-100 |
| ppa008258m | gi|225456177| | XP_002530475.1 | -1.16 | 3.5 | minor CHO metabolism.others | Aldose-1-epimerase, putative [ *Ricinus communis* ] | 1E-73 |
| ppa008785m | gi|302143792| | AAC27745.1 | -1.16 | 26.1 | misc.misc2 | Glutamine cyclotransferase precursor [ *Carica papaya* ] | 1E-88 |
| ppa013075m | gi|225445398| | ABX46148.1 | -1.16 | 35.2 | not assigned.unknown | Blight-associated protein P12 [ *Citrus aurantium* ] | 0 |
| ppa005592m | gi|224124112| | XP_002520072.1 | -1.16 | 34.1.1.6 | transport.p- and v-ATPases.H+-transporting two-sector ATPase.subunit H | Vacuolar ATP synthase subunit h, putative [ *Ricinus communis* ] | 0 |
| ppa009214m | gi|297733795| | XP_002511412.1 | -1.16 | 35.2 | not assigned.unknown | Phosphoglycerate mutase, putative [ *Ricinus communis* ] | 2E-153 |
| ppa006713m | gi|224113265| | ADN34060.1 | -1.16 | 28.1 | DNA.synthesis/chromatin structure | Endonuclease/exonuclease/phosphatase family protein [ *Cucumis melo subsp. Melo* ] | 0 |
| ppa013781m | gi|195617694| | ACG30677.1 | -1.16 | 28.1.3 | DNA.synthesis/chromatin structure.histone | Histone H4 [ *Zea mays* ] | 0 |
| ppa013719m | - | unkown | -1.16 | 35.2 | not assigned.unknown | Unkown | 0 |
| ppa012313m | gi|302143542| | XP_002681944.1 | -1.16 | 35.2 | not assigned.unknown | RWP-RK domain-containing protein [ *Naegleria gruberi* ] | 1E-60 |
| ppa012840m | gi|224085603| | XP_002520996.1 | -1.16 | 29.2.1.2.1.15 | protein.synthesis.ribosomal protein.eukaryotic.40S subunit.S15 | 40S ribosomal protein S15, putative [ *Ricinus communis* ] | 5E-22 |
| ppa002968m | gi|296087585| | ABV89662.1 | -1.16 | 20.2.3 | stress.abiotic.drought/salt | Dehydration-responsive protein-related [ *Brassica rapa* ] | 0 |
| ppa002590m | gi|225446883| | BAB02646.1 | -1.16 | 28.1.1.4 | DNA.synthesis/chromatin structure.retrotransposon/transposase.hat-like transposase | Ac transposase-like protein [ *Arabidopsis thaliana* ] | 5E-131 |
| ppa014064m | gi|255553909| | NP_567802.1 | -1.16 | 35.2 | not assigned.unknown | Wound-responsive protein-related [ *Arabidopsis thaliana* ] | 8E-161 |
| ppa010482m | gi|224097124| | XP_002517389.1 | -1.16 | 31.1 | cell.organisation | Aberrant large forked product, putative   [ *Ricinus communis* ] | 6E-110 |
| ppa007575m | gi|297847914| | XP_002891838.1 | -1.17 | 8.1.1.1 | TCA / org transformation.TCA.pyruvate DH.E1 | Branched-chain alpha-keto acid decarboxylase e1 beta subunit [ *Arabidopsis lyrata subsp. Lyrata* ] | 0 |
| ppa005941m | gi|224053781| | XP_002297976.1 | -1.17 | 34.13 | transport.peptides and oligopeptides | Inner membrane protein [ *Populus trichocarpa* ] | 0 |
| ppa003539m | gi|255563562| | BAD81275.1 | -1.17 | 2.2.2.1.2 | major CHO metabolism.degradation.starch.starch cleavage.beta amylase | Beta-amylase PCT-BMYI,putative [ *Oryza sativa Japonica Group* ] | 9E-10 |
| ppa013321m | gi|255585518| | AAO32066.1 | -1.17 | 35.2 | not assigned.unknown | Erwinia induced protein 2 [ *Solanum tuberosum* ] | 1E-128 |
| ppa012984m | gi|224096251| | XP_002525271.1 | -1.17 | 29.2.1.2.2.26 | protein.synthesis.ribosomal protein.eukaryotic.60S subunit.L26 | 60S ribosomal protein L26, putative [ *Ricinus communis* ] | 5E-129 |
| ppa007072m | gi|225457425| | XP_002284929.1 | -1.17 | 35.2 | not assigned.unknown | Uncharacterized protein LOC100260374 [ *Vitis vinifera* ] | 0 |
| ppa004486m | gi|255556616| | XP_002519342.1 | -1.17 | 29.5.4 | protein.degradation.aspartate protease | Aspartic proteinase oryzasin-1 precursor, putative [ *Ricinus communis* ] | 0 |
| ppa000521m | gi|225448459| | XP_002532114.1 | -1.17 | 35.1 | not assigned.no ontology | Nucleotide binding protein, putative [ *Ricinus communis* ] | 8E-23 |
| ppa004411m | gi|63087742|e | CAI93186.1 | -1.17 | 26.2 | misc.UDP glucosyl and glucoronyl transferases | Glycosyltransferase [ *Gossypium raimondii* ] | 0 |
| ppa003694m | gi|255543829| | XP_002512977.1 | -1.17 | 20.1 | stress.biotic | DNA-damage-inducible protein f, putative [ *Ricinus communis* ] | 2E-47 |
| ppa004905m | gi|255586449| | XP_002533869.1 | -1.17 | 27.3.32 | RNA.regulation of transcription.WRKY domain transcription factor family | WRKY transcription factor, putative [ *Ricinus communis* ] | 1E-148 |
| ppa006279m | gi|225445480| | XP_002873705.1 | -1.17 | 26.2 | misc.UDP glucosyl and glucoronyl transferases | Glycosyltransferase family 14 protein [ *Arabidopsis lyrata subsp. Lyrata* ] | 0 |
| ppa009277m | gi|255554172| | XP_002892240.1 | -1.17 | 33.99 | development.unspecified | Male sterility MS5 family protein [ *Arabidopsis lyrata subsp. Lyrata* ] | 4.00E-133 |
| ppa008513m | gi|225462070| | NP_175972.1 | -1.17 | 35.1.9 | not assigned.no ontology.BTB/POZ domain-containing protein | BTB/POZ domain-containing protein [ *Arabidopsis thaliana* ] | 1E-134 |
| ppb011786m | - | unkown | -1.17 | 35.2 | not assigned.unknown | Unkown | 0 |
| ppa009640m | gi|225459888| | XP_002262923.1 | -1.18 | 35.2 | not assigned.unknown | Adp76805.1 | 0 |
| ppa003231m | gi|225456155| | XP_002530483.1 | -1.18 | 29.3.3 | protein.targeting.chloroplast | Amidase, putative [ *Ricinus communis* ] | 0 |
| ppa007036m | gi|158427472| | ABW38330.1 | -1.18 | 1.3.7 | PS.calvin cycle.FBPase | Chloroplast fructose-1,6-bisphosphatase I [ *Fragaria x ananassa* ] | 6E-15 |
| ppa006291m | gi|255579743| | XP_002530710.1 | -1.18 | 23.1.2.9 | nucleotide metabolism.synthesis.purine.AICAR transformylase | Bifunctional purine biosynthesis protein, putative [ *Ricinus communis* ] | 0 |
| ppa005760m | gi|225453732| | CAY85529.1 | -1.18 | 30.11.1 | signalling.light.COP9 signalosome | Cop11 protein [ *Carica papaya* ] | 0 |
| ppa008271m | gi|225459229| | AAG31326.1 | -1.18 | 29.4 | protein.postranslational modification | Putative serine/threonine kinase gdbrpk [ *Vitis vinifera* ] | 0 |
| ppa012818m | gi|224104365| | XP_002313412.1 | -1.18 | 34.12 | transport.metal | Copper transporter [ *Populus trichocarpa* ] | 0 |
| ppa010221m | gi|224088842| | XP_002308564.1 | -1.18 | 35.2 | not assigned.unknown | Predicted protein [ *Populus trichocarpa* ] | 3E-145 |
| ppa000435m | gi|225463689| | XP_002523758.1 | -1.18 | 31.1 | cell.organisation | Myosin viii, putative [ *Ricinus communis* ] | 0.000002 |
| ppa013045m | gi|224143930| | XP_002325126.1 | -1.18 | 35.2 | not assigned.unknown | Predicted protein [ *Populus trichocarpa* ] | 0 |
| ppa002733m | gi|224073194| | XP_002304017.1 | -1.18 | 25 | C1-metabolism | 10-formyltetrahydrofolate synthetase [ *Populus trichocarpa* ] | 0 |
| ppa000655m | gi|225450013| | XP_002517770.1 | -1.19 | 31.2 | cell.division | Structural maintenance of chromosomes 5 smc5, putative [ *Ricinus communis* ] | 0 |
| ppb010285m | - | unkown | -1.19 | 35.2 | not assigned.unknown | Unkown | 0 |
| ppa007150m | gi|109895092| | XP_002873018.1 | -1.19 | 2.2.2.4 | major CHO metabolism.degradation.starch.D enzyme | Glycoside hydrolase starch-binding domain-containing protein [ *Arabidopsis lyrata subsp. Lyrata* ] | 0 |
| ppa006811m | gi|225461965| | XP_002522786.1 | -1.19 | 27.2 | RNA.transcription | DNA-directed RNA polymerase I/III subunits, putative [ *Ricinus communis* ] | 0 |
| ppa001631m | gi|225450227| | ADO51752.1 | -1.19 | 17.7.1.2 | hormone metabolism.jasmonate.synthesis-degradation.lipoxygenase | Lipoxygenase [ *Camellia sinensis* ] | 0 |
| ppa005028m | gi|225463912| | XP_002266398.1 | -1.19 | 13.2.7 | amino acid metabolism.degradation.histidine | Histidine decarboxylase-like [ *Vitis vinifera* ] | 0 |
| ppa008383m | gi|255568792| | AAA79703.1 | -1.19 | 35.2 | not assigned.unknown | OBP32pep [ *Arabidopsis thaliana* ] | 0 |
| ppa002999m | gi|225457618| | XP_002510712.1 | -1.19 | 29.7 | protein.glycosylation | Ribophorin, putative [ *Ricinus communis* ] | 0 |
| ppa010056m | gi|118486162| | YP_001814674.1 | -1.19 | 35.2 | not assigned.unknown | Acid phosphatase/vanadium-dependent haloperoxidase related [ *Exiguobacterium sibiricum 255-15* ] | 2E-158 |
| ppa005969m | gi|255546149| | XP_002514134.1 | -1.19 | 29.4 | protein.postranslational modification | N-myristoyl transferase, putative [ *Ricinus communis* ] | 3E-176 |
| ppa014175m | gi|6967041|em | CAB72442.1 | -1.19 | 35.2 | not assigned.unknown | Phase-change related protein [ *Quercus robur* ] | 5E-47 |
| ppa004810m | gi|296082200| | NP_566850.3 | -1.19 | 35.1.41 | not assigned.no ontology.hydroxyproline rich proteins | RIK (RS2-Interacting KH protein); RNA binding [ *Arabidopsis thaliana* ] | 0 |
| ppa011833m | gi|255541644| | NP_567171.1 | -1.19 | 29.5.11.4.2 | protein.degradation.ubiquitin.E3.RING | RHB1A; protein binding / zinc ion binding [ *Arabidopsis thaliana* ] | 4E-36 |
| ppa002477m | gi|225438295| | XP_002513274.1 | -1.20 | 30.5 | signalling.G-proteins | GTP-binding protein lepa, putative [ *Ricinus communis* ] | 3E-154 |
| ppa006818m | gi|255644613| | XP_002533664.1 | -1.20 | 34.12 | transport.metal | Cation efflux protein/ zinc transporter, putative [ *Ricinus communis* ] | 2E-84 |
| ppa003902m | gi|255568687| | XP_002525315.1 | -1.20 | 29.4 | protein.postranslational modification | Protein phosphatase 2a, regulatory subunit, putative [ *Ricinus communis* ] | 4E-49 |
| ppa008241m | gi|255584608| | XP_002874475.1 | -1.20 | 29.5.11.1 | protein.degradation.ubiquitin.ubiquitin | Ubiquitin family protein [ *Arabidopsis lyrata subsp. Lyrata* ] | 0.35 |
| ppa009731m | gi|255559653| | XP_002520846.1 | -1.20 | 35.2 | not assigned.unknown | Dual specificity protein phosphatase, putative [ *Ricinus communis* ] | 9E-129 |
| ppa009807m | gi|224088176| | XP_002308356.1 | -1.20 | 35.2 | not assigned.unknown | Predicted protein [ *Populus trichocarpa* ] | 0 |
| ppa001491m | gi|225434891| | AAK77908.1 | -1.20 | 29.5.7 | protein.degradation.metalloprotease | AAA-metalloprotease ftsh [ *Pisum sativum* ] | 0 |
| ppa002002m | gi|224116528| | XP_002331919.1 | -1.20 | 34.13 | transport.peptides and oligopeptides | Oligopeptide transporter OPT family [ *Populus trichocarpa* ] | 0 |
| ppa005980m | gi|225440270| | EOY08776.1 | -1.20 | 35.1 | not assigned.no ontology | FAD-dependent oxidoreductase family protein isoform 1 [ *Theobroma cacao* ] | 0 |
| ppa021958m | gi|225429876| | XP_002280997.2 | -1.20 | 35.2 | not assigned.unknown | Hypothetical protein [ *Vitis vinifera* ] | 0 |
| ppa005901m | gi|224057042| | XP_002299116.1 | -1.20 | 34.3 | transport.amino acids | Neutral amino acid transport protein [ *Populus trichocarpa* ] | 0 |
| ppa012764m | gi|255559018| | XP_002520532.1 | -1.20 | 20.2.1 | stress.abiotic.heat | Chaperone protein dnaj, putative [ *Ricinus communis* ] | 0 |
| ppa005402m | gi|255542366| | XP_002891901.1 | -1.20 | 35.2 | not assigned.unknown | Hypothetical protein ARALYDRAFT_474728 [ *Arabidopsis lyrata subsp. Lyrata* ] | 0 |
| ppa004314m | gi|224070482| | XP_001694960.1 | -1.21 | 35.2 | not assigned.unknown | Chloroplast lumenal protein [ *Chlamydomonas reinhardtii* ] | 1E-95 |
| ppa002585m | gi|225432051| | CAJ87637.1 | -1.21 | 26.3 | misc.gluco-, galacto- and mannosidases | Putative beta-glycosidase [ *Solanum lycopersicum* ] | 3E-66 |
| ppa005271m | gi|225429626| | XP_002521353.1 | -1.21 | 31.1 | cell.organisation | Protein binding protein, putative [ *Ricinus communis* ] | 2E-171 |
| ppa007980m | gi|255542632| | CAN60540.1 | -1.21 | 35.2 | not assigned.unknown | Hypothetical protein VITISV_018288 [ *Vitis vinifera* ] | 1E-174 |
| ppa021687m | gi|224055703| | CAB96664.1 | -1.21 | 35.2 | not assigned.unknown | Putative protein [ *Arabidopsis thaliana* ] | 2E-161 |
| ppa008340m | gi|255639255| | XP_002510771.1 | -1.21 | 20.1 | stress.biotic | Aig1, putative [ *Ricinus communis* ] | 5E-127 |
| ppa006962m | gi|255567082| | XP_002524523.1 | -1.21 | 29.5 | protein.degradation | Gamma-glutamyl hydrolase precursor, putative [ *Ricinus communis* ] | 0 |
| ppa008742m | gi|224089895| | ACE97591.1 | -1.21 | 29.5.11.4.2 | protein.degradation.ubiquitin.E3.RING | Thioredoxin-related protein [ *Populus tremula* ] | 0 |
| ppa001620m | gi|225451827| | ABD33348.2 | -1.21 | 35.2 | not assigned.unknown | IMP dehydrogenase/GMP reductase, putative [ *Medicago truncatula* ] | 7E-112 |
| ppa013166m | gi|296083584| | NP_001078516.1 | -1.21 | 28.1.3.2.3 | DNA.synthesis/chromatin structure.histone.core.H3 | Histone H3.2 [ *Arabidopsis thaliana* ] | 0 |
| ppa013347m | gi|224098596| | AAX47177.2 | -1.21 | 30.11 | signalling.light | Early flowering 4 [ *Pisum sativum* ] | 0 |
| ppa008708m | gi|224129976| | ABY40730.1 | -1.21 | 29.5.11.4.3.2 | protein.degradation.ubiquitin.E3.SCF.FBOX | F-box protein [ *Citrus trifoliata* ] | 2E-119 |
| ppa010183m | gi|224134122| | XP_002869043.1 | -1.21 | 29.2.1.2.2.8 | protein.synthesis.ribosomal protein.eukaryotic.60S subunit.L8 | 60S ribosomal protein L8 [ *Arabidopsis lyrata subsp. Lyrata* ] | 0 |
| ppa013188m | gi|255631870| | AAA86950.1 | -1.22 | 29.2.1.2.2.27 | protein.synthesis.ribosomal protein.eukaryotic.60S subunit.L27 | Ribosomal protein L27 homolog [ *Pisum sativum* ] | 9E-149 |
| ppa006294m | gi|224100353| | XP_002873908.1 | -1.22 | 19.13 | tetrapyrrole synthesis.divinyl chlorophyllide-a 8-vinyl-reductase | Pale-green and chlorophyll B reduced 2 [ *Arabidopsis lyrata subsp. Lyrata* ] | 0 |
| ppa012770m | gi|1684851| | AAB36543.1 | -1.22 | 20.2.1 | stress.abiotic.heat | Dnaj-like protein [ *Phaseolus vulgaris* ] | 0 |
| ppa007431m | gi|224093876| | XP_002532275.1 | -1.22 | 30.5 | signalling.G-proteins | Developmentally regulated GTP-binding protein, putative [ *Ricinus communis* ] | 9E-146 |
| ppa010528m | gi|302144100| | NP_849649.1 | -1.22 | 35.1 | not assigned.no ontology | Alanine racemase family protein [ *Arabidopsis thaliana* ] | 0 |
| ppa004119m | gi|225446940| | XP_002517946.1 | -1.22 | 34.2 | transport.sugars | Sugar transporter, putative [ *Ricinus communis* ] | 0 |
| ppa014171m | - | unkown | -1.22 | 35.2 | not assigned.unknown | Unkown | 0 |
| ppa000826m | gi|225455802| | XP_002511999.1 | -1.22 | 27.1 | RNA.processing | RNA binding protein, putative [ *Ricinus communis* ] | 0 |
| ppa014107m | - | unkown | -1.22 | 35.2 | not assigned.unknown | Unkown | 0 |
| ppa011911m | gi|1843533| | AAB47752.1 | -1.23 | 17.2.2 | hormone metabolism.auxin.signal transduction | Auxin binding protein [ *Malus x domestica* ] | 0 |
| ppa020143m | gi|224106816| | XP_002314295.1 | -1.23 | 35.2 | not assigned.unknown | Predicted protein [ *Populus trichocarpa* ] | 2.00E-113 |
| ppa000569m | gi|255571970| | ABN08575.1 | -1.23 | 35.2 | not assigned.unknown | Esterase/lipase/thioesterase; Lipase, active site [ *Medicago truncatula* ] | 1E-109 |
| ppa004751m | gi|255558654| | XP_002520352.1 | -1.23 | 5.10 | fermentation.aldehyde dehydrogenase | Aldehyde dehydrogenase, putative [ *Ricinus communis* ] | 4E-155 |
| ppa005963m | gi|224087162| | NP_567751.1 | -1.23 | 2.2.1.99 | major CHO metabolism.degradation.sucrose.misc | Sucrase-related [ *Arabidopsis thaliana* ] | 1E-111 |
| ppa005905m | gi|225439840| | AAX37334.1 | -1.23 | 10.1.5 | cell wall.precursor synthesis.UXS | UDP-glucuronic acid decarboxylase 1 [ *Populus tomentosa* ] | 1E-71 |
| ppa001254m | gi|225437573| | XP_002515279.1 | -1.23 | 27.3.65 | RNA.regulation of transcription.Polycomb Group (PcG) | Enhancer of zeste, ezh, putative [ *Ricinus communis* ] | 2E-12 |
| ppa001456m | gi|255564739| | XP_002523364.1 | -1.23 | 27.1.2 | RNA.processing.RNA helicase | Dead box ATP-dependent RNA helicase, putative [ *Ricinus communis* ] | 2.6 |
| ppa000535m | gi|255566265| | XP_002524120.1 | -1.23 | 29.5 | protein.degradation | Ubiquitin carboxyl-terminal hydrolase, putative [ *Ricinus communis* ] | 0 |
| ppa011822m | gi|224142377| | ZP_05877948.1 | -1.23 | 35.2 | not assigned.unknown | DUF149 domain-containing protein [ *Vibrio furnissii CIP 102972* ] | 3E-89 |
| ppa002510m | gi|224138652| | XP_002525341.1 | -1.23 | 11.9.4.2 | lipid metabolism.lipid degradation.beta-oxidation.acyl CoA DH | Acyl-coa oxidase, putative [ *Ricinus communis* ] | 0 |
| ppa001391m | gi|255539777| | NP_199956.1 | -1.23 | 35.2 | not assigned.unknown | EYE (EMBRYO YELLOW) [ *Arabidopsis thaliana* ] | 5E-66 |
| ppa005904m | gi|225448699| | NP_196142.3 | -1.23 | 29.5.11.3 | protein.degradation.ubiquitin.E2 | Ubiquitin-protein ligase [ *Arabidopsis thaliana* ] | 9E-74 |
| ppa022268m | gi|2499967| | NP_565906.1 | -1.23 | 1.1.1.2 | PS.lightreaction.photosystem II.PSII polypeptide subunits | PPL2 (psbp-like protein 2); calcium ion binding [ *Arabidopsis thaliana* ] | 8E-70 |
| ppa006941m | gi|296081197| | XP_002524302.1 | -1.23 | 29.2.1.2.2.3 | protein.synthesis.ribosomal protein.eukaryotic.60S subunit.L3 | 60S ribosomal protein L3, putative [ *Ricinus communis* ] | 1E-151 |
| ppa010506m | gi|118488485| | ACV41080.1 | -1.24 | 10.1.21 | cell wall.precursor synthesis.phosphomannomutase | Phosphomannomutase D2 [ *Triticum aestivum* ] | 9E-88 |
| ppa010557m | gi|224091859| | NP_973522.1 | -1.24 | 35.1 | not assigned.no ontology | Metal-dependent phosphohydrolase HD domain-containing protein [ *Arabidopsis thaliana* ] | 3E-23 |
| ppa023050m | gi|296082548| | BAC79194.1 | -1.24 | 27.3.67 | RNA.regulation of transcription.putative transcription regulator | Chloroplast nucleoid DNA-binding protein -like protein [ *Oryza sativa Japonica Group* ] | 8E-148 |
| ppa005632m | gi|225425204| | XP_002514875.1 | -1.24 | 35.1 | not assigned.no ontology | Amino acid binding protein, putative [ *Ricinus communis* ] | 0 |
| ppa005538m | gi|255565307| | XP_002523645.1 | -1.24 | 35.2 | not assigned.unknown | Catalytic, putative [ *Ricinus communis* ] | 0 |
| ppa010740m | gi|225458283| | 2KK1_A | -1.24 | 35.2 | not assigned.unknown | Hypothetical protein [ *Vitis vinifera* ] | 5E-175 |
| ppa007520m | gi|225448631| | XP_002520841.1 | -1.24 | 35.1 | not assigned.no ontology | 2-deoxyglucose-6-phosphate phosphatase, putative [ *Ricinus communis* ] | 0 |
| ppa001897m | gi|225432189| | BAB10320.1 | -1.24 | 27.3.99 | RNA.regulation of transcription.unclassified | Mutator-like transposase-like protein [ *Arabidopsis thaliana* ] | 3E-100 |
| ppa006270m | gi|225459591| | AAS55852.1 | -1.24 | 21.2.1 | redox.ascorbate and glutathione.ascorbate | Chloroplast thylakoid-bound ascorbate peroxidase [ *Vigna unguiculata* ] | 8E-157 |
| ppa009189m | gi|255641128| | NP_191155.1 | -1.24 | 29.3.99 | protein.targeting.unknown | NLI interacting factor (NIF) family protein [ *Arabidopsis thaliana* ] | 0.46 |
| ppa002451m | gi|296089085| | NP_563641.1 | -1.24 | 27.3.99 | RNA.regulation of transcription.unclassified | Zinc finger protein-related [ *Arabidopsis thaliana* ] | 0 |
| ppa001744m | gi|224131914| | XP_002524657.1 | -1.24 | 3.1.2.1 | minor CHO metabolism.raffinose family.raffinose synthases.known | Stachyose synthase precursor, putative [ *Ricinus communis* ] | 7E-81 |
| ppa007086m | gi|224117100| | XP_002529518.1 | -1.24 | 3.5 | minor CHO metabolism.others | Ribokinase, putative [ *Ricinus communis* ] | 1.4 |
| ppa004229m | gi|255558490| | XP_002520270.1 | -1.24 | 31.4 | cell.vesicle transport | Protein transporter, putative [ *Ricinus communis* ] | 0 |
| ppa006586m | gi|225464121| | XP_002524982.1 | -1.24 | 27.4 | RNA.RNA binding | RNA binding protein, putative [ *Ricinus communis* ] | 4E-101 |
| ppa012058m | gi|77744891| | ABB02399.1 | -1.25 | 34.99 | transport.misc | Temperature-induced lipocalin [ *Prunus persica* ] | 2E-112 |
| ppa007639m | gi|224100879| | XP_002525195.1 | -1.25 | 27.3.99 | RNA.regulation of transcription.unclassified | Zinc finger protein, putative [ *Ricinus communis* ] | 0 |
| ppa014192m | gi|224134172| | XP_002321754.1 | -1.25 | 35.2 | not assigned.unknown | Xp_002265886.1 | 4.00E-46 |
| ppa006440m | gi|225451915| | XP_002282805.1 | -1.25 | 35.1 | not assigned.no ontology | Uncharacterized protein LOC100244706 [ *Vitis vinifera* ] | 0 |
| ppb020871m | gi|71738563| | AAW58109.2 | -1.25 | 35.2 | not assigned.unknown | Polyphenol oxidase [ *Prunus salicina var. Cordata* ] | 0 |
| ppa013553m | gi|6967041| | CAB72442.1 | -1.25 | 35.2 | not assigned.unknown | Phase-change related protein [ *Quercus robur* ] | 2E-90 |
| ppa010267m | gi|225442349| | XP_002515883.1 | -1.25 | 35.2 | not assigned.unknown | Rwd domain-containing protein, putative [ *Ricinus communis* ] | 0 |
| ppa009493m | gi|224105311| | XP_002512985.1 | -1.25 | 27.3.9 | RNA.regulation of transcription.C2C2(Zn) GATA transcription factor family | GATA transcription factor, putative [ *Ricinus communis* ] | 0 |
| ppa012949m | gi|255630218| | AAD25354.1 | -1.25 | 33.2 | development.late embryogenesis abundant | Seed maturation protein PM22 [ *Glycine max* ] | 0.00002 |
| ppa006263m | gi|255569189| | NP_566423.1 | -1.25 | 35.1 | not assigned.no ontology | ELC; ubiquitin binding [ *Arabidopsis thaliana* ] | 9E-125 |
| ppa008839m | gi|224075888| | EOY19581.1 | -1.26 | 27.3.16 | RNA.regulation of transcription.CCAAT box binding factor family, HAP5 | Nuclear factor Y isoform 1 [ *Theobroma cacao* ] | 1.00E-119 |
| ppa002782m | gi|225450498| | XP_002512557.1 | -1.26 | 7.1.1 | OPP.oxidative PP.G6PD | Glucose-6-phosphate 1-dehydrogenase, putative [ *Ricinus communis* ] | 2E-69 |
| ppa008045m | gi|262192723| | XP_002519451.1 | -1.26 | 35.1.41 | not assigned.no ontology.hydroxyproline rich proteins | Splicing factor 3A subunit, putative [ *Ricinus communis* ] | 3E-36 |
| ppa013804m | gi|15241477| | XP_643471.1 | -1.26 | 35.2 | not assigned.unknown | UPF0139 membrane protein [ *Dictyostelium discoideum AX4* ] | 0 |
| ppa025624m | gi|224108906| | XP_002406019.1 | -1.26 | 35.1 | not assigned.no ontology | Membrane protein, putative [ *Ixodes scapularis* ] | 8E-74 |
| ppa003532m | gi|297744222| | AAC16751.1 | -1.26 | 35.2 | not assigned.unknown | Contains similarity to pre-mrna processing protein PRP39 gb|L29224 from S. Cerevisiae. Ests gb|R64908 and gb|T88158, gb|N38703 and gb|AA651043 come from this gene [ *Arabidopsis thaliana* ] | 3E-70 |
| ppa003981m | gi|225431952| | XP_002519222.1 | -1.26 | 27.1 | RNA.processing | Pre-mrna splicing factor, putative [ *Ricinus communis* ] | 4E-104 |
| ppa006619m | gi|225464928| | XP_002520905.1 | -1.26 | 27.1.2 | RNA.processing.RNA helicase | Dead box ATP-dependent RNA helicase, putative [ *Ricinus communis* ] | 0 |
| ppa012517m | gi|255576633| | EOY11824.1 | -1.26 | 25 | C1-metabolism | Ribonuclease E inhibitor rraa/Dimethylmenaquinone methyltransferase [ *Theobroma cacao* ] | 1.00E-100 |
| ppa004494m | gi|255571031| | ZP_08065655.1 | -1.26 | 35.2 | not assigned.unknown | Carbamoylphosphate synthase large subunit [ *Streptococcus peroris ATCC 700780* ] | 0 |
| ppa011489m | gi|225435768| | CAN78878.1 | -1.27 | 35.2 | not assigned.unknown | Hypothetical protein VITISV_024989 [ *Vitis vinifera* ] | 2E-93 |
| ppa013105m | gi|225435040| | XP_002299887.1 | -1.27 | 31.1 | cell.organisation | Actin depolymerizing factor 1 [ *Populus trichocarpa* ] | 2E-174 |
| ppa004730m | gi|224108786| | NP_568662.1 | -1.27 | 35.1 | not assigned.no ontology | O-acetyltransferase-related [ *Arabidopsis thaliana* ] | 1E-151 |
| ppa012193m | gi|29423617| | AAO73433.1 | -1.27 | 34.1.1.2 | transport.p- and v-ATPases.H+-transporting two-sector ATPase.subunit C | Vacuolar membrane atpase subunit c'' [ *Citrus limon* ] | 2E-85 |
| ppa012790m | gi|255628767| | XP_002516834.1 | -1.27 | 30.1 | signalling.phosphorelay | Histidine-containing phosphotransfer protein, putative [ *Ricinus communis* ] | 0 |
| ppa012145m | gi|224108583| | NP_177040.2 | -1.27 | 27.3.67 | RNA.regulation of transcription.putative transcription regulator | Zinc finger (DNL type) family protein [ *Arabidopsis thaliana* ] | 2E-115 |
| ppa018961m | gi|225443738| | XP_002867974.1 | -1.27 | 30.11 | signalling.light | Transcriptional repressor [ *Arabidopsis lyrata subsp. Lyrata* ] | 3E-172 |
| ppa004609m | gi|225434512| | NP_180571.2 | -1.27 | 29.5.11.1 | protein.degradation.ubiquitin.ubiquitin | Ubiquitin family protein [ *Arabidopsis thaliana* ] | 0 |
| ppa012923m | gi|6996562| | BAA07667.1 | -1.27 | 1.1.2.2 | PS.lightreaction.photosystem I.PSI polypeptide subunits | PSI-E subunit of photosystem I [ *Nicotiana sylvestris* ] | 2E-87 |
| ppa012956m | gi|255632828| | ACU16767.1 | -1.27 | 35.2 | not assigned.unknown | Unknown [ *Glycine max* ] | 6E-75 |
| ppa003472m | gi|147826923| | XP_002327271.1 | -1.27 | 34.13 | transport.peptides and oligopeptides | Proton-dependent oligopeptide transporter [ *Populus trichocarpa* ] | 4E-70 |
| ppa007228m | gi|224088196| | XP_002308365.1 | -1.28 | 31.1 | cell.organisation | Actin 3 [ *Populus trichocarpa* ] | 0 |
| ppa021565m | gi|225451445| | XP_002269795.1 | -1.28 | 29.3.2 | protein.targeting.mitochondria | Mitochondrial import receptor subunit TOM20 [ *Vitis vinifera* ] | 1.00E-147 |
| ppa012440m | gi|224138684| | CAB10530.1 | -1.28 | 35.2 | not assigned.unknown | EREBP-4 like protein [ *Arabidopsis thaliana* ] | 8E-32 |
| ppa006845m | gi|255568816| | XP_002525379.1 | -1.28 | 26.7 | misc.oxidases - copper, flavone etc | Alcohol dehydrogenase, putative [ *Ricinus communis* ] | 0 |
| ppa012622m | gi|302143840| | XP_002514388.1 | -1.28 | 31.4 | cell.vesicle transport | Clathrin coat assembly protein ap19, putative [ *Ricinus communis* ] | 0.48 |
| ppa001553m | gi|225463815| | XP_002268907.2 | -1.28 | 35.1 | not assigned.no ontology | Hypothetical protein [ *Vitis vinifera* ] | 0 |
| ppa018717m | gi|224115778| | XP_002317122.1 | -1.28 | 20.1 | stress.biotic | Nbs-lrr resistance protein [ *Populus trichocarpa* ] | 3E-125 |
| ppa017932m | gi|225424462| | XP_002527551.1 | -1.28 | 35.1 | not assigned.no ontology | Oxidoreductase, putative [ *Ricinus communis* ] | 0 |
| ppa010103m | gi|255576192| | NP_974761.1 | -1.28 | 27.2 | RNA.transcription | Rnase L inhibitor protein-related [ *Arabidopsis thaliana* ] | 0 |
| ppa008190m | gi|297745270| | CBI40350.3 | -1.28 | 35.2 | not assigned.unknown | Unnamed protein product [ *Vitis vinifera* ] | 0 |
| ppa005753m | gi|255559599| | AAW83327.1 | -1.28 | 20 | stress | Cys-rich domain protein [ *Citrus trifoliata* ] | 0 |
| ppa001568m | gi|225441513| | ABB47548.2 | -1.28 | 35.2 | not assigned.unknown | Colon cancer-associated protein Mic1-like containing protein, expressed [ *Oryza sativa Japonica Group* ] | 0 |
| ppa002375m | gi|224063235| | ABX80391.1 | -1.29 | 30.11 | signalling.light | Cryptochrome 1 [ *Vitis riparia* ] | 1E-49 |
| ppa000355m | gi|224061172| | XP_002300362.1 | -1.29 | 34.16 | transport.ABC transporters and multidrug resistance systems | Multidrug resistance protein ABC transporter family [ *Populus trichocarpa* ] | 0 |
| ppa002756m | gi|297737715| | XP_002526006.1 | -1.29 | 29.5.11.4.2 | protein.degradation.ubiquitin.E3.RING | E3 ubiquitin ligase PUB14, putative [ *Ricinus communis* ] | 2E-81 |
| ppa003395m | gi|297737720| | CBI26921.3 | -1.29 | 35.1 | not assigned.no ontology | Unnamed protein product [ *Vitis vinifera* ] | 0 |
| ppa005775m | gi|209362272| | CAD70621.1 | -1.29 | 27.1 | RNA.processing | Polypyrimidine track-binding protein homologue [ *Cicer arietinum* ] | 0 |
| ppa008846m | gi|166203228| | ABY84654.1 | -1.29 | 27.3.32 | RNA.regulation of transcription.WRKY domain transcription factor family | Transcription factor [ *Glycine max* ] | 0 |
| ppa008287m | gi|255560888| | EOY02486.1 | -1.29 | 33.99 | development.unspecified | Transducin/WD40 repeat-like superfamily protein [ *Theobroma cacao* ] | 0 |
| ppa001658m | gi|225443237| | NP_565218.1 | -1.30 | 35.2 | not assigned.unknown | Atnudt3 (Arabidopsis thaliana Nudix hydrolase homolog 3); hydrolase [ ] | 0 |
| ppa008647m | gi|224089993| | NP_001148712.1 | -1.30 | 35.2 | not assigned.unknown | Plant-specific domain TIGR01589 family protein [ *Zea mays* ] | 1E-09 |
| ppa006438m | gi|225432062| | XP_002280604.1 | -1.30 | 29.2.1.1.3.1.1 | protein.synthesis.ribosomal protein.prokaryotic.unknown organellar.30S subunit.S1 | 30S ribosomal protein S1, chloroplastic [ *Vitis vinifera* ] | 0 |
| ppa027045m | gi|296088883| | CBI38427.3 | -1.30 | 35.2 | not assigned.unknown | Unnamed protein product [ *Vitis vinifera* ] | 0 |
| ppa021489m | gi|242038951| | NP_001150828.1 | -1.30 | 13.2.3.5.1 | mino acid metabolism.degradation.aspartate family.lysine.lysine decarboxylase | Decarboxylase family protein [ *Zea mays* ] | 2E-176 |
| ppa007948m | gi|225469490| | NP_566950.1 | -1.30 | 27.3.99 | RNA.regulation of transcription.unclassified | Zinc finger (DHHC type) family protein [ *Arabidopsis thaliana* ] | 1E-136 |
| ppa010124m | gi|255581357| | XP_002531488.1 | -1.30 | 27.4 | RNA.RNA binding | Serine/arginine rich splicing factor, putative [ *Ricinus communis* ] | 0 |
| ppa011925m | gi|225449088| | XP_002533247.1 | -1.31 | 29.5.11.3 | protein.degradation.ubiquitin.E2 | Ubiquitin-conjugating enzyme E2 C, putative [ *Ricinus communis* ] | 3E-57 |
| ppa005739m | gi|225455555| | XP_002510911.1 | -1.31 | 4.1.13 | glycolysis.cytosolic branch.enolase | Enolase, putative [ *Ricinus communis* ] | 5E-59 |
| ppa007497m | gi|296085488| | XP_002519903.1 | -1.31 | 28.1 | DNA.synthesis/chromatin structure | RNA exonuclease NGL1, putative [ *Ricinus communis* ] | 0 |
| ppa005012m | gi|224053983| | ABF98085.1 | -1.31 | 35.1 | not assigned.no ontology | Ranbp1 domain containing protein, expressed [ *Oryza sativa Japonica Group* ] | 1.00E-87 |
| ppa014215m | gi|225452851| | XP_002283710.1 | -1.31 | 35.2 | not assigned.unknown | Hypothetical protein [ *Vitis vinifera* ] | 0 |
| ppa009348m | gi|225432037| | ABA98957.2 | -1.31 | 29.2.1.1.3.1.6 | protein.synthesis.ribosomal protein.prokaryotic.unknown organellar.30S subunit.S6 | Ribosomal protein S6 containing protein, expressed [ *Oryza sativa Japonica Group* ] | 2E-138 |
| ppa006501m | gi|297737199| | BAD90706.1 | -1.31 | 35.2 | not assigned.unknown | Plastid DNA-binding protein [ *Brassica napus* ] | 3E-168 |
| ppa022316m | gi|224086952| | XP_002308016.1 | -1.31 | 35.2 | not assigned.unknown | Predicted protein [ *Populus trichocarpa* ] | 9.00E-98 |
| ppa011181m | gi|225429185| | XP_002275810.1 | -1.31 | 35.2 | not assigned.unknown | Uncharacterized protein LOC100266884 [ *Vitis vinifera* ] | 1.00E-154 |
| ppa014159m | gi|255630067| | XP_002511645.1 | -1.31 | 29.3.4.99 | protein.targeting.secretory pathway.unspecified | Protein transport protein Sec61 subunit beta, putative [ *Ricinus communis* ] | 2E-110 |
| ppa004295m | gi|147866320| | XP_002867493.1 | -1.31 | 3.5 | minor CHO metabolism.others | Pfkb-type carbohydrate kinase family protein [ *Arabidopsis lyrata subsp. Lyrata* ] | 0.57 |
| ppa004595m | gi|118488127| | XP_002881079.1 | -1.31 | 9.2.1.4 | mitochondrial electron transport / ATP synthesis.NADH-DH.type II.internal matrix | NDA2 H dehydrogenase 2 [ *Arabidopsis lyrata subsp. Lyrata* ] | 2E-143 |
| ppa007830m | gi|267881840| | ACY82515.1 | -1.31 | 29.4 | protein.postranslational modification | Mitogen-activated protein kinase kinase [ *Malus x domestica* ] | 1E-54 |
| ppa001624m | gi|255580677| | XP_002531161.1 | -1.32 | 29.3.4.3 | protein.targeting.secretory pathway.vacuole | Vacuolar sorting protein, putative [ *Ricinus communis* ] | 0.02 |
| ppa011575m | gi|297737668| | CBI26869.3 | -1.32 | 35.2 | not assigned.unknown | Unnamed protein product [ *Vitis vinifera* ] | 2.00E-159 |
| ppa013064m | gi|255539324| | CAB75430.1 | -1.32 | 1.1.2.2 | PS.lightreaction.photosystem I.PSI polypeptide subunits | 16kDa membrane protein,putative [ *Nicotiana tabacum* ] | 0 |
| ppa010070m | gi|225427890| | XP_002529258.1 | -1.32 | 13.1.3.4 | amino acid metabolism.synthesis.aspartate family.methionine | Mta/sah nucleosidase, putative [ *Ricinus communis* ] | 0 |
| ppa003687m | gi|255572789| | XP_002527327.1 | -1.32 | 35.1 | not assigned.no ontology | F-box/LRR-repeat protein, putative [ *Ricinus communis* ] | 0 |
| ppa016382m | gi|255545040| | XP_002513581.1 | -1.32 | 29.5.7 | protein.degradation.metalloprotease | ATP-dependent peptidase, putative [ *Ricinus communis* ] | 0 |
| ppa006561m | gi|225425660| | XP_002522449.1 | -1.32 | 27.3.20 | RNA.regulation of transcription.G2-like transcription factor family, GARP | Transcription factor, putative [ *Ricinus communis* ] | 6E-84 |
| ppa001679m | gi|297741318| | XP_002521838.1 | -1.32 | 35.1.5 | not assigned.no ontology.pentatricopeptide (PPR) repeat-containing protein | Pentatricopeptide repeat-containing protein, putative [ *Ricinus communis* ] | 1E-08 |
| ppa000464m | gi|297742796| | EAW81422.1 | -1.32 | 35.2 | not assigned.unknown | Chromosome 14 open reading frame 102, isoform CRA_a [ *Homo sapiens* ] | 3.4 |
| ppa003873m | gi|255542542| | XP_002512334.1 | -1.32 | 34.14 | transport.unspecified cations | Sodium/hydrogen exchanger, putative [ *Ricinus communis* ] | 5E-96 |
| ppa006282m | gi|225426594| | XP_002280182.1 | -1.32 | 33.2 | development.late embryogenesis abundant | Embryogenesis-associated protein EMB8 [ *Vitis vinifera* ] | 0 |
| ppa003121m | gi|225424297| | NP_001090439.1 | -1.32 | 35.2 | not assigned.unknown | Transmembrane protein 214-A [ *Xenopus laevis* ] | 0 |
| ppa008294m | gi|167858183| | ACA04032.1 | -1.32 | 3.1.1.2 | minor CHO metabolism.raffinose family.galactinol synthases.putative | Galactinol synthase 3 [ *Populus trichocarpa* ] | 0 |
| ppa008131m | gi|225429492| | XP_002278242.1 | -1.33 | 35.2 | not assigned.unknown | Uncharacterized protein LOC100243991 [ *Vitis vinifera* ] | 0 |
| ppa009499m | gi|297737287| | XP_002270810.2 | -1.33 | 35.2 | not assigned.unknown | Unnamed protein product [ *Vitis vinifera* ] | 0 |
| ppa005346m | gi|297734068| | XP_002514643.1 | -1.33 | 29.3.1 | protein.targeting.nucleus | RNA binding protein, putative [ *Ricinus communis* ] | 5E-76 |
| ppa003448m | gi|297740127| | XP_002530328.1 | -1.33 | 29.3.3 | protein.targeting.chloroplast | Signal recognition particle protein, putative [ *Ricinus communis* ] | 2E-94 |
| ppa011724m | gi|224091686| | XP_002523504.1 | -1.33 | 31.4 | cell.vesicle transport | Snare protein ykt6, putative [ *Ricinus communis* ] | 9.00E-139 |
| ppa001255m | gi|225441407| | NP_196299.1 | -1.33 | 27.4 | RNA.RNA binding | Mitochondrial transcription termination factor-related / mterf-related [ *Arabidopsis thaliana* ] | 4E-38 |
| ppa009109m | gi|255547472| | XP_003539104.1 | -1.33 | 3.99 | minor CHO metabolism.misc | 4-nitrophenylphosphatase, putative [ *Ricinus communis* ] | 0 |
| ppa018057m | gi|225441517| | XP_002531176.1 | -1.33 | 27.3.11 | RNA.regulation of transcription.C2H2 zinc finger family | Nucleic acid binding protein, putative [ *Ricinus communis* ] | 0 |
| ppa004077m | gi|225456898| | XP_002511324.1 | -1.33 | 35.1 | not assigned.no ontology | Leucine-rich repeat-containing protein, putative [ *Ricinus communis* ] | 8E-143 |
| ppa004540m | gi|297734081| | XP_002514631.1 | -1.33 | 35.1.5 | not assigned.no ontology.pentatricopeptide (PPR) repeat-containing protein | Pentatricopeptide repeat-containing protein, putative [ *Ricinus communis* ] | 2E-150 |
| ppa012623m | gi|225462677| | YP_787663.1 | -1.33 | 35.2 | not assigned.unknown | Phosphatase [ *Bordetella avium 197N* ] | 0.0003 |
| ppa002721m | gi|225424995| | XP_002267025.1 | -1.33 | 29.5.11 | protein.degradation.ubiquitin | Probable phosphatidylinositol 4-kinase type 2-beta At1g26270 isoform 1 [ *Vitis vinifera* ] | 0 |
| ppa004717m | gi|255560451| | XP_002521240.1 | -1.34 | 35.1 | not assigned.no ontology | WD-repeat protein, putative [ *Ricinus communis* ] | 3E-121 |
| ppa012977m | gi|255583289| | XP_002532408.1 | -1.34 | 35.1 | not assigned.unknown | Transferase, transferring glycosyl groups, putative [ *Ricinus communis* ] | 2E-111 |
| ppa011172m | gi|224125036| | XP_002329875.1 | -1.34 | 35.2 | not assigned.unknown | Predicted protein [ *Populus trichocarpa* ] | 2.00E-159 |
| ppa012624m | gi|224071405| | NP_001154457.1 | -1.34 | 35.2 | not assigned.unknown | Vacuolar sorting signal binding [ *Arabidopsis thaliana* ] | 0 |
| ppa006292m | gi|225447779| | XP_002527936.1 | -1.34 | 35.1 | not assigned.no ontology | Pinn, putative [ *Ricinus communis* ] | 0 |
| ppa001799m | gi|259090632| | ACV91932.1 | -1.34 | 11.1.1.2.1 | lipid metabolism.FA synthesis and FA elongation.Acetyl CoA Carboxylation.heteromeric Complex.alpha Carboxyltransferase | Alpha-carboxyltransferase subunit [ *Jatropha curcas* ] | 4E-168 |
| ppa007813m | gi|297744389| | XP_002529248.1 | -1.34 | 1.3.5 | PS.calvin cycle.TPI | Triosephosphate isomerase, putative [ *Ricinus communis* ] | 0 |
| ppa000990m | gi|225436053| | ACZ04920.1 | -1.34 | 27.3.36 | RNA.regulation of transcription.Argonaute | Argonaute 4-like protein [ *Pelargonium x hortorum* ] | 0 |
| ppa006891m | gi|224090529| | XP_002888947.1 | -1.34 | 35.2 | not assigned.unknown | DNA binding protein [ *Arabidopsis lyrata subsp. Lyrata* ] | 0 |
| ppa018107m | gi|147856780| | XP_002527325.1 | -1.35 | 29.4.1.57 | protein.postranslational modification.kinase.receptor like cytoplasmatic kinase VII | Serine/threonine-protein kinase PBS1, putative [ *Ricinus communis* ] | 2E-77 |
| ppa000792m | gi|225432161| | XP_002519316.1 | -1.35 | 33.3 | development.squamosa promoter binding like (SPL) | Squamosa promoter-binding protein, putative [ *Ricinus communis* ] | 0 |
| ppa008445m | gi|224124018| | AAK83083.1 | -1.35 | 35.2 | not assigned.unknown | Nuclear matrix protein 1 [ *Solanum lycopersicum* ] | 0 |
| ppa007597m | gi|225453297| | XP_002528475.1 | -1.35 | 17.5.1 | hormone metabolism.ethylene.synthesis-degradation | Leucoanthocyanidin dioxygenase, putative [ *Ricinus communis* ] | 1E-143 |
| ppa001580m | gi|255557871| | EOY02910.1 | -1.35 | 20.2.3 | stress.abiotic.drought/salt | Auxin signaling F-box 2 [ *Theobroma cacao* ] | 0 |
| ppa011611m | gi|217071188| | ADN34124.1 | -1.35 | 35.1 | not assigned.no ontology | Serine-rich protein [ *Cucumis melo subsp. Melo* ] | 5E-177 |
| ppa008596m | gi|297735012| | XP_002521657.1 | -1.35 | 27.3.6 | RNA.regulation of transcription.bHLH,Basic Helix-Loop-Helix family | DNA binding protein, putative [ *Ricinus communis* ] | 6E-119 |
| ppa009000m | gi|224076000| | ACO57639.1 | -1.35 | 29.4 | protein.postranslational modification | Protein phosphatase 2A catalytic subunit [ *Castanea mollissima* ] | 5E-166 |
| ppa007988m | gi|225426098| | XP_002509884.1 | -1.35 | 29.5 | protein.degradation | Prenyl-dependent CAAX protease, putative [ *Ricinus communis* ] | 6E-82 |
| ppa025450m | gi|224058951| | XP_002523752.1 | -1.36 | 29.5 | protein.degradation | Lysosomal Pro-X carboxypeptidase, putative [ *Ricinus communis* ] | 4E-64 |
| ppa002821m | gi|225435812| | EOY31649.1 | -1.36 | 30.3 | signalling.calcium | Calmodulin-binding protein isoform 1 [ *Theobroma cacao* ] | 3E-117 |
| ppa005403m | gi|225426218| | XP_002518877.1 | -1.36 | 35.2 | not assigned.unknown | Cytochrome C oxidase assembly protein cox11, putative [ *Ricinus communis* ] | 0 |
| ppa013035m | gi|296084062| | XP_002884077.1 | -1.36 | 35.2 | not assigned.unknown | Protein-methionine-s-oxide reductase [ *Arabidopsis lyrata subsp. Lyrata* ] | 2E-71 |
| ppa005189m | gi|224102601| | NP_193159.3 | -1.36 | 35.2 | not assigned.unknown | CBS domain-containing protein-related [ *Arabidopsis thaliana* ] | 0 |
| ppa012000m | gi|225460977| | EOY12356.1 | -1.36 | 27.1.19 | RNA.processing.ribonucleases | Endoribonuclease L-PSP family protein [ *Theobroma cacao* ] | 2.00E-93 |
| ppa001097m | gi|225461122| | EOY12604.1 | -1.36 | 29.5.2 | protein.degradation.autophagy | Autophagy 18 F isoform 1 [ *Theobroma cacao* ] | 0 |
| ppa007489m | gi|296087301| | NP_198667.1 | -1.36 | 29.5.5 | protein.degradation.serine protease | Rhomboid family protein [ *Arabidopsis thaliana* ] | 4E-86 |
| ppa013510m | gi|225638002| | AGG19165.1 | -1.36 | 33.99 | development.unspecified | Auxin-repressed protein [ *Pyrus pyrifolia* ] | 2.00E-76 |
| ppa003623m | gi|225434706| | NP_200704.2 | -1.36 | 35.2 | not assigned.unknown | GIL1 (GRAVITROPIC IN THE LIGHT) [ *Arabidopsis thaliana* ] | 0 |
| ppa003831m | gi|17066577| | AAA91166.1 | -1.36 | 26.3 | misc.gluco-, galacto- and mannosidases | Beta-glucosidase, partial [ *Prunus avium* ] | 0 |
| ppa020372m | gi|255627231| | XP_002522234.1 | -1.36 | 29.2.1.2.2.11 | protein.synthesis.ribosomal protein.eukaryotic.60S subunit.L11 | 60S ribosomal protein L11, putative [ *Ricinus communis* ] | 5E-178 |
| ppa005829m | gi|225428454| | ACN21632.1 | -1.37 | 27.3.6 | RNA.regulation of transcription.bHLH,Basic Helix-Loop-Helix family | Putative basic helix-loop-helix protein BHLH7 [ *Lotus japonicus* ] | 0 |
| ppa007837m | gi|302143498| | EMJ16811.1 | -1.37 | 35.1 | not assigned.no ontology | Unnamed protein product [ *Vitis vinifera* ] | 0 |
| ppa008221m | gi|255546463| | XP_002514291.1 | -1.37 | 29.3.3 | protein.targeting.chloroplast | Signal peptidase I, putative [ *Ricinus communis* ] | 7E-100 |
| ppa004744m | gi|255575340| | EOY12877.1 | -1.37 | 31.2 | cell.division | Regulator of chromosome condensation (RCC1) family protein isoform 1 [ *Theobroma cacao* ] | 0 |
| ppa000137m | gi|225432140| | NP_974804.4 | -1.37 | 35.1 | not assigned.no ontology | ATP binding / protein kinase/ protein serine/threonine kinase/ protein tyrosine kinase [ *Arabidopsis thaliana* ] | 1E-09 |
| ppa007250m | gi|225462522| | XP_002514384.1 | -1.37 | 10.1.30.1 | cell wall.precursor synthesis.sugar kinases.galacturonic acid kinase | Galactokinase, putative [ *Ricinus communis* ] | 0 |
| ppa006812m | gi|147858322| | XP_002522328.1 | -1.37 | 27.3.20 | RNA.regulation of transcription.G2-like transcription factor family, GARP | DNA binding protein, putative [ *Ricinus communis* ] | 4E-71 |
| ppa000626m | gi|225449440| | XP_002524200.1 | -1.37 | 35.1.12 | not assigned.no ontology.pumilio/Puf RNA-binding domain-containing protein | Pumilio, putative [ *Ricinus communis* ] | 0 |
| ppa023632m | - | unkown | -1.37 | 35.2 | not assigned.unknown | Unkown | 0 |
| ppa000434m | gi|224120412| | EOY21534.1 | -1.37 | 29.4 | protein.postranslational modification | Kinase domain-containing protein isoform 1   [ *Theobroma cacao* ] | 0 |
| ppa001754m | gi|225427993| | XP_003631542.1 | -1.37 | 29.5.1 | protein.degradation.subtilases | Subtilisin-like protease-like [ *Vitis vinifera* ] | 0 |
| ppa004050m | gi|225430390| | XP_002514088.1 | -1.37 | 13.1.3.2.1 | amino acid metabolism.synthesis.aspartate family.threonine.threonine synthase | Threonine synthase, putative [ *Ricinus communis* ] | 0.34 |
| ppa011522m | gi|225463868| | XP_002521071.1 | -1.37 | 35.1 | not assigned.no ontology | Transferase, transferring glycosyl groups, putative [ *Ricinus communis* ] | 9.5 |
| ppa011243m | gi|255581578| | XP_002531594.1 | -1.38 | 29.2.1.2.1.5 | protein.synthesis.ribosomal protein.eukaryotic.40S subunit.S5 | 40S ribosomal protein S5, putative [ *Ricinus communis* ] | 9E-142 |
| ppa000058m | gi|224061471| | XP_002300496.1 | -1.38 | 31.1 | cell.organisation | Microtubule organization protein [ *Populus trichocarpa* ] | 0 |
| ppa007135m | gi|255584961| | XP_002533192.1 | -1.38 | 13.1.3.6.1.2 | amino acid metabolism.synthesis.aspartate family.misc.homoserine.aspartate semialdehyde dehydrogenase | Aspartate semialdehyde dehydrogenase, putative [ *Ricinus communis* ] | 2E-162 |
| ppa008641m | gi|225445442| | XP_002524462.1 | -1.39 | 21.99 | redox.misc | NADH-cytochrome B5 reductase, putative [ *Ricinus communis* ] | 3E-39 |
| ppa008261m | gi|225451439| | EOY02792.1 | -1.39 | 29.5 | protein.degradation | Peptidase family M48 family protein [ *Theobroma cacao* ] | 0 |
| ppa007263m | gi|255575275| | XP_002518477.1 | -1.39 | 1.1.4.4 | PS.lightreaction.ATP synthase.gamma chain | ATP synthase gamma chain 2, chloroplast, putative [ *Ricinus communis* ] | 1E-52 |
| ppa002484m | gi|147792453| | NP_001011344.1 | -1.39 | 35.2 | not assigned.unknown | Transmembrane protein 209 [ *Xenopus (Silurana) tropicalis* ] | 0 |
| ppa010398m | gi|255542546| | XP_002512336.1 | -1.39 | 35.2 | not assigned.unknown | Conserved hypothetical protein [ *Ricinus communis* ] | 2E-172 |
| ppa008208m | gi|225437697| | XP_002279985.1 | -1.39 | 35.2 | not assigned.unknown | Uncharacterized protein LOC100253796 [ *Vitis vinifera* ] | 0 |
| ppa007284m | gi|255543415| | XP_002512770.1 | -1.39 | 28.2 | DNA.repair | Uv excision repair protein rad23, putative [ *Ricinus communis* ] | 0 |
| ppa007628m | gi|255561108| | XP_002521566.1 | -1.39 | 3.5 | minor CHO metabolism.others | Aldose 1-epimerase, putative [ *Ricinus communis* ] | 0 |
| ppa011725m | gi|296083823| | XP_002515034.1 | -1.39 | 1.1.1.2 | PS.lightreaction.photosystem II.PSII polypeptide subunits | Photosystem II core complex proteins psbY, chloroplast precursor [ *Ricinus communis* ] | 0 |
| ppa000923m | gi|255580622| | XP_002531134.1 | -1.39 | 29.1.7 | protein.aa activation.alanine-tRNA ligase | Alanyl-trna synthetase, putative [ *Ricinus communis* ] | 0 |
| ppa009005m | gi|225425991| | XP_002304554.1 | -1.39 | 27.3.3 | RNA.regulation of transcription.AP2/EREBP, APETALA2/Ethylene-responsive element binding protein family | AP2/ERF domain-containing transcription factor [ *Populus trichocarpa* ] | 0 |
| ppa014026m | gi|225432120| | EAY83543.1 | -1.39 | 35.2 | not assigned.unknown | Hypothetical protein osi_38754 [ *Oryza sativa Indica Group* ] | 0 |
| ppa000698m | gi|225446720| | NP_172244.2 | -1.39 | 30.2.17 | signalling.receptor kinases.DUF 26 | Leucine-rich repeat transmembrane protein kinase, putative [ *Arabidopsis thaliana* ] | 2E-97 |
| ppa013348m | gi|225442069| | XP_002532727.1 | -1.39 | 27.1 | RNA.processing | Lsm1, putative [ *Ricinus communis* ] | 0 |
| ppa003900m | gi|255579535| | XP_002530610.1 | -1.39 | 17.3.1.2.99 | hormone metabolism.brassinosteroid.synthesis-degradation.sterols.other | Squalene monooxygenase, putative [ *Ricinus communis* ] | 0 |
| ppa1027211m | gi|225459760| | CAA71103.1 | -1.39 | 35.2 | not assigned.unknown | CDSP32 protein (Chloroplast Drought-induced Stress Protein of 32kda) [ *Solanum tuberosum* ] | 0 |
| ppa023132m | gi|147853974| | CAN81695.1 | -1.39 | 35.2 | not assigned.unknown | Hypothetical protein VITISV_042576 [ *Vitis vinifera* ] | 0 |
| ppa004301m | gi|225467408| | XP_002531094.1 | -1.39 | 26.1 | misc.cytochrome P450 | Cytochrome P450, putative [ *Ricinus communis* ] | 0 |
| ppa003542m | gi|154968281| | ABS88997.1 | -1.39 | 29.4 | protein.postranslational modification | Calcium-dependent calmodulin-independent protein kinase [ *Malus hupehensis* ] | 1E-61 |
| ppa018551m | gi|255538700| | XP_002510415.1 | -1.39 | 31.4 | cell.vesicle transport | Auxilin, putative [ *Ricinus communis* ] | 0 |
| ppa012449m | gi|255548479| | NP_191814.1 | -1.40 | 20.2.2 | stress.abiotic.cold | Universal stress protein (USP) family protein [ *Arabidopsis thaliana* ] | 1E-52 |
| ppa008021m | gi|225434189| | BAA89423.1 | -1.40 | 26.7 | misc.oxidases - copper, flavone etc | Allyl alcohol dehydrogenase [ *Nicotiana tabacum* ] | 2E-87 |
| ppa005095m | gi|224055715| | XP_002509535.1 | -1.40 | 23.3.1.3 | nucleotide metabolism.salvage.phosphoribosyltransferases.upp | Uracil phosphoribosyltransferase, putative [ *Ricinus communis* ] | 0 |
| ppa012617m | gi|225429115| | ADN43418.1 | -1.40 | 20.1 | stress.biotic | Pathogenesis-related protein 1 [ *Vitis hybrid cultivar* ] | 1E-76 |
| ppa019301m | gi|255576160| | XP_002528974.1 | -1.40 | 35.2 | not assigned.unknown | D-alanyl-d-alanine carboxypeptidase, putative [ *Ricinus communis* ] | 4E-161 |
| ppa002749m | gi|255571911| | XP_002526898.1 | -1.40 | 29.1.19 | protein.aa activation.arginine-tRNA ligase | Arginyl-trna--protein transferase, putative [ *Ricinus communis* ] | 0 |
| ppa000997m | gi|297734509| | XP_002511091.1 | -1.41 | 27.4 | RNA.RNA binding | RNA-binding protein, putative [ *Ricinus communis* ] | 0 |
| ppa006204m | gi|224098004| | XP_002522486.1 | -1.41 | 29.5.11.20 | protein.degradation.ubiquitin.proteasom | 26S proteasome subunit S9, putative [ *Ricinus communis* ] | 1E-164 |
| ppa000574m | gi|255573673| | EOY04911.1 | -1.41 | 27.3.69 | RNA.regulation of transcription.SET-domain transcriptional regulator family | Trithorax-like protein 2 isoform 1 [ *Theobroma cacao* ] | 0 |
| ppa002583m | gi|225436140| | XP_002524053.1 | -1.41 | 27.3.22 | RNA.regulation of transcription.HB,Homeobox transcription factor family | Bel1 homeotic protein, putative [ *Ricinus communis* ] | 1E-56 |
| ppa007695m | - | unkown | -1.41 | 35.2 | not assigned.unknown | Unkown | 0 |
| ppa011438m | gi|255583570| | NP_566396.1 | -1.41 | 35.1 | not assigned.no ontology | NHL1 [ *Arabidopsis thaliana* ] | 1E-170 |
| ppa011254m | gi|255547273| | XP_002514694.1 | -1.41 | 31.4 | cell.vesicle transport | Synaptosomal associated protein, putative [ *Ricinus communis* ] | 0 |
| ppa012419m | gi|18423437| | NP_568778.1 | -1.41 | 9.1.2 | mitochondrial electron transport / ATP synthesis.NADH-DH.localisation not clear | NADH-ubiquinone oxidoreductase-related [ *Arabidopsis thaliana* ] | 0 |
| ppa000148m | gi|225432614| | EOY05638.1 | -1.41 | 30.11 | signalling.light | Time for coffee, putative isoform 2 [ *Theobroma cacao* ] | 0 |
| ppa006025m | gi|26892028| | AAN84537.1 | -1.41 | 12.2.2 | N-metabolism.ammonia metabolism.glutamine synthetase | Putative plastidic glutamine synthetase [ *Crataegus crus-galli* ] | 0 |
| ppa009513m | gi|255569410| | XP_002525672.1 | -1.41 | 26.12 | misc.peroxidases | Peroxidase 44 precursor, putative [ *Ricinus communis* ] | 4E-38 |
| ppa002904m | gi|225445585| | XP_002285365.1 | -1.42 | 30.5 | signalling.G-proteins | Gtpase-activating protein gyp7-like [ *Vitis vinifera* ] | 0 |
| ppa008273m | gi|255569153| | XP_002525545.1 | -1.42 | 35.1 | not assigned.no ontology | Catalytic, putative [ *Ricinus communis* ] | 5E-37 |
| ppa000095m | gi|296081595| | XP_002867227.1 | -1.42 | 35.2 | not assigned.unknown | Binding protein [ *Arabidopsis lyrata subsp. Lyrata* ] | 0 |
| ppa009927m | gi|255546001| | NP_175547.1 | -1.42 | 35.1 | not assigned.no ontology | DABB1 (DIMERIC A/B BARREL DOMAINS-PROTEIN 1) [ *Arabidopsis thaliana* ] | 0 |
| ppa004316m | gi|225434102| | XP_002530577.1 | -1.42 | 29.2.3 | protein.synthesis.initiation | Eukaryotic translation initiation factor 3 subunit, putative [ *Ricinus communis* ] | 0 |
| ppa006623m | gi|225440083| | XP_002282555.1 | -1.42 | 35.2 | not assigned.unknown | Uncharacterized protein LOC100251727 [ *Vitis vinifera* ] | 0 |
| ppa012334m | gi|297738227| | YP_004151523.1 | -1.42 | 35.2 | not assigned.unknown | Protein of unknown function DUF177 [ *Thermovibrio ammonificans HB-1* ] | 4E-39 |
| ppa013610m | gi|225445444| | CBZ25591.1 | -1.42 | 35.2 | not assigned.unknown | Periodic tryptophan protein 2-like protein [ *Leishmania mexicana MHOM/GT/2001/U1103* ] | 0 |
| ppa006349m | gi|225443766| | XP_002522231.1 | -1.42 | 29.4 | protein.postranslational modification | Glycogen synthase kinase-3 beta, putative [ *Ricinus communis* ] | 1E-100 |
| ppa009314m | gi|224133570| | NP_001151326.1 | -1.42 | 35.2 | not assigned.unknown | Uvrb/uvrc motif family protein [ *Zea mays* ] | 3E-146 |
| ppa002006m | gi|255582880| | XP_002532212.1 | -1.42 | 11.1.9 | lipid metabolism.FA synthesis and FA elongation.long chain fatty acid CoA ligase | Long-chain-fatty-acid coa ligase, putative [ *Ricinus communis* ] | 1E-180 |
| ppa005393m | gi|297741477| | XP_002531335.1 | -1.42 | 13.1.2.3.2 | amino acid metabolism.synthesis.glutamate family.arginine.acetylglutamate kinase | Arginine biosynthesis protein argj 1, putative [ *Ricinus communis* ] | 1.8 |
| ppa012823m | gi|224110818| | NP_563737.1 | -1.42 | 1.1.1.2 | PS.lightreaction.photosystem II.PSII polypeptide subunits | Photosystem II D1 precursor processing protein PSB27-H2 [ *Arabidopsis thaliana* ] | 0 |
| ppa005590m | gi|255539070| | XP_002510600.1 | -1.42 | 17.3.1.2.2 | hormone metabolism.brassinosteroid.synthesis-degradation.sterols.SMT2 | S-adenosyl-l-methionine:delta24-sterol-C-methyltransferase, putative [ *Ricinus communis* ] | 0 |
| ppa004638m | gi|296083935| | XP_002523243.1 | -1.42 | 27.1.19 | RNA.processing.ribonucleases | Transcription factor, putative [ *Ricinus communis* ] | 0 |
| ppa005267m | gi|255583293| | XP_002532410.1 | -1.43 | 29.4 | protein.postranslational modification | CBL-interacting serine/threonine-protein kinase, putative [ *Ricinus communis* ] | 0 |
| ppa011088m | gi|225433658| | NP_194528.1 | -1.43 | 33.99 | development.unspecified | YGGT family protein [ *Arabidopsis thaliana* ] | 2E-176 |
| ppa005362m | gi|255543315| | NP_563986.1 | -1.43 | 35.2 | not assigned.unknown | NDF1 (NDH-DEPENDENT CYCLIC ELECTRON FLOW 1) [ *Arabidopsis thaliana* ] | 6E-149 |
| ppa009283m | gi|28629811| | AAO45179.1 | -1.43 | 27.3.26 | RNA.regulation of transcription.MYB-related transcription factor family | Transcription factor Myb1 [ *Malus xiaojinensis* ] | 0 |
| ppa010089m | gi|224137948| | XP_002524363.1 | -1.43 | 27.3.23 | RNA.regulation of transcription.HSF,Heat-shock transcription factor family | Transcription factor, putative [ *Ricinus communis* ] | 4.00E-124 |
| ppa012122m | gi|255568247| | XP_002525098.1 | -1.43 | 29.5.11.3 | protein.degradation.ubiquitin.E2 | Ubiquitin-conjugating enzyme m, putative [ *Ricinus communis* ] | 0 |
| ppa003953m | gi|225457921| | XP_002510559.1 | -1.43 | 35.1.19 | not assigned.no ontology.C2 domain-containing protein | Calcium lipid binding protein, putative [ *Ricinus communis* ] | 0 |
| ppa008311m | gi|224086126| | BAF76429.1 | -1.43 | 27.3.35 | RNA.regulation of transcription.bZIP transcription factor family | Basic region leucine zipper protein [ *Nicotiana tabacum* ] | 0 |
| ppa008400m | gi|224071155| | XP_002531467.1 | -1.44 | 35.2 | not assigned.unknown | Protein-tyrosine phosphatase mitochondrial 1, mitochondrial precursor, putative [ *Ricinus communis* ] | 3.6 |
| ppa013881m | gi|224106387| | XP_002520493.1 | -1.44 | 29.5.11.4.3.1 | protein.degradation.ubiquitin.E3.SCF.SKP | Transcription elongation factor B polypeptide, putative [ *Ricinus communis* ] | 0 |
| ppa005318m | gi|147783056| | XP_002531355.1 | -1.44 | 16.2 | secondary metabolism.phenylpropanoids | Anthocyanin 5-aromatic acyltransferase, putative [ *Ricinus communis* ] | 0 |
| ppa009418m | gi|255568141| | XP_002525046.1 | -1.44 | 23.4.1 | nucleotide metabolism.phosphotransfer and pyrophosphatases.adenylate kinase | Adenylate kinase 1 chloroplast, putative [ *Ricinus communis* ] | 7E-119 |
| ppa008779m | gi|225459119| | XP_002514023.1 | -1.44 | 34.9 | transport.metabolite transporters at the mitochondrial membrane | Mitochondrial dicarboxylate carrier protein, putative [ *Ricinus communis* ] | 3E-92 |
| ppa009188m | gi|297738696| | XP_002282754.1 | -1.44 | 35.2 | not assigned.unknown | Unnamed protein product [ *Vitis vinifera* ] | 0 |
| ppa010684m | gi|124360377| | ABN08390.1 | -1.44 | 35.2 | not assigned.unknown | Harpin-induced 1 [ *Medicago truncatula* ] | 1E-148 |
| ppa014453m | gi|224115602| | XP_002317076.1 | -1.44 | 35.2 | not assigned.unknown | Predicted protein   [ *Populus trichocarpa* ] | 2.00E-41 |
| ppa000230m | gi|225448150| | XP_002528999.1 | -1.44 | 28.1 | DNA.synthesis/chromatin structure | ATP-dependent RNA helicase, putative [ *Ricinus communis* ] | 1E-130 |
| ppa002527m | gi|225459683| | XP_002881674.1 | -1.44 | 20.2.3 | stress.abiotic.drought/salt | Dehydration-responsive family protein [ *Arabidopsis lyrata subsp. Lyrata* ] | 0 |
| ppa007809m | gi|255634606| | ABI31652.1 | -1.45 | 29.5.11.20 | protein.degradation.ubiquitin.proteasom | 26S proteasome regulatory particle non-atpase subunit 12 [ *Camellia sinensis* ] | 0 |
| ppa004501m | gi|255554517| | XP_002313948.1 | -1.45 | 35.1 | not assigned.no ontology | Sugar transporter/spinster transmembrane protein [ *Populus trichocarpa* ] | 0 |
| ppa012545m | gi|225425386| | XP_002890719.1 | -1.45 | 35.2 | not assigned.unknown | Hypothetical protein ARALYDRAFT_472926 [ *Arabidopsis lyrata subsp. Lyrata* ] | 5.6 |
| ppa004538m | gi|224132038| | XP_002527263.1 | -1.45 | 29.5.5 | protein.degradation.serine protease | Serine carboxypeptidase, putative [ *Ricinus communis* ] | 3E-51 |
| ppa020977m | gi|255560677| | XP_002521352.1 | -1.45 | 27.3.14 | RNA.regulation of transcription.CCAAT box binding factor family, HAP2 | Nuclear transcription factor Y subunit A-3, putative [ *Ricinus communis* ] | 1E-47 |
| ppa005667m | gi|224126333| | YP_004001458.1 | -1.45 | 35.2 | not assigned.unknown | Multi-sensor signal transduction histidine kinase [ *Caldicellulosiruptor owensensis OL* ] | 0 |
| ppa001260m | gi|255550313| | XP_002516207.1 | -1.45 | 30.4 | signalling.phosphinositides | Phosphoinositide 5-phosphatase, putative [ *Ricinus communis* ] | 0 |
| ppa003664m | gi|224098814| | XP_002518823.1 | -1.45 | 35.2 | not assigned.unknown | Yth domain-containing protein, putative [ *Ricinus communis* ] | 0 |
| ppa012129m | gi|297845088| | ZP_06124440.1 | -1.45 | 35.2 | not assigned.unknown | Tonb-dependent receptor [ *Providencia rettgeri DSM 1131* ] | 3E-104 |
| ppa001368m | gi|255544686| | XP_002513404.1 | -1.45 | 29.2.4 | protein.synthesis.elongation | Eukaryotic translation elongation factor, putative [ *Ricinus communis* ] | 0 |
| ppa020028m | gi|225427364| | XP_002517514.1 | -1.45 | 29.5.5 | protein.degradation.serine protease | Placental protein 11 precursor, putative [ *Ricinus communis* ] | 6E-129 |
| ppa006632m | gi|225470311| | XP_002509611.1 | -1.46 | 13.2.5.2 | amino acid metabolism.degradation.serine-glycine-cysteine group.glycine | Aminomethyltransferase, putative [ *Ricinus communis* ] | 1E-32 |
| ppa013543m | gi|255638476| | XP_002304244.1 | -1.46 | 17.2.3 | hormone metabolism.auxin.induced-regulated-responsive-activated | SAUR family protein [ *Populus trichocarpa* ] | 0 |
| ppa001670m | gi|225432900| | XP_002517022.1 | -1.46 | 29.3.4.2 | protein.targeting.secretory pathway.golgi | Protein transport protein sec23, putative [ *Ricinus communis* ] | 1E-178 |
| ppa008591m | gi|225449158| | EGG21685.1 | -1.46 | 35.2 | not assigned.unknown | DUF410 family protein [ *Dictyostelium fasciculatum* ] | 6E-24 |
| ppa007075m | gi|225453144| | XP_002882294.1 | -1.46 | 29.5.11.4.2 | protein.degradation.ubiquitin.E3.RING | Armadillo/beta-catenin repeat family protein [ *Arabidopsis lyrata subsp. Lyrata* ] | 0 |
| ppa005702m | gi|209922600| | ACI96243.1 | -1.46 | 29.2.4 | protein.synthesis.elongation | Elongation factor 1-alpha [ *Prunus persica* ] | 5E-78 |
| ppa006402m | gi|225433918| | AAX33234.1 | -1.46 | 2.2.2.1.1 | major CHO metabolism.degradation.starch.starch cleavage.alpha amylase | Cytosolic alpha-amylase [ *Malus x domestica* ] | 8E-162 |
| ppa006504m | gi|297740322| | XP_002533696.1 | -1.46 | 27.3.6 | RNA.regulation of transcription.bHLH,Basic Helix-Loop-Helix family | Basic helix-loop-helix-containing protein, putative [ *Ricinus communis* ] | 9E-95 |
| ppa011201m | gi|224084028| | NP_173506.1 | -1.46 | 27.3.99 | RNA.regulation of transcription.unclassified | Zinc finger (C3HC4-type RING finger) family protein [ *Arabidopsis thaliana* ] | 0 |
| ppa003218m | gi|296084280| | CBI24668.3 | -1.47 | 35.1 | not assigned.no ontology | Unnamed protein product [ *Vitis vinifera* ] | 0 |
| ppa008574m | gi|297743328| | XP_002304283.1 | -1.47 | 1.20 | PS.photorespiration | Formyltetrahydrofolate deformylase [ *Populus trichocarpa* ] | 5E-114 |
| ppa012407m | gi|225426405| | XP_002299535.1 | -1.47 | 21.2.2 | redox.ascorbate and glutathione.glutathione | Glutathione peroxidase [ *Populus trichocarpa* ] | 2E-165 |
| ppa003458m | gi|224116936| | AAZ05069.1 | -1.47 | 5.2 | fermentation.PDC | Pyruvate decarboxylase [ *Citrus sinensis* ] | 2E-120 |
| ppa013691m | gi|255626799| | XP_002512926.1 | -1.47 | 29.2.1.2.1.25 | protein.synthesis.ribosomal protein.eukaryotic.40S subunit.S25 | 40S ribosomal protein S25-1, putative [ *Ricinus communis* ] | 7E-09 |
| ppa014089m | gi|222636094| | NP_001118788.1 | -1.47 | 29.2.1.2.2.537 | protein.synthesis.ribosomal protein.eukaryotic.60S subunit.L37A | Ubiquitin thiolesterase/ zinc ion binding [ *Arabidopsis thaliana* ] | 0 |
| ppa001443m | gi|225449130| | XP_002529704.1 | -1.47 | 34.9 | transport.metabolite transporters at the mitochondrial membrane | Mitochondrial carrier protein, putative [ *Ricinus communis* ] | 2E-169 |
| ppa006836m | gi|40457267| | ABK76304.1 | -1.47 | 1.3.9 | PS.calvin cycle.seduheptulose bisphosphatase | Chloroplast sedoheptulose-1,7-bisphosphatase [ *Morus alba var. Multicaulis* ] | 2E-49 |
| ppa006185m | gi|255538976| | XP_002510553.1 | -1.47 | 27.3.99 | RNA.regulation of transcription.unclassified | Zinc finger protein, putative [ *Ricinus communis* ] | 0 |
| ppa023049m | gi|224065274| | NP_189013.1 | -1.47 | 29.3.3 | protein.targeting.chloroplast | Chloroplast inner membrane import protein Tic22, putative [ *Arabidopsis thaliana* ] | 0 |
| ppa007055m | gi|255555606| | XP_002894323.1 | -1.47 | 35.2 | not assigned.unknown | FMN binding protein [ *Arabidopsis lyrata subsp. Lyrata* ] | 1E-28 |
| ppa000629m | gi|225439821| | XP_002277306.1 | -1.47 | 30.3 | signalling.calcium | Calcium-transporting atpase 4, endoplasmic reticulum-type-like [ *Vitis vinifera* ] | 0 |
| ppa015545m | gi|224053521| | XP_002514877.1 | -1.47 | 7.1.1 | OPP.oxidative PP.G6PD | Glucose-6-phosphate 1-dehydrogenase, putative [ *Ricinus communis* ] | 0 |
| ppa002869m | gi|255584380| | XP_002532924.1 | -1.48 | 35.1 | not assigned.unknown | Transferase, transferring glycosyl groups, putative [ *Ricinus communis* ] | 3E-142 |
| ppa014464m | gi|255584312| | AAM65256.1 | -1.48 | 35.2 | not assigned.unknown | Pollen coat-like protein [ *Arabidopsis thaliana* ] | 0 |
| ppa009919m | gi|225433872| | XP_002264839.2 | -1.48 | 29.4 | protein.postranslational modification | Probable serine/threonine-protein kinase GCN2-like [ *Vitis vinifera* ] | 0 |
| ppa004824m | gi|297745973| | XP_002280021.2 | -1.48 | 29.4.1.57 | protein.postranslational modification.kinase.receptor like cytoplasmatic kinase VII | Probable serine/threonine-protein kinase At5g41260-like [ *Vitis vinifera* ] | 0 |
| ppa009987m | gi|255539358| | XP_002525758.1 | -1.48 | 1.1.1.1 | PS.lightreaction.photosystem II.LHC-II | Chlorophyll A/B binding protein, Lhcb3[ *Ricinus communis* ] | 0.00003 |
| ppa008038m | gi|224127592| | YP_003481617.1 | -1.48 | 35.2 | not assigned.unknown | Chromosome segregation atpase-like protein [ *Natrialba magadii ATCC 43099* ] | 0 |
| ppa011583m | gi|224119124| | XP_002317991.1 | -1.48 | 35.2 | not assigned.unknown | Predicted protein [ *Populus trichocarpa* ] | 1.00E-134 |
| ppa004001m | gi|147774578| | XP_002876248.1 | -1.48 | 27.4 | RNA.RNA binding | Nucleic acid binding protein [ *Arabidopsis lyrata subsp. Lyrata* ] | 5.2 |
| ppa013178m | gi|255627701| | NP_564382.1 | -1.48 | 35.2 | not assigned.unknown | SWIB complex BAF60b domain-containing protein [ *Arabidopsis thaliana* ] | 7E-153 |
| ppa010661m | gi|224140695| | XP_002529105.1 | -1.48 | 13.1.3.4.13 | amino acid metabolism.synthesis.aspartate family.methionine.methionine S-methyltransferase | Thiopurine S-methyltransferase, putative [ *Ricinus communis* ] | 0 |
| ppa007078m | gi|255567786| | NP_564053.1 | -1.48 | 26.27 | misc.calcineurin-like phosphoesterase family protein | Calcineurin-like phosphoesterase family protein [ *Arabidopsis thaliana* ] | 0 |
| ppa022930m | gi|34851124|g | AAL13082.1 | -1.49 | 27.4 | RNA.RNA binding | Putative glycine-rich RNA-binding protein [ *Prunus avium* ] | 0 |
| ppa000559m | gi|224066625| | XP_002302169.1 | -1.49 | 10.2.1 | cell wall.cellulose synthesis.cellulose synthase | Cellulose synthase [ *Populus trichocarpa* ] | 1E-143 |
| ppa006262m | gi|225425762| | XP_002520117.1 | -1.49 | 29.4.1.57 | protein.postranslational modification.kinase.receptor like cytoplasmatic kinase VII | Protein kinase APK1A, chloroplast precursor, putative [ *Ricinus communis* ] | 0 |
| ppa003320m | gi|225449214| | AAZ66745.1 | -1.49 | 29.5.11.4.3.2 | protein.degradation.ubiquitin.E3.SCF.FBOX | Coronatine-insensitive 1 [ *Glycine max* ] | 3E-130 |
| ppa026991m | gi|224135523| | XP_002874884.1 | -1.49 | 27.3.99 | RNA.regulation of transcription.unclassified | Mitochondrial transcription termination factor family protein [ *Arabidopsis lyrata subsp. Lyrata* ] | 0 |
| ppa026735m | gi|225464604| | NP_001151720.1 | -1.49 | 35.2 | not assigned.unknown | ABC-type Co2+ transport system, permease component [ *Zea mays* ] | 1E-154 |
| ppa003104m | gi|225439610| | XP_002533826.1 | -1.49 | 31.4 | cell.vesicle transport | Protein transporter, putative [ *Ricinus communis* ] | 7E-45 |
| ppa009996m | gi|225469306| | XP_002318844.1 | -1.49 | 35.2 | not assigned.unknown | Predicted protein   [ *Populus trichocarpa* ] | 2.00E-78 |
| ppa010686m | gi|88604775|g | ABD46741.1 | -1.49 | 34.19.4 | transport.Major Intrinsic Proteins.SIP | Small basic intrinsic protein 1 [ *Vitis vinifera* ] | 1E-82 |
| ppa002539m | gi|255585146| | XP_002533278.1 | -1.49 | 29.4.1.57 | protein.postranslational modification.kinase.receptor like cytoplasmatic kinase VII | BRASSINOSTEROID INSENSITIVE 1-associated receptor kinase 1 precursor, putative [ *Ricinus communis* ] | 3E-142 |
| ppa012497m | - | unkown | -1.50 | 35.2 | not assigned.unknown | Unkown | 0 |
| ppa006272m | gi|225435020| | XP_002528441.1 | -1.50 | 35.2 | not assigned.unknown | Amino acid binding protein, putative [ *Ricinus communis* ] | 4E-161 |
| ppa007946m | gi|255579673| | XP_002530676.1 | -1.50 | 29.4 | protein.postranslational modification | Serine/threonine-protein kinase ASK1, putative [ *Ricinus communis* ] | 0 |
| ppa010172m | gi|224134122| | XP_002869043.1 | -1.50 | 29.2.1.2.2.8 | protein.synthesis.ribosomal protein.eukaryotic.60S subunit.L8 | 60S ribosomal protein L8 [ *Arabidopsis lyrata subsp. Lyrata* ] | 2E-41 |
| ppa022572m | gi|225456205| | NP_173549.1 | -1.50 | 29.4.1.57 | protein.postranslational modification.kinase.receptor like cytoplasmatic kinase VII | WAK2; ATP binding / calcium ion binding / protein kinase/ protein serine/threonine kinase [ *Arabidopsis thaliana* ] | 0 |
| ppa010809m | gi|255539785| | ACF22769.1 | -1.50 | 35.2 | not assigned.unknown | DUF614 containing protein [ *Brachypodium distachyon* ] | 0.028 |
| ppa002323m | gi|296081618| | NP_180167.1 | -1.51 | 27.4 | RNA.RNA binding | KH domain-containing protein [ *Arabidopsis thaliana* ] | 0.044 |
| ppa003163m | gi|255553847| | XP_002517964.1 | -1.51 | 17.2.3 | hormone metabolism.auxin.induced-regulated-responsive-activated | Indole-3-acetic acid-amido synthetase GH3.5, putative [ *Ricinus communis* ] | 0 |
| ppa012568m | gi|255550341| | XP_002516221.1 | -1.51 | 26.3 | misc. other Ferredoxins and Rieske domain | Adrenodoxin, putative [ *Ricinus communis* ] | 0 |
| ppa009540m | gi|13486942|d | BAB40143.1 | -1.51 | 34.19.1 | transport.Major Intrinsic Proteins.PIP | Plasma membrane intrinsic protein 2-2 [ *Pyrus communis* ] | 0 |
| ppa008584m | gi|217073071| | NP_187503.1 | -1.51 | 20.2.1 | stress.abiotic.heat | DNAJ heat shock protein, putative [ *Arabidopsis thaliana* ] | 0 |
| ppa000919m | gi|297738122| | XP_002517429.1 | -1.51 | 35.2 | not assigned.unknown | Nop14, putative [ *Ricinus communis* ] | 2E-107 |
| ppa006207m | gi|79473201| | NP_189079.2 | -1.51 | 13.2.6.3 | amino acid metabolism.degradation.aromatic aa.tryptophan | 3-hydroxyisobutyryl-coa hydrolase-like protein 4   [ *Arabidopsis thaliana* ] | 0 |
| ppa005007m | gi|255571071| | XP_002526486.1 | -1.51 | 30.4.1 | signalling.phosphinositides.phosphatidylinositol-4-phosphate 5-kinase | 1-phosphatidylinositol-4-phosphate 5-kinase, putative [ *Ricinus communis* ] | 0 |
| ppa005124m | gi|225429928| | EOY29758.1 | -1.52 | 31.2 | cell.division | Regulator of chromosome condensation family protein isoform 2 [ *Theobroma cacao* ] | 0 |
| ppa007559m | gi|225461886| | ACG39671.1 | -1.52 | 34.9 | transport.metabolite transporters at the mitochondrial membrane | Import inner membrane translocase subunit TIM50 [ *Zea mays* ] | 1E-151 |
| ppa009750m | gi|255563224| | XP_002522615.1 | -1.52 | 27.3.67 | RNA.regulation of transcription.putative transcription regulator | Pentatricopeptide repeat-containing protein, putative [ *Ricinus communis* ] | 0 |
| ppa012523m | gi|225430762| | NP_190655.2 | -1.52 | 31.4 | cell.vesicle transport | Clathrin adaptor complex small chain family protein [ *Arabidopsis thaliana* ] | 0 |
| ppa006873m | gi|255639455| | NP_564560.1 | -1.52 | 29.5.5 | protein.degradation.serine protease | CLPR1; serine-type endopeptidase [ *Arabidopsis thaliana* ] | 2E-112 |
| ppa017473m | gi|118488240| | XP_002515493.1 | -1.52 | 26.21 | misc.protease inhibitor/seed storage/lipid transfer protein (LTP) family protein | 14 kda proline-rich protein DC2.15 precursor, putative [ *Ricinus communis* ] | 0 |
| ppa007621m | gi|224128940| | XP_002329004.1 | -1.52 | 17.2.2 | hormone metabolism.auxin.signal transduction | Auxin efflux carrier component [ *Populus trichocarpa* ] | 5E-128 |
| ppa011092m | gi|225439060| | XP_002519976.1 | -1.52 | 35.2 | not assigned.unknown | GTP binding protein, putative [ *Ricinus communis* ] | 4E-180 |
| ppa002614m | gi|60652323| | XP_002532011.1 | -1.52 | 2.2.1.3.1 | major CHO metabolism.degradation.sucrose.invertases.neutral | Beta-fructofuranosidase, putative [ *Ricinus communis* ] | 3E-29 |
| ppa024270m | gi|225429171| | XP_002522090.1 | -1.52 | 29.1.12 | protein.aa activation.aspartate-tRNA ligase | Aspartyl-trna synthetase, putative [ *Ricinus communis* ] | 4E-70 |
| ppa009883m | gi|224117478| | XP_002523001.1 | -1.53 | 30.5 | signalling.G-proteins | Sgp1 monomeric G-protein, putative [ *Ricinus communis* ] | 6E-38 |
| ppa004448m | gi|255574875| | XP_002528344.1 | -1.53 | 8.1.3 | TCA / org transformation.TCA.aconitase | 3-isopropylmalate dehydratase, putative [ *Ricinus communis* ] | 1E-139 |
| ppa009370m | gi|224110250| | ADP30960.1 | -1.53 | 20.2.3 | stress.abiotic.drought/salt | Dehydration-induced 19-like protein [ *Gossypium hirsutum* ] | 5E-147 |
| ppa003230m | gi|224113335| | CAN81742.1 | -1.53 | 35.2 | not assigned.unknown | Hypothetical protein VITISV_040850 [ *Vitis vinifera* ] | 0 |
| ppa009590m | gi|225461193| | XP_002864339.1 | -1.53 | 35.2 | not assigned.unknown | Hypothetical protein ARALYDRAFT_495534 [ *Arabidopsis lyrata subsp. Lyrata* ] | 0 |
| ppa001102m | gi|225457237| | NP_001267822.1 | -1.53 | 20.2.1 | stress.abiotic.heat | Heat shock protein 101   [ *Vitis vinifera* ] |  |
| ppa018500m | gi|255630659| | AAF18514.1 | -1.53 | 35.2 | not assigned.unknown | Ests gb|T41688, gb|AI992698, gb|AA394805 come from this gene [ *Arabidopsis thaliana* ] | 2E-85 |
| ppa007040m | gi|225431669| | NP_179334.4 | -1.54 | 21.1 | redox.thioredoxin | NTRA (nadph-dependent thioredoxin reductase a); thioredoxin-disulfide reductase [ *Arabidopsis thaliana* ] | 2E-169 |
| ppa022307m | gi|225433483| | XP_002514050.1 | -1.54 | 16.2 | secondary metabolism.phenylpropanoids | Anthranilate N-benzoyltransferase protein, putative [ *Ricinus communis* ] | 0 |
| ppa019445m | gi|255548844| | XP_002515478.1 | -1.54 | 35.1 | not assigned.no ontology | Heparanase, putative [ *Ricinus communis* ] | 0 |
| ppa005609m | gi|224083522| | XP_002869163.1 | -1.54 | 5.10 | fermentation.aldehyde dehydrogenase | ALDH3I1 [ *Arabidopsis lyrata subsp. Lyrata* ] | 1E-139 |
| ppa019630m | gi|296082193| | ABT18095.1 | -1.54 | 29.4.1.57 | protein.postranslational modification.kinase.receptor like cytoplasmatic kinase VII | FERONIA receptor-like kinase [ *Cardamine flexuosa* ] | 2E-76 |
| ppa024261m | gi|302142349| | XP_002510258.1 | -1.54 | 33.1 | development.storage proteins | Patatin T5 precursor, putative [ *Ricinus communis* ] | 3 |
| ppa010992m | gi|224056693| | NP_174575.1 | -1.54 | 29.2.1.1.1.2.11 | protein.synthesis.ribosomal protein.prokaryotic.chloroplast.50S subunit.L11 | PRPL11 receptor-like protein kinase1); structural constituent of ribosome [ *Arabidopsis thaliana* ] | 2E-79 |
| ppa006584m | gi|255635321| | AAO38707.1 | -1.54 | 34.12 | transport.metal | Cation diffusion facilitator 8 [ *Stylosanthes hamata* ] | 0 |
| ppb010066m | - | unkown | -1.54 | 35.2 | not assigned.unknown | Unkown | 0 |
| ppa010812m | gi|225427191| | XP_001031577.1 | -1.54 | 35.2 | not assigned.unknown | Cation channel family protein [ *Tetrahymena thermophila* ] | 1E-122 |
| ppa013578m | gi|225442892| | XP_002517672.1 | -1.55 | 35.1 | not assigned.no ontology | Hydrolase, putative [ *Ricinus communis* ] | 2E-169 |
| ppa010166m | gi|255562096| | CBL41185.1 | -1.55 | 35.2 | not assigned.unknown | Methylase involved in ubiquinone/menaquinone biosynthesis [ *butyrate-producing bacterium SS3/4* ] | 0.072 |
| ppa004239m | gi|225464065| | XP_002518129.1 | -1.55 | 26.7 | misc.oxidases - copper, flavone etc | Monoxygenase, putative [ *Ricinus communis* ] | 0 |
| ppa008626m | gi|255553925| | CBL41185.1 | -1.55 | 35.2 | not assigned.unknown | Methylase involved in ubiquinone/menaquinone biosynthesis [ *butyrate-producing bacterium SS3/4* ] | 4E-12 |
| ppa010395m | gi|224077818| | NP_001181421.1 | -1.55 | 35.1 | not assigned.no ontology | Nogo-B receptor [ *Macaca mulatta* ] | 0 |
| ppa010105m | gi|255561415| | XP_002521718.1 | -1.55 | 27.4 | RNA.RNA binding | Heterogeneous nuclear ribonucleoprotein, putative [ *Ricinus communis* ] | 0 |
| ppa025609m | gi|225434449| | EOY34611.1 | -1.55 | 26.27 | misc.calcineurin-like phosphoesterase family protein | Calcineurin-like metallo-phosphoesterase superfamily protein isoform 1 [ *Theobroma cacao* ] | 0 |
| ppa001332m | gi|297737669| | NP_173731.2 | -1.55 | 35.1.3 | not assigned.no ontology.armadillo/beta-catenin repeat family protein | Armadillo/beta-catenin repeat family protein [ *Arabidopsis thaliana* ] | 0 |
| ppa007767m | gi|12718822| | BAB32405.1 | -1.56 | 29.4 | protein.postranslational modification | NQK1 MAPKK [ *Nicotiana tabacum* ] | 6E-167 |
| ppa002663m | gi|255538090| | XP_002510110.1 | -1.56 | 35.2 | not assigned.unknown | Conserved hypothetical protein [ *Ricinus communis* ] | 0 |
| ppa006039m | gi|255636705| | ACB30362.1 | -1.56 | 27.3.11 | RNA.regulation of transcription.C2H2 zinc finger family | Transcription factor [ *Capsicum annuum* ] | 1E-78 |
| ppa011446m | gi|225448677| | XP_002520866.1 | -1.56 | 29.5.11.4.2 | protein.degradation.ubiquitin.E3.RING | RING-H2 finger protein ATL3C, putative [ *Ricinus communis* ] | 3E-29 |
| ppa007071m | gi|255542956| | XP_004302291.1 | -1.56 | 35.2 | not assigned.unknown | Chloroplast stem-loop binding protein of 41 kda b, chloroplastic-like [ *Fragaria vesca subsp. Vesca* ] | 0 |
| ppa008096m | gi|225444373| | XP_002267126.1 | -1.56 | 34.2 | transport.sugars | Uncharacterized protein LOC100243455 [ *Vitis vinifera* ] | 0 |
| ppa009903m | gi|224054274| | XP_002299309.1 | -1.56 | 1.1.1.1 | PS.lightreaction.photosystem II.LHC-II | Light-harvesting complex I protein Lhca2 [ *Populus trichocarpa* ] | 0 |
| ppa007531m | gi|297740117| | XP_002276674.1 | -1.56 | 13.1.7.3 | amino acid metabolism.synthesis.histidine.phosphoribosyl-AMP cyclohydrolase | 1-(5-phosphoribosyl)-5- [ *(5-phosphoribosylamino)methylideneamino imidazole-4-carboxamide isomerase* ] | 0 |
| ppa009745m | gi|225444525| | NP_194953.1 | -1.56 | 1.1.4.9 | PS.lightreaction.ATP synthase.subunit B_ (ATPX) | ATP synthase family [ *Arabidopsis thaliana* ] | 4E-65 |
| ppa003192m | gi|255548387| | XP_002515250.1 | -1.56 | 27.4 | RNA.RNA binding | RNA binding protein, putative [ *Ricinus communis* ] | 3E-170 |
| ppa010214m | gi|225443415| | XP_002268835.1 | -1.56 | 29.5.11.4.2 | protein.degradation.ubiquitin.E3.RING | E3 ubiquitin-protein ligase MARCH1 [ *Vitis vinifera* ] | 0 |
| ppa007815m | gi|255641342| | XP_002515502.1 | -1.56 | 35.1 | not assigned.no ontology | UDP-sugar transporter, putative [ *Ricinus communis* ] | 0 |
| ppa001739m | gi|225442092| | XP_002885009.1 | -1.56 | 29.5.1 | protein.degradation.subtilases | Subtilase family protein [ *Arabidopsis lyrata subsp. Lyrata* ] | 7E-31 |
| ppa000633m | gi|224080003| | XP_002517456.1 | -1.56 | 35.2 | not assigned.unknown | Acid phosphatase, putative [ *Ricinus communis* ] | 3.9 |
| ppa012420m | gi|225449555| | XP_002528433.1 | -1.56 | 29.5.11.3 | protein.degradation.ubiquitin.E2 | Ubiquitin-conjugating enzyme E2 G, putative [ *Ricinus communis* ] | 1E-138 |
| ppa011584m | gi|217073214| | NP_196647.1 | -1.57 | 35.1 | not assigned.no ontology | CBS domain-containing protein [ *Arabidopsis thaliana* ] | 1E-37 |
| ppa009584m | gi|225436956| | AAK56924.1 | -1.57 | 35.1 | not assigned.no ontology | Circadian clock coupling factor ZGT [ *Nicotiana tabacum* ] | 3E-129 |
| ppa008324m | gi|225445238| | XP_002280972.1 | -1.57 | 35.2 | not assigned.unknown | Uncharacterized protein LOC100249177 [ *Vitis vinifera* ] | 0 |
| ppa006084m | gi|225446088| | NP_568211.1 | -1.57 | 35.1 | not assigned.no ontology | CD2-binding protein-related [ *Arabidopsis thaliana* ] | 2E-151 |
| ppa010893m | gi|222427671| | ACM49845.1 | -1.57 | 17.5.2 | hormone metabolism.ethylene.signal transduction | Ethylene responsive transcription factor 3a [ *Prunus salicina* ] | 1E-81 |
| ppa008115m | gi|255645285| | XP_002524865.1 | -1.57 | 35.1 | not assigned.no ontology | WD-repeat protein, putative [ *Ricinus communis* ] | 0 |
| ppa019898m | gi|255557685| | NP_001150699.1 | -1.57 | 35.2 | not assigned.unknown | ABC-type Co2+ transport system, permease component [ *Zea mays* ] | 1E-83 |
| ppa003958m | gi|255543108| | XP_002512617.1 | -1.57 | 35.1 | not assigned.no ontology | Cytochrome oxidase biogenesis protein, putative [ *Ricinus communis* ] | 0 |
| ppa000822m | gi|225438408| | NP_193013.2 | -1.57 | 31.4 | stress.abiotic.heat | Heat shock protein binding [ *Arabidopsis thaliana* ] | 0 |
| ppa004276m | gi|224092091| | XP_002309468.1 | -1.57 | 17.4.1 | hormone metabolism.cytokinin.synthesis-degradation | Cytokinin oxidase [ *Populus trichocarpa* ] | 0.000001 |
| ppa025252m | - | unkown | -1.57 | 35.2 | not assigned.unknown | Unkown | 0 |
| ppa009826m | gi|225462509| | XP_002867929.1 | -1.57 | 35.2 | not assigned.unknown | Hypothetical protein ARALYDRAFT_492902 [ *Arabidopsis lyrata subsp. Lyrata* ] | 2E-42 |
| ppa011910m | gi|297742316| | CBI34465.3 | -1.57 | 35.2 | not assigned.unknown | Unnamed protein product [ *Vitis vinifera* ] | 0 |
| ppa015127m | gi|225464646| | BAI65910.1 | -1.58 | 26.2 | misc.UDP glucosyl and glucoronyl transferases | UDP-sugar:glycosyltransferase [ *Forsythia x intermedia* ] | 0 |
| ppa007844m | gi|225427518| | CAN82476.1 | -1.58 | 35.2 | not assigned.unknown | Hypothetical protein VITISV_038296 [ *Vitis vinifera* ] | 0 |
| ppa008943m | gi|224130546| | XP_002523339.1 | -1.59 | 33.99 | protein.degradation | Ubiquitin ligase SINAT3, putative [ *Ricinus communis* ] | 0 |
| ppa002606m | gi|255540853| | XP_002511491.1 | -1.59 | 10.3 | cell wall.hemicellulose synthesis | Galactosyltransferase, putative [ *Ricinus communis* ] | 0 |
| ppa009057m | gi|225440476| | XP_002272736.1 | -1.59 | 3.5 | minor CHO metabolism.others | Aldo-keto reductase family 4 member C9-like [ *Vitis vinifera* ] | 0 |
| ppa012210m | gi|224120044| | NP_177447.1 | -1.59 | 26.16 | misc.myrosinases-lectin-jacalin | Jacalin lectin family protein [ *Arabidopsis thaliana* ] | 0 |
| ppa002486m | gi|147827400| | NP_175661.1 | -1.59 | 30.11 | signalling.light | FRS6 (FAR1-related sequence 6); zinc ion binding [ *Arabidopsis thaliana* ] | 1.8 |
| ppa001406m | gi|224055166| | XP_002298427.1 | -1.59 | 35.2 | not assigned.unknown | Predicted protein [ *Populus trichocarpa* ] | 5E-121 |
| ppa006574m | gi|225457731| | XP_002510765.1 | -1.59 | 20.2.1 | stress.abiotic.heat | Chaperone protein dnaj, putative [ *Ricinus communis* ] | 4E-172 |
| ppa001303m | gi|225434275| | NP_179232.1 | -1.59 | 27.3.44 | RNA.regulation of transcription.Chromatin Remodeling Factors | Defective in rna-directed dna methylation 1; ATP binding / DNA binding / helicase/ nucleic acid binding [ *Arabidopsis thaliana* ] | 0 |
| ppa013751m | gi|224069060| | XP_002302890.1 | -1.59 | 35.2 | not assigned.unknown | Predicted protein [ *Populus trichocarpa* ] | 3.00E-113 |
| ppa007161m | gi|225443766| | XP_002522231.1 | -1.59 | 29.4 | protein.postranslational modification | Glycogen synthase kinase-3 beta, putative [ *Ricinus communis* ] | 0 |
| ppa005948m | gi|296084052| | XP_002513725.1 | -1.60 | 30.3 | signalling.calcium | Calmodulin binding protein, putative [ *Ricinus communis* ] | 0 |
| ppa014246m | gi|28624546| | AFK44299.1 | -1.60 | 35.2 | not assigned.unknown | Unknown [ *Lotus japonicus* ] | 5.00E-30 |
| ppa000586m | gi|297745504| | NP_563620.1 | -1.60 | 10.1.1.2 | cell wall.precursor synthesis.NDP sugar pyrophosphorylase.GDP fucose and fucokinase | GHMP kinase-related [ *Arabidopsis thaliana* ] | 9E-117 |
| ppa026297m | gi|297745649| | ABQ42034.1 | -1.60 | 35.2 | not assigned.unknown | Putative receptor-interacting protein [ *Sonneratia caseolaris* ] | 5E-140 |
| ppa007335m | gi|255564802| | XP_002523395.1 | -1.60 | 27.3.99 | RNA.regulation of transcription.unclassified | Zinc finger protein, putative [ *Ricinus communis* ] | 2E-95 |
| ppa010695m | gi|225457225| | NP_564059.1 | -1.61 | 26.4 | misc.beta 1,3 glucan hydrolases | PDCB3 (PLASMODESMATA CALLOSE-BINDING PROTEIN 3); callose binding / polysaccharide binding [ *Arabidopsis thaliana* ] | 7E-64 |
| ppa013124m | - | unkown | -1.61 | 35.2 | not assigned.unknown | Unkown | 0 |
| ppa007160m | gi|255559196| | EFN55157.1 | -1.61 | 35.2 | not assigned.unknown | Expressed protein [ *Chlorella variabilis* ] | 0 |
| ppa006135m | gi|255553023| | XP_002517554.1 | -1.61 | 30.11 | signalling.light | Signal transducer, putative [ *Ricinus communis* ] | 9E-174 |
| ppa011897m | gi|225438569| | XP_002306351.1 | -1.61 | 20 | stress | Stress enhanced protein 2 [ *Populus trichocarpa* ] | 3E-129 |
| ppa009235m | gi|225470670| | EGE72453.1 | -1.61 | 34.16 | transport.ABC transporters and multidrug resistance systems | ABC transporter ATP-binding protein [ *Propionibacterium acnes HL097PA1* ] | 0 |
| ppa008253m | gi|224100313| | XP_002517152.1 | -1.61 | 35.2 | not assigned.unknown | Catalytic, putative [ *Ricinus communis* ] | 0 |
| ppa001105m | gi|225439201| | XP_002525702.1 | -1.61 | 28.1 | DNA.synthesis/chromatin structure | 3'-5' exonuclease, putative   [ *Ricinus communis* ] | 0 |
| ppa004997m | gi|224094753| | ABC47859.1 | -1.61 | 35.2 | not assigned.unknown | Membrane protein-like protein [ *Glycine max* ] | 0 |
| ppa000506m | gi|225447543| | ACC60967.1 | -1.61 | 30.11 | signalling.light | Phytochrome C [ *Vitis vinifera* ] | 0 |
| ppa015639m | gi|255541858| | XP_002511993.1 | -1.61 | 31.3.1 | cell.cycle.peptidylprolyl isomerase | Peptidyl-prolyl cis-trans isomerase B, ppib, putative [ *Ricinus communis* ] | 0 |
| ppa013661m | gi|255566866| | XP_002524416.1 | -1.61 | 29.2.1.2.2.81 | protein.synthesis.ribosomal protein.eukaryotic.60S subunit.P1 | 60S acidic ribosomal protein P1, putative [ *Ricinus communis* ] | 1E-53 |
| ppa003964m | gi|255561576| | XP_002521798.1 | -1.61 | 29.3.4.99 | protein.targeting.secretory pathway.unspecified | Patellin-5, putative [ *Ricinus communis* ] | 7E-107 |
| ppa008364m | gi|297814634| | XP_002875200.1 | -1.62 | 35.2 | not assigned.unknown | Hypothetical protein ARALYDRAFT_484241 [ *Arabidopsis lyrata subsp. Lyrata* ] | 0 |
| ppa001599m | gi|225453336| | XP_002270305.1 | -1.62 | 29.1.18 | protein.aa activation.glutamine-tRNA ligase | Similar to Glutaminyl-trna synthetase [ *Vitis vinifera* ] | 4E-96 |
| ppa010049m | gi|225430832| | EOX91161.1 | -1.62 | 11.8 | lipid metabolism.'exotics'(steroids, squalene etc) | Quinone reductase family protein isoform 1 [ *Theobroma cacao* ] | 8.00E-140 |
| ppa008407m | gi|224106505| | XP_002314189.1 | -1.62 | 27.3.8 | RNA.regulation of transcription.C2C2(Zn) DOF zinc finger family | F-box family protein [ *Populus trichocarpa* ] | 0 |
| ppa006673m | gi|224140795| | XP_002525861.1 | -1.62 | 29.5 | protein.degradation | Caspase, putative [ *Ricinus communis* ] | 7E-20 |
| ppa004692m | gi|255585154| | XP_002533282.1 | -1.62 | 27.3.99 | RNA.regulation of transcription.unclassified | Zinc ion binding protein, putative [ *Ricinus communis* ] | 0 |
| ppa004039m | gi|225430796| | XP_002521582.1 | -1.62 | 27.2 | RNA.transcription | RNA polymerase II transcription factor, putative [ *Ricinus communis* ] | 0 |
| ppa009295m | gi|225440003| | XP_002519525.1 | -1.62 | 27.4 | RNA.RNA binding | Ribonucleoprotein, chloroplast, putative [ *Ricinus communis* ] | 2E-156 |
| ppa004252m | gi|225442231| | XP_002521368.1 | -1.62 | 29.4 | protein.postranslational modification | Protein phosphatase 2c, putative [ *Ricinus communis* ] | 5E-104 |
| ppa010730m | gi|225469006| | XP_002518568.1 | -1.62 | 27.3.6 | RNA.regulation of transcription.bHLH,Basic Helix-Loop-Helix family | DNA binding protein, putative [ *Ricinus communis* ] | 7E-177 |
| ppa011666m | gi|225426124| | ABF39005.1 | -1.62 | 35.2 | not assigned.unknown | Plant cell wall protein sltfr88 [ *Solanum lycopersicum* ] | 2E-19 |
| ppa002315m | gi|255547544| | XP_002514829.1 | -1.63 | 35.1 | not assigned.no ontology | Glucosamine-fructose-6-phosphate aminotransferase, putative [ *Ricinus communis* ] | 0 |
| ppa003173m | gi|224146182| | BAB09619.1 | -1.63 | 27.3.99 | RNA.regulation of transcription.unclassified | Mutator-like transposase-like protein [ *Arabidopsis thaliana* ] | 1E-176 |
| ppa024164m | gi|225428840| | XP_002885306.1 | -1.63 | 29.4.1.57 | protein.postranslational modification.kinase.receptor like cytoplasmatic kinase VII | Kinase family protein [ *Arabidopsis lyrata subsp. Lyrata* ] | 0 |
| ppa001690m | gi|255537029| | XP_002509581.1 | -1.63 | 29.2.4 | protein.synthesis.elongation | Translation elongation factor G, putative [ *Ricinus communis* ] | 0 |
| ppa012606m | - | unkown | -1.63 | 35.2 | not assigned.unknown | Unkown | 0 |
| ppa004232m | gi|5804835| | CAA04772.2 | -1.63 | 13.1.3.4.1 | amino acid metabolism.synthesis.aspartate family.methionine.cystathionine gamma-synthase | Cystathionine gamma synthase [ *Fragaria vesca* ] | 2E-142 |
| ppa001085m | gi|14589309|e | CAC43237.1 | -1.63 | 17.7.1.2 | hormone metabolism.jasmonate.synthesis-degradation.lipoxygenase | Lipoxygenase [ *Sesbania rostrata* ] | 0 |
| ppa007348m | gi|255542044| | XP_002512086.1 | -1.64 | 35.1 | not assigned.no ontology | SPFH domain-containing protein 2 precursor, putative [ *Ricinus communis* ] | 0 |
| ppa004839m | gi|224113247| | XP_002513320.1 | -1.64 | 17.3.1.2.7 | hormone metabolism.brassinosteroid.synthesis-degradation.sterols.DWF5 | 7-dehydrocholesterol reductase, putative [ *Ricinus communis* ] | 6E-13 |
| ppa004849m | gi|255560495| | XP_002866803.1 | -1.64 | 35.2 | not assigned.unknown | Hypothetical protein ARALYDRAFT_490610 [ *Arabidopsis lyrata subsp. Lyrata* ] | 0 |
| ppa009815m | gi|147819180| | XP_002529910.1 | -1.64 | 27.3.99 | RNA.regulation of transcription.unclassified | Zinc finger protein, putative [ *Ricinus communis* ] | 0 |
| ppa003200m | gi|225428572| | XP_002519105.1 | -1.64 | 29.5.11.4.2 | protein.degradation.ubiquitin.E3.RING | Protein ariadne-1, putative [ *Ricinus communis* ] | 2E-81 |
| ppa002637m | gi|225470467| | XP_003635518.1 | -1.64 | 29.4 | protein.postranslational modification | Mitogen-activated protein kinase kinase kinase ANP1-like [ *Vitis vinifera* ] | 0 |
| ppa005435m | gi|225455346| | NP_001061754.1 | -1.64 | 35.2 | not assigned.unknown | Os08g0400300 [ *Oryza sativa Japonica Group* ] | 0 |
| ppa006481m | gi|225426232| | ABG47411.1 | -1.64 | 2.2.2.6 | major CHO metabolism.degradation.starch.transporter | Maltose transporter [ *Malus x domestica* ] | 0 |
| ppa003047m | gi|119507461| | BAF42038.1 | -1.64 | 10.8.1 | cell wall.pectin*esterases.PME | Pectin methylesterase 1 [ *Pyrus communis* ] | 0 |
| ppa010516m | gi|225458354| | XP_002468331.1 | -1.64 | 35.2 | not assigned.unknown | Hypothetical protein SORBIDRAFT_01g043920 [ *Sorghum bicolor* ] | 0 |
| ppa010000m | gi|224106974| | XP_002519319.1 | -1.65 | 31.4 | cell.vesicle transport | Novel plant snare, putative [ *Ricinus communis* ] | 0 |
| ppa024367m | gi|297745646| | NP_181526.1 | -1.65 | 34.9 | transport.metabolite transporters at the mitochondrial membrane | Peroxisomal membrane protein (PMP36) [ *Arabidopsis thaliana* ] | 0 |
| ppa007338m | gi|225430700| | XP_002521548.1 | -1.65 | 30.5 | signalling.G-proteins | GTP-binding protein era, putative [ *Ricinus communis* ] | 5E-130 |
| ppa005076m | gi|117622284| | XP_002280863.2 | -1.65 | 23.2 | nucleotide metabolism.degradation | Nucleoside-triphosphatase [ *Vitis vinifera* ] | 0 |
| ppa001148m | gi|297739742| | EOY11201.1 | -1.65 | 27.3.67 | RNA.regulation of transcription.putative transcription regulator | Transcription regulator NOT2/NOT3/NOT5 family protein [ *Theobroma cacao* ] | 0 |
| ppa018863m | gi|224130512| | XP_002523373.1 | -1.65 | 35.1.41 | not assigned.no ontology.hydroxyproline rich proteins | Predicted protein [ *Populus trichocarpa* ] | 0 |
| ppa006497m | gi|225452418| | NP_196968.2 | -1.65 | 29.4 | protein.postranslational modification | SK13 (SHAGGY-LIKE KINASE 13); ATP binding / protein kinase/ protein serine/threonine kinase [ *Arabidopsis thaliana* ] | 0 |
| ppa000924m | gi|225439041| | XP_002264658.2 | -1.65 | 29.4.1.57 | protein.postranslational modification.kinase.receptor like cytoplasmatic kinase VII | Probable leucine-rich repeat receptor-like protein kinase At5g49770 [ *Vitis vinifera* ] | 0 |
| ppa013737m | gi|255578611| | XP_002874058.1 | -1.65 | 35.2 | not assigned.unknown | Hypothetical protein ARALYDRAFT_489079 [ *Arabidopsis lyrata subsp. Lyrata* ] | 0 |
| ppa009664m | gi|255550393| | XP_002516247.1 | -1.65 | 35.2 | not assigned.unknown | Ubiquinone biosynthesis protein COQ9, mitochondrial precursor, putative [ *Ricinus communis* ] | 1E-90 |
| ppa012466m | gi|224129408| | XP_002320579.1 | -1.65 | 35.2 | not assigned.unknown | Predicted protein [ *Populus trichocarpa* ] | 3.00E-115 |
| ppa016719m | gi|224077064| | XP_002305116.1 | -1.66 | 35.2 | not assigned.unknown | Predicted protein [ *Populus trichocarpa* ] | 0 |
| ppa004675m | gi|225457130| | ABF96807.1 | -1.66 | 30.3 | signalling.calcium | Calmodulin-binding protein, putative, expressed [ *Oryza sativa Japonica Group* ] | 3E-171 |
| ppa007744m | gi|77540212| | AAR86689.1 | -1.66 | 1.3.6 | PS.calvin cycle.aldolase | Fructose-bisphosphate aldolase [ *Glycine max* ] | 0 |
| ppa004603m | gi|224134228| | NP_850841.1 | -1.66 | 29.5.9 | protein.degradation.AAA type | AAA-type atpase family protein [ *Arabidopsis thaliana* ] | 0 |
| ppa001669m | gi|225459461| | XP_002513822.1 | -1.66 | 30.4.1 | signalling.phosphinositides.phosphatidylinositol-4-phosphate 5-kinase | Phosphatidylinositol-4-phosphate 5-kinase, putative [ *Ricinus communis* ] | 0 |
| ppa000156m | gi|225443998| | XP_002281070.1 | -1.66 | 34.16 | transport.ABC transporters and multidrug resistance systems | Similar to putative multidrug resistance-associated protein [ *Vitis vinifera* ] | 2E-121 |
| ppa003465m | gi|225451571| | XP_002274892.1 | -1.66 | 17.2.2 | hormone metabolism.auxin.signal transduction | F-box protein AUXIN SIGNALING F-BOX 3 [ *Vitis viniferafamily protein Populus trichocarpa* ] | 0.00E+00 |
| ppa005163m | gi|62857010| | BAD95890.1 | -1.67 | 29.4 | protein.postranslational modification | Ser/Thr protein kinase [ *Lotus japonicus* ] | 0 |
| ppa027113m | gi|255540813| | XP_002511471.1 | -1.67 | 27.3.5 | RNA.regulation of transcription.ARR | Two-component sensor protein histidine protein kinase, putative [ *Ricinus communis* ] | 0 |
| ppa020675m | gi|224142157| | AAW57535.1 | -1.67 | 30.5 | signalling.G-proteins | Pollen-specific kinase partner protein [ *Solanum lycopersicum* ] | 4E-27 |
| ppa000203m | gi|225436245| | XP_002319739.1 | -1.67 | 27.3.44 | RNA.regulation of transcription.Chromatin Remodeling Factors | Chromatin remodeling complex subunit [ *Populus trichocarpa* ] | 0 |
| ppa013750m | - | unkown | -1.67 | 35.2 | not assigned.unknown | Unkown | 0 |
| ppa025447m | gi|225434534| | XP_002276582.1 | -1.67 | 34.15 | transport.potassium | Potassium transporter 1-like [ *Vitis vinifera* ] | 0 |
| ppa023042m | gi|297743976| | XP_002515288.1 | -1.67 | 35.1 | not assigned.no ontology | WD-repeat protein, putative [ *Ricinus communis* ] | 0 |
| ppa019583m | gi|224157841| | AAB01563.1 | -1.67 | 35.2 | not assigned.unknown | Late embryogenesis abundant protein [ *Picea glauca* ] | 0 |
| ppa012582m | gi|296090223| | XP_002275929.1 | -1.67 | 28.99 | DNA.unspecified | PREDICTED: similar to tatd-related deoxyribonuclease family protein [ *Vitis vinifera* ] | 2E-39 |
| ppa007608m | gi|225448605| | XP_002278805.1 | -1.67 | 35.2 | not assigned.unknown | Uncharacterized protein At2g40430 [ *Vitis vinifera* ] | 0 |
| ppa002445m | gi|224104683| | NP_200884.2 | -1.67 | 35.2 | not assigned.unknown | 2-phosphoglycerate kinase-related [ *Arabidopsis thaliana* ] | 7E-27 |
| ppa005137m | gi|158427470| | ADD60242.1 | -1.67 | 1.3.13 | PS.calvin cycle.rubisco interacting | Alpha-form rubisco activase [ *Glycine max* ] | 0 |
| ppa007094m | gi|255568329| | XP_002525139.1 | -1.67 | 27.3.29 | RNA.regulation of transcription.TCP transcription factor family | Transcription factor, putative [ *Ricinus communis* ] | 0 |
| ppa004014m | gi|225424560| | XP_002515036.1 | -1.67 | 31.2 | cell.division | RCC1 and BTB domain-containing protein, putative [ *Ricinus communis* ] | 0 |
| ppa012940m | gi|18252327|g | ADQ91846.1 | -1.68 | 33.2 | development.late embryogenesis abundant | Late embryogenesis abundant protein group 7 protein [ *Arachis hypogaea* ] | 4.00E-67 |
| ppa004485m | gi|224135729| | XP_002511464.1 | -1.68 | 10.1 | cell wall.precursor synthesis | Mannose-1-phosphate guanyltransferase, putative [ *Ricinus communis* ] | 2E-128 |
| ppa003826m | gi|255565184| | XP_002523584.1 | -1.68 | 35.2 | not assigned.unknown | Splicing factor, arginine/serine-rich, putative [ *Ricinus communis* ] | 0 |
| ppa001847m | gi|255576268| | XP_002529027.1 | -1.68 | 11.9.3.3 | lipid metabolism.lipid degradation.lysophospholipases.glycerophosphodiester phosphodiesterase | Glycerophosphoryl diester phosphodiesterase, putative [ *Ricinus communis* ] | 7E-76 |
| ppa025532m | gi|255558578| | XP_004292721.1 | -1.69 | 34.3 | transport.amino acids | Amino-acid permease BAT1 homolog [ *Fragaria vesca subsp. Vesca* ] | 0 |
| ppa013239m | gi|224146180| | ABP38063.1 | -1.69 | 11.1.12 | lipid metabolism.FA synthesis and FA elongation.ACP protein | Acyl carrier protein [ *Jatropha curcas* ] | 0 |
| ppa005877m | gi|255585841| | XP_002533598.1 | -1.69 | 29.5.11.3 | protein.degradation.ubiquitin.E2 | Ubiquitin-protein ligase, putative [ *Ricinus communis* ] | 1E-138 |
| ppa015117m | gi|225423660| | XP_002517233.1 | -1.69 | 31.1 | cell.organisation | Ankyrin repeat-containing protein, putative [ *Ricinus communis* ] | 0 |
| ppa000337m | gi|255574744| | XP_002528280.1 | -1.69 | 29.3.3 | protein.targeting.chloroplast | Protein translocase, putative [ *Ricinus communis* ] | 0 |
| ppa004184m | gi|225452021| | XP_002868773.1 | -1.69 | 26.2 | misc.UDP glucosyl and glucoronyl transferases | ALG6, ALG8 glycosyltransferase family protein [ *Arabidopsis lyrata subsp. Lyrata* ] | 0 |
| ppa010606m | gi|225450155| | AAM61552.1 | -1.69 | 1.1.1.2 | PS.lightreaction.photosystem II.PSII polypeptide subunits | Thylakoid lumen protein, chloroplast precursor [ *Arabidopsis thaliana* ] | 0 |
| ppa006145m | gi|3914449| | NP_175778.1 | -1.69 | 29.5.11.20 | protein.degradation.ubiquitin.proteasom | RPT1A (REGULATORY PARTICLE TRIPLE-A 1A); atpase [ *Arabidopsis thaliana* ] | 5E-80 |
| ppa010440m | gi|255645309| | AAC17046.1 | -1.69 | 35.2 | not assigned.unknown | EST gb|N65759 comes from this gene [ *Arabidopsis thaliana* ] | 4E-133 |
| ppa010508m | gi|224121954| | XP_002511214.1 | -1.70 | 23.4.1 | nucleotide metabolism.phosphotransfer and pyrophosphatases.adenylate kinase | Adenylate kinase 1, putative [ *Ricinus communis* ] | 2E-71 |
| ppa002582m | gi|255551261| | XP_002516677.1 | -1.70 | 35.1.5 | not assigned.no ontology.pentatricopeptide (PPR) repeat-containing protein | Pentatricopeptide repeat-containing protein, putative [ *Ricinus communis* ] | 0 |
| ppa012295m | gi|225426989| | XP_002528924.1 | -1.70 | 20.2.1 | stress.abiotic.heat | Chaperone protein dnaj, putative [ *Ricinus communis* ] | 0 |
| ppa007219m | gi|255579745| | XP_002530711.1 | -1.70 | 31.1 | cell.organisation | Actin, putative [ *Ricinus communis* ] | 4E-76 |
| ppa014737m | gi|225431938| | NP_176041.2 | -1.70 | 27.3.99 | RNA.regulation of transcription.unclassified | PAPA-1-like family protein / zinc finger (HIT type) family protein [ *Arabidopsis thaliana* ] | 0 |
| ppa005038m | gi|152968456| | ABS50251.1 | -1.70 | 27.3.6 | RNA.regulation of transcription.bHLH,Basic Helix-Loop-Helix family | BHLH transcriptional factor [ *Malus x domestica* ] | 0 |
| ppa007941m | gi|296088554| | XP_002274450.2 | -1.70 | 33.99 | development.unspecified | NAC domain-containing protein 78-like [ *Vitis vinifera* ] | 8.00E-180 |
| ppa016040m | gi|255583914| | XP_002532705.1 | -1.70 | 35.2 | not assigned.unknown | Conserved hypothetical protein [ *Ricinus communis* ] | 4E-87 |
| ppa003536m | gi|224102909| | EOY33146.1 | -1.70 | 29.5.11.4.3.2 | protein.degradation.ubiquitin.E3.SCF.FBOX | F-box protein 2 [ *Theobroma cacao* ] | 0 |
| ppa001964m | gi|224053571| | XP_002512672.1 | -1.71 | 29.1.14 | protein.aa activation.glycine-tRNA ligase | Glycyl-trna synthetase, putative [ *Ricinus communis* ] | 0 |
| ppa013033m | gi|225427991| | ABD66507.1 | -1.71 | 31.1 | cell.organisation | Actin depolymerizing factor 5 [ *Gossypium hirsutum* ] | 7E-172 |
| ppa007784m | gi|224127576| | OY04350.1 | -1.71 | 30.5 | signalling.G-proteins | Plant adhesion molecule 1 isoform 3 [ *Theobroma cacao* ] | 0 |
| ppa005159m | gi|225443472| | XP_002513291.1 | -1.71 | 27.3.20 | RNA.regulation of transcription.G2-like transcription factor family, GARP | Transcription factor, putative [ *Ricinus communis* ] | 6E-122 |
| ppa004934m | gi|255563606| | XP_002522805.1 | -1.71 | 35.2 | not assigned.unknown | Adenosylhomocysteinase, putative [ *Ricinus communis* ] | 1E-73 |
| ppa010093m | gi|118487360| | XP_002521576.1 | -1.71 | 1.1.1.2 | PS.lightreaction.photosystem II.PSII polypeptide subunits | Oxygen-evolving enhancer protein 2, chloroplast precursor, putative [ *Ricinus communis* ] | 0 |
| ppa006521m | gi|255574095| | XP_002527963.1 | -1.71 | 27.3.11 | RNA.regulation of transcription.C2H2 zinc finger family | Transcription factor, putative [ *Ricinus communis* ] | 0 |
| ppa023236m | gi|255580627| | NP_001086389.1 | -1.71 | 35.2 | not assigned.unknown | Methyltransferase like 23 [ *Xenopus laevis* ] | 3E-145 |
| ppa010218m | gi|225434492| | NP_563779.1 | -1.71 | 35.1 | not assigned.no ontology | Gamma interferon responsive lysosomal thiol reductase family protein / GILT family protein [ *Arabidopsis thaliana* ] | 0 |
| ppa004439m | gi|224108067| | XP_002517216.1 | -1.72 | 34.2 | transport.sugars | Sugar transporter, putative [ *Ricinus communis* ] | 4E-69 |
| ppa016276m | gi|225443276| | XP_002525004.1 | -1.72 | 30.2.11 | signalling.receptor kinases.leucine rich repeat XI | Receptor protein kinase CLAVATA1 precursor, putative [ *Ricinus communis* ] | 2E-76 |
| ppa005372m | gi|225465708| | XP_002889494.1 | -1.72 | 29.2.3 | protein.synthesis.initiation | EIF2 gamma [ *Arabidopsis lyrata subsp. Lyrata* ] | 1E-142 |
| ppa003591m | gi|225462064| | XP_002530618.1 | -1.72 | 27.3.99 | RNA.regulation of transcription.unclassified | TFIIH basal transcription factor complex subunit, putative [ *Ricinus communis* ] | 3E-36 |
| ppa015125m | gi|224113557| | XP_002332553.1 | -1.72 | 20.1 | stress.biotic | CC-NBS-LRR resistance protein [ *Populus trichocarpa* ] | 0 |
| ppa003995m | gi|297736353| | ABF94794.1 | -1.72 | 17.3.1.2.99 | hormone metabolism.brassinosteroid.synthesis-degradation.sterols.other | Squalene monooxygenase, putative, expressed [ *Oryza sativa Japonica Group* ] | 8E-176 |
| ppa009657m | gi|225433387| | XP_002518167.1 | -1.73 | 35.2 | not assigned.unknown | Hydroxyethylthiazole kinase, putative [ *Ricinus communis* ] | 0 |
| ppa006544m | gi|254596582| | ACT75571.1 | -1.73 | 16.1.3.2 | secondary metabolism.isoprenoids.tocopherol biosynthesis.homogentisate phytyltransferase | Homogentisate phytyltransferase [ *Malus x domestica* ] | 1E-130 |
| ppa010091m | gi|225445216| | NP_565464.2 | -1.73 | 35.1 | not assigned.no ontology | COV1 (CONTINUOUS VASCULAR RING) [ *Arabidopsis thaliana* ] | 6E-116 |
| ppa019110m | gi|255560485| | XP_002521257.1 | -1.73 | 35.2 | not assigned.unknown | Conserved hypothetical protein [ *Ricinus communis* ] | 4E-112 |
| ppa005373m | gi|255547297| | XP_002514706.1 | -1.73 | 26.7 | misc.oxidases - copper, flavone etc | Dimethylaniline monooxygenase, putative [ *Ricinus communis* ] | 2E-51 |
| ppa013784m | - | unkown | -1.73 | 35.2 | not assigned.unknown | Unkown | 0 |
| ppa007779m | gi|225464748| | XP_002527722.1 | -1.74 | 34.12 | transport.metal | Zinc/iron transporter, putative [ *Ricinus communis* ] | 0 |
| ppa008121m | gi|297744466| | EOX98921.1 | -1.74 | 34.2 | transport.sugars | Nucleotide-sugar transporter family protein isoform 1 [ *Theobroma cacao* ] | 0 |
| ppa007682m | gi|225427183| | XP_002527667.1 | -1.74 | 29.5.11.4.2 | protein.degradation.ubiquitin.E3.RING | Ring finger protein, putative [ *Ricinus communis* ] | 0 |
| ppa002642m | gi|224055697| | NP_195674.2 | -1.74 | 20.2 | stress.abiotic | GGT1 (gamma-glutamyl transpeptidase 1); gamma-glutamyltransferase/ glutathione gamma-glutamylcysteinyltransferase [ *Arabidopsis thaliana* ] | 0 |
| ppa003784m | gi|225453845| | NP_566051.1 | -1.74 | 30.3 | signalling.calcium | Calcineurin B subunit-related [ *Arabidopsis thaliana* ] | 0 |
| ppa008026m | gi|225454510| | XP_002281449.1 | -1.74 | 35.2 | not assigned.unknown | Hypothetical protein [ *Vitis vinifera* ] | 0 |
| ppa011951m | gi|255630681| | XP_002310066.1 | -1.74 | 21.1 | redox.thioredoxin | Thioredoxin x [ *Populus trichocarpa* ] | 0 |
| ppa012994m | gi|255632023| | ACJ02353.1 | -1.74 | 29.2.1.2.2.527 | protein.synthesis.ribosomal protein.eukaryotic.60S subunit.L27A | 60S ribosomal protein L27A [ *Vernicia fordii* ] | 0 |
| ppa005518m | gi|255554747| | NP_001043886.1 | -1.74 | 1.2.7 | PS.photorespiration.glycerate kinase | Phosphoribulokinase/uridine kinase-like [ *Oryza sativa Japonica Group* ] | 0 |
| ppa000178m | gi|255586801| | EOY18833.1 | -1.75 | 27.3.67 | RNA.regulation of transcription.putative transcription regulator | Kow domain-containing transcription factor 1, putative [ *Theobroma cacao* ] | 0 |
| ppa016117m | gi|225423905| | ACO87667.1 | -1.75 | 35.2 | not assigned.unknown | Zn-dependent hydrolases of the beta-lactamase fold [ *Brachypodium sylvaticum* ] | 0 |
| ppa001811m | gi|255558560| | EOY16450.1 | -1.75 | 29.4.1.57 | protein.postranslational modification.kinase.receptor like cytoplasmatic kinase VII | Kinase protein with adenine nucleotide alpha hydrolases-like domain [ *Theobroma cacao* ] | 0 |
| ppa002618m | gi|255538240| | XP_002510185.1 | -1.75 | 27.1 | RNA.processing | Polyadenylate-binding protein, putative [ *Ricinus communis* ] | 0 |
| ppa007018m | gi|255549686| | AAD40142.1 | -1.75 | 35.2 | not assigned.unknown | Contains similarity to several Arabidopsis thaliana hypothetical proteins including GB:U95973 and GB:AC002392 [ *Arabidopsis thaliana* ] | 0 |
| ppa000753m | gi|255561929| | XP_002521973.1 | -1.75 | 27.3.26 | RNA.regulation of transcription.MYB-related transcription factor family | Cell division control protein, putative [ *Ricinus communis* ] | 0 |
| ppa000256m | gi|296081973| | XP_002514350.1 | -1.76 | 34.16 | transport.ABC transporters and multidrug resistance systems | ATP-binding cassette transporter, putative [ *Ricinus communis* ] | 0 |
| ppa010886m | gi|255536745| | EOY28175.1 | -1.76 | 20.2.3 | stress.abiotic.drought/salt | Drought-responsive family protein [ *Theobroma cacao* ] | 1.00E-148 |
| ppa003497m | gi|255558446| | NP_197952.2 | -1.76 | 27.1.19 | RNA.processing.ribonucleases | Exonuclease family protein [ *Arabidopsis thaliana* ] | 0 |
| ppa001338m | gi|225461381| | XP_002534244.1 | -1.76 | 35.1 | not assigned.no ontology | WD-repeat protein, putative [ *Ricinus communis* ] | 2E-132 |
| ppa007127m | gi|255565007| | XP_002523496.1 | -1.76 | 35.2 | not assigned.unknown | Conserved hypothetical protein [ *Ricinus communis* ] | 2E-28 |
| ppa007802m | gi|225453883| | ABN08246.1 | -1.76 | 34.11 | transport.NDP-sugars at the ER | Solute carrier family 35 member B3, related [ *Medicago truncatula* ] | 8E-54 |
| ppa003741m | gi|297736821| | NP_564524.1 | -1.76 | 35.1.41 | not assigned.no ontology.hydroxyproline rich proteins | Hydroxyproline-rich glycoprotein family protein [ *Arabidopsis thaliana* ] | 0 |
| ppa007129m | gi|225451873| | XP_002514591.1 | -1.76 | 27.3.99 | RNA.regulation of transcription.unclassified | Zinc finger protein, putative [ *Ricinus communis* ] | 0 |
| ppa002310m | gi|255583467| | EOY21285.1 | -1.76 | 31.4 | cell.vesicle transport | Golgin candidate 2, putative isoform 1 [ *Theobroma cacao* ] | 0 |
| ppa020262m | gi|255547207| | XP_002866417.1 | -1.76 | 27.3.99 | RNA.regulation of transcription.unclassified | Zinc ion binding protein [ *Arabidopsis lyrata subsp. Lyrata* ] | 0 |
| ppa007166m | gi|255553691| | XP_002517886.1 | -1.76 | 34.9 | transport.metabolite transporters at the mitochondrial membrane | ADP,ATP carrier protein, putative [ *Ricinus communis* ] | 3E-81 |
| ppa009843m | gi|225439305| | XP_002524085.1 | -1.77 | 29.2.1.1.1.2.22 | protein.synthesis.ribosomal protein.prokaryotic.chloroplast.50S subunit.L22 | 50S ribosomal protein L22, putative [ *Ricinus communis* ] | 0 |
| ppa008090m | gi|255636059| | AAT36331.1 | -1.77 | 26.8 | misc.nitrilases, *nitrile lyases, berberine bridge enzymes, reticuline oxidases, troponine reductases | Nitrilase 4A [ *Lupinus angustifolius* ] | 1E-55 |
| ppa008172m | gi|298204851| | ADX60279.1 | -1.77 | 27.3.6 | RNA.regulation of transcription.bHLH,Basic Helix-Loop-Helix family | BHLH transcription factor [ *Oryza sativa Japonica Group* ] | 0 |
| ppa003206m | gi|66816747| | AAY56750.1 | -1.77 | 27.3.21 | RNA.regulation of transcription.GRAS transcription factor family | DELLA protein [ *Malus x domestica* ] | 0 |
| ppa019834m | gi|225424831| | XP_002318534.1 | -1.77 | 20.1.7 | stress.biotic.PR-proteins | Tir-nbs-lrr resistance protein [ *Populus trichocarpa* ] | 0 |
| ppa003334m | gi|224104295| | NP_565685.1 | -1.77 | 35.2 | not assigned.unknown | Binding [ *Arabidopsis thaliana* ] | 0 |
| ppa014179m | gi|116779464| | ABK21295.1 | -1.77 | 35.2 | not assigned.unknown | Unknown [ *Picea sitchensis* ] | 2.00E-102 |
| ppa011667m | gi|255552287| | XP_002517188.1 | -1.77 | 21.5 | redox.peroxiredoxin | Peroxiredoxin, putative [ *Ricinus communis* ] | 0 |
| ppa010390m | gi|298204756| | AAF19758.1 | -1.78 | 35.1 | not assigned.no ontology | Contains similarity to gi|629253 lmbw protein from Streptomyces lincolnensis [ *Arabidopsis thaliana* ] | 1E-161 |
| ppa002048m | gi|255545686| | EOY16028.1 | -1.78 | 35.1.5 | not assigned.no ontology.pentatricopeptide (PPR) repeat-containing protein | Tetratricopeptide repeat-containing protein, putative isoform 1 [ *Theobroma cacao* ] | 0 |
| ppa025630m | gi|255628091| | NP_001150376.1 | -1.78 | 35.2 | not assigned.unknown | Enzyme of the cupin superfamily [ *Zea mays* ] | 1E-35 |
| ppa004973m | gi|147865531| | XP_002519849.1 | -1.78 | 27.3.99 | RNA.regulation of transcription.unclassified | Transcription factor IWS1, putative [ *Ricinus communis* ] | 5E-139 |
| ppa001971m | gi|224072232| | XP_002518851.1 | -1.78 | 10.2.1 | cell wall.cellulose synthesis.cellulose synthase | Transferase, putative [ *Ricinus communis* ] | 4E-44 |
| ppa010782m | gi|255625831| | NP_201026.1 | -1.78 | 33.99 | development.unspecified | Embryo-specific protein-related [ *Arabidopsis thaliana* ] | 5E-79 |
| ppa013301m | gi|225455256| | XP_002321970.1 | -1.78 | 17.2.3 | hormone metabolism.auxin.induced-regulated-responsive-activated | SAUR family protein [ *Populus trichocarpa* ] | 4E-148 |
| ppa009475m | gi|255647110| | XP_002530636.1 | -1.78 | 26.7 | misc.oxidases - copper, flavone etc | Prolyl 4-hydroxylase alpha subunit, putative [ *Ricinus communis* ] | 5E-180 |
| ppa007556m | gi|118483602| | NP_187476.1 | -1.78 | 35.2 | not assigned.unknown | Alphavirus core protein family [ *Arabidopsis thaliana* ] | 2E-84 |
| ppa007030m | gi|296090290| | ABD28641.1 | -1.79 | 31.4 | cell.vesicle transport | Oxysterol-binding protein [ *Medicago truncatula* ] | 3E-21 |
| ppa023587m | gi|225438279| | XP_002325278.1 | -1.79 | 27.3.62 | RNA.regulation of transcription.Nucleosome/chromatin assembly factor group | Nucleosome/chromatin assembly factor group [ *Populus trichocarpa* ] | 5E-102 |
| ppa000807m | gi|225443588| | EOY28149.1 | -1.79 | 30.5 | signalling.G-proteins | Guanylate-binding family protein isoform 1 [ *Theobroma cacao* ] | 0 |
| ppa006148m | gi|225454014| | NP_191922.1 | -1.79 | 10.1.6 | cell wall.precursor synthesis.GAE | GAE3 (udp-d-glucuronate 4-epimerase 3); UDP-glucuronate 4-epimerase/ catalytic [ *Arabidopsis thaliana* ] | 0 |
| ppa009124m | gi|225451126| | NP_566244.1 | -1.79 | 35.1 | not assigned.no ontology | Transmembrane protein, putative [ *Arabidopsis thaliana* ] | 2E-113 |
| ppa006512m | gi|225444472| | ADN34064.1 | -1.79 | 13.2.5.2 | amino acid metabolism.degradation.serine-glycine-cysteine group.glycine | Aminomethyltransferase [ *Cucumis melo subsp. Melo* ] | 0 |
| ppa016951m | gi|255550018| | XP_002516060.1 | -1.79 | 34.9 | transport.metabolite transporters at the mitochondrial membrane | Mitochondrial carnitine/acylcarnitine carrier protein, putative [ *Ricinus communis* ] | 2E-123 |
| ppa005092m | gi|224091092| | XP_002531313.1 | -1.80 | 13.1.1.3.11 | amino acid metabolism.synthesis.central amino acid metabolism.alanine.alanine-glyoxylate aminotransferase | Alanine-glyoxylate aminotransferase, putative [ *Ricinus communis* ] | 0 |
| ppa001578m | gi|225427387| | XP_002510483.1 | -1.80 | 20.2.1 | stress.abiotic.heat | Heat shock protein binding protein, putative [ *Ricinus communis* ] | 0 |
| ppa010892m | gi|225443508| | NP_564316.1 | -1.80 | 23.3.3 | nucleotide metabolism.salvage.NUDIX hydrolases | ATNUDX15 (arabidopsis thaliana nudix hydrolase homolog 15); hydrolase [ *Arabidopsis thaliana* ] | 0 |
| ppa010346m | gi|225446445| | XP_002517864.1 | -1.80 | 27.1.1 | RNA.processing.splicing | 20 kd nuclear cap binding protein, putative [ *Ricinus communis* ] | 5E-57 |
| ppa001526m | gi|27261142| | AAN87547.1 | -1.80 | 23.5.4 | nucleotide metabolism.deoxynucleotide metabolism.ribonucleoside-diphosphate reductase | Ribonucleotide reductase large subunit A [ *Glycine max* ] | 8E-142 |
| ppa016246m | gi|255636754| | XP_002526282.1 | -1.80 | 20.2.3 | stress.abiotic.drought/salt | Phosphopentothenoylcysteine decarboxylase, putative [ *Ricinus communis* ] | 0 |
| ppa023993m | gi|224096774| | XP_002522044.1 | -1.80 | 30.2.17 | signalling.receptor kinases.DUF 26 | Kinase, putative [ *Ricinus communis* ] | 0 |
| ppa002688m | gi|255545206| | XP_002513664.1 | -1.80 | 27.3.21 | RNA.regulation of transcription.GRAS transcription factor family | DELLA protein GAI, putative [ *Ricinus communis* ] | 8E-102 |
| ppa002940m | gi|225424281| | XP_002527800.1 | -1.81 | 4.2.2 | glycolysis.plastid branch.phosphoglucomutase (PGM) | Phosphoglucomutase, putative [ *Ricinus communis* ] | 0 |
| ppa010633m | gi|225448890| | XP_002270872.1 | -1.81 | 35.2 | not assigned.unknown | Hypothetical protein [ *Vitis vinifera* ] | 0 |
| ppa012243m | gi|225424977| | NP_001148446.1 | -1.81 | 35.2 | not assigned.unknown | Cp protein [ *Zea mays* ] | 0 |
| ppa010480m | gi|255568563| | XP_002525255.1 | -1.82 | 27.3.37 | RNA.regulation of transcription.AS2,Lateral Organ Boundaries Gene Family | LOB domain-containing protein, putative [ *Ricinus communis* ] | 0 |
| ppa004372m | gi|293792354| | ADE61678.1 | -1.82 | 26.1 | misc.cytochrome P450 | Ent-kaurene oxidase [ *Pyrus pyrifolia* ] | 4E-146 |
| ppa000986m | gi|224141079| | NP_186862.2 | -1.82 | 29.4.1.57 | protein.postranslational modification.kinase.receptor like cytoplasmatic kinase VII | RPK2 (RECEPTOR-LIKE PROTEIN KINASE 2); ATP binding / kinase/ protein serine/threonine kinase [ *Arabidopsis thaliana* ] | 0 |
| ppa006599m | gi|225470589| | XP_002527916.1 | -1.82 | 23.1.3 | nucleotide metabolism.synthesis.PRS-PP | Ribose-phosphate pyrophosphokinase, putative [ *Ricinus communis* ] | 0 |
| ppa002524m | gi|255555735| | XP_002518903.1 | -1.82 | 34.13 | transport.peptides and oligopeptides | Oligopeptide transporter, putative [ *Ricinus communis* ] | 0 |
| ppa006876m | gi|147767812| | XP_002518506.1 | -1.83 | 35.1.5 | not assigned.no ontology.pentatricopeptide (PPR) repeat-containing protein | Pentatricopeptide repeat-containing protein, putative [ *Ricinus communis* ] | 0 |
| ppa000721m | gi|255564168| | XP_002523081.1 | -1.83 | 28.1 | DNA.synthesis/chromatin structure | ATP-dependent RNA helicase, putative [ *Ricinus communis* ] | 4E-137 |
| ppa004655m | gi|255585488| | XP_002533436.1 | -1.83 | 35.2 | not assigned.unknown | Glycosyltransferase, putative [ *Ricinus communis* ] | 2E-165 |
| ppa001645m | gi|75120874| | ABA55727.1 | -1.83 | 11.10.2 | lipid metabolism.glycolipid synthesis.DGDG synthase | Digalactosyldiacylglycerol synthase 1 [ *Vigna unguiculata* ] | 0 |
| ppa001027m | gi|255583509| | NP_187930.2 | -1.83 | 35.1 | not assigned.no ontology | Protein binding [ *Arabidopsis thaliana* ] | 1E-08 |
| ppa000599m | gi|225445148| | XP_002514253.1 | -1.83 | 29.4 | protein.postranslational modification | Protein phosphatase 2c, putative [ *Ricinus communis* ] | 9E-37 |
| ppa010167m | gi|225465959| | XP_002299213.1 | -1.83 | 29.5.11.4.3.2 | protein.degradation.ubiquitin.E3.SCF.FBOX | F-box family protein [ *Populus trichocarpa* ] | 6E-128 |
| ppa005309m | gi|147791134| | XP_002528377.1 | -1.83 | 27.4 | RNA.RNA binding | Heterogeneous nuclear ribonucleoprotein 27C, putative [ *Ricinus communis* ] | 7E-107 |
| ppa011701m | gi|224141003| | NP_568568.1 | -1.83 | 30.3 | signalling.calcium | Calcium-binding EF hand family protein [ *Arabidopsis thaliana* ] | 0 |
| ppa025310m | gi|255578414| | XP_004499788.1 | -1.84 | 20.1.7 | stress.biotic.PR-proteins | TMV resistance protein N-like [ *Cicer arietinum* ] | 0 |
| ppa008198m | gi|225434879| | AAK73147.1 | -1.84 | 29.5.11.4.2 | protein.degradation.ubiquitin.E3.RING | Putative RING-H2 finger protein [ *Oryza sativa* ] | 6E-84 |
| ppa016016m | gi|217074282| | XP_004498408.1 | -1.84 | 29.4 | protein.postranslational modification | Probable protein phosphatase 2C 51-like [ *Cicer arietinum* ] | 0 |
| ppa000011m | gi|89357137| | ABD72304.1 | -1.84 | 35.2 | not assigned.unknown | Apple fruit acidity-related protein [ *Malus x domestica* ] | 0 |
| ppa012691m | gi|259123931| | ACV93248.1 | -1.84 | 20.2.1 | stress.abiotic.heat | CI small heat shock protein 1 [ *Prunus salicina* ] | 1E-144 |
| ppa009983m | gi|255550842| | XP_002516469.1 | -1.84 | 35.2 | not assigned.unknown | Conserved hypothetical protein [ *Ricinus communis* ] | 0 |
| ppa007368m | gi|157965847| | ABW06960.1 | -1.85 | 16.1.1.10 | secondary metabolism.isoprenoids.non-mevalonate pathway.geranylgeranyl pyrophosphate synthase | Geranylgeranyl diphosphate synthase [ *Corylus avellana* ] | 0 |
| ppa005463m | gi|18417054| | ABB47893.2 | -1.85 | 35.2 | not assigned.unknown | Major facilitator superfamily protein, expressed [ *Oryza sativa Japonica Group* ] | 0 |
| ppa000164m | gi|297741709| | NP_566903.1 | -1.85 | 27.3.52 | RNA.regulation of transcription.Global transcription factor group | Haloacid dehalogenase-like hydrolase family protein [ *Arabidopsis thaliana* ] | 0 |
| ppa008755m | gi|224136009| | XP_002511268.1 | -1.85 | 35.1 | not assigned.no ontology | 2-deoxyglucose-6-phosphate phosphatase, putative [ *Ricinus communis* ] | 0 |
| ppa007820m | gi|224141905| | XP_002518411.1 | -1.86 | 1.1.40 | PS.lightreaction.cyclic electron flow-chlororespiration | Alternative oxidase 4, chloroplast precursor, putative [ *Ricinus communis* ] | 0 |
| ppa004451m | gi|225452849| | ACU80551.1 | -1.86 | 27.3.8 | RNA.regulation of transcription.C2C2(Zn) DOF zinc finger family | Dof3 protein [ *Jatropha curcas* ] | 0 |
| ppa006417m | gi|255583798| | NP_188473.1 | -1.86 | 35.2 | not assigned.unknown | Atalmt9 (aluminum-activated malate transporter 9); anion channel [ *Arabidopsis thaliana* ] | 7E-112 |
| ppa006257m | gi|255567172| | XP_002524567.1 | -1.86 | 31.2 | cell.division | Cell cycle regulatory protein, putative [ *Ricinus communis* ] | 5E-90 |
| ppa016003m | gi|225437706| | XP_002515207.1 | -1.86 | 30.5 | signalling.G-proteins | Nucleotide binding protein, putative [ *Ricinus communis* ] | 0 |
| ppa003768m | gi|224121366| | XP_002330809.1 | -1.86 | 35.2 | not assigned.unknown | Predicted protein [ *Populus trichocarpa* ] | 2E-114 |
| ppa001694m | gi|255558846| | XP_002520446.1 | -1.86 | 11.8.1.1 | lipid metabolism.'exotics' (steroids, squalene etc).sphingolipids.ceramidase | Ceramidase, putative [ *Ricinus communis* ] | 0 |
| ppa007521m | gi|255541482| | XP_002511805.1 | -1.87 | 29.5.11.4.2 | protein.degradation.ubiquitin.E3.RING | Cleavage and polyadenylation specificity factor, putative [ *Ricinus communis* ] | 1E-130 |
| ppa017158m | gi|224080277| | EOX98539.1 | -1.87 | 30.11 | signalling.light | NAD(P)-binding Rossmann-fold superfamily protein isoform 1 [ *Theobroma cacao* ] | 0 |
| ppa006691m | gi|255648020| | NP_568131.1 | -1.87 | 29.5.11.4.3.2 | protein.degradation.ubiquitin.E3.SCF.FBOX | F-box family protein [ *Arabidopsis thaliana* ] | 5E-144 |
| ppa009392m | gi|86611379|g | ABD14373.1 | -1.87 | 29.4 | protein.postranslational modification | Cyclin-dependent kinase type A [ *Prunus dulcis* ] | 0 |
| ppa005161m | gi|60280217| | AAX16493.1 | -1.87 | 26.2 | misc.UDP glucosyl and glucoronyl transferases | UDP-glucose:flavonoid 7-O-glucosyltransferase [ *Malus x domestica* ] | 0 |
| ppa006295m | gi|225435516| | XP_002513457.1 | -1.88 | 27.3.6 | RNA.regulation of transcription.bHLH,Basic Helix-Loop-Helix family | DNA binding protein, putative [ *Ricinus communis* ] | 2E-123 |
| ppa002537m | gi|225468694| | XP_002509994.1 | -1.88 | 29.5.11.4.2 | protein.degradation.ubiquitin.E3.RING | Protein binding protein, putative [ *Ricinus communis* ] | 2E-180 |
| ppa002474m | gi|225458352| | XP_002510352.1 | -1.88 | 35.1 | not assigned.no ontology | Heat shock protein binding protein, putative [ *Ricinus communis* ] | 0 |
| ppa007076m | gi|302144000| | XP_002521312.1 | -1.89 | 27.3.27 | RNA.regulation of transcription.NAC domain transcription factor family | Transcription factor, putative [ *Ricinus communis* ] | 3E-156 |
| ppa005598m | gi|224098511| | ABA86964.1 | -1.89 | 1.3.4 | PS.calvin cycle.GAP | Glyceraldehyde-3-phosphate dehydrogenase B subunit [ *Glycine max* ] | 4E-57 |
| ppa007562m | gi|225457423| | ABK59040.1 | -1.89 | 27.3.25 | RNA.regulation of transcription.MYB domain transcription factor family | R2r3 Myb30 transcription factor [ *Vitis vinifera* ] | 3E-98 |
| ppa013183m | gi|145334271| | NP_001078516.1 | -1.89 | 28.1.3.2.3 | DNA.synthesis/chromatin structure.histone.core.H3 | Histone H3.2 [ *Arabidopsis thaliana* ] | 7.00E-114 |
| ppa002581m | gi|255561957| | XP_002521987.1 | -1.89 | 31.1 | cell.organisation | Protein binding protein, putative [ *Ricinus communis* ] | 2E-88 |
| ppa023464m | - | unkown | -1.89 | 35.2 | not assigned.unknown | Unkown | 0 |
| ppa009932m | gi|225444782| | ACF06499.1 | -1.90 | 29.2.1.2.1.3 | protein.synthesis.ribosomal protein.eukaryotic.40S subunit.S3 | Ribosomal protein [ *Elaeis guineensis* ] | 9E-124 |
| ppa012792m | gi|255557991| | XP_002283174.1 | -1.90 | 35.2 | not assigned.unknown | Uncharacterized protein LOC100248460 [ *Vitis vinifera* ] | 7.00E-123 |
| ppa002908m | gi|224095037| | XP_002525281.1 | -1.90 | 26.24 | misc.GCN5-related N-acetyltransferase | N-acetyltransferase, putative [ *Ricinus communis* ] | 2E-112 |
| ppa011940m | gi|255581048| | XP_002531340.1 | -1.90 | 35.1 | not assigned.no ontology | Frataxin, mitochondrial precursor, putative [ *Ricinus communis* ] | 0 |
| ppa012252m | gi|225429848| | XP_002527994.1 | -1.90 | 20.1 | stress.biotic | Protein AIG2, putative [ *Ricinus communis* ] | 0 |
| ppa014118m | - | unkown | -1.90 | 35.2 | not assigned.unknown | Unkown | 0 |
| ppa000485m | gi|225465133| | XP_002517570.1 | -1.91 | 29.5.9 | protein.degradation.AAA type | Peroxisome biogenesis factor, putative [ *Ricinus communis* ] | 0 |
| ppa013200m | gi|145328714| | NP_001077892.1 | -1.91 | 35.2 | not assigned.unknown | Uncharacterized protein [ *Arabidopsis thaliana* ] | 1E-48 |
| ppa017098m | gi|297733618| | EOY22383.1 | -1.91 | 35.2 | not assigned.unknown | Cysteine proteinases superfamily protein, putative isoform 1 [ *Theobroma cacao* ] | 9.00E-123 |
| ppa000515m | gi|255552728| | NP_186786.2 | -1.91 | 35.2 | not assigned.unknown | ATCFM2 (crm family member 2); RNA binding [ *Arabidopsis thaliana* ] | 8E-64 |
| ppa011996m | gi|225462908| | NP_057154.2 | -1.91 | 35.2 | not assigned.unknown | 28S ribosomal protein S23, mitochondrial [ *Homo sapiens* ] | 0 |
| ppa008683m | gi|297745025| | XP_002511009.1 | -1.91 | 35.2 | not assigned.unknown | Catalytic, putative [ *Ricinus communis* ] | 1E-135 |
| ppa000025m | gi|225438692| | XP_002277575.1 | -1.91 | 35.2 | not assigned.unknown | Hypothetical protein [ *Vitis vinifera* ] | 7E-97 |
| ppa005291m | gi|255558113| | BAD96943.1 | -1.92 | 35.2 | not assigned.unknown | G protein-coupled receptor 89 variant [ *Homo sapiens* ] | 9E-128 |
| ppa005827m | gi|255641336| | XP_002530834.1 | -1.92 | 29.5.11 | protein.degradation.ubiquitin | 26S proteasome non-atpase regulatory subunit, putative [ *Ricinus communis* ] | 0 |
| ppa000673m | gi|224138752| | AAF81910.1 | -1.92 | 28.1 | DNA.synthesis/chromatin structure | DNA repair endonuclease UVH1-like [ *Glycine max* ] | 2E-102 |
| ppa000660m | gi|225447959| | NP_194494.2 | -1.92 | 29.3.1 | protein.targeting.nucleus | Importin beta-2 subunit family protein [ *Arabidopsis thaliana* ] | 6.2 |
| ppa004490m | gi|225438902| | XP_002524950.1 | -1.92 | 34.12 | transport.metal | Natural resistance-associated macrophage protein, putative [ *Ricinus communis* ] | 0 |
| ppa005718m | gi|209922600| | ACI96243.1 | -1.92 | 29.2.4 | protein.synthesis.elongation | Elongation factor 1-alpha [ *Prunus persica* ] | 3E-147 |
| ppa009184m | gi|225449539| | NP_001147221.1 | -1.93 | 35.2 | not assigned.unknown | Plant-specific domain TIGR01615 family protein [ *Zea mays* ] | 0 |
| ppa008498m | gi|217072084| | XP_002516715.1 | -1.93 | 1.1.5.4 | PS.lightreaction.other electron carrier (ox/red).ferredoxin oxireductase | Phytochromobilin:ferredoxin oxidoreductase, chloroplast precursor, putative [ *Ricinus communis* ] | 0 |
| ppa010961m | gi|296083047| | NP_179082.1 | -1.94 | 14.15 | S-assimilation.AKN | APK (aps kinase); ATP binding / adenylylsulfate kinase/ kinase/ transferase, transferring phosphorus-containing groups [ *Arabidopsis thaliana* ] | 2E-102 |
| ppa011210m | gi|255645833| | NP_001148441.1 | -1.94 | 35.2 | not assigned.unknown | Cyclase/dehydrase [ *Zea mays* ] | 2E-69 |
| ppa010937m | gi|225430384| | AAF14657.1 | -1.94 | 35.2 | not assigned.unknown | Contains similarity to gb|AF151904 CGI-146 protein from Homo sapiens. EST gb|T44446 comes from this gene [ *Arabidopsis thaliana* ] | 3E-97 |
| ppa005720m | gi|255574131| | NP_567873.1 | -1.94 | 31.1 | cell.organisation | Myosin heavy chain-related [ *Arabidopsis thaliana* ] | 2E-80 |
| ppa013054m | gi|255630845| | ADB94675.1 | -1.94 | 11.1.12 | lipid metabolism.FA synthesis and FA elongation.ACP protein | Acyl carrier protein 4 [ *Arachis hypogaea* ] | 5E-44 |
| ppa012289m | gi|224130146| | XP_002328665.1 | -1.95 | 35.2 | not assigned.unknown | Predicted protein [ *Populus trichocarpa* ] | 0 |
| ppa008525m | gi|255579712| | XP_002530695.1 | -1.95 | 34.9 | transport.metabolite transporters at the mitochondrial membrane | Mitochondrial dicarboxylate carrier protein, putative [ *Ricinus communis* ] | 0 |
| ppa001003m | gi|255561552| | XP_002521786.1 | -1.95 | 20.1 | stress.biotic | Disease resistance protein RPP13, putative [ *Ricinus communis* ] | 6E-63 |
| ppa000176m | gi|296087697| | XP_002517370.1 | -1.95 | 28.2 | DNA.repair | DNA-repair protein UVH3, putative [ *Ricinus communis* ] | 2E-93 |
| ppa006978m | gi|224135865| | XP_002511359.1 | -1.95 | 29.5 | protein.degradation | UBX domain-containing protein 8-B, putative [ *Ricinus communis* ] | 9E-131 |
| ppa000775m | gi|225459659| | XP_002520312.1 | -1.95 | 20.2.5 | stress.abiotic.light | Poly(p)/ATP NAD kinase, putative [ *Ricinus communis* ] | 0 |
| ppa018433m | gi|225456973| | XP_002511370.1 | -1.95 | 27.3.99 | RNA.regulation of transcription.unclassified | Zinc finger protein, putative [ *Ricinus communis* ] | 9E-95 |
| ppa007796m | gi|225444861| | XP_002330511.1 | -1.95 | 27.3.26 | RNA.regulation of transcription.MYB-related transcription factor family | Single myb histone [ *Populus trichocarpa* ] | 8E-164 |
| ppa013316m | gi|255637262| | AAD47346.1 | -1.95 | 29.2.1.2.1.26 | protein.synthesis.ribosomal protein.eukaryotic.40S subunit.S26 | Ribosomal protein S26 [ *Pisum sativum* ] | 0 |
| ppa009067m | gi|82568691| | BAE48659.1 | -1.95 | 17.5.1 | hormone metabolism.ethylene.synthesis-degradation | 2-oxoacid-dependent dioxygenase [ *Prunus mume* ] | 5E-51 |
| ppa000351m | gi|255555580| | NP_178053.3 | -1.96 | 33.99 | development.unspecified | EMB1135 (embryo defective 1135); DNA binding / protein binding / zinc ion binding [ *Arabidopsis thaliana* ] | 0 |
| ppa003676m | gi|255561295| | XP_002521658.1 | -1.96 | 29.4 | protein.postranslational modification | Calcium-dependent protein kinase, putative [ *Ricinus communis* ] | 0 |
| ppa012934m | gi|224056889| | XP_002532936.1 | -1.97 | 29.5.11.3 | protein.degradation.ubiquitin.E2 | Ubiquitin-conjugating enzyme E2, putative [ *Ricinus communis* ] | 9E-161 |
| ppa008948m | gi|225437203| | XP_002520975.1 | -1.97 | 13.1.6.5.5 | amino acid metabolism.synthesis.aromatic aa.tryptophan.tryptophan synthase | Trytophan synthase alpha subunit, putative [ *Ricinus communis* ] | 0 |
| ppa007998m | gi|225449493| | XP_002532295.1 | -1.97 | 16.1.1 | secondary metabolism.isoprenoids.non-mevalonate pathway | Geranylgeranyl transferase type I beta subunit, putative [ *Ricinus communis* ] | 2E-94 |
| ppa002259m | gi|224057952| | XP_002519390.1 | -1.97 | 15.1 | metal handling.acquisition | Ferric-chelate reductase, putative [ *Ricinus communis* ] | 0 |
| ppa007739m | gi|224053517| | XP_002297852.1 | -1.97 | 35.2 | not assigned.unknown | Predicted protein [ *Populus trichocarpa* ] | 0 |
| ppa010370m | gi|225450155| | XP_002526766.1 | -1.97 | 1.1.1.2 | PS.lightreaction.photosystem II.PSII polypeptide subunits | Thylakoid lumenal 19 kda protein, chloroplast precursor, putative [ *Ricinus communis* ] | 8E-80 |
| ppa025097m |  | XP_002529900.1 | -1.98 | 2.1.2.4 | major CHO metabolism.synthesis.starch.debranching | Isoamylase, putative [ *Ricinus communis* ] | 0 |
| ppa004457m | gi|225440692| | XP_002873012.1 | -1.98 | 35.1 | not assigned.no ontology | LMBR1 integral membrane family protein [ *Arabidopsis lyrata subsp. Lyrata* ] | 0 |
| ppa007202m | gi|225425368| | ACL51016.1 | -1.98 | 33.3 | development.squamosa promoter binding like (SPL) | Squamosa promoter-binding protein [ *Citrus trifoliata* ] | 2E-155 |
| ppb024455m | gi|224094995| | XP_002310320.1 | -1.98 | 35.2 | not assigned.unknown | Predicted protein [ *Populus trichocarpa* ] | 0 |
| ppa000805m | gi|224092282| | XP_002309542.1 | -1.98 | 35.2 | not assigned.unknown | Predicted protein [ *Populus trichocarpa* ] | 0 |
| ppa010097m | gi|225444061| | NP_567759.1 | -1.98 | 21.2.1.2 | redox.ascorbate and glutathione.ascorbate.GDP-L-galactose-hexose-1-phosphate guanyltransferase | VTC2 (vitamin c defective 2); GDP-D-glucose phosphorylase/ GDP-galactose:glucose-1-phosphate guanyltransferase/ GDP-galactose:mannose-1-phosphate guanyltransferase/ GDP-galactose:myoinositol-1-phosphate guanyltransferase/ galactose-1-phosphate guanylyltransferase (GDP)/ glucose-1-phosphate guanylyltransferase (GDP)/ mannose-1-phosphate guanylyltransferase (GDP)/ quercetin 4'-O-glucosyltransferase [ *Arabidopsis thaliana* ] | 0 |
| ppa003430m | gi|225459475| | AAK93632.1 | -1.98 | 17.2.3 | hormone metabolism.auxin.induced-regulated-responsive-activated | Putative growth regulator protein [ *Arabidopsis thaliana* ] | 0 |
| ppa019257m | gi|224055327| | XP_002298482.1 | -1.99 | 17.5.1 | hormone metabolism.ethylene.synthesis-degradation | 20G-Fe(II) oxidoreductase [ *Populus trichocarpa* ] | 0 |
| ppa012777m | gi|255561168| | XP_002521596.1 | -1.99 | 35.2 | not assigned.unknown | Protein FAM96B, putative [ *Ricinus communis* ] | 2E-73 |
| ppa010036m | gi|255642072| | ACU21302.1 | -1.99 | 35.2 | not assigned.unknown | Unknown [ *Glycine max* ] | 0 |
| ppa007419m | gi|255543122| | AAN63500.1 | -1.99 | 35.2 | not assigned.unknown | Lysine ketoglutarate reductase trans-splicing related 1 [ *Arabidopsis thaliana* ] | 1E-145 |
| ppa008142m | gi|6692816| | BAA89423.1 | -1.99 | 26.7 | misc.oxidases - copper, flavone etc | Allyl alcohol dehydrogenase [ *Nicotiana tabacum* ] | 0 |
| ppa007889m | gi|255576223| | XP_002529005.1 | -1.99 | 35.2 | not assigned.unknown | Non-imprinted in Prader-Willi/Angelman syndrome region protein, putative [ *Ricinus communis* ] | 1E-45 |
| ppa004562m | gi|224064854| | ADK92867.1 | -2.00 | 35.1 | not assigned.no ontology | DGCR-like protein [ *Hypericum perforatum* ] | 8E-48 |
| ppa010085m | gi|224060471| | XP_002300216.1 | -2.00 | 35.2 | not assigned.unknown | Predicted protein [ *Populus trichocarpa* ] | 1.00E-163 |
| ppa005655m | gi|225438253| | NP_566927.1 | -2.00 | 27.3.64 | RNA.regulation of transcription.PHOR1 | U-box domain-containing protein [ *Arabidopsis thaliana* ] | 3E-88 |
| ppa011096m | gi|255587476| | YP_002372015.1 | -2.00 | 35.2 | not assigned.unknown | Putative thiol-disulfide oxidoreductase DCC [ *Cyanothece sp. PCC 8801* ] | 4E-87 |
| ppa012845m | gi|90186542| | AAT66935.1 | -2.00 | 21.6 | redox.dismutases and catalases | Superoxide dismutase [ *Malus xiaojinensis* ] | 0 |
| ppa003418m | gi|224074599| | NP_567588.1 | -2.00 | 33.99 | development.unspecified | Nodulin-related [ *Arabidopsis thaliana* ] | 0.002 |
| ppa010430m | gi|225452924| | CBI22270.3 | -2.00 | 35.1 | not assigned.no ontology | Unnamed protein product [ *Vitis vinifera* ] | 0 |
| ppa018164m | gi|255547379| | XP_002875843.1 | -2.00 | 35.2 | not assigned.unknown | Hypothetical protein ARALYDRAFT_485116 [ *Arabidopsis lyrata subsp. Lyrata* ] | 8E-124 |
| ppa008981m | gi|225454961| | ABH07405.1 | -2.01 | 35.2 | not assigned.unknown | OR [ *Brassica oleracea var. Botrytis* ] | 0 |
| ppa007447m | gi|296085803| | XP_002519982.1 | -2.01 | 25.5 | C1-metabolism.Methylenetetrahydrofolate dehydrogenase & Methenyltetrahydrofolate cyclohydrolase | Methylenetetrahydrofolate dehydrogenase, putative [ *Ricinus communis* ] | 0 |
| ppa012919m | gi|225429714| | XP_002308515.1 | -2.01 | 34.12 | transport.metal | Copper transporter [ *Populus trichocarpa* ] | 2E-95 |
| ppa008830m | gi|225445248| | XP_002514309.1 | -2.01 | 27.3.23 | RNA.regulation of transcription.HSF,Heat-shock transcription factor family | DNA binding protein, putative [ *Ricinus communis* ] | 2E-147 |
| ppa001469m | gi|224087319| | ADW11232.1 | -2.01 | 29.5.1 | protein.degradation.subtilases | Subtilisin-like protease 1 [ *Phaseolus vulgaris* ] | 6E-22 |
| ppa009204m | gi|225423527| | XP_002892778.1 | -2.01 | 27.3 | RNA.regulation of transcription | DRL1 protein [ *Arabidopsis lyrata subsp. Lyrata* ] | 3.00E-162 |
| ppa008671m | gi|157365234| | ABV44811.1 | -2.01 | 16.8.3.1 | secondary metabolism.flavonoids.dihydroflavonols.dihydroflavonol 4-reductase | Cinnamyl alcohol dehydrogenase 2 [ *Eriobotrya japonica* ] | 1E-173 |
| ppa000845m | gi|296085525| | ADN33699.1 | -2.01 | 27.4 | RNA.RNA binding | RNA-binding protein [ *Cucumis melo subsp. Melo* ] | 0 |
| ppa002746m | gi|225451199| | BAB40709.1 | -2.02 | 31.1 | cell.organisation | BY-2 kinesin-like protein 5 [ *Nicotiana tabacum* ] | 7E-91 |
| ppa009597m | gi|217074094| | XP_002520001.1 | -2.02 | 29.8 | protein.assembly and cofactor ligation | Electron transporter, putative [ *Ricinus communis* ] | 0 |
| ppa010945m | gi|225425294| | NP_197083.1 | -2.03 | 27.3.99 | RNA.regulation of transcription.unclassified | Zinc finger (C3HC4-type RING finger) family protein [ *Arabidopsis thaliana* ] | 0 |
| ppa004573m | gi|297736512| | XP_002871149.1 | -2.03 | 35.2 | not assigned.unknown | Hypothetical protein ARALYDRAFT_487324 [ *Arabidopsis lyrata subsp. Lyrata* ] | 0 |
| ppa003560m | gi|225447818| | XP_002526061.1 | -2.03 | 35.2 | not assigned.unknown | Multidrug resistance pump, putative [ *Ricinus communis* ] | 1E-55 |
| ppa008491m | gi|118488767| | NP_193037.1 | -2.03 | 26.7 | misc.oxidases - copper, flavone etc | Oxidoreductase, zinc-binding dehydrogenase family protein [ *Arabidopsis thaliana* ] | 1E-68 |
| ppa000482m | gi|296086225| | NP_974443.4 | -2.03 | 31.4 | cell.vesicle transport | Adaptin family protein [ *Arabidopsis thaliana* ] | 6E-144 |
| ppa1027165m | gi|255567349| | XP_002524654.1 | -2.03 | 35.2 | not assigned.unknown | Conserved hypothetical protein [ *Ricinus communis* ] | 0 |
| ppa012595m | gi|255542502| | XP_002512314.1 | -2.04 | 30.1 | signalling.phosphorelay | Histidine-containing phosphotransfer protein, putative [ *Ricinus communis* ] | 4E-82 |
| ppa004926m | gi|255550307| | XP_002516204.1 | -2.04 | 27.3.99 | RNA.regulation of transcription.unclassified | Zinc ion binding protein, putative [ *Ricinus communis* ] | 0 |
| ppa003514m | gi|224072254| | XP_002518875.1 | -2.04 | 3.2.4 | minor CHO metabolism.trehalose.trehalase | Alpha,alpha-trehalase, putative [ *Ricinus communis* ] | 0 |
| ppa006647m | gi|225460879| | XP_002529466.1 | -2.04 | 23.1.1.2 | nucleotide metabolism.synthesis.pyrimidine.aspartate transcarbamoylase | Aspartate carbamoyltransferase 2, chloroplast precursor, putative [ *Ricinus communis* ] | 0 |
| ppa012409m | gi|225444335| | EEE68716.1 | -2.04 | 35.2 | not assigned.unknown | Hypothetical protein osj_27374 [ *Oryza sativa Japonica Group* ] | 9E-120 |
| ppa009971m | gi|225458910| | XP_002463943.1 | -2.04 | 35.2 | not assigned.unknown | Hypothetical protein SORBIDRAFT_01g009360 [ *Sorghum bicolor* ] | 2E-133 |
| ppa009994m | gi|225425061| | CAN71337.1 | -2.04 | 35.2 | not assigned.unknown | Hypothetical protein VITISV_035762 [ *Vitis vinifera* ] | 5E-76 |
| ppa023910m | gi|296082534| | XP_002532774.1 | -2.04 | 29.5.11.5 | protein.degradation.ubiquitin.ubiquitin protease | Ubiquitin-specific protease, putative [ *Ricinus communis* ] | 4E-109 |
| ppa007148m | gi|255584954| | XP_002533189.1 | -2.04 | 34.12 | transport.metal | Urease accessory protein ureh, putative [ *Ricinus communis* ] | 7E-168 |
| ppa002659m | gi|225429644| | XP_002873549.1 | -2.05 | 35.1 | not assigned.no ontology | Mechanosensitive ion channel domain-containing protein [ *Arabidopsis lyrata subsp. Lyrata* ] | 1E-46 |
| ppa012106m | gi|297745938| | XP_002525606.1 | -2.05 | 35.1 | not assigned.no ontology | Josephin-2, putative [ *Ricinus communis* ] | 3E-45 |
| ppa002100m | gi|225457015| | XP_002511389.1 | -2.05 | 34.18 | transport.unspecified anions | Boron transporter, putative [ *Ricinus communis* ] | 0 |
| ppa008437m | gi|255546185| | XP_002514152.1 | -2.05 | 34.9 | transport.metabolite transporters at the mitochondrial membrane | Grave disease carrier protein, putative [ *Ricinus communis* ] | 0 |
| ppa005024m | gi|225428857| | XP_002992800.1 | -2.06 | 35.2 | not assigned.unknown | Hypothetical protein SELMODRAFT_269987 [ *Selaginella moellendorffii* ] | 0 |
| ppa003922m | gi|225441228| | XP_002881760.1 | -2.06 | 34.3 | transport.amino acids | Amino acid transporter family protein [ *Arabidopsis lyrata subsp. Lyrata* ] | 0 |
| ppa012650m | gi|190613877| | ACE80942.1 | -2.06 | 27.1.19 | RNA.processing.ribonucleases | Putative allergen Pru p 1.02 [ *Prunus dulcis x Prunus persica* ] | 5E-21 |
| ppa024780m | gi|224061979| | CAN63050.1 | -2.07 | 35.2 | not assigned.unknown | Hypothetical protein VITISV_027808 [ *Vitis vinifera* ] | 0 |
| ppa005229m | gi|225436765| | XP_002520668.1 | -2.07 | 27.3.99 | RNA.regulation of transcription.unclassified | Nucleic acid binding protein, putative [ *Ricinus communis* ] | 0 |
| ppa006089m | gi|225423489| | XP_002512091.1 | -2.07 | 34.8 | transport.metabolite transporters at the envelope membrane | Triose phosphate/phosphate translocator, chloroplast precursor, putative [ *Ricinus communis* ] | 5E-131 |
| ppa000200m | gi|255562960| | XP_002522485.1 | -2.07 | 33.99 | development.unspecified | Pattern formation protein, putative [ *Ricinus communis* ] | 0 |
| ppa008787m | gi|255552788| | XP_002517437.1 | -2.08 | 26.7 | misc.oxidases - copper, flavone etc | Prolyl 4-hydroxylase alpha subunit, putative [ *Ricinus communis* ] | 7E-165 |
| ppa000110m | gi|255541336| | EOX96191.1 | -2.08 | 30.5 | signalling.G-proteins | SEC7-like guanine nucleotide exchange family protein [ *Theobroma cacao* ] | 0 |
| ppa006798m | gi|255583042| | XP_002884671.1 | -2.08 | 30.5 | signalling.G-proteins | Rabgap/TBC domain-containing protein [ *Arabidopsis lyrata subsp. Lyrata* ] | 2E-179 |
| ppa008470m | gi|255581935| | XP_002531766.1 | -2.08 | 27.3.62 | RNA.regulation of transcription.Nucleosome/chromatin assembly factor group | Transcription factor, putative [ *Ricinus communis* ] | 2E-84 |
| ppa008010m | gi|118483148| | XP_002869999.1 | -2.08 | 35.2 | not assigned.unknown | Hypothetical protein ARALYDRAFT_492939 [ *Arabidopsis lyrata subsp. Lyrata* ] | 0 |
| ppa011349m | gi|255641784| | XP_002513374.1 | -2.09 | 29.5.11.20 | protein.degradation.ubiquitin.proteasom | Proteasome subunit alpha type, putative [ *Ricinus communis* ] | 0 |
| ppa005497m | gi|225448723| | BAD95247.1 | -2.09 | 26.23 | misc.rhodanese | Rhodanese like protein [ *Arabidopsis thaliana* ] | 0 |
| ppa004409m | gi|225437158| | XP_002871909.1 | -2.09 | 27.3.57 | RNA.regulation of transcription.JUMONJI family | Transcription factor jumonji domain-containing protein [ *Arabidopsis lyrata subsp. Lyrata* ] | 4E-164 |
| ppa015604m | gi|147772239| | XP_002531149.1 | -2.09 | 35.1.5 | not assigned.no ontology.pentatricopeptide (PPR) repeat-containing protein | Pentatricopeptide repeat-containing protein, putative [ *Ricinus communis* ] | 2E-107 |
| ppa000437m | gi|297734185| | XP_002511967.1 | -2.10 | 35.1 | not assigned.no ontology | F-box and wd40 domain protein, putative [ *Ricinus communis* ] | 0 |
| ppa008734m | gi|224129560| | XP_002869476.1 | -2.10 | 35.2 | not assigned.unknown | Hypothetical protein ARALYDRAFT_913640 [ *Arabidopsis lyrata subsp. Lyrata* ] | 1E-135 |
| ppa009520m | gi|255573584| | XP_002864509.1 | -2.10 | 35.2 | not assigned.unknown | Hypothetical protein ARALYDRAFT_495827 [ *Arabidopsis lyrata subsp. Lyrata* ] | 0 |
| ppa004968m | gi|157734205| | ABV68925.1 | -2.11 | 26.2 | misc.UDP glucosyl and glucoronyl transferases | Mandelonitrile glucosyltransferase UGT85A19 [ *Prunus dulcis* ] | 0 |
| ppa004963m | gi|225458024| | AAM63309.1 | -2.11 | 14.1 | S-assimilation.ATPS | Sulfate adenylyltransferase [ *Arabidopsis thaliana* ] | 2E-98 |
| ppa016008m | gi|225444800| | XP_002514074.1 | -2.11 | 27.3.14 | RNA.regulation of transcription.CCAAT box binding factor family, HAP2 | Nuclear transcription factor Y subunit A-3, putative [ *Ricinus communis* ] | 1E-103 |
| ppa005570m | gi|225461677| | XP_002524520.1 | -2.11 | 35.2 | not assigned.unknown | Rab3 gtpase-activating protein non-catalytic subunit, putative [ *Ricinus communis* ] | 0 |
| ppa005515m | gi|225441890| | EOY09194.1 | -2.12 | 26.2 | misc.UDP glucosyl and glucoronyl transferases | UDP-glucosyltransferase, putative [ *Theobroma cacao* ] | 0 |
| ppa012891m | gi|224113229| | XP_002316429.1 | -2.13 | 35.2 | not assigned.unknown | Predicted protein [ *Populus trichocarpa* ] | 1.00E-99 |
| ppa022037m | gi|297742269| | XP_002509890.1 | -2.13 | 35.2 | not assigned.unknown | Transmembrane protein TPARL, putative [ *Ricinus communis* ] | 4E-45 |
| ppa005322m | gi|225426905| | XP_002509592.1 | -2.13 | 26.1 | misc.cytochrome P450 | Cytochrome P450, putative [ *Ricinus communis* ] | 0 |
| ppa008778m | gi|225446010| | XP_002512775.1 | -2.13 | 3.5 | minor CHO metabolism.others | Aldose 1-epimerase, putative [ *Ricinus communis* ] | 0 |
| ppa018924m | gi|147772498| | XP_002280197.1 | -2.13 | 31.1 | cell.organisation | Ankyrin repeat-containing protein At2g01680-like [ *Vitis vinifera* ] | 0 |
| ppa001291m | gi|224061527| | XP_002300524.1 | -2.14 | 33.99 | development.unspecified | NAC domain protein, IPR003441 [ *Populus trichocarpa* ] | 8E-168 |
| ppa004238m | gi|255586750| | XP_002533995.1 | -2.14 | 35.2 | not assigned.unknown | Conserved hypothetical protein [ *Ricinus communis* ] | 3E-08 |
| ppa007017m | gi|147806247| | CAN72196.1 | -2.14 | 35.2 | not assigned.unknown | Hypothetical protein VITISV_014980 [ *Vitis vinifera* ] | 3E-86 |
| ppa013172m | gi|224105759| | NP_196631.1 | -2.15 | 35.2 | not assigned.unknown | Aminoacyl-trna hydrolase/ protein tyrosine phosphatase [ *Arabidopsis thaliana* ] | 2E-54 |
| ppa003196m | gi|255568703| | XP_002525323.1 | -2.15 | 34.13 | transport.peptides and oligopeptides | Oligopeptide transporter, putative [ *Ricinus communis* ] | 9E-79 |
| ppa006708m | gi|225445464| | XP_002515534.1 | -2.15 | 34.9 | transport.metabolite transporters at the mitochondrial membrane | ADP,ATP carrier protein, putative [ *Ricinus communis* ] | 0 |
| ppa010433m | gi|255627565| | NP_001149877.1 | -2.16 | 35.1 | not assigned.no ontology | Transmembrane BAX inhibitor motif-containing protein 4 [ *Zea mays* ] | 5E-164 |
| ppa000916m | gi|224085908| | XP_002510557.1 | -2.16 | 30.2.11 | signalling.receptor kinases.leucine rich repeat XI | Receptor protein kinase CLAVATA1 precursor, putative [ *Ricinus communis* ] | 5E-137 |
| ppa006146m | gi|297745419| | NP_192013.1 | -2.16 | 35.1 | not assigned.no ontology | Glycosyl hydrolase family 18 protein [ *Arabidopsis thaliana* ] | 0 |
| ppa011948m | gi|225457381| | NP_173308.2 | -2.16 | 35.2 | not assigned.unknown | NDF6 (NDH DEPENDENT FLOW 6) [ *Arabidopsis thaliana* ] | 1E-109 |
| ppa010780m | gi|255583183| | XP_002532357.1 | -2.17 | 29.5 | protein.degradation | Ubiquitin carboxyl-terminal hydrolase isozyme L3, putative [ *Ricinus communis* ] | 0 |
| ppa015696m | gi|225459705| | XP_002284700.1 | -2.17 | 29.4.1.57 | protein.postranslational modification.kinase.receptor like cytoplasmatic kinase VII | Wall-associated receptor kinase 1-like [ *Vitis vinifera* ] | 0 |
| ppa019954m | gi|296084501| | CBI25060.3 | -2.17 | 35.2 | not assigned.unknown | Unnamed protein product [ *Vitis vinifera* ] | 2E-151 |
| ppa008806m | gi|225426649| | XP_002509630.1 | -2.18 | 27.3.67 | RNA.regulation of transcription.putative transcription regulator | DNA binding protein, putative [ *Ricinus communis* ] | 6E-31 |
| ppa014429m | gi|297735234| | XP_002533843.1 | -2.18 | 29.2.1.2.2.38 | protein.synthesis.ribosomal protein.eukaryotic.60S subunit.L38 | 60S ribosomal protein L38, putative [ *Ricinus communis* ] | 2E-53 |
| ppa007007m | gi|210063639| | ACJ06578.1 | -2.18 | 27.3.7 | RNA.regulation of transcription.C2C2(Zn) CO-like, Constans-like zinc finger family | CONSTANS [ *Fragaria x ananassa* ] | 0 |
| ppa005983m | gi|225442223| | XP_002514134.1 | -2.18 | 29.4 | protein.postranslational modification | N-myristoyl transferase, putative [ *Ricinus communis* ] | 0.0000001 |
| ppa010709m | gi|225454823| | ABC88653.1 | -2.19 | 35.2 | not assigned.unknown | Protein phosphatase 4 regulatory subunit 2 [ *Haemopis marmorata* ] | 0 |
| ppa000135m | gi|147802928| | XP_002892858.1 | -2.19 | 35.1 | not assigned.no ontology | Binding protein [ *Arabidopsis lyrata subsp. Lyrata* ] | 0 |
| ppa003315m | gi|14041827| | BAB55003.1 | -2.19 | 12.1.2 | N-metabolism.nitrate metabolism.nitrite reductase | Nitrite reductase [ *Prunus persica* ] | 1E-123 |
| ppa009408m | gi|225468850| | XP_002524571.1 | -2.19 | 7.1.3 | OPP.oxidative PP.6-phosphogluconate dehydrogenase | 3-hydroxyisobutyrate dehydrogenase, putative [ *Ricinus communis* ] | 4E-173 |
| ppa023662m | gi|255559925| | XP_002889432.1 | -2.19 | 23.3.2.2 | nucleotide metabolism.salvage.nucleoside kinases.uridine kinase | Phosphoribulokinase/uridine kinase [ *Arabidopsis lyrata subsp. Lyrata* ] | 0.00E+00 |
| ppa027017m | gi|297743970| | NP_567222.1 | -2.19 | 29.5.11.4.2 | protein.degradation.ubiquitin.E3.RING | PIT1 (pitchoun 1); protein binding / zinc ion binding [ *Arabidopsis thaliana* ] | 2E-48 |
| ppa002037m | gi|297738969| | CBI28214.3 | -2.20 | 35.2 | not assigned.unknown | Unnamed protein product [ *Vitis vinifera* ] | 0 |
| ppa015080m | gi|255540279| | XP_002511204.1 | -2.21 | 35.2 | not assigned.unknown | Conserved oligomeric Golgi complex component, putative [ *Ricinus communis* ] | 0 |
| ppa011034m | gi|297739290| | XP_002279657.1 | -2.21 | 29.2.1.1.1.2.6 | protein.synthesis.ribosomal protein.prokaryotic.chloroplast.50S subunit.L6 | Structural constituent of ribosome [ *Vitis vinifera* ] | 8E-138 |
| ppa005783m | gi|225007946| | EOX99434.1 | -2.21 | 10.6.2 | cell wall.degradation.mannan-xylose-arabinose-fucose | Glycosyl hydrolase superfamily protein [ *Theobroma cacao* ] | 0 |
| ppa008919m | gi|255572422| | XP_002527148.1 | -2.22 | 35.2 | not assigned.unknown | Conserved hypothetical protein [ *Ricinus communis* ] | 0 |
| ppa007026m | gi|255564816| | XP_002523402.1 | -2.22 | 29.4.1.57 | protein.postranslational modification.kinase.receptor like cytoplasmatic kinase VII | Protein kinase APK1B, chloroplast precursor, putative [ *Ricinus communis* ] | 1E-127 |
| ppa003810m | gi|255574863| | XP_002528338.1 | -2.22 | 19.1 | tetrapyrrole synthesis.glu-tRNA synthetase | Aspartyl/glutamyl-trna(Asn/Gln) amidotransferase subunit B, putative [ *Ricinus communis* ] | 9E-141 |
| ppa005946m | gi|255544624| | XP_002513373.1 | -2.22 | 27.1.2 | RNA.processing.RNA helicase | Dead box ATP-dependent RNA helicase, putative [ *Ricinus communis* ] | 0 |
| ppa008782m | gi|224114273| | XP_002519655.1 | -2.23 | 29.5.5 | protein.degradation.serine protease | Protease degs precursor, putative [ *Ricinus communis* ] | 0 |
| ppa014406m | - | unkown | -2.23 | 35.2 | not assigned.unknown | Unkown | 0 |
| ppa015834m | gi|225448552| | XP_002520784.1 | -2.23 | 26.1 | misc.misc2 | Endo beta n-acetylglucosaminidase, putative [ *Ricinus communis* ] | 5E-78 |
| ppa009696m | gi|225427732| | XP_002265886.1 | -2.23 | 35.2 | not assigned.unknown | Uncharacterized protein LOC100243481 [ *Vitis vinifera* ] | 0 |
| ppa011230m | gi|147795757| | ACG27544.1 | -2.24 | 29.3.5 | protein.targeting.peroxisomes | Peroxisomal membrane protein PMP22 [ *Zea mays* ] | 0 |
| ppa004462m | gi|225448900| | XP_002532624.1 | -2.24 | 11.1.13 | lipid metabolism.FA synthesis and FA elongation.acyl-CoA binding protein | Acyl-coa binding protein, putative [ *Ricinus communis* ] | 7E-133 |
| ppa004055m | gi|255552640| | NP_177185.3 | -2.24 | 35.1 | not assigned.no ontology | NHL repeat-containing protein [ *Arabidopsis thaliana* ] | 0 |
| ppa009556m | gi|225442665| | XP_002528196.1 | -2.24 | 27.3.30 | RNA.regulation of transcription.Trihelix, Triple-Helix transcription factor family | Transcription factor, putative [ *Ricinus communis* ] | 0 |
| ppa006654m | gi|225452638| | XP_002864826.1 | -2.25 | 29.5.11.4.2 | protein.degradation.ubiquitin.E3.RING | Protein binding protein [ *Arabidopsis lyrata subsp. Lyrata* ] | 2.00E-53 |
| ppa007907m | gi|225423935| | YP_001995215.1 | -2.25 | 35.2 | not assigned.unknown | Family 9 glycosyl transferase [ *Chloroherpeton thalassium ATCC 35110* ] | 0 |
| ppa004980m | gi|284519840| | ADB92670.1 | -2.25 | 34.3 | transport.amino acids | Amino acid permease 6 [ *Populus tremula x Populus alba* ] | 0 |
| ppa014138m | gi|224130486| | XP_002320849.1 | -2.25 | 35.2 | not assigned.unknown | Predicted protein [ *Populus trichocarpa* ] | 1.9 |
| ppa013538m | - | unkown | -2.26 | 35.2 | not assigned.unknown | Unkown | 0 |
| ppa003254m | gi|255579669| | XP_002530674.1 | -2.26 | 35.1 | not assigned.no ontology | Endomembrane protein emp70, putative [ *Ricinus communis* ] | 0 |
| ppa005525m | gi|255578386| | AAC69460.1 | -2.27 | 27.3.34 | RNA.regulation of transcription.Orphan family | ROOT HAIRLESS 1 [ *Arabidopsis thaliana* ] | 5E-79 |
| ppa010107m | gi|255585960| | XP_002533651.1 | -2.27 | 35.2 | not assigned.unknown | Nodulation receptor kinase precursor, putative [ *Ricinus communis* ] | 0 |
| ppa008602m | gi|255550858| | AAD40142.1 | -2.27 | 35.2 | not assigned.unknown | Contains similarity to several Arabidopsis thaliana hypothetical proteins including GB:U95973 and GB:AC002392 [ ] | 0 |
| ppa025823m | gi|224146120| | XP_002524008.1 | -2.27 | 11.1.8 | lipid metabolism.FA synthesis and FA elongation.acyl coa ligase | AMP dependent ligase, putative [ *Ricinus communis* ] | 0 |
| ppa026745m | gi|225439311| | XP_002267275.1 | -2.27 | 29.5 | protein.degradation | Probable glutamate carboxypeptidase 2-like [ *Vitis vinifera* ] | 0 |
| ppa026217m | gi|224063167| | XP_002513850.1 | -2.27 | 35.1 | not assigned.no ontology | Cornichon, putative [ *Ricinus communis* ] | 2E-142 |
| ppa000745m | gi|289540885| | ADD09562.1 | -2.27 | 30.3 | signalling.calcium | Calcium atpase [ *Trifolium repens* ] | 0 |
| ppa002725m | gi|224134691| | XP_002510861.1 | -2.28 | 35.2 | not assigned.unknown | Molybdopterin cofactor sulfurase, putative [ *Ricinus communis* ] | 3E-132 |
| ppa002466m | gi|225442081| | NP_566475.1 | -2.28 | 11.9.2.1 | lipid metabolism.lipid degradation.lipases.triacylglycerol lipase | Lipase class 3 family protein [ *Arabidopsis thaliana* ] | 0 |
| ppa001820m | gi|225434528| | AAF04293.2 | -2.28 | 28.1.1 | DNA.synthesis/chromatin structure.retrotransposon/transposase | Fructose-6-phosphate 2-kinase/fructose-2,6-bisphosphatase [ *Arabidopsis thaliana* ] | 3E-106 |
| ppa011187m | gi|255548872| | XP_002515492.1 | -2.29 | 31.4 | cell.vesicle transport | Vesicle-associated membrane protein, putative [ *Ricinus communis* ] | 0 |
| ppa023083m | gi|225449975| | NP_191650.2 | -2.29 | 13.1.3.4.12 | amino acid metabolism.synthesis.aspartate family.methionine.homocysteine S-methyltransferase | Catalytic/ methyltransferase [ *Arabidopsis thaliana* ] | 2E-12 |
| ppa010604m | gi|225444734| | ACG28615.1 | -2.29 | 35.2 | not assigned.unknown | MTD1 [ *Zea mays* ] | 2E-126 |
| ppa009536m | gi|297741277| | XP_002518765.1 | -2.31 | 11.8.1 | lipid metabolism.'exotics' (steroids, squalene etc).sphingolipids | Longevity assurance factor, putative [ *Ricinus communis* ] | 3E-104 |
| ppa025053m | gi|225424736| | XP_002531394.1 | -2.31 | 33.99 | development.unspecified | Auxin-induced protein 5NG4, putative [ *Ricinus communis* ] | 1E-82 |
| ppa001354m | gi|147794040| | XP_002520435.1 | -2.32 | 2.2.2.2 | major CHO metabolism.degradation.starch.starch phosphorylase | Glycogen phosphorylase, putative [ *Ricinus communis* ] | 0 |
| ppa005748m | gi|297743555| | EOY25933.1 | -2.32 | 31.1 | cell.organisation | Plastid transcriptionally active 17 isoform 1 [ *Theobroma cacao* ] | 0 |
| ppa019644m | gi|225454236| | XP_002302586.1 | -2.33 | 17.5.1 | hormone metabolism.ethylene.synthesis-degradation | 2-oxoglutarate-dependent dioxygenase [ *Populus trichocarpa* ] | 7E-143 |
| ppa007125m | gi|224095851| | ZP_08066706.1 | -2.33 | 35.2 | not assigned.unknown | Octaprenyl-diphosphate synthase [ *Actinobacillus ureae ATCC 25976* ] | 0.85 |
| ppa024800m | gi|255540503| | XP_002511316.1 | -2.33 | 35.2 | not assigned.unknown | Conserved hypothetical protein [ *Ricinus communis* ] | 2.00E-106 |
| ppa009306m | gi|225435760| | XP_002285722.1 | -2.34 | 34.9 | transport.metabolite transporters at the mitochondrial membrane | Similar to dicarboxylate/tricarboxylate carrier [ *Vitis vinifera* ] | 0 |
| ppa021513m | gi|225431096| | NP_201416.1 | -2.34 | 26.3 | misc.gluco-, galacto- and mannosidases | Glycosyl hydrolase family 38 protein [ *Arabidopsis thaliana* ] | 5E-143 |
| ppa009519m | gi|224142221| | XP_002527712.1 | -2.35 | 18.00 | Co-factor and vitamine metabolism | Ubiquinone/menaquinone biosynthesis methyltransferase, putative [ *Ricinus communis* ] | 6.4 |
| ppa015487m | gi|225448747| | NP_001102252.1 | -2.35 | 35.2 | not assigned.unknown | Transmembrane protein 103 [ *Rattus norvegicus* ] | 7E-163 |
| ppa010786m | gi|255550062| | XP_002516082.1 | -2.35 | 35.2 | not assigned.unknown | Conserved hypothetical protein [ *Ricinus communis* ] | 2E-20 |
| ppa005713m | gi|225423977| | XP_002533412.1 | -2.35 | 27.3.7 | RNA.regulation of transcription.C2C2(Zn) CO-like, Constans-like zinc finger family | Transcription factor, putative [ *Ricinus communis* ] | 0 |
| ppa007579m | gi|224089012| | ABI64127.1 | -2.35 | 29.5.11.4.3.2 | protein.degradation.ubiquitin.E3.SCF.FBOX | Putative F-box and leucine-rich repeat protein [ *Jatropha curcas* ] | 2E-141 |
| ppa007439m | gi|255543965| | XP_002513045.1 | -2.35 | 35.2 | not assigned.unknown | Conserved hypothetical protein [ *Ricinus communis* ] | 0 |
| ppa010657m | gi|296081822| | CBI20827.3 | -2.36 | 35.2 | not assigned.unknown | Unnamed protein product [ *Vitis vinifera* ] | 1E-155 |
| ppa001869m | gi|225459663| | EOY16441.1 | -2.36 | 20.2.4 | stress.abiotic.touch/wounding | Wound-responsive family protein, putative isoform 3 [ *Theobroma cacao* ] | 0 |
| ppa005756m | gi|224096536| | NP_197715.1 | -2.36 | 20.2.1 | stress.abiotic.heat | DNAJ heat shock N-terminal domain-containing protein [ *Arabidopsis thaliana* ] | 5E-13 |
| ppa002765m | gi|255560645| | ADL36631.1 | -2.36 | 27.2 | RNA.transcription | C2H2L domain class transcription factor [ *Malus x domestica* ] | 0 |
| ppa014715m | gi|224093874| | NP_565522.1 | -2.37 | 29.5.11.4.3.2 | protein.degradation.ubiquitin.E3.SCF.FBOX | SKIP6 (SKP1 interacting partner 6); ubiquitin-protein ligase [ *Arabidopsis thaliana* ] | 5E-115 |
| ppa001029m | gi|224100089| | NP_564904.1 | -2.37 | 30.2.99 | signalling.receptor kinases.misc | Leucine-rich repeat family protein / protein kinase family protein [ *Arabidopsis thaliana* ] | 0 |
| ppa005082m | gi|224105323| | XP_002512973.1 | -2.37 | 3.7 | minor CHO metabolism.sugar kinases | Xylulose kinase, putative [ *Ricinus communis* ] | 0 |
| ppa005645m | gi|45385321| | AAM77643.2 | -2.37 | 11.2.3 | lipid metabolism.FA desaturation.omega 3 desaturase | Chloroplast omega-3 desaturase [ *Prunus persica* ] | 0 |
| ppa019271m | gi|255571222| | XP_002526561.1 | -2.38 | 30.2.11 | signalling.receptor kinases.leucine rich repeat XI | Receptor protein kinase, putative [ *Ricinus communis* ] | 0 |
| ppa025449m | gi|225441941| | EOY26168.1 | -2.38 | 30.2.11 | signalling.receptor kinases.leucine rich repeat XI | Leucine-rich receptor-like protein kinase family protein, putative isoform 1 [ *Theobroma cacao* ] | 0 |
| ppa005583m | gi|225426008| | XP_002509935.1 | -2.38 | 34.3 | transport.amino acids | Amino acid transporter, putative [ *Ricinus communis* ] | 0 |
| ppa004140m | gi|224108087| | XP_002517205.1 | -2.38 | 30.5 | signalling.G-proteins | Leucine rich repeat-containing protein, putative [ *Ricinus communis* ] | 0 |
| ppa001807m | gi|224061521| | XP_002300521.1 | -2.38 | 35.2 | not assigned.unknown | Predicted protein [ *Populus trichocarpa* ] | 4E-149 |
| ppa020984m | gi|297746180| | NP_190160.1 | -2.39 | 35.1 | not assigned.no ontology | Hydrolase family protein / HAD-superfamily protein [ *Arabidopsis thaliana* ] | 1E-144 |
| ppa004937m | gi|255571835| | XP_002526860.1 | -2.39 | 16.2 | secondary metabolism.phenylpropanoids | Anthocyanin 5-aromatic acyltransferase, putative [ *Ricinus communis* ] | 0 |
| ppa016549m | gi|255647249| | ABN08658.1 | -2.39 | 35.2 | not assigned.unknown | Hypothetical protein mtrdraft_AC157891g31v2 [ *Medicago truncatula* ] | 4E-180 |
| ppa000080m | gi|255579971| | XP_002530820.1 | -2.39 | 29.5.11.4.1 | protein.degradation.ubiquitin.E3.HECT | Hect ubiquitin-protein ligase, putative [ *Ricinus communis* ] | 0 |
| ppa013921m | gi|118483487| | ABK93642.1 | -2.40 | 35.2 | not assigned.unknown | Unknown [ *Populus trichocarpa* ] | 3E-71 |
| ppa011598m | gi|225456868| | ACV33078.2 | -2.40 | 34.4 | transport.nitrate | High-affinity nitrate transport system component [ *Cucumis sativus* ] | 2E-68 |
| ppa007825m | gi|255553843| | XP_002517962.1 | -2.40 | 35.1 | not assigned.no ontology | Peroxisomal membrane protein, putative [ *Ricinus communis* ] | 2E-45 |
| ppa008113m | gi|224132924| | XP_002517891.1 | -2.40 | 26.19 | misc.plastocyanin-like | Early nodulin 55-2 precursor, putative [ *Ricinus communis* ] | 5E-31 |
| ppb017681m | gi|224056272| | XP_002522135.1 | -2.40 | 29.5.5 | protein.degradation.serine protease | Serine carboxypeptidase, putative [ *Ricinus communis* ] | 0 |
| ppa005547m | gi|224071449| | XP_002518706.1 | -2.40 | 11.9.2.1 | lipid metabolism.lipid degradation.lipases.triacylglycerol lipase | Triacylglycerol lipase, putative [ *Ricinus communis* ] | 0 |
| ppa014415m | gi|167599639| | ABZ88805.1 | -2.41 | 34.1 | transport.p- and v-ATPases | ATP synthase subunit H protein [ *Hevea brasiliensis* ] | 7E-137 |
| ppa017023m | gi|224111566| | XP_002532228.1 | -2.41 | 34.99 | transport.misc | TRANSPARENT TESTA 12 protein, putative [ *Ricinus communis* ] | 5E-54 |
| ppa011862m | gi|255542110| | XP_002512119.1 | -2.41 | 35.2 | not assigned.unknown | Conserved hypothetical protein [ *Ricinus communis* ] | 2E-96 |
| ppa010364m | gi|255582378| | XP_002531978.1 | -2.42 | 34.19.2 | transport.Major Intrinsic Proteins.TIP | Tonoplast intrinsic protein, putative [ *Ricinus communis* ] | 8E-179 |
| ppa003748m | gi|144225745| | XP_003544590.1 | -2.43 | 29.4 | protein.postranslational modification | Protein phosphatase 2C 16-like [ *Glycine max* ] | 0 |
| ppa009587m | gi|296085337| | EOY06547.1 | -2.43 | 29.2.3 | protein.synthesis.initiation | Eukaryotic translation initiation factor 2 gamma subunit, GAMMA isoform 1 [ *Theobroma cacao* ] | 0 |
| ppa009630m | gi|260408312| | ACX37450.1 | -2.44 | 34.19.1 | transport.Major Intrinsic Proteins.PIP | Plasma membrane intrinsic protein [ *Hevea brasiliensis* ] | 0 |
| ppa012218m | gi|223029867| | ACM78615.1 | -2.44 | 26.21 | misc.protease inhibitor/seed storage/lipid transfer protein (LTP) family protein | Non-specific lipid-transfer protein-like protein [ *Tamarix hispida* ] | 0 |
| ppa019595m | gi|297830352| | XP_002883058.1 | -2.44 | 35.1 | not assigned.no ontology | Hypothetical protein ARALYDRAFT_479214 [ *Arabidopsis lyrata subsp. Lyrata* ] | 1E-106 |
| ppa025098m | gi|51507375| | CAH18935.1 | -2.45 | 10.6.3 | cell wall.degradation.pectate lyases and polygalacturonases | Polygalacturonase [ *Pyrus communis* ] | 0.00006 |
| ppa005148m | gi|224068939| | ABE80120.2 | -2.45 | 27.3.30 | RNA.regulation of transcription.Trihelix, Triple-Helix transcription factor family | Homeodomain-like [ *Medicago truncatula* ] | 7E-129 |
| ppa011130m | gi|225436243| | XP_002523967.1 | -2.45 | 29.2.1.1.1.2.3 | protein.synthesis.ribosomal protein.prokaryotic.chloroplast.50S subunit.L3 | 50S ribosomal protein L3, putative [ *Ricinus communis* ] | 0 |
| ppa011816m | gi|255557991| | XP_002520024.1 | -2.46 | 35.2 | not assigned.unknown | Conserved hypothetical protein [ *Ricinus communis* ] | 2E-63 |
| ppa015738m | gi|225433722| | XP_002529693.1 | -2.46 | 26.2 | misc.UDP glucosyl and glucoronyl transferases | UDP-glucosyltransferase, putative [ *Ricinus communis* ] | 0 |
| ppa002246m | gi|224133338| | XP_002517448.1 | -2.46 | 31.1 | cell.organisation | HIPL1 protein precursor, putative [ *Ricinus communis* ] | 3E-109 |
| ppa017449m | gi|224114347| | AAG43556.1 | -2.46 | 35.2 | not assigned.unknown | Avr9/Cf-9 rapidly elicited protein 180 [ *Nicotiana tabacum* ] | 0 |
| ppa011116m | gi|255542134| | AAZ23775.1 | -2.47 | 31.2.5 | cell.division.plastid | Plastid division regulator mine [ *Glycine max* ] | 0 |
| ppa014565m | gi|297737969| | XP_002524124.1 | -2.48 | 26.16 | misc.myrosinases-lectin-jacalin | ATPP2-A2, putative [ *Ricinus communis* ] | 0 |
| ppa016720m | gi|255544856| | XP_002513489.1 | -2.48 | 28.1 | DNA.synthesis/chromatin structure | Origin recognition complex subunit, putative [ *Ricinus communis* ] | 5E-85 |
| ppa012772m | gi|255561439| | XP_002521730.1 | -2.48 | 29.2.1.1.1.2.11 | protein.synthesis.ribosomal protein.prokaryotic.chloroplast.50S subunit.L11 | 50S robosomal protein L11, putative [ *Ricinus communis* ] | 7E-79 |
| ppa009525m | gi|224062595| | NP_196706.2 | -2.49 | 1.1.1.2 | PS.lightreaction.photosystem II.PSII polypeptide subunits | PsbP domain-containing protein 5 [ *Arabidopsis thaliana* ] | 3E-101 |
| ppa018449m | gi|125531904| | EAY78469.1 | -2.49 | 35.2 | not assigned.unknown | Hypothetical protein osi_33557 [ *Oryza sativa Indica Group* ] | 0 |
| ppa002233m | gi|225465206| | AAC33218.1 | -2.50 | 29.4 | protein.postranslational modification | Similar to cdc2 protein kinases [ *Arabidopsis thaliana* ] | 1E-52 |
| ppa002863m | gi|87138101| | ABD28287.1 | -2.50 | 29.5.11.4.3.2 | protein.degradation.ubiquitin.E3.SCF.FBOX | Circadian clock-associated FKF1 [ *Glycine max* ] | 0 |
| ppa000380m | gi|255560731| | XP_002521379.1 | -2.50 | 34.1 | transport.p- and v-ATPases | Unkown | 0 |
| ppa005467m | gi|224122284| | XP_002330585.1 | -2.50 | 34.3 | transport.amino acids | Amino acid permease [ *Populus trichocarpa* ] | 0 |
| ppa001319m | gi|255557168| | XP_002330585.1 | -2.50 | 35.2 | not assigned.unknown | Amino acid permease [ *Populus trichocarpa* ] | 0 |
| ppa008454m | gi|224088200| | XP_002519615.1 | -2.51 | 35.2 | not assigned.unknown | Lupus la ribonucleoprotein, putative [ *Ricinus communis* ] | 0 |
| ppa001296m | gi|225452990| | XP_002878181.1 | -2.51 | 35.2 | not assigned.unknown | Hypothetical protein ARALYDRAFT_486249 [ *Arabidopsis lyrata subsp. Lyrata* ] | 0 |
| ppa001733m | gi|255579183| | ABM68547.1 | -2.52 | 35.2 | not assigned.unknown | IFA binding protein [ *Lilium longiflorum* ] | 1E-103 |
| ppa004513m | gi|255567648| | ABN06034.1 | -2.52 | 35.1.8 | not assigned.no ontology.BSD domain-containing protein | Hypothetical protein mtrdraft_AC149576g13v2 [ *Medicago truncatula* ] | 8E-141 |
| ppa005991m | gi|225439856| | NP_190925.1 | -2.52 | 29.4 | protein.postranslational modification | AFC1 (ARABIDOPSIS FUS3-COMPLEMENTING GENE 1); ATP binding / kinase/ protein kinase/ protein serine/threonine kinase/ protein tyrosine kinase [ *Arabidopsis thaliana* ] | 0 |
| ppa003929m | gi|255575021| | XP_002528416.1 | -2.52 | 34.3 | transport.amino acids | Amino acid transporter, putative [ *Ricinus communis* ] | 2E-22 |
| ppa013788m | gi|255628261| | ABR09296.1 | -2.53 | 33.99 | development.unspecified | Ethphon-induced protein [ *Hevea brasiliensis* ] | 3E-44 |
| ppa004556m | gi|224142461| | XP_002532502.1 | -2.53 | 34.98 | transport.membrane system unknown | Regulatory protein uhpc, putative [ *Ricinus communis* ] | 0 |
| ppa023594m | gi|225469343| | XP_002270086.1 | -2.54 | 35.2 | not assigned.unknown | Hypothetical protein [ *Vitis vinifera* ] | 0 |
| ppb005202m | gi|225449018| | XP_002531678.1 | -2.54 | 23.3.2.1 | nucleotide metabolism.salvage.nucleoside kinases.adenosine kinase | Adenosine kinase, putative [ *Ricinus communis* ] | 0 |
| ppa004247m | gi|225458281| | XP_002510387.1 | -2.55 | 35.1 | not assigned.no ontology | Cytosolic purine 5-nucleotidase, putative [ *Ricinus communis* ] | 0 |
| ppa000447m | gi|225435213| | XP_002282042.1 | -2.56 | 23.1.1.1 | nucleotide metabolism.synthesis.pyrimidine.carbamoyl phosphate synthetase | Carbamoyl-phosphate synthase large chain-like [ *Vitis vinifera* ] | 0 |
| ppa007636m | gi|62632853| | AAX89399.1 | -2.56 | 17.5.1 | hormone metabolism.ethylene.synthesis-degradation | Flavanon 3 beta-hydroxylase [ *Pyrus communis* ] | 0.009 |
| ppa013855m | gi|224125608| | XP_001751913.1 | -2.56 | 35.2 | not assigned.unknown | Qc-SNARE, SYP5 family [ *Physcomitrella patens subsp. Patens* ] | 0 |
| ppa004971m | gi|297735060| | NP_195348.2 | -2.58 | 5.10 | fermentation.aldehyde dehydrogenase | ALDH3F1 (Aldehyde Dehydrogenase 3F1); 3-chloroallyl aldehyde dehydrogenase/ aldehyde dehydrogenase (NAD) [ *Arabidopsis thaliana* ] | 5E-135 |
| ppa010006m | gi|302142238| | ABD33279.1 | -2.58 | 29.5.11.4.2 | protein.degradation.ubiquitin.E3.RING | Zinc finger, RING-type [ *Medicago truncatula* ] | 2E-103 |
| ppa005138m | gi|224056719| | NP_973956.1 | -2.58 | 29.4.1.57 | protein.postranslational modification.kinase.receptor like cytoplasmatic kinase VII | Protein kinase family protein [ *Arabidopsis thaliana* ] | 2E-88 |
| ppa005298m | gi|225435925| | XP_002523229.1 | -2.58 | 30.1 | signalling.in sugar and nutrient physiology | Cysteine desulfurylase, putative [ *Ricinus communis* ] | 1E-129 |
| ppa008857m | gi|297742832| | XP_002320573.1 | -2.58 | 34.14 | transport.unspecified cations | Bile acid:Na+ symporter family protein [ *Populus trichocarpa* ] | 0 |
| ppa009476m | gi|297734310| | NP_974140.1 | -2.59 | 35.2 | not assigned.unknown | Oxidoreductase, acting on the CH-CH group of donors [ *Arabidopsis thaliana* ] | 4E-167 |
| ppa008008m | gi|302141995| | NP_192640.1 | -2.59 | 21.2.1 | redox.ascorbate and glutathione.ascorbate | APX4 (ascorbate peroxidase 4); heme binding / peroxidase [ *Arabidopsis thaliana* ] | 0 |
| ppa008093m | gi|225426407| | XP_002509794.1 | -2.60 | 27.3.67 | RNA.regulation of transcription.putative transcription regulator | DNA binding protein, putative [ *Ricinus communis* ] | 7E-127 |
| ppa018561m | gi|224133650| | XP_002511336.1 | -2.60 | 27.3.25 | RNA.regulation of transcription.MYB domain transcription factor family | R2r3-myb transcription factor, putative [ *Ricinus communis* ] | 2E-115 |
| ppa008477m | gi|255562130| | XP_002522073.1 | -2.60 | 35.2 | not assigned.unknown | Hypothetical protein RCOM_1382010 [ *Ricinus communis* ] | 1E-141 |
| ppa006699m | gi|224131208| | NP_568185.1 | -2.61 | 27.3.99 | RNA.regulation of transcription.unclassified | Mitochondrial transcription termination factor family protein / mterf family protein [ *Arabidopsis thaliana* ] | 0 |
| ppa010803m | gi|255541462| | XP_002273596.1 | -2.62 | 29.3.5 | protein.targeting.peroxisomes | Peroxisomal membrane protein 11C isoform 2 [ *Vitis vinifera* ] | 6.00E-151 |
| ppa012418m | gi|224134078| | XP_002516617.1 | -2.63 | 1.1.5.2 | PS.lightreaction.other electron carrier (ox/red).ferredoxin | Electron carrier, putative [ *Ricinus communis* ] | 1E-56 |
| ppa023588m | gi|225423925| | XP_002529140.1 | -2.66 | 35.1 | not assigned.no ontology | Catalytic, putative [ *Ricinus communis* ] | 0 |
| ppa007517m | gi|255540019| | XP_002284739.1 | -2.66 | 35.2 | not assigned.unknown | Similar to mtn19-like protein [ *Vitis vinifera* ] | 2E-138 |
| ppa001874m | gi|224122208| | XP_002266713.1 | -2.66 | 29.3.5 | protein.targeting.peroxisomes | Similar to peroxisomal targeting signal 1 receptor; PTS1 receptor; Pex5p [ *Vitis vinifera* ] | 3E-163 |
| ppa003401m | gi|296086100| | XP_002530414.1 | -2.67 | 23.1.2.8 | nucleotide metabolism.synthesis.purine.SAICAR lyase | Adenylosuccinate lyase, putative [ *Ricinus communis* ] | 0 |
| ppa001453m | gi|255567899| | XP_002524927.1 | -2.67 | 34.12 | transport.metal | Heavy metal cation transport atpase, putative [ *Ricinus communis* ] | 0 |
| ppa011580m | gi|255553189| | XP_002517637.1 | -2.69 | 35.2 | not assigned.unknown | TP binding protein, putative [ *Ricinus communis* ] | 0 |
| ppb025386m | gi|255561496| | XP_002521758.1 | -2.70 | 20.1.5 | stress.biotic.regulation of transcription | TMV resistance protein N, putative [ *Ricinus communis* ] | 0 |
| ppa001911m | gi|224133902| | XP_002511211.1 | -2.71 | 29.4.1.57 | signalling.receptor kinases.leucine rich repeat XI | Leucine-rich repeat protein, putative [ *Ricinus communis* ] | 0 |
| ppa020110m | gi|225455537| | XP_002513567.1 | -2.71 | 30.2.17 | signalling.receptor kinases.DUF 26 | Kinase, putative [ *Ricinus communis* ] | 2E-117 |
| ppa003959m | gi|227121304| | ACP19342.1 | -2.72 | 34.7 | transport.phosphate | Phosphate transporter [ *Glycine max* ] | 0 |
| ppb019489m | gi|6686397| | AAF23831.1 | -2.73 | 35.2 | not assigned.unknown | F1E22.12 [ *Arabidopsis thaliana* ] | 0 |
| ppa002325m | gi|225444007| | XP_002274799.1 | -2.73 | 34.14 | transport.unspecified cations | Similar to cation chloride cotransporter [ *Vitis vinifera* ] | 0 |
| ppa010373m | gi|217075290| | CBX43985.1 | -2.74 | 27.3.5 | RNA.regulation of transcription.ARR | Putative A-type response regulator 3 [ *Populus x canadensis* ] | 2E-64 |
| ppa009646m | gi|297738012| | XP_002533420.1 | -2.74 | 11.3.6 | lipid metabolism.Phospholipid synthesis.choline-phosphate cytidylyltransferase | Cholinephosphate cytidylyltransferase, putative [ *Ricinus communis* ] | 7E-169 |
| ppa002804m | gi|224121552| | XP_002529528.1 | -2.74 | 17.1.1.1.1 | hormone metabolism.abscisic acid.synthesis-degradation.synthesis.9-cis-epoxycarotenoid dioxygenase | 9-cis-epoxycarotenoid dioxygenase, putative [ *Ricinus communis* ] | 2E-65 |
| ppa013561m | gi|83701143| | ABC41134.1 | -2.75 | 29.5.11.4.3.4 | protein.degradation.ubiquitin.E3.SCF.RBX | RING-box protein [ *Arachis hypogaea* ] | 3E-131 |
| ppa008106m | gi|255641823| | XP_002515530.1 | -2.75 | 34.8 | transport.metabolite transporters at the envelope membrane | Triose phosphate/phosphate translocator, non-green plastid, chloroplast precursor, putative [ *Ricinus communis* ] | 5E-63 |
| ppa011331m | gi|255561933| | BAI52974.1 | -2.75 | 35.2 | not assigned.unknown | G1-like protein [ *Selaginella moellendorffii* ] | 0 |
| ppa004667m | gi|224129218| | XP_002532210.1 | -2.76 | 27.3.69 | RNA.regulation of transcription.SET-domain transcriptional regulator family | Ribulose-1,5 bisphosphate carboxylase/oxygenase large subunit N-methyltransferase, chloroplast precursor, putative [ *Ricinus communis* ] | 1E-62 |
| ppa012835m | gi|255562763| | XP_002522387.1 | -2.76 | 35.1 | not assigned.no ontology | Conserved hypothetical protein [ *Ricinus communis* ] | 4E-165 |
| ppa012348m | gi|225457329| | NP_001148446.1 | -2.76 | 35.2 | not assigned.unknown | Cp protein [ *Zea mays* ] | 1E-174 |
| ppa002913m | gi|225428969| | XP_002527501.1 | -2.76 | 30.11 | signalling.light | Signal transducer, putative [ *Ricinus communis* ] | 1E-80 |
| ppa006108m | gi|225427681| | XP_002517501.1 | -2.77 | 29.4 | protein.postranslational modification | Serine/threonine-protein kinase SAPK3, putative [ *Ricinus communis* ] | 0 |
| ppa009320m | gi|255570037| | EOY20373.1 | -2.77 | 30.2.99 | signalling.receptor kinases.misc | Plasmodesmata-located protein 6 [ *Theobroma cacao* ] | 0 |
| ppa010443m | gi|13898653| | AAK48847.1 | -2.77 | 10.7 | cell wall.modification | Expansin [ *Prunus cerasus* ] | 1E-141 |
| ppa010017m | gi|255580851| | XP_002531245.1 | -2.78 | 34.14 | transport.unspecified cations | Sodium-bile acid cotransporter, putative [ *Ricinus communis* ] | 0 |
| ppa025502m | gi|225441343| | CAN74767.1 | -2.80 | 35.2 | not assigned.unknown | Hypothetical protein VITISV_041860 [ *Vitis vinifera* ] | 7.00E-133 |
| ppa001936m | gi|224061234| | XP_002522779.1 | -2.81 | 10.2.1 | cell wall.cellulose synthesis.cellulose synthase | Cellulose synthase, putative [ *Ricinus communis* ] | 0 |
| ppa003360m | gi|255540909| | XP_002511519.1 | -2.81 | 34.13 | transport.peptides and oligopeptides | Nitrate transporter, putative [ *Ricinus communis* ] | 0 |
| ppa004350m | gi|225435987| | XP_002272429.1 | -2.82 | 29.4 | protein.postranslational modification | Similar to cdc2msc [ *Vitis vinifera* ] | 0 |
| ppa004877m | gi|255557022| | XP_002519544.1 | -2.82 | 35.2 | not assigned.unknown | Conserved hypothetical protein [ *Ricinus communis* ] | 0 |
| ppa000185m | gi|225449690| | _002534113.1 | -2.82 | 34.16 | transport.ABC transporters and multidrug resistance systems | ATP-binding cassette transporter, putative [ *Ricinus communis* ] | 2E-69 |
| ppa010650m | gi|147833421| | CAN68521.1 | -2.82 | 35.2 | not assigned.unknown | Hypothetical protein VITISV_025154 [ *Vitis vinifera* ] | 3E-81 |
| ppa012729m | gi|225431527| | XP_002873421.1 | -2.83 | 29.2.1.1.3.2.17 | protein.synthesis.ribosomal protein.prokaryotic.unknown organellar.50S subunit.L17 | Ribosomal protein L17 family protein [ *Arabidopsis lyrata subsp. Lyrata* ] | 4E-112 |
| ppa023516m | gi|224126577| | XP_002526502.1 | -2.83 | 26.18 | misc.invertase/pectin methylesterase inhibitor family protein | Pectinesterase inhibitor, putative [ *Ricinus communis* ] | 1E-27 |
| ppa009154m | gi|224130796| | NP_001042772.1 | -2.86 | 35.2 | not assigned.unknown | Os01g0284900 [ *Oryza sativa Japonica Group* ] | 0 |
| ppa022067m | gi|225470155| | XP_002528808.1 | -2.87 | 33.99 | protein.degradation | Ubiquitin ligase protein cop1, putative [ *Ricinus communis* ] | 5E-21 |
| ppa008304m | gi|297834322| | NP_566487.1 | -2.87 | 34.8 | transport.metabolite transporters at the envelope membrane | Nucleotide/sugar transporter family protein [ *Arabidopsis thaliana* ] | 0 |
| ppa020598m | gi|225442918| | XP_002526525.1 | -2.88 | 13.2.6.3 | amino acid metabolism.degradation.aromatic aa.tryptophan | 3-hydroxybutyryl-coa dehydratase, putative [ *Ricinus communis* ] | 0 |
| ppa008266m | gi|224091544| | XP_002309282.1 | -2.88 | 29.5.11.4.3.2 | protein.degradation.ubiquitin.E3.SCF.FBOX | F-box family protein [ *Populus trichocarpa* ] | 0 |
| ppa000089m | gi|255574960| | XP_002892858.1 | -2.89 | 35.2 | not assigned.unknown | Binding protein   [ *Arabidopsis lyrata subsp. Lyrata* ] | 0 |
| ppa002463m | gi|225451381| | XP_002531932.1 | -2.90 | 34.99 | transport.misc | Nicastrin precursor, putative [ *Ricinus communis* ] | 0 |
| ppa000530m | gi|225437320| | NP_567238.2 | -2.91 | 29.5.9 | protein.degradation.AAA type | AAA-type atpase family protein [ *Arabidopsis thaliana* ] | 0 |
| ppa010994m | gi|224108484| | XP_002892744.1 | -2.93 | 35.2 | not assigned.unknown | Hypothetical protein ARALYDRAFT_888702 [ *Arabidopsis lyrata subsp. Lyrata* ] | 2E-28 |
| ppa024708m | gi|147853140| | XP_002879100.1 | -2.94 | 27.4 | RNA.RNA binding | Dsrna-binding protein 2 [ *Arabidopsis lyrata subsp. Lyrata* ] | 1E-108 |
| ppa017260m | gi|255541844| | CAN78725.1 | -2.94 | 35.1.41 | not assigned.no ontology.hydroxyproline rich proteins | Hypothetical protein VITISV_020008 [ *Vitis vinifera* ] | 7.00E-69 |
| ppa010197m | gi|302142150| | XP_002866690.1 | -2.94 | 35.2 | not assigned.unknown | Hypothetical protein ARALYDRAFT_332797 [ *Arabidopsis lyrata subsp. Lyrata* ] | 0 |
| ppa004282m | gi|224105977| | XP_002880842.1 | -2.95 | 35.1 | not assigned.no ontology | Phosphoesterase family protein [ *Arabidopsis lyrata subsp. Lyrata* ] | 6E-129 |
| ppa011240m | gi|255562369| | XP_002522191.1 | -2.97 | 29.5.11.4.2 | protein.degradation.ubiquitin.E3.RING | RING-H2 finger protein ATL1L, putative [ *Ricinus communis* ] | 2E-126 |
| ppa012221m | gi|225440862| | XP_002870036.1 | -2.98 | 35.2 | not assigned.unknown | Hypothetical protein ARALYDRAFT_329677 [ *Arabidopsis lyrata subsp. Lyrata* ] | 1E-36 |
| ppa001401m | gi|225426136| | CAA66482.1 | -3.02 | 27.3.50 | RNA.regulation of transcription.General Transcription | Transcription factor [ *Vicia faba var. Minor* ] | 0 |
| ppa007122m | gi|296083914| | ABD28673.1 | -3.06 | 35.1.5 | not assigned.no ontology.pentatricopeptide (PPR) repeat-containing protein | Tetratricopeptide-like helical [ *Medicago truncatula* ] | 0 |
| ppa014122m | - | unkown | -3.06 | 35.2 | not assigned.unknown | Unkown | 0 |
| ppa002621m | gi|225424956| | XP_002514371.1 | -3.06 | 23.1.1.10 | nucleotide metabolism.synthesis.pyrimidine.CTP synthetase | Ctp synthase, putative [ *Ricinus communis* ] | 0 |
| ppa009692m | gi|296088907| | P82413.2 | -3.07 | 29.2.1.1.1.2.19 | protein.synthesis.ribosomal protein.prokaryotic.chloroplast.50S subunit.L19 | Recname: Full=50S ribosomal protein L19, chloroplastic; altname: Full=CL19; Flags: Precursor [ ] | 5E-67 |
| ppa011498m | gi|225441611| | XP_002525424.1 | -3.09 | 14.15 | S-assimilation.AKN | Adenyl sulfate kinase, putative [ *Ricinus communis* ] | 7E-70 |
| ppa008393m | gi|255552057| | NP_193208.2 | -3.09 | 33.99 | development.unspecified | PPD2; dioxygenase/ metal ion binding [ *Arabidopsis thaliana* ] | 1E-104 |
| ppa010539m | gi|225446168| | NP_850057.1 | -3.11 | 29.3.4.99 | protein.targeting.secretory pathway.unspecified | ARF3 (ADP-RIBOSYLATION FACTOR 3); protein binding [ *Arabidopsis thaliana* ] | 1E-96 |
| ppa007109m | gi|225425597| | ACW82829.1 | -3.12 | 29.5.11.4.3.2 | protein.degradation.ubiquitin.E3.SCF.FBOX | F-box/kelch protein [ *Vitis vinifera* ] | 0 |
| ppa005226m | gi|225428814| | XP_002330327.1 | -3.16 | 26.1 | misc.cytochrome P450 | Cytochrome P450 [ *Populus trichocarpa* ] | 0 |
| ppa004037m | gi|225441006| | ABG34547.1 | -3.20 | 10.2 | cell wall.cellulose synthesis | Cellulose synthase-like A1 [ *Pinus taeda* ] | 0 |
| ppa001247m | gi|225444055| | NP_181020.2 | -3.20 | 31.1 | cell.organisation | Myosin heavy chain-related [ *Arabidopsis thaliana* ] | 8.00E-122 |
| ppa011110m | gi|296088209| | XP_002462122.1 | -3.21 | 35.2 | not assigned.unknown | Hypothetical protein SORBIDRAFT_02g019520 [ *Sorghum bicolor* ] | 3E-69 |
| ppa022396m | gi|225440737| | NP_850257.1 | -3.26 | 27.3.99 | RNA.regulation of transcription.unclassified | Mitochondrial transcription termination factor-related / mterf-related [ *Arabidopsis thaliana* ] | 3E-83 |
| ppa008395m | gi|225458802| | BAA01091.1 | -3.28 | 29.4 | protein.postranslational modification | Casein kinase II catalytic subunit [ *Arabidopsis thaliana* ] | 0 |
| ppa007415m | gi|255555623| | XP_002518847.1 | -3.31 | 35.2 | not assigned.unknown | N-rich protein, putative [ *Ricinus communis* ] | 0 |
| ppa006762m | gi|224129412| | NP_181981.2 | -3.35 | 23.1.3 | nucleotide metabolism.synthesis.PRS-PP | Ribose-phosphate pyrophosphokinase, putative / phosphoribosyl diphosphate synthetase, putative [ *Arabidopsis thaliana* ] | 0 |
| ppa014249m | gi|255560060| | XP_002879372.1 | -3.35 | 35.2 | not assigned.unknown | Hypothetical protein ARALYDRAFT_482146 [ *Arabidopsis lyrata subsp. Lyrata* ] | 8E-14 |
| ppa002188m | gi|157399680| | ABV53464.1 | -3.36 | 27.3.66 | RNA.regulation of transcription.Psudo ARR transcription factor family | Pseudo-response regulator 5 [ *Castanea sativa* ] | 0 |
| ppa000610m | gi|225456989| | NP_001056507.1 | -3.38 | 35.2 | not assigned.unknown | Os05g0594500 [ *Oryza sativa Japonica Group* ] | 0 |
| ppa010631m | gi|225448313| | NP_001048144.1 | -3.38 | 35.2 | not assigned.unknown | Os02g0753200 [ *Oryza sativa Japonica Group* ] | 2E-55 |
| ppa019447m | gi|255545204| | AAO24543.1 | -3.40 | 35.2 | not assigned.unknown | At3g14190 [ *Arabidopsis thaliana* ] | 0.018 |
| ppa013098m | gi|147810457| | AAC32136.1 | -3.48 | 35.1 | not assigned.no ontology | YGL010w-like protein [ *Picea mariana* ] | 1E-68 |
| ppa007070m | gi|224107669| | NP_176934.2 | -3.54 | 35.2 | not assigned.unknown | DNA binding / nuclease [ *Arabidopsis thaliana* ] | 7E-107 |
| ppa001814m | gi|225438589| | NP_201299.2 | -3.55 | 35.1.1 | not assigned.no ontology.ABC1 family protein | ATATH13; transporter [ *Arabidopsis thaliana* ] | 0 |
| ppa010865m | gi|255581426| | XP_002531521.1 | -3.56 | 18.3.2 | Co-factor and vitamine metabolism.riboflavin.riboflavin synthase | 6,7-dimethyl-8-ribityllumazine synthase, putative [ *Ricinus communis* ] | 7E-125 |
| ppa002536m | gi|255585468| | EOY21411.1 | -3.66 | 30.2.3 | signalling.receptor kinases.leucine rich repeat III | Receptor-like kinase 1 [ *Theobroma cacao* ] | 0 |
| ppa001612m | gi|280967728| | XP_004287384.1 | -3.69 | 33.99 | development.unspecified | Transcriptional corepressor LEUNIG-like [ *Fragaria vesca subsp. Vesca* ] | 0 |
| ppa007329m | gi|225458489| | XP_002510285.1 | -3.74 | 29.3.3 | protein.targeting.chloroplast | Signal peptidase I, putative [ *Ricinus communis* ] | 2E-125 |
| ppa000340m | gi|225427157| | XP_002277547.1 | -3.81 | 34.16 | transport.ABC transporters and multidrug resistance systems | Similar to P-glycoprotein [ *Vitis vinifera* ] | 0 |
| ppa008509m | gi|224131252| | XP_002520695.1 | -4.16 | 26.22 | misc.short chain dehydrogenase/reductase (SDR) | Short chain alcohol dehydrogenase, putative [ *Ricinus communis* ] | 5E-125 |
| ppa018789m | gi|255540579| | XP_002511354.1 | -4.28 | 30.2.11 | signalling.receptor kinases.leucine rich repeat XI | Receptor protein kinase, putative [ *Ricinus communis* ] | 0 |
| ppa016083m | gi|147866710| | CAN78405.1 | -4.50 | 35.2 | not assigned.unknown | Hypothetical protein VITISV_023174 [ *Vitis vinifera* ] | 3E-61 |
| ppa008246m | gi|118486766| | XP_002890650.1 | -7.78 | 23.4.99 | nucleotide metabolism.phosphotransfer and pyrophosphatases.misc | Aspartate/glutamate/uridylate kinase family protein [ *Arabidopsis lyrata subsp. Lyrata* ] | 0 |
| ppa017360m | gi|223972274| | BAH23314.1 | -7.78 | 26.8 | misc.nitrilases, *nitrile lyases, berberine bridge enzymes, reticuline oxidases, troponine reductases | (R)-hydroxynitrile lyase [ *Prunus mume* ] | 0 |
| ppa003822m | gi|224137116| | XP_002892022.1 | -7.78 | 27.3.67 | RNA.regulation of transcription.putative transcription regulator | Pentatricopeptide repeat-containing protein [ *Arabidopsis lyrata subsp. Lyrata* ] | 0 |
| ppa007048m | gi|225457054| | XP_002877589.1 | -7.78 | 27.3.99 | RNA.regulation of transcription.unclassified | Zinc finger family protein [ *Arabidopsis lyrata subsp. Lyrata* ] | 0 |
| ppa004321m | gi|255540859| | XP_002511494.1 | -7.78 | 29.5.9 | protein.degradation.AAA type | ATP binding protein, putative [ *Ricinus communis* ] | 1E-157 |
| ppa005444m | gi|225448485| | XP_002528312.1 | -7.78 | 35.1 | not assigned.no ontology | Glycosyl transferase, putative [ *Ricinus communis* ] | 6E-147 |
| ppa007765m | gi|223452540| | ACM89597.1 | -7.89 | 30.2.11 | signalling.receptor kinases.leucine rich repeat XI | Leucine rich repeat protein [ *Glycine max* ] | 0 |
| ppa010826m | gi|255585255| | BAA02109.1 | -7.89 | 30.50 | signalling.G-proteins | GTP-binding protein [ *Pisum sativum* ] | 3E-100 |
| ppa017381m | gi|225431423| | XP_002280019.1 | -7.89 | 35.1 | not assigned.no ontology | Uncharacterized protein LOC100262676 [ *Vitis vinifera* ] | 0 |
| ppa008255m | gi|225442309| | XP_002521394.1 | -7.99 | 7.1.3 | OPP.oxidative PP.6-phosphogluconate dehydrogenase | 3-hydroxyisobutyrate dehydrogenase, putative [ *Ricinus communis* ] | 1E-90 |
| ppa018095m | gi|224095730| | XP_002530547.1 | -7.99 | 16.8.5.1 | secondary metabolism.flavonoids.isoflavones.isoflavone reductase | Isoflavone reductase, putative [ *Ricinus communis* ] | 0 |
| ppa009756m | gi|255555623| | XP_002518847.1 | -7.99 | 20.2.4 | stress.abiotic.touch/wounding | N-rich protein, putative [ *Ricinus communis* ] | 2E-159 |
| ppa010414m | gi|255537437| | XP_002509785.1 | -7.99 | 26.9 | misc.glutathione S transferases | Glutathione-s-transferase theta, gst, putative [ *Ricinus communis* ] | 0 |
| ppa012090m | gi|297734406| | AAL09401.1 | -7.99 | 29.2.1.2.2.17 | protein.synthesis.ribosomal protein.eukaryotic.60S subunit.L17 | Ribosomal protein [ *Petunia x hybrida* ] | 0 |
| ppa001259m | gi|302143924| | _002526256.1 | -7.99 | 29.3.1 | protein.targeting.nucleus | Importin beta-1, putative [ *Ricinus communis* ] | 0 |
| ppa000215m | gi|255547892| | XP_002515003.1 | -7.99 | 34.16 | transport.ABC transporters and multidrug resistance systems | Multidrug resistance-associated protein 2, 6 (mrp2, 6), abc-transoprter, putative [ *Ricinus communis* ] | 0 |
| ppa000227m | gi|255543331| | XP_002512728.1 | -7.99 | 34.16 | transport.ABC transporters and multidrug resistance systems | ATP-binding cassette transporter, putative [ *Ricinus communis* ] | 0 |
| ppa020695m | gi|296082561| | XP_002276999.1 | -8.08 | 30.1 | signalling.in sugar and nutrient physiology | Glutamate receptor 3.6-like [ *Vitis vinifera* ] | 0 |
| ppa002425m | gi|225438658| | XP_002309991.1 | -8.08 | 34.60 | transport.sulphate | Sulfate/bicarbonate/oxalate exchanger and transporter sat-1 [ *Populus trichocarpa* ] | 0 |
| ppa004227m | gi|255555937| | NP_564388.1 | -8.08 | 35.1 | not assigned.no ontology | Membrane protein, putative [ *Arabidopsis thaliana* ] | 0 |
| ppa004607m | gi|255568313| | XP_002525131.1 | -8.17 | 27.3.52 | RNA.regulation of transcription.Global transcription factor group | Bromodomain-containing protein, putative [ *Ricinus communis* ] | 0 |
| ppa017049m | gi|225447810| | XP_002267129.1 | -8.17 | 30.2.17 | signalling.receptor kinases.DUF 26 | Probable LRR receptor-like serine/threonine-protein kinase At1g56130 [ *Vitis vinifera* ] | 0 |
| ppa003996m | gi|297739205| | NP_171996.2 | -8.17 | 34.2 | transport.sugars | Hexose transporter, putative [ *Arabidopsis thaliana* ] | 0 |
| ppa023881m | - | unkown | -8.17 | 35.2 | not assigned.unknown | Unkown | 0 |
| ppa004516m | gi|255585735| | XP_002533549.1 | -8.25 | 30.3 | signalling.calcium | Calmodulin binding protein, putative [ *Ricinus communis* ] | 0 |
| ppa005153m | gi|224110908| | AAK84952.2 | -8.33 | 35.2 | not assigned.unknown | Putative carboxyl-terminal proteinase [ *Gossypium hirsutum* ] | 0 |
| ppa020243m | gi|2688828| | AAB88878.1 | -8.48 | 17.5.1 | hormone metabolism.ethylene.synthesis-degradation | Ethylene-forming-enzyme-like dioxygenase [ *Prunus armeniaca* ] | 0 |
| ppa004078m | gi|225426453| | AAZ39642.1 | -8.48 | 26.1 | misc.cytochrome P450 | Ytochrome P450 fatty acid omega-hydroxylase [ *Petunia x hybrida* ] | 0 |
| ppa004371m | gi|225424162| | BAJ54084.1 | -8.54 | 21.1.2 | redox.thioredoxin.QSOX | Protein disulfide isomerase family [ *Glycine max* ] | 0 |
| ppa007175m | gi|116268421| | XP_002514038.1 | -8.61 | 20.2.1 | stress.abiotic.heat | Dnajc14 protein, putative [ *Ricinus communis* ] | 1E-105 |
| ppa011874m | gi|255557991| | XP_001916800.1 | -8.61 | 35.2 | not assigned.unknown | Caspase recruitment domain family, member 6 [ *Equus caballus* ] | 2.7 |
| ppa022706m | gi|296082018| | XP_002527539.1 | -8.84 | 10.7 | cell wall.modification | EG45-like domain containing protein [ *Vitis vinifera* ] | 9E-21 |
| ppa000174m | gi|225445069| | XP_002280317.1 | -8.89 | 29.4 | protein.postranslational modification | Similar to protein kinase family protein / WD-40 repeat family protein [ *Vitis vinifera* ] | 0 |
| ppa018514m | gi|255572727| | CAN63004.1 | -11.35 | 35.2 | not assigned.unknown | Hypothetical protein VITISV_004364 [ *Vitis vinifera* ] | 3E-14 |
