# Supplementary material for: Genome-Wide Transcriptional Profile Analysis of Prunus persica in Response to Low Sink Demand after Fruit Removal
Source: Front Plant Sci. 2016 Jun 22;7:883. doi: 10.3389/fpls.2016.00883 (PMC4916340; doi:10.3389/fpls.2016.00883)
Supplement: Table S4 — Alignment of the unique tags to the reference genome and annotated genes from peach leaves under normal sink demand (+fruit) or low sink demand (–fruit) peach trees. [file Table4.DOC]

**Table S4.** Alignment of the unique tags to the reference genome and annotated genes from peach leaves under normal sink demand (+ fruit) or low sink demand (– fruit) peach trees.

| **Tag mapping** | **+ fruit** | **– fruit** |
| --- | --- | --- |
| Tags matched to sense gene |  |  |
| Perfect match | 23 310 (19.73%) | 20 051 (19.13%) |
| 1 bp mismatch | 6 789 (5.74%) | 5 709 (5.45%) |
| Tags matched to antisense gene |  |  |
| Perfect match | 20 863 (17.65%) | 16 956 (16.18%) |
| 1 bp mismatch | 1 385 (1.18%) | 1 147 (1.10%) |
| All tags mapping to genome | 43 079 (36.45%) | 42 274 (40.33%) |
| All tags mapping to gene | 52 347 (44.29%) | 43863 (41.84%) |
| No matched tags | 22 766 (19.26%) | 18 689 (17.83%) |
