# Supplementary material for: Genome-Wide Transcriptional Profile Analysis of Prunus persica in Response to Low Sink Demand after Fruit Removal
Source: Front Plant Sci. 2016 Jun 22;7:883. doi: 10.3389/fpls.2016.00883 (PMC4916340; doi:10.3389/fpls.2016.00883)
Supplement: Table S5 — List of genes selected for Real-time PCR. [file Table5.DOC]

| **Table S5.** List of genes selected for Real-time PCR | | | |  |  |
| --- | --- | --- | --- | --- | --- |
| **Accession NO.** | **BIN** | **Solexa fold** | **RT-PCR fold** | **Species** | **Annotation** |
| ACP19342.1 | 34.7 | -6.58 | -3.1 | *Glycine max* | Phosphate transporter |
| XP_002523967.1 | 29.2.1.1.1.2.3 | -5.48 | -3 | *Ricinus communis* | 50S ribosomal protein L3, putative |
| NP_564904.1 | 30.2.99 | -5.18 | -1.9 | *Arabidopsis thaliana* | Leucine-rich repeat family protein / protein kinase family protein |
| AAF04293.2 | 28.1.1 | -4.86 | -3.7 | *Arabidopsis thaliana* | Fructose-6-phosphate 2-kinase/fructose-2,6-bisphosphatase |
| ACZ52964.1 | 20.1 | 4.62 | 14.7 | *Dimocarpus longan* | Chitinase |
| P30236.1 | 20.2.1 | 5.61 | 33.3 | *Glycine max* | 22.0 kda class IV heat shock protein |
| BAC66141.1 | 26.4.1 | 6.02 | 6.2 | *Fragaria x ananassa* | Beta-1,3-glucanase |
| ACE80957.1 | 20.1 | 8.17 | 29.4 | *Prunus dulcis x Prunus persica* | Putative allergen Pru p 2.01A |
| ACB70176.1 | 20.2.1 | 9.39 | 138.5 | *Capparis spinosa* | Small heat shock protein |
| ABA26457.1 | 20.1 | 13.04 | 25.1 | *Citrullus lanatus* | Acidic class III chitinase |
